# Hydrofluoromethylation of alkenes with fluoroiodomethane and beyond†

**DOI:** 10.1039/d1sc03421a

**Published:** 2021-08-11

**Authors:** Sandrine M. Hell, Claudio F. Meyer, Sebastiano Ortalli, Jeroen B. I. Sap, Xuanxiao Chen, Véronique Gouverneur

**Affiliations:** University of Oxford, Chemistry Research Laboratory 12 Mansfield Road Oxford OX1 3TA UK veronique.gouverneur@chem.ox.ac.uk

## Abstract

A process for the direct hydrofluoromethylation of alkenes is reported for the first time. This straighforward silyl radical-mediated reaction utilises CH_2_FI as a non-ozone depleting reagent, traditionally used in electrophilic, nucleophilic and carbene-type chemistry, but not as a CH_2_F radical source. By circumventing the challenges associated with the high reduction potential of CH_2_FI being closer to CH_3_I than CF_3_I, and harnessing instead the favourable bond dissociation energy of the C–I bond, we demonstrate that feedstock electron-deficient alkenes are converted into products resulting from net hydrofluoromethylation with the intervention of (Me_3_Si)_3_SiH under blue LED activation. This deceptively simple yet powerful methodology was extended to a range of (halo)methyl radical precursors including ICH_2_I, ICH_2_Br, ICH_2_Cl, and CHBr_2_F, as well as CH_3_I itself; this latter reagent therefore enables direct hydromethylation. This versatile chemistry was applied to ^18^F-, ^13^C-, and D-labelled reagents as well as complex biologically relevant alkenes, providing facile access to more than fifty products for applications in medicinal chemistry and positron emission tomography.

## Abstract 2

Herein, we report the direct hydro(halo)methylation of alkenes from a variety of (halo)methyl iodides (including F-18, C-13, D-2 isotopologues), enabling the incorporation of a plethora of C-1 fragments into complex biologically active molecules.

## Graphical abstract

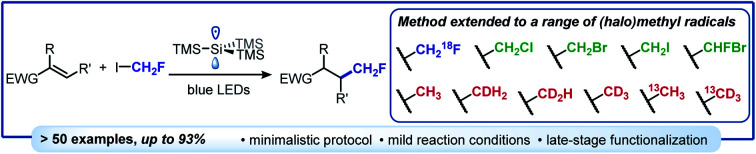

## Introduction

**Scheme 1:**
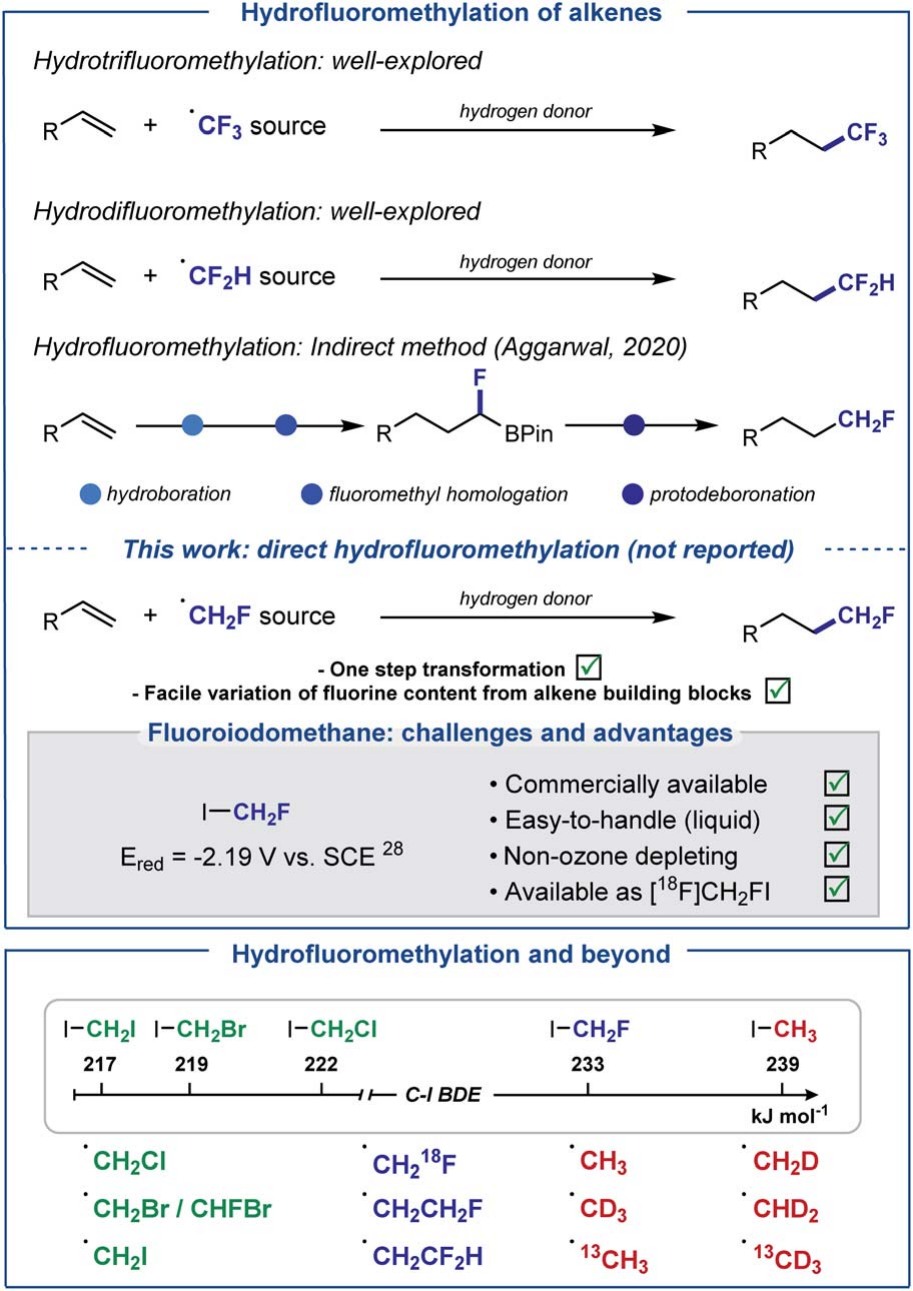
Hydro(per)fluoromethylation of alkenes. This work: direct silyl radical-mediated hydrofluoromethylation of electron-deficient alkenes and extension to numerous hydro(halo)methylation reactions.

The introduction of fluoroalkyl groups has garnered significant interest in medicinal chemistry, enabling the modulation of biological and physicochemical properties of lead candidates for drug discovery.^1–3^ Whilst the fields of radical trifluoromethylation and difluoromethylation have been extensively explored,^4–10^ the fluoromethyl radical has received far less attention.^11–13^ This is unexpected as the fluoromethyl group features frequently in pharmaceutical drugs, more often to improve metabolic stability by serving as a bioisosteric replacement of functional groups responsible for poor performance.^14,15^ In recent years, several reagents for the generation of the CH_2_F radical have been developed.^16–20^ Often, efficient activation of these reagents requires harsh reaction conditions, such as elevated temperatures, strong oxidants, or strong reductants. Furthermore, many of these reagents are either expensive, highly toxic or non-commercial, requiring multistep syntheses for their preparation. As part of our growing interest in developing “minimalistic” procedures for the late-stage hydrofluoroalkylation of alkene-containing biologically active molecules,^21–23^ we sought to develop an operationally simple method for the direct hydrofluoromethylation of alkenes, as an attractive strategy for the introduction of this motif to C(sp^3^)-enriched backbones (Scheme 1).

In 2020, an indirect method for the hydrofluoromethylation of alkenes was developed by Aggarwal and co-workers;^13^ this elegant multi-step procedure starts with the conversion of alkenes into boronic esters, subsequent treatment at low temperature (−78 °C) with *in situ* formed fluoroiodomethyl lithium to generate fluoroboronic esters, and a final protodeboronation. Our aim was to develop a one-step method that avoids operational complexity and over-engineering, ideally using fluoroiodomethane which is a non-ozone depleting, easy to handle and inexpensive commercial CH_2_F radical precursor. We noted that fluoroiodomethane has found applications as an electrophilic or nucleophilic fluoromethylation reagent as well as in cross-coupling reactions,^24–27^ but has not been explored in the context of radical chemistry.

The high reduction potential of CH_2_FI (*E*_red_ = −2.19 V *vs.* saturated calomel electrode (SCE) in MeCN),^28^ much closer to MeI (*E*_red_ = −2.39 V *vs.* SCE in MeCN)^28^ than CF_3_I (*E*_red_ = −1.22 V *vs.* SCE in MeCN),^29^ encouraged the implementation of an activation pathway exploiting instead the favourable bond dissociation energy (BDE) of C–I (BDE (FH_2_C–I) = 233 kJ mol^−1^) *versus* C–F (BDE (IH_2_C–F) = 460 kJ mol^−1^).^30^ Since the pioneering work of Chatgilialoglu,^31^ tris(trimethylsilyl)silane (TTMSS) has found ample applications as a powerful tool for mild radical generation *via* the activation of alkyl halides.^32–36^ In addition, TTMSS has valuably complemented Giese-type reactions, a commonly exploited platform for late-stage functionalisation, by providing a suitable alternative to traditional toxic tin-based reagents.^37^ Consequently, we envisioned that the supersilyl radical (TMS)_3_Si˙ would be well suited to release ˙CH_2_F from CH_2_FI. Subsequent Giese-type addition of ˙CH_2_F to the electron-deficient alkene would generate a carbon-centered radical intermediate. Hydrogen-atom transfer (HAT) between this electrophilic species and hydridic (TMS)_3_SiH would afford the desired hydrofluoromethylated product, and (TMS)_3_Si˙ entering chain propagation. Initiation for this process would be triggered by photolytic C–I cleavage of FH_2_C–I.^38^ This method offers the prospect of being applicable to a range of other halo-containing alkyl radicals, provided that competitive hydrogen atom abstraction with (TMS)_3_SiH does not occur prior to Giese addition. Herein, we report the realisation of this strategy with a wide range of haloiodomethanes for the direct hydrohalomethylation of electron-deficient alkenes including biologically relevant molecules. The method was extended to ^18^F-hydrofluoromethylation and hydromethylation with iodomethane along with five of its D and ^13^C isotopomers.

## Results and discussion

Preliminary experiments were conducted with *N*-phenyl acrylamide (**1a**) (Table 1).^39^ Various combinations of silanes and solvents revealed that the desired hydrofluoromethylated product (**2a**) was obtained in 71% with (TMS)_3_SiH in MeCN at room temperature under blue light irradiation for 16 h (entry 1).^40^ The addition of *fac*-Ir(ppy)_3_ (0.5 mol%) did not lead to significant improvement (entry 2). The simpler protocol was therefore retained for further investigations. Control experiments indicate that the reaction was not effective in absence of light (entry 3), and unsuccessful in absence of silane or in presence of the radical scavenger TEMPO (entries 4 and 5). No deuterium incorporation was observed in the product when the reaction was performed in CD_3_CN.^40^ These data corroborate our proposed radical chain propagation mechanism, initiated by blue-light homolysis of the CH_2_F–I bond.^38^ Giese addition of the fluoromethyl radical to an electron-deficient alkene furnishes an electrophilic carbon-centered radical intermediate, capable of undergoing HAT with (TMS)_3_SiH. The resulting silyl radical enables chain propagation by abstracting iodine from CH_2_FI to afford (TMS)_3_SiI along with ˙CH_2_F.^40^

**Table 1:** Hydrofluoromethylation of *N*-phenyl acrylamide **1a**^a^

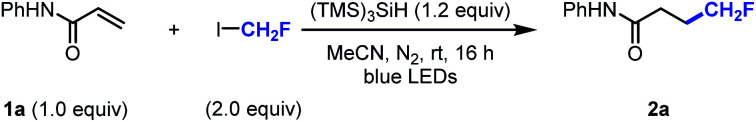		
Entry	Deviations from standard conditions	Yield (%)
1	None	71
2	With *fac*-Ir(ppy)_3_ (0.5 mol%)	75
3	No light	Traces
4	No silane	0
5	With TEMPO (4.0 equiv.)^b^	0

a Reaction conditions: CH_2_FI (0.2 mmol), **1a** (0.1 mmol), (TMS)_3_SiH (0.12 mmol), solvent (0.6 mL) under blue light (*λ*_max_ = 450 nm) irradiation for 16 h. Yields of isolated products.

b Addition of 4.0 equiv. of TEMPO ((2,2,6,6-tetramethylpiperidin-1-yl)oxyl). TEMPO-CH_2_F was observed in quantitative ^19^F NMR analysis of the crude reaction mixture using α,α,α-trifluorotoluene as an internal standard. *fac*-Ir(ppy)_3_ = tris[2-phenylpyridinato-C2,*N*]iridium(iii).

**Scheme 2:**
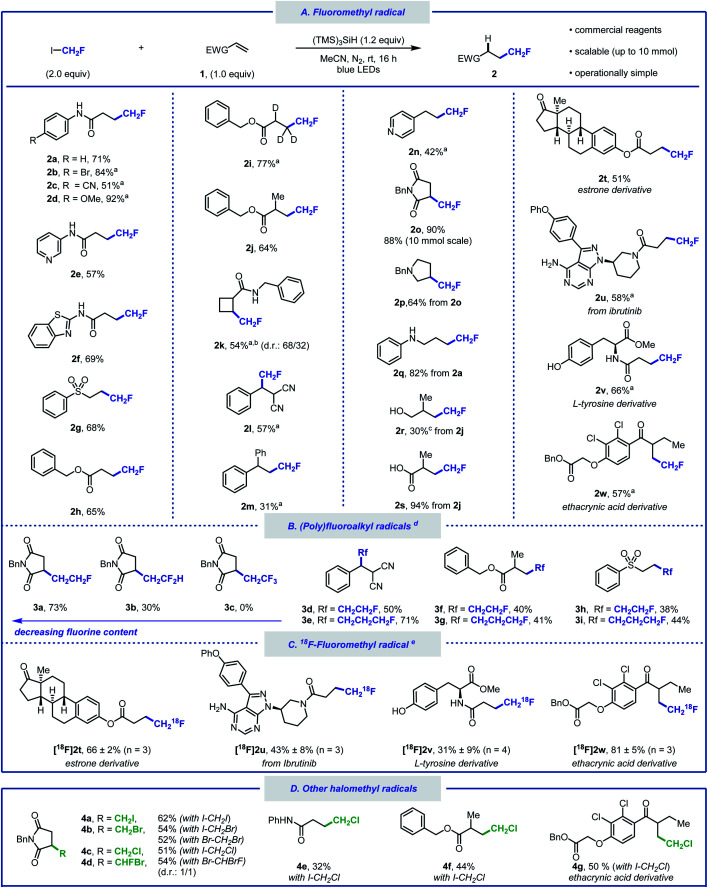
Substrate scope of the hydro(halo)methylation of electron-deficient alkenes. Reaction conditions: alkene (0.5 mmol), (TMS)_3_SiH (0.6 mmol), radical precursor (1.0 mmol), MeCN (3.0 mL), N_2_ atmosphere, blue light (*λ*_max_ = 450 nm) irradiation for 16 h, room temperature. (a) Reaction performed with *fac*-Ir(ppy)_3_ (0.5 mol%). (b) Product isolated as a mixture of diastereomers. (c) Yield determined by quantitative ^1^H NMR using triphenylmethane as an internal standard. (d) Reaction performed using (poly)fluoroalkyl iodide. (e) ^18^F-Hydrofluoromethylation performed with [^18^F]CH_2_FI. Reaction conditions: [^18^F]CH_2_FI (10 MBq), alkene (0.1 mmol), (TMS)_3_SiH (0.12 mmol), *fac*-Ir(ppy)_3_ (0.5 mol%), DMF (0.6 mL), blue light (*λ*_max_ = 450 nm) irradiation for 20 min, room temperature.

With the optimised reaction conditions in hand, we sought to explore the scope of this hydrofluoromethylation protocol (Scheme 2A). Various functional groups, such as methoxy, nitrile, halide, ketone, ether, amide, ester, aniline, and sulfone were tolerated. The addition of *fac*-Ir(ppy)_3_ (0.5 mol%) led to higher yields for selected substrates.^40^*N*-Aryl acrylamides bearing electron-withdrawing and electron-donating groups afforded the desired products in moderate to excellent yields (**2a–d**). The hydrofluoromethylation of *N*-heteroaryl acrylamides, such as pyridyl and benzothiazyl was also successful (**2e**, **2f**). Alkenes substituted with sulfones and esters were competent substrates generating **2g** and **2h** in moderate yield. As deuteration can improve metabolic stability,^41^ we investigated the hydrofluoromethylation of a deuterated alkene (**1i**) that was successfully converted into [*D*_3_]**2i**. The *gem*-disubstituted alkene **1j** provided **2j** in 64% yield. Pleasingly, the internal alkene **1k** was reactive under our reaction conditions and afforded fluoromethylcyclobutane **2k** in moderate yield. This result is significant as 1,2-disubstituted fluoroalkyl cyclobutanes currently require multiple steps for their preparation.^42^ A non-cyclic trisubstituted alkene afforded the product in 57% yield (**2l**). Styrene derivatives such as **1m** and **1n** afforded the desired products in synthetically useful yields (**2m**, **2n**). Our protocol is amenable to scale-up as demonstrated by the 10 mmol scale hydrofluoromethylation of *N*-benzylmaleimide affording **2o** in 88% yield. The synthesis of fluorinated pyrrolidine **2p**, amine **2q**, alcohol **2r** and carboxylic acid **2s** was performed in two steps, offering a pathway to diversify the range of products within reach from CH_2_FI. The late-stage hydrofluoromethylation of complex biologically active molecules was considered next. The anti-cancer drug ibrutinib as well as estrone, tyrosine and ethacrynic acid derivatives afforded the desired hydrofluoromethylated products in good yields (**2t–w**). The tolerance of functional groups was investigated with a robustness screening.^40^ These experimental data provide an overview of the many heteroarenes (*e.g.* pyridazine, 1,3,5-triazine, indole, benzothiazole or oxazole) that are tolerated under the optimised reaction conditions.

Whilst additives containing nucleophilic functional groups such as alcohols and anilines were tolerated, side reactivity arising from nucleophilic substitution was observed.^40^ Competitive alkylation was suppressed when using 1.0 equivalent of CH_2_FI, albeit at the expense of reduced yield for the hydrofluoromethylated product. Aliphatic amines were tolerated but yields did not exceed 30%.^40^ The hydrofluoromethylation of alkenes not bearing electron-withdrawing groups was possible albeit significantly less efficient.^40^ With a protocol relying on the favourable C–I bond dissociation energy and considering the importance of homologation in medicinal chemistry,^43^ we considered the generation of products from a series of homologated fluoroiodoalkanes (Scheme 2B).^44,45^ Hydrofluoroalkylation of alkenes **1g**, **1j** and **1l** provided effortlessly the homologous series of products **3d–i**. Specifically, the fluoroethyl radical was efficiently generated applying similar silyl radical activation, and **3a** was isolated in good yield. The introduction of the fluoroethyl radical was successfully performed on linear terminal, *gem*-disubstituted, and trisubstituted alkenes (**3d**, **3f**, **3h**). The method was further extended to fluoroiodopropane as shown with the synthesis of **3e**, **3g**, and **3i**. Precursors featuring additional fluorine atoms were less suitable with the difluoroethylated product **3b** isolated in 30%, and no product observed when attempting to prepare the hydrotrifluoroethylated product **3c**. Increased fluorine content enhances radical electrophilicity, thereby encouraging undesired H-atom abstraction from (TMS)_3_SiH.^40^

Given the success of our protocol, we further investigated the applicability of our method for the generation of [^18^F]CH_2_F radical from [^18^F]CH_2_FI (Scheme 2C).^46–48^ Compounds labelled with the radioisotope F-18 are important for applications in Positron Emission Tomography (PET).^49–53^ The synthesis of [^18^F]CH_2_FI in high molar activity (*A*_m_) is well-established and has been automated.^54–57^ To date, this labelled reagent is mainly employed for the electrophilic ^18^F-fluoromethylation of phenols.^58,59^ We now demonstrate that [^18^F]CH_2_FI is well suited for [^18^F]CH_2_F radical chemistry. Specifically, Ibrutinib, an estrone, a tyrosine, and an ethacrynic acid derivative underwent ^18^F-hydrofluoromethylation in radiochemical yields up to 81% ([^18^F]**2t–w**). This reaction was best performed for 20 minutes at ambient temperature in the presence of *fac*-Ir(ppy)_3_ under blue-light irradiation. This method offers an alternative to nucleophilic ^18^F-fluorination with [^18^F]fluoride for precursors that are either unstable, require complex multiple steps synthesis, or lead predominantly to elimination products. Haloiodomethanes other than fluoroiodomethane were also considered as they would allow for the one-step introduction of reactive halomethyl groups to alkenes (Scheme 2D). Controlled activation of reagents such as ICH_2_X (X = Cl, Br, I) would enable their use for example as ˙CH_2_^+^ synthon. To date, only few examples for the generation and use of halomethyl radicals have been reported.^60–64^ When diiodomethane was employed under the standard reaction conditions, *N*-benzylmaleimide underwent hydroiodomethylation in 62% yield (**4a**). Similarly, hydrobromomethylation (from dibromomethane or bromoiodomethane), hydrochloromethylation (from chloroiodomethane), and hydrobromofluoromethylation (from dibromofluoromethane) provided the corresponding halomethyl alkanes in moderate yields (**4b–4d**).^23,65^ Other alkenes afforded the hydrochloromethylated products in moderate yields (**4e–4g**). Although full conversion of starting material was observed for these reactions, purification *via* silica gel chromatography led to elimination, which is reflected in the lower yield for these compounds upon isolation.

**Scheme 3:**
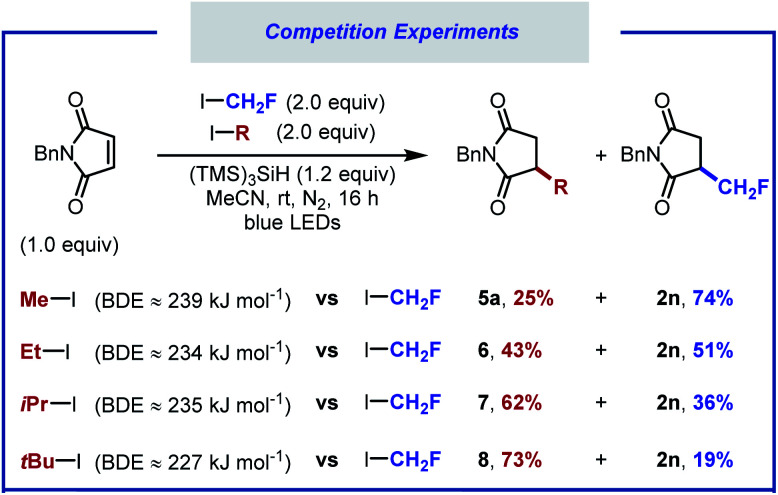
Competition experiments with equimolar amounts of different alkyl iodides.

Competition experiments were performed to calibrate the reactivity of fluoroiodomethane *versus* other alkyl iodides (Scheme 3). When equimolar amounts of iodomethane and fluoroiodomethane were subjected to the standard reaction conditions, product resulting from fluoromethyl radical addition was obtained in 74% yield (**2n**), along with 25% of the hydromethylated product **5a**. When the reaction was carried out with equimolar amounts of iodoethane, products **2n** and **6** were formed in close to 1 : 1 ratio. Additional competition experiments showed that the *iso*-propyl and *tert*-butyl adducts (**7**, **8**) were formed preferentially over the hydrofluoromethylated product. The reactivity of these alkyl iodides therefore decreases in the following order: *t*BuI > iPrI > CH_2_FI ∼ EtI > MeI.

**Scheme 4:**
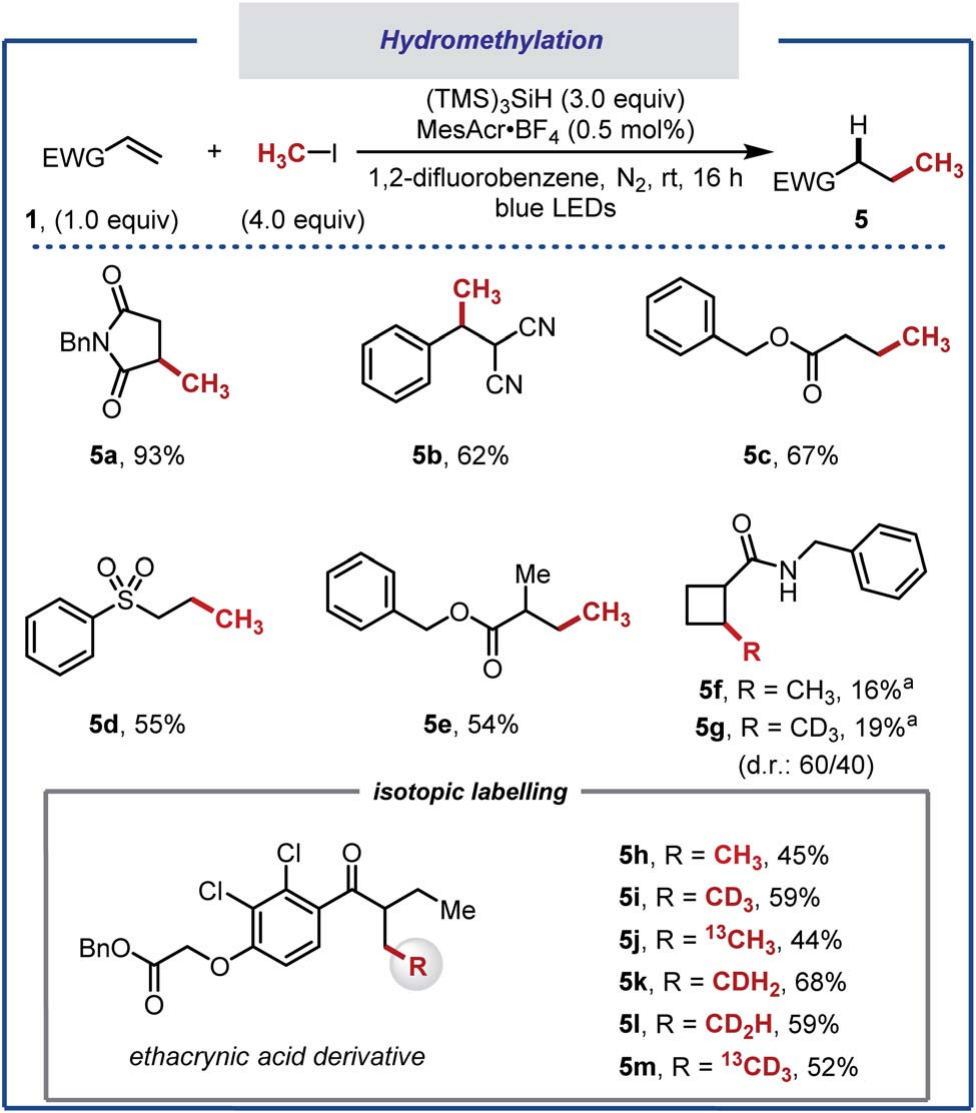
Hydromethylation of electron-deficient alkenes. Reaction conditions: alkene (0.5 mmol), (TMS)_3_SiH (1.5 mmol), iodomethane (2.0 mmol), 1,2-difluorobenzene (3.0 mL), N_2_ atmosphere, blue light (*λ*_max_ = 450 nm) irradiation for 16 h, room temperature. (a) Product isolated as a mixture of diastereomers.

A notable outcome of this study was the observation that net methane addition across the double bond took place with iodomethane. Currently, protocols for the generation of the methyl radical from iodomethane (BDE_CH3–I_ = 239 kJ mol^−1^, *E*_red_ = −2.39 V *vs.* SCE in MeCN) remain underdeveloped.^34,66^ In recent years, the methyl radical has been generated from numerous precursors.^67–71^ The formation of the methyl radical often requires harsh reaction conditions, limiting the applicability of these protocols. Furthermore, the use of the methyl radical towards application to isotopic labelling is far from trivial. Iodomethane, on the other hand, can provide effortless access to a variety of useful isotopologues that would otherwise be beyond reach. The straightforwardness of our protocol prompted us to optimise the hydromethylation of alkenes using iodomethane as methyl radical precursor (Scheme 4). We noted significant gas release when applying our reaction conditions, attributed to methane resulting from competitive HAT between the methyl radical and MeCN (BDE_NCCH2–H_ = 389 kJ mol; BDE_CH3–H_ = 439 kJ mol^−1^).^69^ A screen of solvents, reactants stoichiometry and photocatalysts allowed for hydromethylation to occur in up to 93% yield (**5a**).^40^ Under the optimised reaction conditions consisting of 4.0 equivalents of MeI, 3.0 equivalents of (TMS)_3_SiH and 1,2-difluorobenzene as solvent, in combination with photocatalyst MesAcrBF_4_ (0.5 mol%), the hydromethylation of various alkenes took place in good to excellent yield (**5b–f**). Considering that bioactive compounds containing stable heavy isotopes are useful for example as mass spectroscopy standards,^41,72^ the hydromethylation of an ethacrynic acid derivative was performed with CH_3_I, CH_2_DI, CHD_2_I, CD_3_I, ^13^CH_3_I, and ^13^CD_3_I. All six isotopologues (**5h–5m**) were obtained in moderate yield.^73–75^

## Conclusions

In conclusion, the first direct hydrofluoromethylation of a broad range of electron-deficient alkenes has been developed using fluoroiodomethane. Mechanistically, the process harnesses known principles; so its core value is rooted in its immediate synthetic power. With the current global necessity “to do more with less”, this minimalistic and mild chemical method stands out as it is operationally simple with the supersilyl radical precursor (TMS)_3_SiH being the only chemical required in addition to the reaction partners. The mild reaction conditions are compatible with complex biologically active molecules such as Ibrutinib. The methodology was successfully adapted for the ^18^F-labelling of complex alkenes, and offers a new C–CH_2_^18^F disconnection strategy for radiotracer development. The method was extended to additional fluoroiodoalkanes enabling facile product homologation, as well as multiple (halo)methyl radicals including the methyl radical itself and five of its D and ^13^C isotopomers.

## Data availability

The datasets supporting this article have been uploaded as part of the ESI.†

## Author contributions

S. M. H., C. F. M., S. O. and J. B. I. S. performed the experiments and analysed the results. C. F. M. and X. C. performed the cyclic voltammetry experiments. S. M. H., C. F. M., S. O., J. B. I. S. and V. G. designed the project and wrote the manuscript.

## Conflicts of interest

There are no conflicts to declare.

## Supplementary Material

SC-012-D1SC03421A-s001
