# Supplementary material for: Hydrofluoromethylation of alkenes with fluoroiodomethane and beyond
Source: Chem Sci. 2021 Aug 11;12(36):12149–55. doi: 10.1039/d1sc03421a (PMC8457377; doi:10.1039/d1sc03421a)
Supplement: SC-012-D1SC03421A-s001 [file SC-012-D1SC03421A-s001.pdf]

Electronic Supplementary Material (ESI) for Chemical Science.
This journal is © The Royal Society of Chemistry 2021

Supporting Information

Hydrofluoromethylation of Alkenes with Fluoroiodomethane and Beyond

Sandrine M. Hell,[§,†] Claudio F. Meyer,[§,†] Sebastiano Ortalli,[§,†] Jeroen B. I. Sap,[†] Xuanxiao Chen,[†] Véronique Gouverneur*,[†]

[†] University of Oxford, Chemistry Research Laboratory, 12 Mansfield Road, Oxford, OX1 3TA (United-Kingdom)

[§] These authors contributed equally to this work

Table of contents:

1. General information S3

2. Starting material synthesis S4

3. Cyclic Voltammetry of CH_2FI and CH_3I S6

4. Optimization of the reaction conditions S8

 4.1. Hydrofluoromethylation of electron-deficient alkenes S8

 4.2. Hydromethylation of electron-deficient alkenes S11

5. Mechanistic investigations S15

6. Proposed mechanisms S18

7. Kinetic profile S19

8. Scale-up experiment S21

9. Robustness screen S23

10. Further functionalizations S27

11. ^{18}F-Hydrofluoromethylation of alkenes S31

12. Competition experiments S34

13. Limitations S37

14. Isolation of $(TMS)_3SiOH$ S39

15. General Procedures S40

16. Characterizations S41

 16.1. Hydrofluoromethylation of electron-deficient alkenes S41

 16.2. Hydro(poly)fluoroalkylation of electron-deficient alkenes S52

 16.3. Hydrohalomethylation of electron-deficient alkenes S56

 16.4. Hydromethylation of electron-deficient alkenes S60

17. References S67

18. Spectra S68

1. General information

All NMR spectra were recorded on a Bruker AVIIIHD 400 spectrometer with standard pulse sequences operating at 400 MHz, using $CDCl_3$ as solvent. ^{1}H and ^{13}C NMR spectral data are reported as chemical shifts (δ) in parts per million (ppm) relative to the solvent peak using the Bruker internal referencing procedure (edlock). ^{19}F NMR spectra are referenced relative to $CFCl_3$ in $CDCl_3$. Coupling constants (J) are measured in hertz (Hz). The following abbreviations are used to describe multiplicities s=singlet, d=doublet, t=triplet, q=quartet, quint=quintet, m=multiplet, dd=doublet of doublets, dt=doublet of triplets, td=triplet of doublets, tt=triplet of triplets. NMR spectra were processed with MestReNova 11.0 or higher. High resolution mass spectra (HRMS, m/z) were recorded on a Thermo Exactive HighResolution Orbitrap FTMS instrument equipped with Waters Acquity liquid chromatography system using either the heated electrospray (HESI-II) probe for positive electrospray ionization (ESI+) or the atmospheric pressure chemical ionization (APCI) probe. Melting points of solids were measured on a Griffin apparatus and are uncorrected. Infrared spectra were recorded as the neat compound or in solution using a Bruker tensor 27 FT-IR spectrometer. Absorptions are reported in wavenumber (cm^{-1}). IUPAC names were obtained using the ChemDraw service. Weighing was performed with a 4 decimal place balance. All reactions for the hydrofluoromethylation of electron-deficient alkenes were conducted in non-dried glassware with magnetic stirring. All solvents were used as received without further purification. $(TMS)_3SiH$ was purchased from Fluorochem. Fluoroiodomethane was purchased from abcr. All commercially available substrates were purchased from commercial suppliers or otherwise synthesized according to literature. Reactions were performed in 7 mL vials in a EvoluChemTM PhotoRedOx Box with a 18W blue LED lamp (λ = 450 nm). The yields were determined by isolation on SiO_2 gel column chromatography.

2. Starting material synthesis

Compounds **1a**, **1b**, **1c**, **1d**, **1e**, **1f**, **1l**, **1r**, and **1u** were synthesised according to literature procedures and spectroscopic data were in accordance with previous reports.[1–6]

Benzyl acrylate-*d₃* (1i)

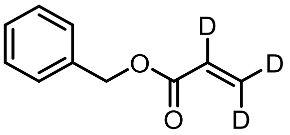

Benzyl bromide (143 uL, 1.2 mmol, 1.2 equiv) was added dropwise to a stirred suspension of acrylic-2,3,3-d_3 acid (75 mg, 1.0 mmol, 1.0 equiv) and K_2CO_3 (387 mg, 2.8 mmol, 2.8 equiv) in DMF (6.0 mL) at 0 °C. The reaction mixture was stirred at room temperature for 16 hours, then diluted with Et_2O and washed with an aqueous solution of saturated $NaHCO_3$. The organic layer was then washed with an aqueous solution of LiCl (10% w/w) five times. The organic phases were combined, dried (Na_2SO_4), filtered and evaporated *in vacuo*. The residue was purified by column chromatography (silica, Et_2O in pentane 0/100 to 100/0) to yield the desired product as a colourless oil in quantitative yield.

^{1}H NMR (400 MHz, $CDCl_3$) δ 7.46 – 7.29 (m, 5H), 5.23 (s, 2H); **^{13}C NMR** (101 MHz, $CDCl_3$) δ 166.1, 136.0, 131.1 – 129.7 (m), 128.9 – 127.4 (m), 128.6, 128.3, 128.3, 66.3. The compound did not ionize; **IR** (neat) 3035, 2955, 1718, 1565, 1498, 1455, 1375, 1225, 1073, 1015, 965, 918, 814, 749, 716, 696, 615.

Benzyl 2-(2,3-dichloro-4-(2-methylenebutanoyl)phenoxy)acetate (1w)

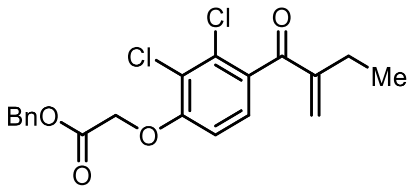

Benzyl bromide (143 uL, 1.2 mmol, 1.2 equiv) was added dropwise to a stirred suspension of 2-(2,3-dichloro-4-(2-methylenebutanoyl)phenoxy)acetic acid (302.0 mg, 1.0 mmol, 1.0 equiv) and K_2CO_3 (387 mg, 2.8 mmol, 2.8 equiv) in DMF (6.0 mL) at 0 °C. The reaction mixture was stirred at room temperature for 16 hours, then diluted with Et_2O and washed with an aqueous solution of saturated $NaHCO_3$. The organic layer was then washed with an aqueous solution of LiCl (10% w/w) five times. The organic phases were combined, dried (Na_2SO_4), filtered and evaporated *in vacuo*. The desired product was obtained as a colourless oil in quantitative yield, and used as such in the next step.

^{1}H NMR (400 MHz, $CDCl_3$) δ 7.40 – 7.30 (m, 5H), 7.09 (d, J = 8.5 Hz, 1H), 6.73 (d, J = 8.5 Hz, 1H), 5.93 (t, J = 1.5 Hz, 1H), 5.58 (s, 1H), 5.25 (s, 2H), 4.79 (s, 2H), 2.47 (qdd, J = 7.4, 1.5, 0.9 Hz, 2H), 1.15 (t, J = 7.4 Hz, 3H); **^{13}C NMR** (101 MHz, $CDCl_3$) δ 195.9, 167.7, 155.5, 150.3, 135.0, 134.1, 131.6, 128.8, 128.8, 128.7, 128.6, 126.9, 123.5, 110.9, 67.5, 66.4, 23.6, 12.5; **HRMS** (ESI-TOF) calculated for $C_{20}H_{19}{}^{35}Cl_2O_4$ $[M+H]^+$: 393.0655; found 393.0654; **IR** (neat) 2969, 1757, 1665, 1585, 1498, 1468, 1439, 1384, 1339, 1293, 1259, 1190, 1122, 1078, 1001, 946, 893, 804, 753, 697, 634.

3. Cyclic Voltammetry

The cyclic voltammetry (CV) measurements were conducted at room temperature using an Autolab PGSTAT204 potentiostat (Metrohm, The Netherlands). Experiments were performed using a standard three-electrode setup, comprising a glassy carbon working electrode (diameter 3.0 mm, CH Instrument), an $Ag/AgNO_3$ (0.01 M) reference electrode and a Pt wire counter electrode. Before each measurement, the working electrode was mechanically polished with alumina slurries in the size 1.0 µm, 0.3 µm and 0.05 µm (purchased from Buehler Ltd, USA) respectively. All CV experiments were carried out at a concentration of 3 mM in anhydrous CH_3CN with an electrolyte $LiClO_4$ (0.1 M) at 100 mV/s scan rate. Solutions containing the compound of interest were thoroughly degassed with argon to remove oxygen prior to any measurement. The reduction potentials were measured against a ferrocene/ferrocenium (Fc/Fc^+) internal reference, and then converted to V vs. SCE (saturated calomel electrode) using the conversion $E°_{1/2}$ (Fc/Fc^+) = + 0.400 V vs. SCE.

Background:

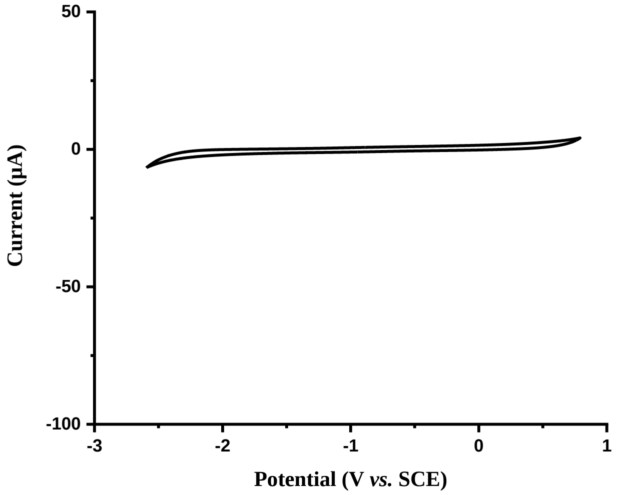

Figure S1. Voltammetric response of 0.1 M $LiClO_4$ in anhydrous CH_3CN.

Fluoroiodomethane: E (V *vs*. SCE) = − 2.19 V

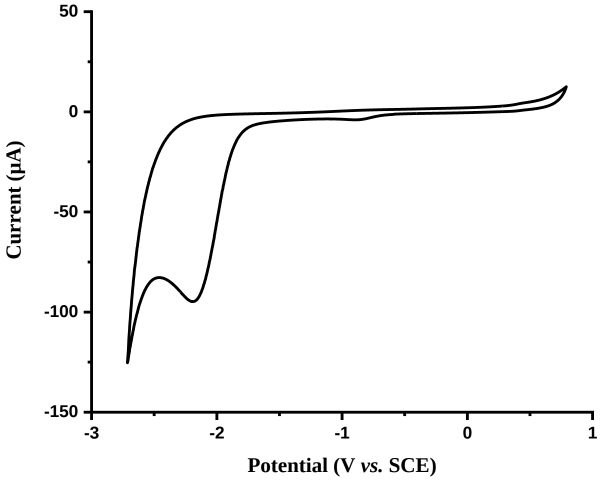

Figure S2. Voltammetric response of 3 mM fluoroiodomethane in anhydrous CH_3CN and 0.1M $LiClO_4$.

Iodomethane: E (V *vs*. SCE) = − 2.39 V

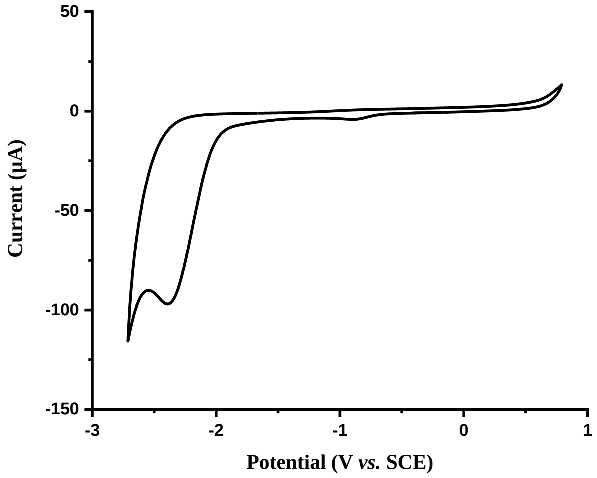

Figure S3. Voltammetric response of 3 mM methyl iodide in anhydrous CH_3CN and 0.1M $LiClO_4$.

4. Optimization of the reaction conditions

4.1 Hydrofluoromethylation of the electron-deficient alkenes

Procedure for the optimization studies:

To a 7 mL vial equipped with a stir bar was added photocatalyst (0.5 mol%), *N*-phenylacrylamide (14.7 mg, 0.1 mmol, 1.0 equiv.), solvent (dry; 0.6 mL), hydrogen atom donor (HAD) (given equivalents), fluoroiodomethane (CH_2FI) (given equivalents). The vial was sealed with a septum, degassed with nitrogen bubbling for 10 seconds and wrapped with parafilm. The reaction mixture was stirred in a EvoluChemTM PhotoRedOx Box with a 18W blue LED lamp (λ = 450 nm) at room temperature (using a fan) for 16 hours. *α,α,α*-Trifluorotoluene (internal standard, 123 µL, 1.0 mmol) was added, and the reaction mixture was analyzed by quantitative ^{19}F NMR.

Table S1: Screening of photocatalysts

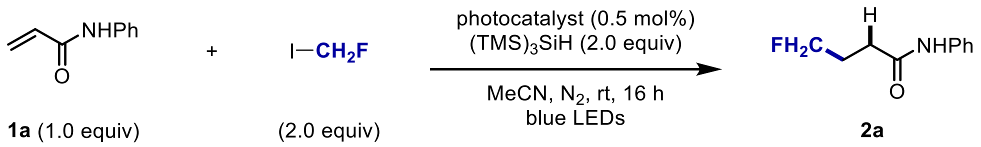

entry	photocatalyst	yield (2a)
**1**	Eosin Y	56%
**2**	*fac*-Ir(ppy)$_3$	75%
**3**	MesAcrBF$_4$	73%
**4**	Benzophenone	75%
**5**	-	71%

We initially investigated our hydrofluoromethylation protocol of **1a** using the reaction conditions we developed previously.[7] Employing Eosin Y as the photocatalyst, $(TMS)_3SiH$ as the HAD and MeCN as the solvent, the desired hydrofluoromethylated product **2a** was obtained in 56% yield. Alternative photocatalysts such as MesAcrBF$_4$, *fac*-Ir(pppy)$_3$ and benzophenone resulted in higher yields. A control experiment revealed that the reaction is also efficient in absence of photocatalyst.

Table S2: Screening of solvents

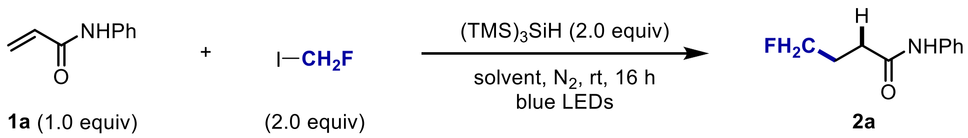

entry	solvent	yield (2a)
**1**	MeCN	71%
**2**	DCE	60%
**3**	THF	55%
**4**	DMF	68%
**5**	DMSO	0%
**6**	$MeNO_2$	17%
**7**	$PhCF_3$	6%

The reaction was successful in other polar and non-polar aprotic solvents, such as *N*,*N*-dimethylformamide (DMF), dichloroethane (DCE) or tetrahydrofuran (THF), but in all cases, lower yields were obtained compared to MeCN. The use of DMSO, nitromethane ($MeNO_2$) or trifluorotoluene ($PhCF_3$) resulted in low to no conversion towards the desired product. MeCN was chosen as the optimum solvent for this transformation, with DMF being a viable alternative for less soluble substrates.

Table S3: Screening of hydrogen-atom donors

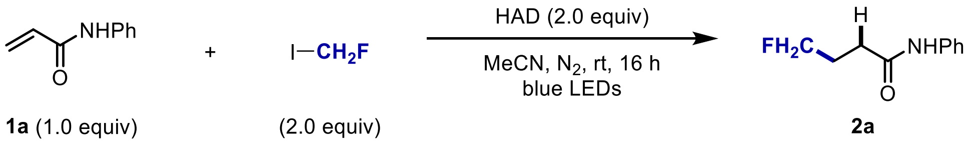

entry	HAD	yield (2a)
**1**	$(TMS)_3SiH$	71%
**2**	Ph_3SiH	0%
**3**	Et_3SiH	traces
**4**	Hantzsch ester	7%
**5**	Ph_3CH	0%

No other silanes or HAD allowed for efficient hydrofluoromethylation of electron-deficient alkenes.

Table S4: Screening of reagent equivalents

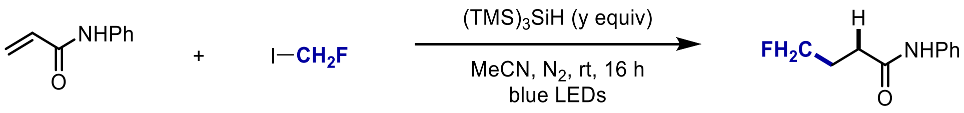

1a (1.0 equiv) (x equiv) **2a**

entry	$(TMS)_3SiH$ (y equiv)	CH_2FI (x equiv)	yield (2a)
**1**	2.0	2.0	71%
**2**	1.5	2.0	75%
**3**	1.2	2.0	76%
**4**	1.2	1.5	68%
**5**	1.2	1.0	64%

The equivalents of $(TMS)_3SiH$ could be reduced to 1.2 equivalents, with no drop in yield. Furthermore, 2.0 equivalents of CH_2FI were shown to be optimal for this hydrofluoromethylation protocol.

4.2 Hydromethylation of the electron-deficient alkenes

Procedure for the optimization studies:

To a 7 mL vial equipped with a stir bar was added photocatalyst (0.5 mol%), solvent (dry; 3.0 mL), benzyl acrylate (81.1 mg, 0.5 mmol, 1.0 equiv), $(TMS)_3SiH$ (given equivalents) and iodomethane (given equivalents). The vial was sealed with a septum, degassed with nitrogen bubbling for 10 seconds and wrapped with parafilm. The reaction mixture was stirred in a EvoluChemTM PhotoRedOx Box with a 18W blue LED lamp (λ = 450 nm) at room temperature (using a fan) for 16 hours. The reaction mixture was transferred to a flask containing triphenylmethane (122 mg, 0.5 mmol). The mixture was concentrated *in vacuo* and analyzed by quantitative ^{1}H NMR.

Note I: The yields might be slightly overestimated due to overlapping peaks. Therefore, these yields should be interpreted cautiously. The yields of isolated products are more accurately displaying the efficiency of this reaction.

Note II: Please carefully read the material safety data sheets (MSDS) of solvents and reagents used in this protocol. Iodomethane has been shown to be toxic and carcinogenic.

Table S5. Iodomethane equivalents

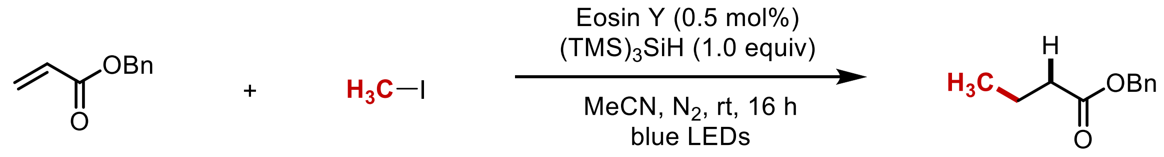

1h (1.0 equiv) (x equiv) **5c**

entry	MeI (x equiv)	yield (5c)
**1**	2.0	20%
**2**	4.0	28%
**3**	6.0	29%

The amount of iodomethane did not drastically affect the yield of the desired product. On the other hand, we noted extensive gas formation during the reaction. We attributed this to the potential unproductive H-atom transfer from the solvent to the methyl radical resulting in the

formation of methane gas. To circumvent that pathway, an extensive solvent screen was performed.

Table S6. Solvent screen

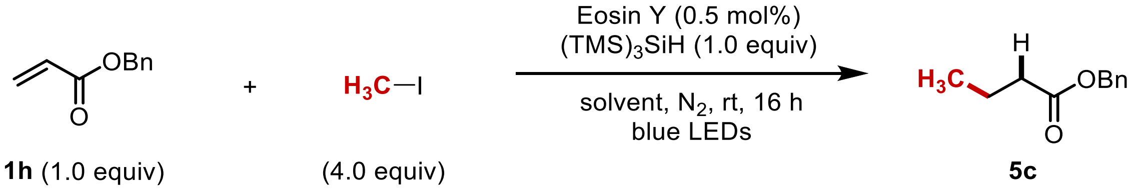

entry	solvent	yield (5c)
**1**	Acetonitrile	28%
**2**	1,2-Dichloroethane	37%
**3**	Water	21%
**4**	Tetrahydrofuran	27%
**5**	Hexane	31%
**6**	Cyclohexane	31%
**7**	Benzene	45%
**8**	Chlorobenzene	41%
**9**	Fluorobenzene	44%
**10**	1,2-Dichlorobenzene	46%
**11**	1,2-Difluorobenzene	48%

As expected, the reaction proceeds best in solvents with poor H-atom donor properties such as benzene derivatives with 1,2-difluorobenzene being superior to other halogenated benzene derivatives. We further screened a range of common photocatalysts to identify the optimal reaction conditions.

Table S7. Photocatalyst screen in 1,2-difluorobenzene

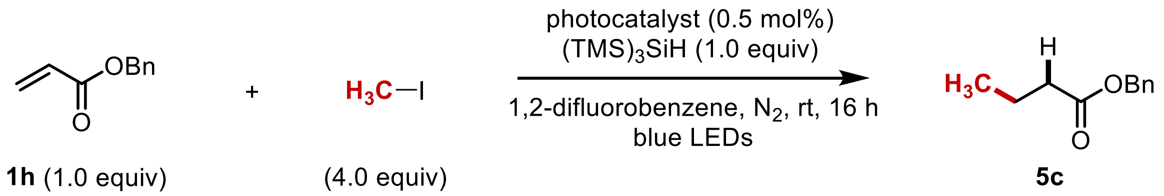

entry	photocatalyst	yield (5c)
**1**	*none*	11%
**2**	Eosin Y	48%
**3**	*fac*-Ir(ppy)$_3$	60%
**4**	MesAcr·BF$_4$	59%
**5**	Ir(dF[CF$_3$]ppy)$_2$(dtbbpy)·PF$_6$	59%

As shown, a range of photocatalysts are suitable for this transformation. We selected the organophotocatalyst MesAcr·BF$_4$ as a green and inexpensive alternative to iridium-based catalysts.

Table S8. Screen of (TMS)$_3$SiH equivalents

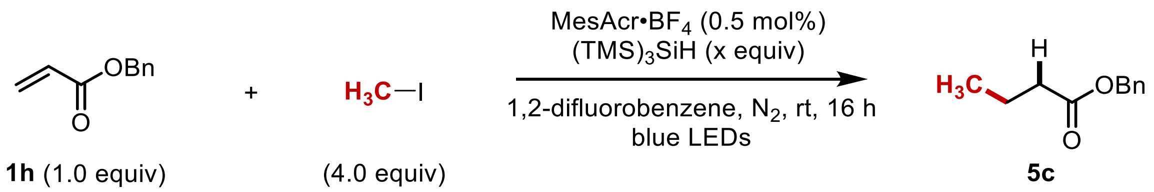

entry	(TMS)$_3$SiH (x equiv)	yield (5c)
**1**	1.0 equiv	48%
**2**	1.2 equiv	55%
**3**	1.5 equiv	59%
**4**	2.0 equiv	60%
**5**	3.0 equiv	79% (67%*)
**6**	4.0 equiv	64%

*Yield of isolated product is depicted in brackets.

The product yield was further increased by using excess of $(TMS)_3SiH$. The desired product was isolated in 67% yield when using 3.0 equivalents of $(TMS)_3SiH$. The discrepancy in quantitative ^1H NMR yield and isolated yield likely stems from overestimation of the NMR yield due to overlapping peaks.

5. Mechanistic investigation

5.1 Radical-trapping experiment with TEMPO

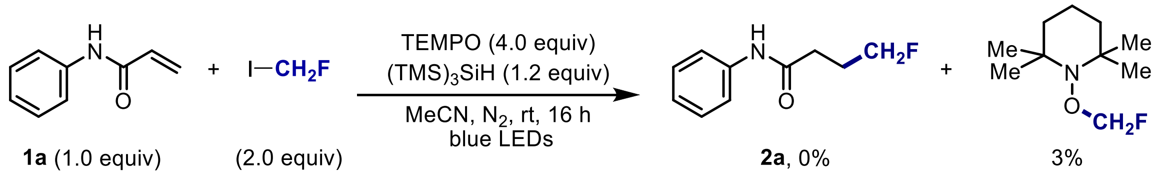

To a 7 mL vial equipped with a stir bar was added *N*-phenylacrylamide (74.0 mg, 0.5 mmol, 0.5 equiv), MeCN (3.0 mL), $(TMS)_3SiH$ (185 µL, 0.6 mmol, 0.6 equiv), CH_2FI (68 µL, 1.0 mmol, 2.0 equiv), and TEMPO (313 mg, 2.0 mmol, 4.0 equiv). The vial was sealed with a septum, degassed by nitrogen bubbling for 10 seconds and wrapped with parafilm. The reaction mixture was stirred in a EvoluChemTM PhotoRedOx Box with a 18W blue LED lamp (λ = 450 nm) at room temperature (using a fan) for 16 hours. *α*,*α*,*α*-Trifluorotoluene (internal standard, 123 µL, 1.0 mmol, 1.0 equiv) was added, and the reaction mixture was analyzed by quantitative ^{19}F NMR (**^{19}F NMR** (377 MHz, $CDCl_3$) δ -137.59 (t, J = 57.0 Hz)).

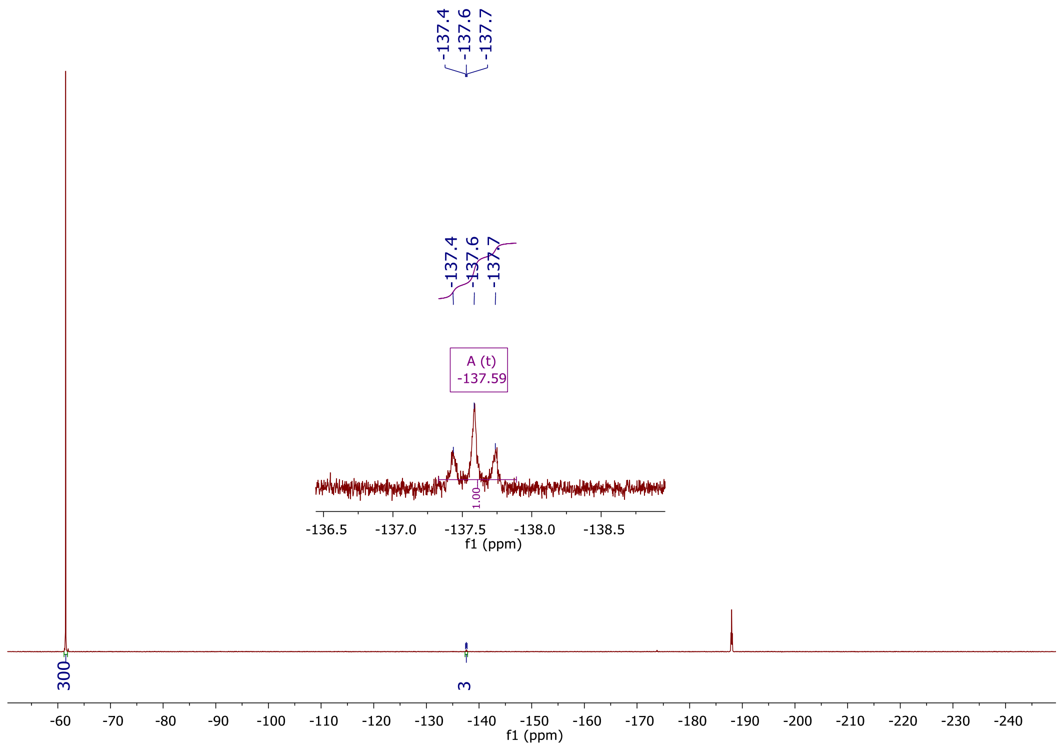

Figure S4: Quantitative ^{19}F NMR of the TEMPO experiment.

5.2 Deuteration experiment

To probe the hydrogen source in this transformation, a deuteration experiment in MeCN-d_3 was performed.

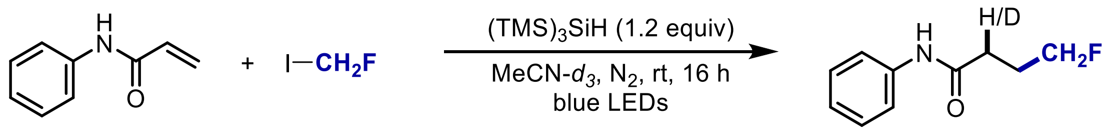

1a (1.0 equiv) (2.0 equiv) **2a[H]**, 70% (^{19}F NMR) **2a[D]**, <1% (^{19}F NMR)

To a 7 mL vial equipped with a stir bar was added *N*-phenylacrylamide (74.0 mg, 0.5 mmol, 0.5 equiv), MeCN-d_3 (3.0 mL), $(TMS)_3SiH$ (185 µL, 0.6 mmol, 1.2 equiv), CH_2FI (68 µL, 1.0 mmol, 2.0 equiv). The vial was sealed with a septum, degassed by nitrogen bubbling for 10 seconds and wrapped with parafilm. The reaction mixture was stirred in a EvoluChemTM PhotoRedOx Box with a 18W blue LED lamp (λ = 450 nm) at room temperature (using a fan) for 16 hours. α,α,α-Trifluorotoluene (internal standard, 123 µL, 1.0 mmol, 1.0 equiv) was added, and the reaction mixture was analyzed by quantitative ^{19}F NMR and ^{1}H NMR.

Addition of deuterated solvents did not result in deuterium incorporation; selective formation of [H]**2a** was observed over [D]**2a**. This result suggests that the solvent is not the hydrogen source in the hydrofluoromethylation of electron-deficient alkenes.

5.3 Control experiments with AIBN/Bu_3SnH

To a 7 mL vial equipped with a stir bar was added *N*-phenylacrylamide (74.0 mg, 0.5 mmol, 0.5 equiv), AIBN (41.1 mg, 0.25 mmol, 0.5 equiv), MeCN (3.0 mL), Bu_3SnH (161 µL, 0.6 mmol, 1.2 equiv) or $(TMS)_3SiH$ (185 µL, 0.6 mmol, 1.2 equiv), and CH_2FI (68 µL, 1.0 mmol, 2.0 equiv). The vial was sealed with a septum, degassed by nitrogen bubbling for 10 seconds and wrapped with parafilm. The reaction mixture was stirred at 80° C for 16h. *α,α,α*-Trifluorotoluene (internal standard, 123 µL, 1.0 mmol, 1.0 equiv) was added, and the reaction mixture was analyzed by quantitative ^{19}F NMR.

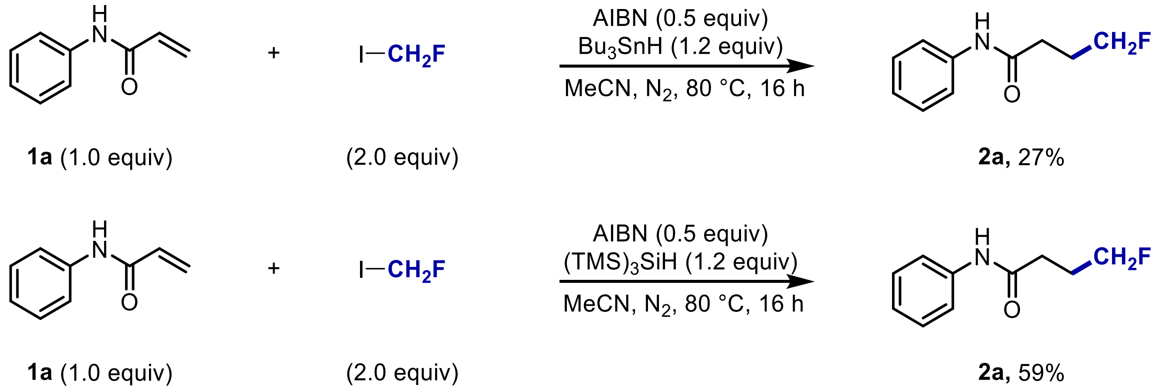

6. Proposed mechanisms

We propose the following reaction mechanism for the hydrofluoromethylation of electron-deficient alkenes: upon blue-light irradiation, the reaction is initiated by homolytic bond cleavage of the carbon-iodine bond of fluoroiodomethane to generate trace amounts of the fluoromethyl radical **A**, along with an iodine radical. The latter radical can undergo hydrogen atom abstraction with $(TMS)_3SiH$ to afford hydrogen iodide and the corresponding nucleophilic tris(trimethylsilyl)silyl radical **B**. Subsequent iodine atom abstraction from fluoroiodomethane would then yield radical **A**. This fluoroalkyl radical undergoes Giese-addition to the electron-deficient alkene and furnishes an electrophilic carbon-centred radical intermediate **C**. HAT between the carbon-centred radical and $(TMS)_3SiH$ affords the desired hydrofluoromethylated product, along with a new silyl radical **B**, which enters chain propagation.

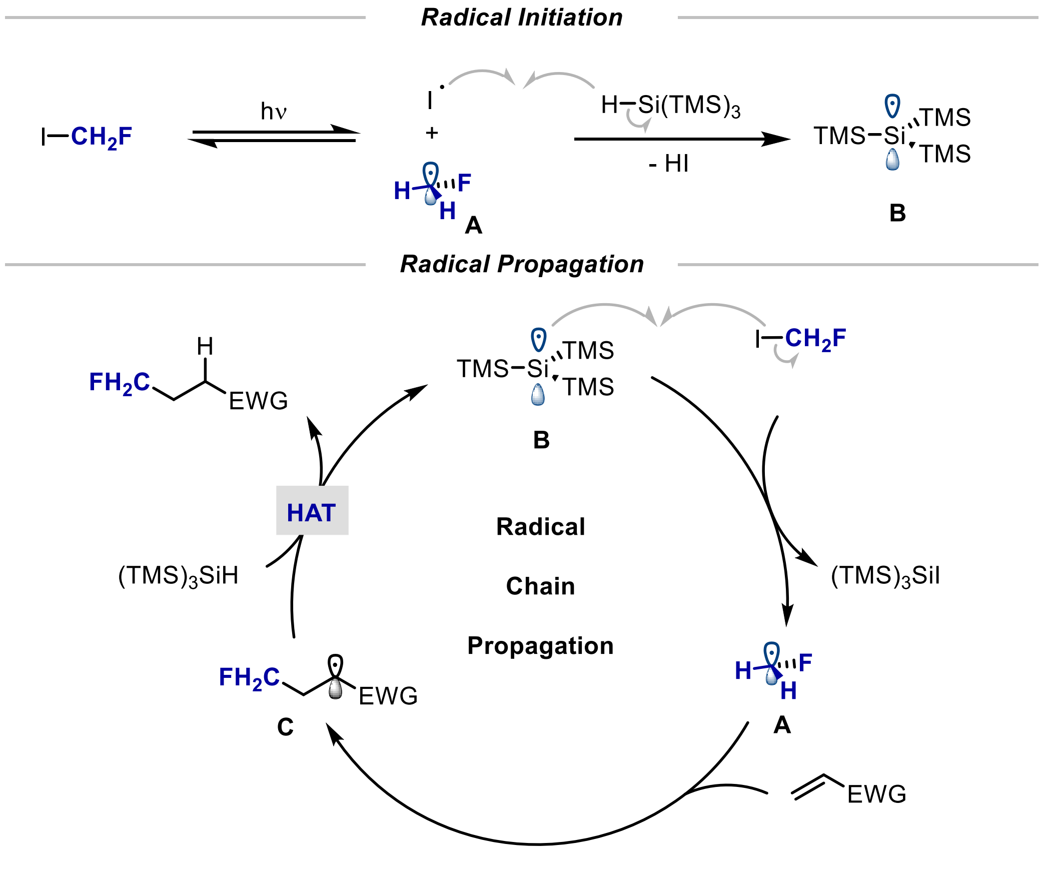

Scheme S5: Proposed radical chain propagation mechanism for the hydrofluoromethylation of electron-deficient alkenes.

7. Kinetic profile

Experiments were conducted to evaluate the influence of the photocatalyst on the kinetics of the reaction; this was accomplished by monitoring the conversion of **1a** to hydrofluoromethylated product **2a** under our standard reaction conditions in the presence and absence of the photocatalyst *fac*-Ir(ppy)$_3$ over the course of four hours.

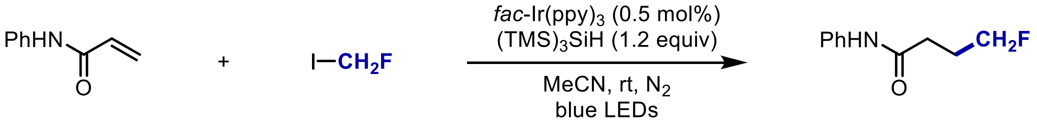

1a (1.0 equiv) (2.0 equiv) **2a**

Whilst the final yield of the desired product was not significantly affected by the presence or absence of photocatalyst (see table S1), the hydrofluoromethylation of **1a** was found to take place significantly faster in the presence of *fac*-Ir(ppy)$_3$, with most conversion of the starting material to the desired product occurring within the first ten minutes of irradiation (Table S9). In contrast, the reaction in absence of photocatalyst is much slower and requires longer reaction time. These observations suggest that the photocatalyst acts as initiator.

Procedure: To a 7 mL vial equipped with a magnetic stir bar, was added *N*-phenylacrylamide (73.6 mg, 0.50 mmol, 1.0 equiv), *fac*-Ir(ppy)$_3$ (1.6 mg, 0.0025 mmol, 0.5 mol%), MeCN (dry; 3.0 mL), (TMS)$_3$SiH (185 μL, 0.6 mmol, 1.2 equiv), CH_2FI (68 μL, 1.0 mmol, 2.0 equiv) and *α,α,α*-Trifluorotoluene as an internal standard (123 μL, 1.0 mmol). The vial was sealed with a septum, degassed by nitrogen bubbling for 10 seconds and wrapped with parafilm. The reaction mixture was stirred in a EvoluChemTM PhotoRedOx Box with a 18W blue LED lamp (λ = 450 nm) at room temperature (using a fan). At intervals of ten minutes to four hours, aliquots (50 μL) of the reaction mixture were removed and analysed by quantitative ^{19}F NMR.

Table S9: Yield of **2a** measured at regular time intervals with and without photocatalyst (average of two runs).

reaction time	yield (2a) without photocatalyst	yield (2a) with photocatalyst
**10 min**	14%	66%
**20 min**	29%	66%
**30 min**	40%	67%
**40 min**	45%	67%
**50 min**	51%	67%
**1 h**	54%	68%
**2 h**	59%	70%
**3 h**	60%	70%
**4 h**	64%	70%

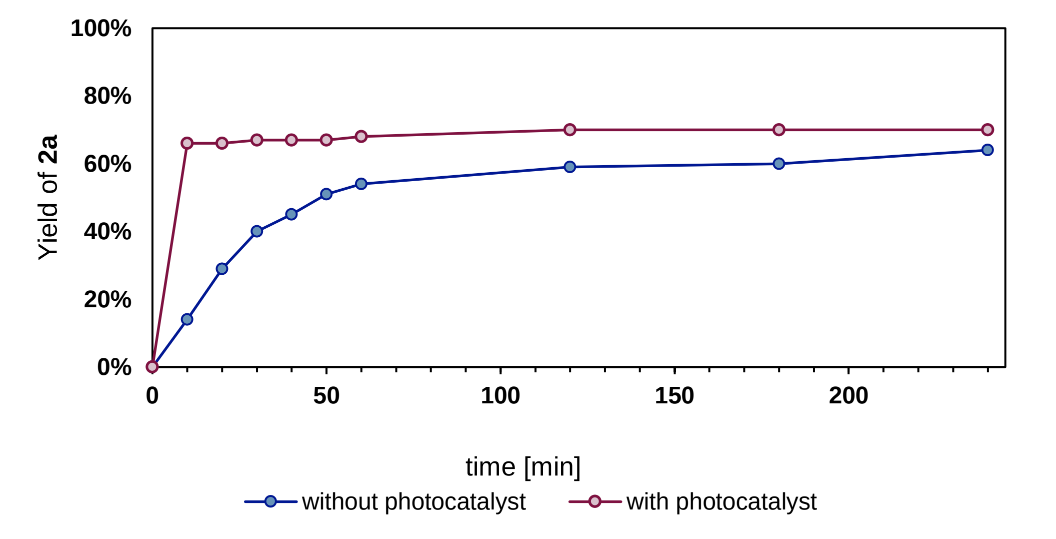

8. Scale-up experiment

The scale-up experiment was performed on the synthesis of 1-benzyl-3-(fluoromethyl)pyrrolidine-2,5-dione.

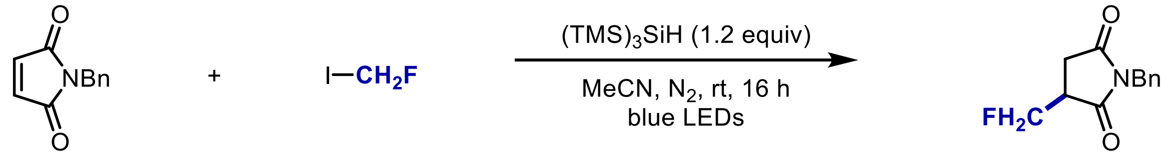

1o, (1.0 equiv) (2.0 equiv) **2o**, 88%

Scheme S6: Multigram synthesis of **2n**.

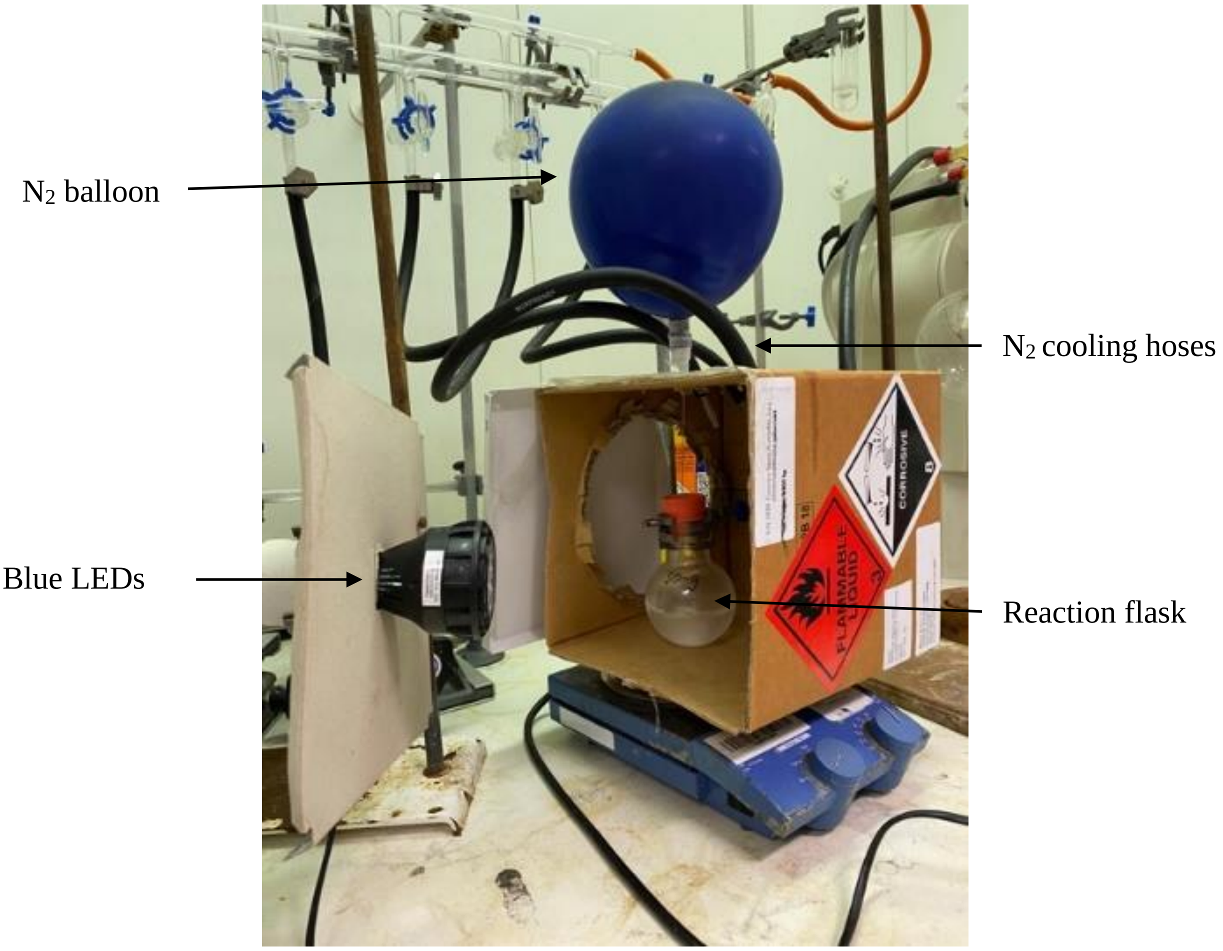

Figure S7: Scale-up experimental set-up.

To a 150 mL oven-dried round-bottomed flask equipped with a magnetic stir bar, *N*-benzylmaleimide (1.872 g, 10 mmol, 1.0 equiv) was added, followed by dry MeCN (60 mL), $(TMS)_3SiH$ (3.70 mL, 12 mmol, 1.2 equiv) and CH_2FI (1.35 mL, 20 mmol, 2.0 equiv). The flask was equipped with a Teflon septum, degassed by nitrogen bubbling for 1 minute and stirred in a custom-made photoreactor (see figures S8 and S9) with a 12 W blue HepatoChem LED lamp (λ_{max} = 470 nm) at room temperature for 16 hours. Et_2O (100 mL) was then added to the reaction mixture, which was extracted twice with a saturated aqueous solution of $NaHCO_3$ (2 x 50 mL). The combined aqueous phases were extracted once more with Et_2O (30 mL). The combined organic phases were dried (Na_2SO_4), filtered and the solvent was removed *in vacuo*. The residue was purified by flash chromatography (SiO_2, Et_2O in Pentane, 30/70 to 60/40). The desired fractions were collected and concentrated *in vacuo* to afford the desired hydrofluoromethylated product **2o** as a pale yellow oil (1.953 g, 8.8 mmol, 88.0 %).

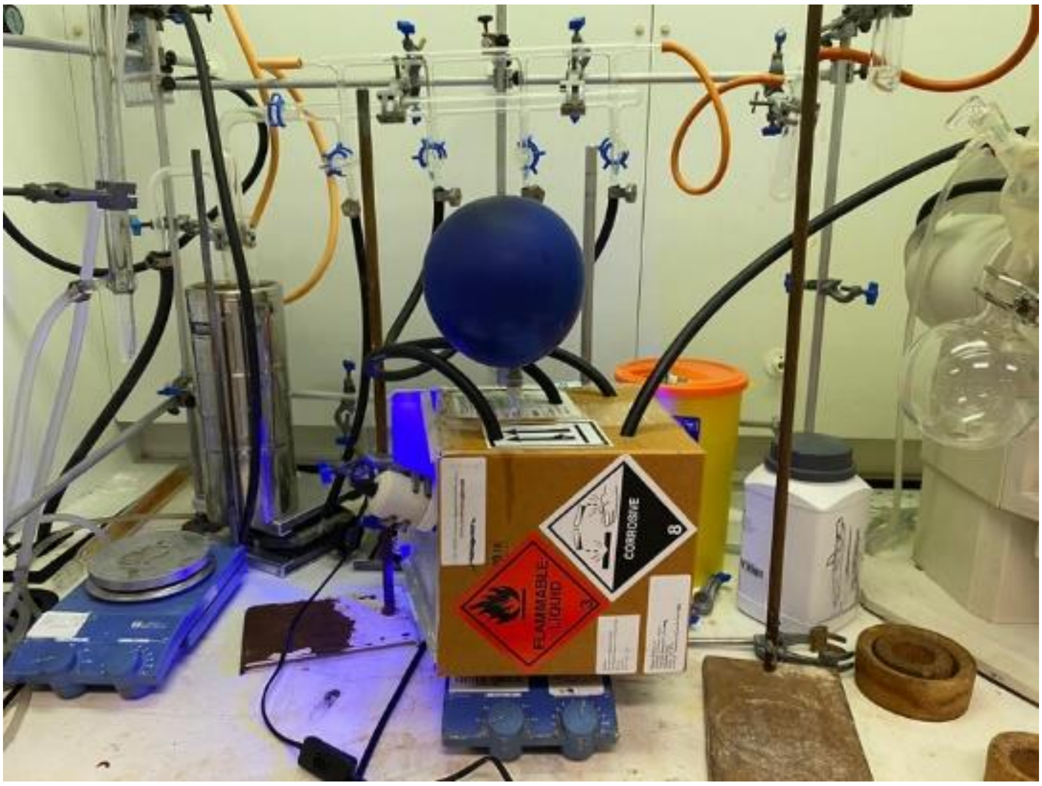

Figure S8: Scale-up experimental set-up.

9. Robustness screen

The robustness screening was performed by spiking one equivalent of an additive (heterocycle, molecule containing a specific functional group, or amino acid) under our standard reaction conditions. In contrast to our standard reaction conditions, screenings were performed in DMF to allow for homogenous reaction mixtures. All reactions were performed on 0.1 mmol scale of model substrate **1o** and the crude reaction mixtures were analysed by quantitative ^{19}F NMR using *α*,*α*,*α*-trifluorotoluene as the internal standard.

These experimental robustness data provide an overview of the heterocycles, functional groups or amino acids that are likely to be tolerated under our reaction conditions. This screening demonstrates that our methodology is robust enough to be considered as a valuable transformation for the chemoselective late-stage hydrofluoromethylation of complex molecules.

Important note: Potential side reactivity of the additive during the reaction was not assessed in this screening. In presence of nucleophilic functional groups (e.g. phenols, thiols), side products resulting from nucleophilic substitution with fluoroiodomethane was observed by ^{19}F NMR. As a general rule, lowering the amount of fluoroiodomethane to 1.0 equivalent in 1,2-difluorobenzene, allowed to reduce the formation of these side products, however leading also to lower yields of the desired product.

The hydrofluoromethylation of Ibrutinib and *N*-acryloyl *L*-tyrosine methyl ester, which gave the desired products in 58% and 66% respectively, correlate with our robustness screen, as complex fused heterocycles (e.g. 1*H*-pyrazolo[3,4-d]pyrimidin-4-amine), phenols and protected amino acids were shown to be tolerated under our reaction conditions.

Procedure: To an 7 mL vial equipped with a stir bar was added additive (0.1 mmol, 1.0 equiv), *N*-benzylmaleimide (18.7 mg, 0.1 mmol, 1.0 equiv.), DMF (dry; 0.6 mL), $(TMS)_3SiH$ (37 µL, 0.12 mmol, 1.2 equiv), fluoroiodomethane (CH_2FI) (13.6 µL, 0.2 mmol, 2.0 equiv.). The vial was sealed with a septum, degassed by nitrogen bubbling for 10 seconds and wrapped with parafilm. The reaction mixture was stirred in a EvoluChemTM PhotoRedOx Box with a 18W blue LED lamp (λ = 450 nm) at room temperature (using a fan) for 16 hours. α,α,α-Trifluorotoluene (internal standard, 123 µL, 1.0 mmol) was added, and the reaction mixture was analysed by quantitative ^{19}F NMR.

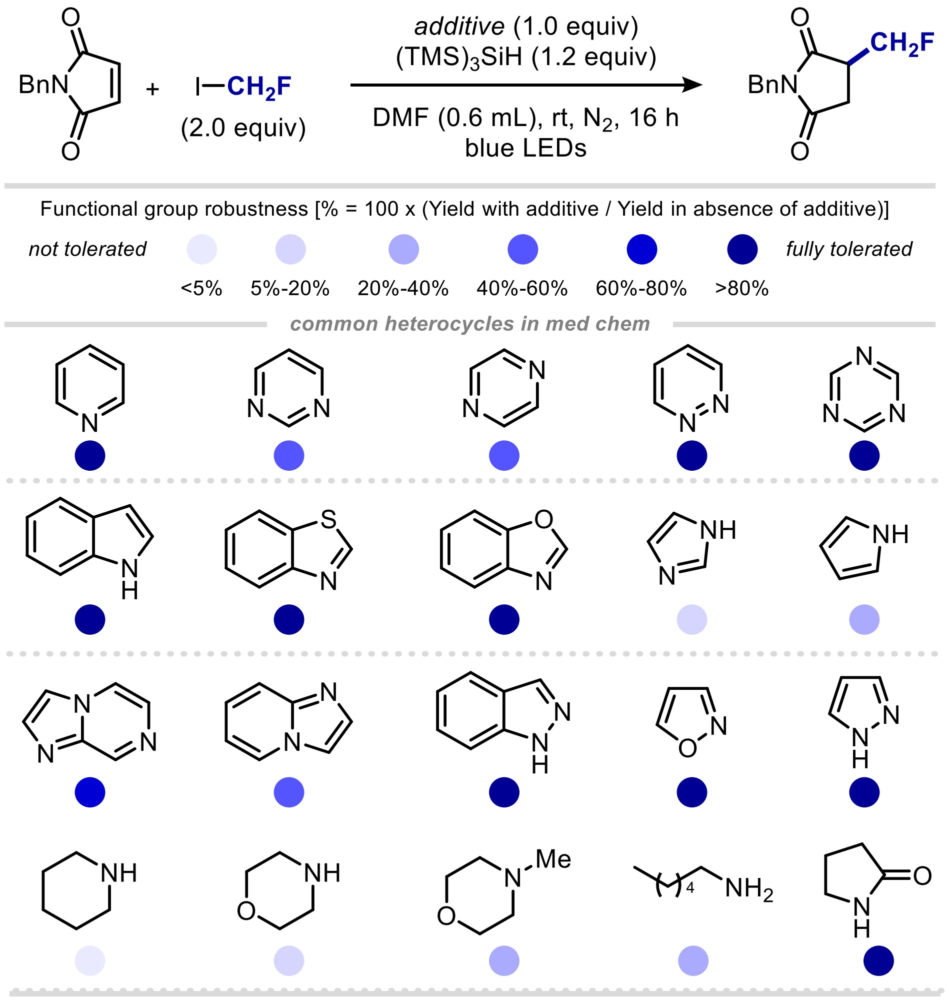

Scheme S9: Robustness screen of common heterocycles in medicinal chemistry.

Common heterocycles in medicinal chemistry, such as pyridine, pyridazine, 1,3,5-triazine, indole or benzothiazole and oxazole were broadly tolerated and did not significantly affect the outcome of the reaction. However, other heteroarenes, such as imidazole and pyrrole prevented partly the hydrofluoromethylation of our model substrate. Other fused and five-membered heteroarenes were well tolerated. However, aliphatic amines, such as piperidine, morpholine, *N*-

methyl morpholine and hexylamine resulted in lower yields. Although cyclic aliphatic amines are not well tolerated, the broad tolerance to the other heterocycles is substantial, particularly considering the ubiquity of these motifs in drug discovery.

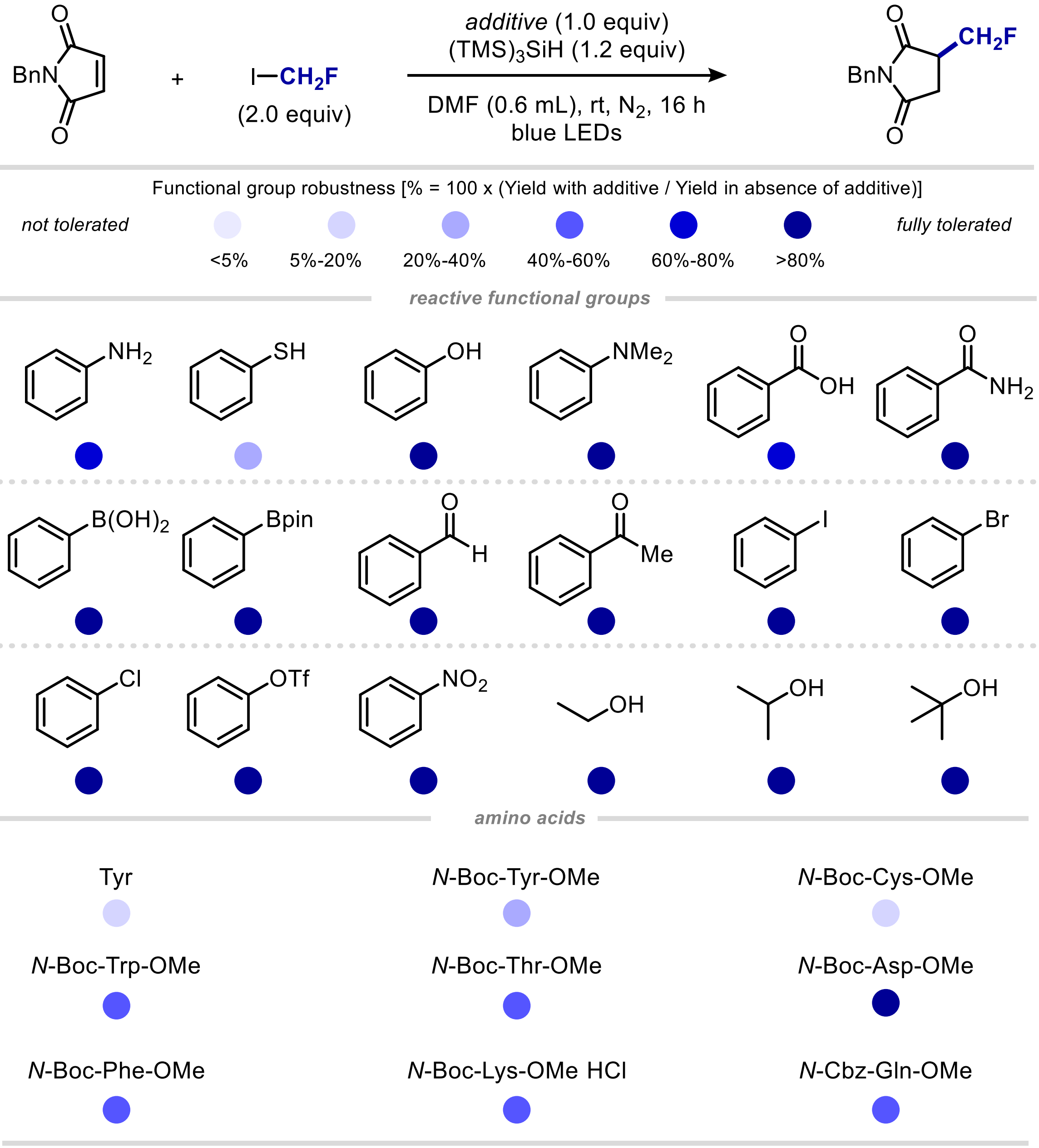

Scheme S10: Robustness screen of reactive functional groups and amino acids.

Reactive functional groups commonly found in complex molecules, including primary and secondary amines, alcohol, carboxylic acid, amide, boronic acid, ester, aldehyde, ketone, halogens, as well as triflate and nitro groups were well tolerated, with little to no effect on the reaction yield. Thiols, however, significantly reduced the reaction yield, presumably due to their nucleophilic character. Furthermore, thiols are excellent H-atom donors, and thus might undergo H-atom transfer with the CH_2F radical. Importantly, although alcohol functionalities, including phenols and aliphatic alcohols, did not alter the yield of the desired hydrofluoromethylation reaction, side products arising from nucleophilic attack were observed by ^{19}F NMR (~ 35%). However, it was found that competitive alkylation could be significantly reduced by using 1.0 equivalent of CH_2FI in 1,2-difluorobenzene as solvent, albeit at the expense of reduced yield for the hydrofluoromethylated product.

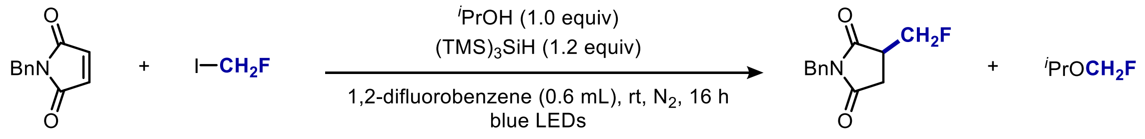

1o (1.0 equiv) (1.0 equiv) 52% 4%

Finally, we were also interested in investigating the tolerance of amino acids under our reaction conditions. The data collected demonstrated that unprotected amino acids, such as *L*-tyrosine for example, are not well tolerated by the reaction conditions. In contrast, reactions in the presence of fully protected (N- and O-protected) amino acids proceeded in higher yields. Most amino acids, except cysteine derivatives, gave the desired product in moderate to excellent yield.

10. Further functionalizations

1-Benzyl-3-(fluoromethyl)pyrrolidine (2p)

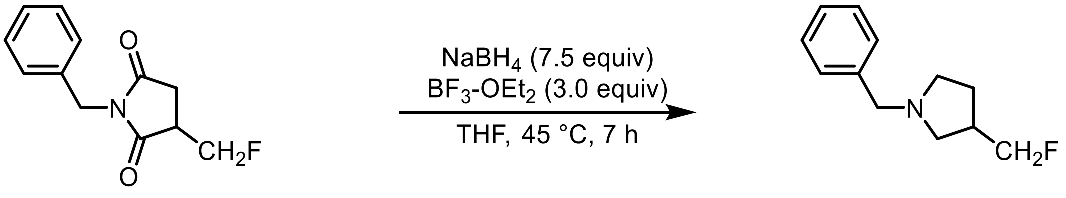

2o (1.0 equiv) **2p**, 64%

To a well-stirred solution of 1-benzyl-3-(fluoromethyl)pyrrolidine-2,5-dione (111 mg, 0.5 mmol, 1.0 equiv) in dry THF (1.5 mL), sodium borohydride (142 mg, 3.75 mmol, 7.5 equiv) was added at -20 °C (ice + NaCl). $BF_3 \cdot Et_2O$ (185 uL, 213 mg, 1.5 mmol, 3.0 equiv) was added slowly at the same temperature. The reaction mixture was warmed to room temperature and stirred for 7 h at 45 °C. Unreacted $NaBH_4$ was quenched dropwise with methanol, diluted with water, and extracted with DCM. The combined DCM layers were washed with water and dried over anhydrous sodium sulfate. After evaporation, the desired compound was obtained by flash chromatography (silica, Et_2O in pentane 10/90 to 20/80) to yield the desired product (61 mg, 0.32 mmol, 64%) as a white solid.

^{1}H NMR (400 MHz, $CDCl_3$) δ 7.43 – 7.35 (m, 5H), 4.42 – 4.16 (m, 2H), 4.08 (s, 2H), 3.25 – 3.06 (m, 2H), 3.06 – 2.89 (m, 1H), 2.88 – 2.78 (m, 1H), 2.75 – 2.66 (m, 1H), 2.48 – 2.35 (m, 1H), 1.65 – 1.54 (m, 1H). **^{13}C NMR** (101 MHz, $CDCl_3$) δ 132.7, 131.4, 129.1, 128.4, 84.2 (d, $J_{C\text{-}F}$ = 170.1 Hz), 66.4, 60.2 (d, $J_{C\text{-}F}$ = 5.1 Hz), 58.2, 37.1 (d, $J_{C\text{-}F}$ = 19.1 Hz), 25.1 (d, $J_{C\text{-}F}$ = 5.9 Hz). **^{19}F NMR** (377 MHz, $CDCl_3$) δ -224.9 (td, J = 47.3, 25.7 Hz). **HRMS** (ESI-TOF) calculated for $C_{12}H_{17}NF$ $[M+H]^+$: 194.1340, found 194.1340; **IR** (neat) 3734, 3066, 2957, 2918, 2850, 2363, 2337, 2282, 1735, 1653, 1541, 1499, 1455, 1388, 1362, 1351, 1312, 1244, 1212, 1177, 1167, 1121, 1107, 1084, 1068, 1031, 1017, 996, 934, 920, 890, 865, 801, 757, 700, 669, 648, 628 ; **m.p.**: 75 – 77 °C.

N-(4-fluorobutyl)aniline (2q)

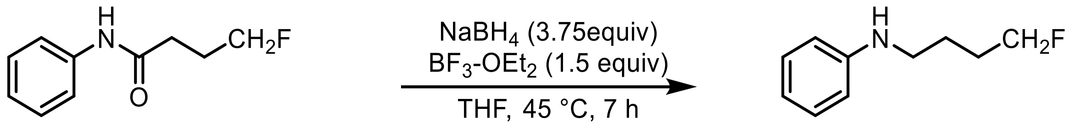

2a (1.0 equiv) **2q**, 82%

To a well-stirred solution of 4-fluoro-*N*-phenylbutanamide (45 mg, 0.25 mmol, 1.0 equiv) in dry THF (0.75 mL), sodium borohydride (36 mg, 0.94 mmol, 3.5 equiv) was added at -20 °C (ice + NaCl). $BF_3 \cdot Et_2O$ (46 uL, 53 mg, 0.38 mmol, 1.5 equiv) was added slowly at the same temperature. The reaction mixture was warmed to room temperature and stirred for 7 h at 45 °C. Unreacted $NaBH_4$ was quenched dropwise with methanol, diluted with water, and extracted with DCM. The combined DCM layers were washed with water and dried over anhydrous sodium sulfate. After evaporation, the desired compound was obtained by flash chromatography (silica, Et_2O in pentane 0/100 to 20/80) to yield the desired product **2q** (34 mg, 0.2 mmol, 82%) as a pale-yellow oil.

^{1}H NMR (400 MHz, $CDCl_3$) δ 7.25 – 7.16 (m, 2H), 6.77 – 6.70 (m, 1H), 6.68 – 6.59 (m, 2H), 4.51 (dt, J = 47.1, 5.8 Hz, 2H), 3.65 (s, 1H), 3.20 (t, J = 6.9 Hz, 2H), 1.92 – 1.71 (m, 4H); **^{13}C NMR** (101 MHz, $CDCl_3$) δ 148.3, 129.4, 117.5, 112.9, 83.9 (d, $J_{C\text{-}F}$ = 164.9 Hz), 43.7, 28.2 (d, $J_{C\text{-}F}$ = 19.9 Hz), 25.6 (d, $J_{C\text{-}F}$ = 4.5 Hz); **^{19}F NMR** (377 MHz, $CDCl_3$) δ -218.2 (tt, J = 47.7, 25.5 Hz). The compound did not ionize; **IR** (neat) 3414, 3052, 2961, 2868, 2161, 2021, 1602, 1506, 1477, 1432, 1389, 1320, 1257, 1180, 1154, 1134, 1042, 991, 947, 894, 870, 838, 747, 692, 618.

4-fluoro-2-methylbutan-1-ol (2r)

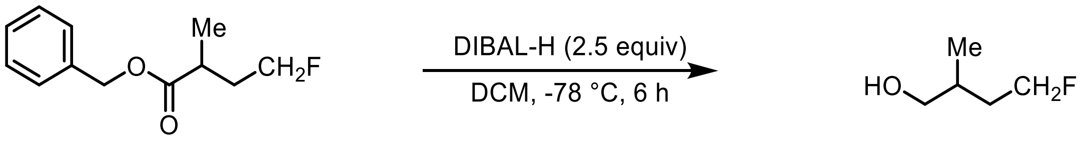

2j (1.0 equiv) **2r**, 30%

DIBAL-H (1.25 mL, 1.25 mmol, 1.0 M in DCM, 2.5 equiv), was added dropwise to a stirred solution of benzyl 4-fluoro-2-methylbutanoate (105 mg, 0.5 mmol, 1.0 equiv) in DCM (5.0 mL) at -78 °C under N_2 atmosphere. The reaction mixture was stirred at room temperature for 16 hours. The desired product (30%) was observed by quantitative ^{1}H NMR analysis of the crude reaction mixture using triphenylmethane as an internal standard.

4-fluoro-2-methylbutanoic acid (2s)

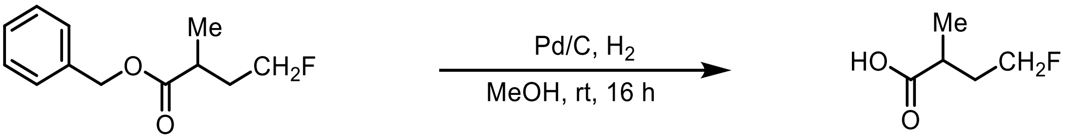

2j (1.0 equiv) **2s**, 94%

10% Pd/C (11 mg, 0.1 mmol, 5.0 equiv) was added to a solution of benzyl 4-fluoro-2-methylbutanoate (105 mg, 0.5 mmol, 1.0 equiv) in MeOH (3.0 mL). The air was replaced with H_2 (balloon) by three vacuum/H_2 cycles. The reaction mixture was stirred at room temperature for 16 h and filtered over Celite to afford the desired compound as white oil (56 mg, 0.47 mmol, 94%).

^{1}H NMR (400 MHz, MeOD) δ 4.58 – 4.33 (m, 2H), 2.50 – 2.41 (m, 1H), 2.10 – 1.91 (m, 1H), 1.79 – 1.62 (m, 1H), 1.15 (d, J = 7.0 Hz, 3H); **^{19}F NMR** (377 MHz, MeOD) δ -221.3 (tt, J = 47.9, 25.1 Hz); **HRMS** (ESI-TOF) calculated for $C_5H_8O_2F$ [M-H]$^-$: 119.0514, found 119.0503; **IR** (neat) 2967, 2918, 2850, 2528, 2361, 2342,2160, 1973, 1770, 1701, 1570, 1461, 1412, 1376, 1292, 1241, 1206, 1138, 1082, 1030, 989, 953, 885, 849, 784, 720, 668, 649. The product decomposed within a few hours after isolation (^{13}C NMR was therefore not measured for this compound).

11. ^{18}F-Hydrofluoromethylation of alkenes

Synthesis and purification of [^{18}F]CH_2FI

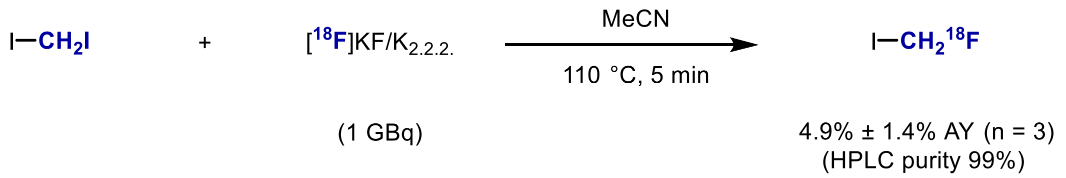

^{18}F-Fluoride was separated from ^{18}O-enriched-water using an anion exchange cartridge (Waters Sep-Pak AccellPlus QMA Carbonate Plus Light Cartridge, activated with H_2O (10.0 mL) prior to use) and released with a solution of Kryptofix (17 mg), and K_2CO_3 (3.5 mg) in MeCN/H_2O (0.75 mL, 4:1, v/v), which was concentrated by azeotropic drying. To dry [^{18}F]fluoride was added a solution of CH_2I_2 (50 µL in 1.0 mL) in dry MeCN. The vial was equipped with a PEEK line leading into a P_2O_5/NaOH* cartridge. The outlet of this cartridge was leading to a v-vial containing DMF (1.0 mL) which was cooled to -20 °C. The reaction vial was heated to 110 °C for 5 minutes and the distilled product was analysed by radioHPLC. [^{18}F]CH_2FI was isolated in 4.9% ± 1.4% AY (n = 3).

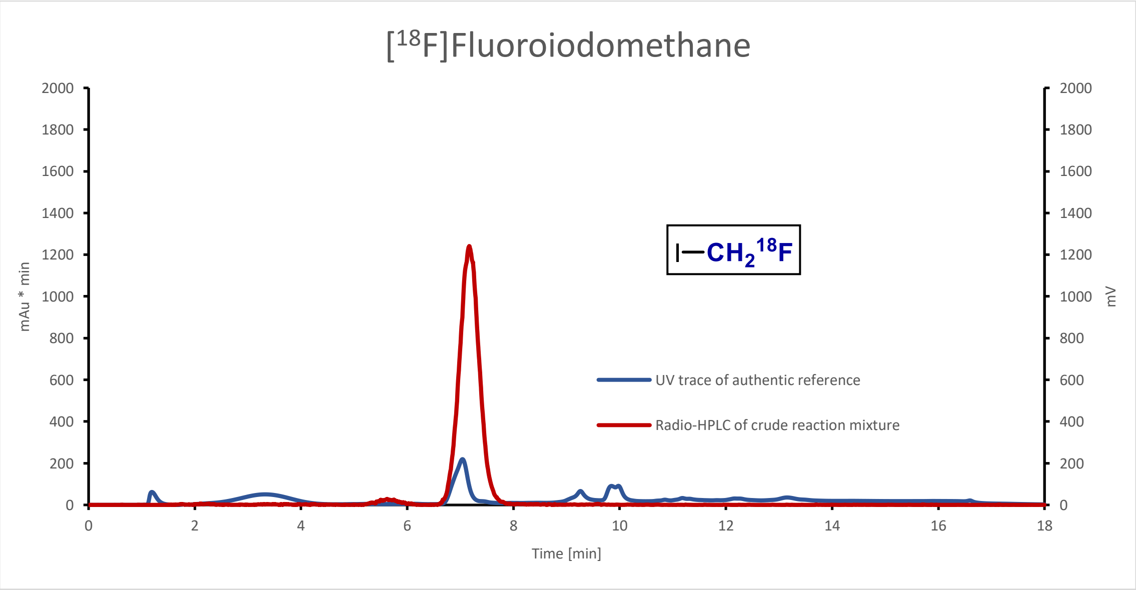

***Note**: The P_2O_5/NaOH cartridge was prepared according to the following procedure:*

The plunger of a 3.0 mL syringe was removed, and a piece of cotton wool was placed in the neck of the syringe. The syringe was cut at the 1.5 mL mark and P_2O_5 (250 mg) and finely grinded NaOH (250 mg) were added as solids (careful; reacts violently in presence of water). The cartridge was then sealed with a rubber septum and used within 18 hours. Unused cartridges were quenched in an ice bath.

General procedure for the ^{18}F-Hydrofluoromethylation of electron-deficient alkenes

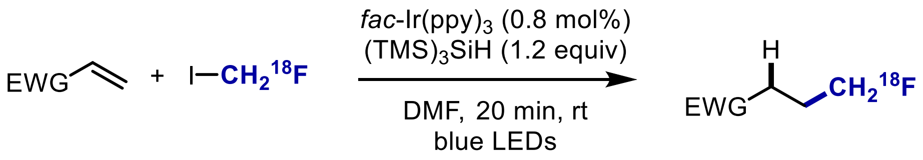

To a 1.75 mL vial containing alkene (0.1 mmol), $(TMS)_3SiH$ (37 µL, 0.12 mmol, 1.2 equiv), *fac*-Ir(ppy)$_3$ (0.5 mg) and DMF (300 µL) was added freshly distilled [^{18}F]CH_2FI (8 – 10 MBq) in DMF (300 µL). The mixture was placed in a photoreactor and left under blue LED irradiation for 20 min without stirring. An aliquot of this mixture was filtered and analysed by radioHPLC.

***Note:** The reaction performed better in absence of stirring leading to a cleaner reaction profile.*

Overlay of radiotraces

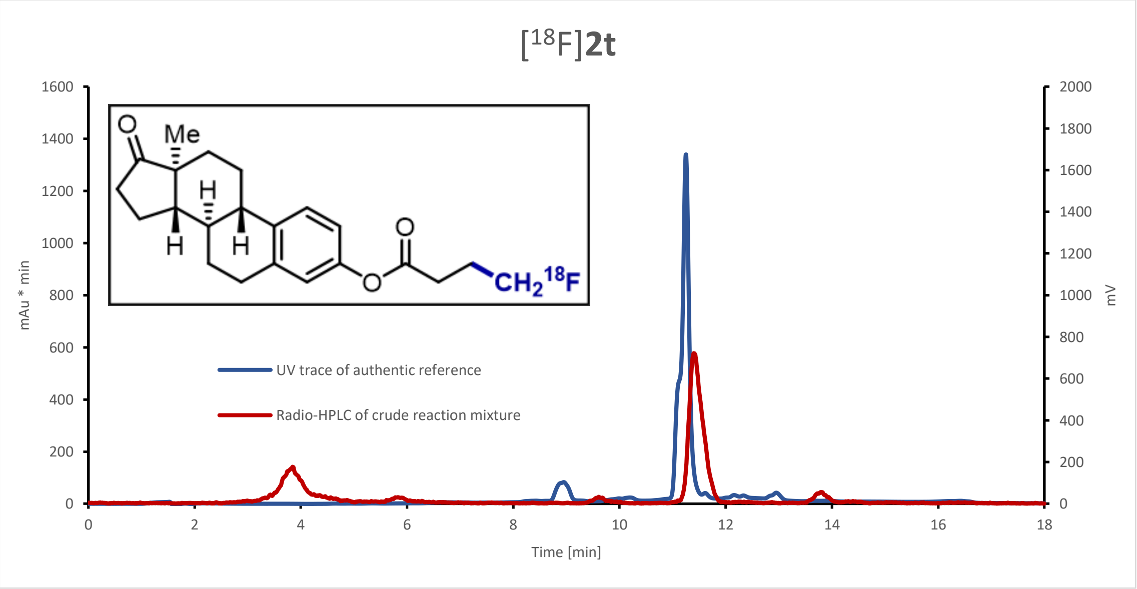

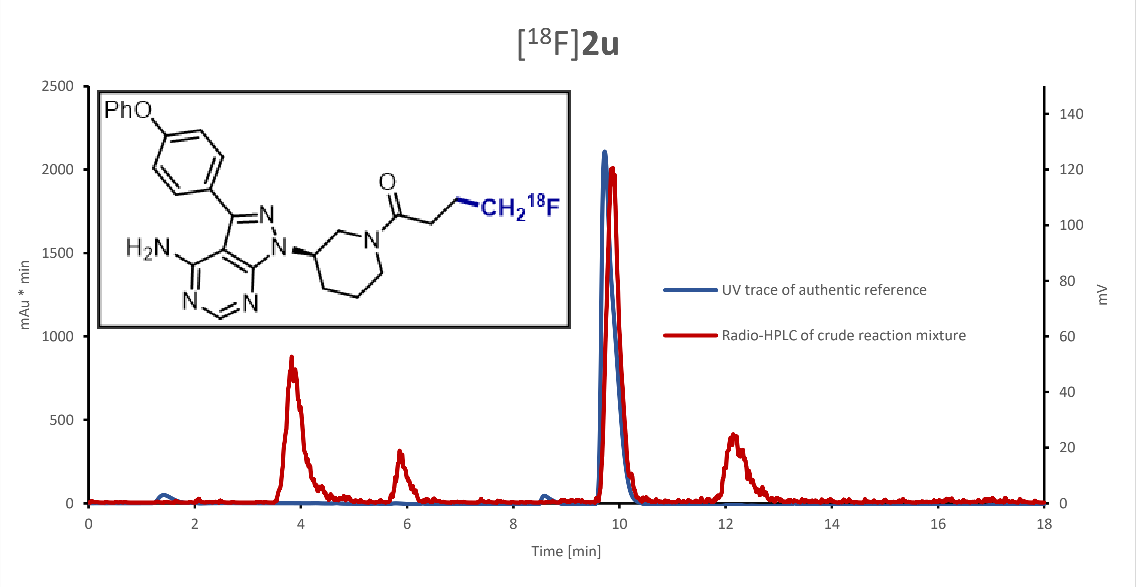

[18F]2u
PhO
H2N
CH2 18F
UV trace of authentic reference
Radio-HPLC of crude reaction mixture
mAu * min
mV
Time [min]

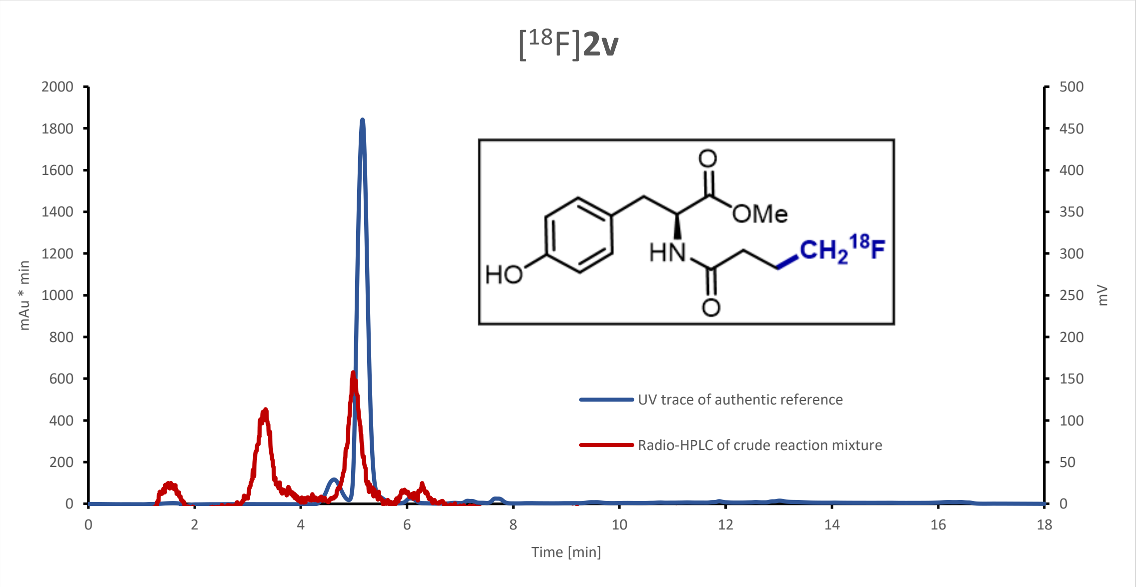

[18F]2v
HO
HN
OMe
CH2 18F
UV trace of authentic reference
Radio-HPLC of crude reaction mixture
mAu * min
mV
Time [min]

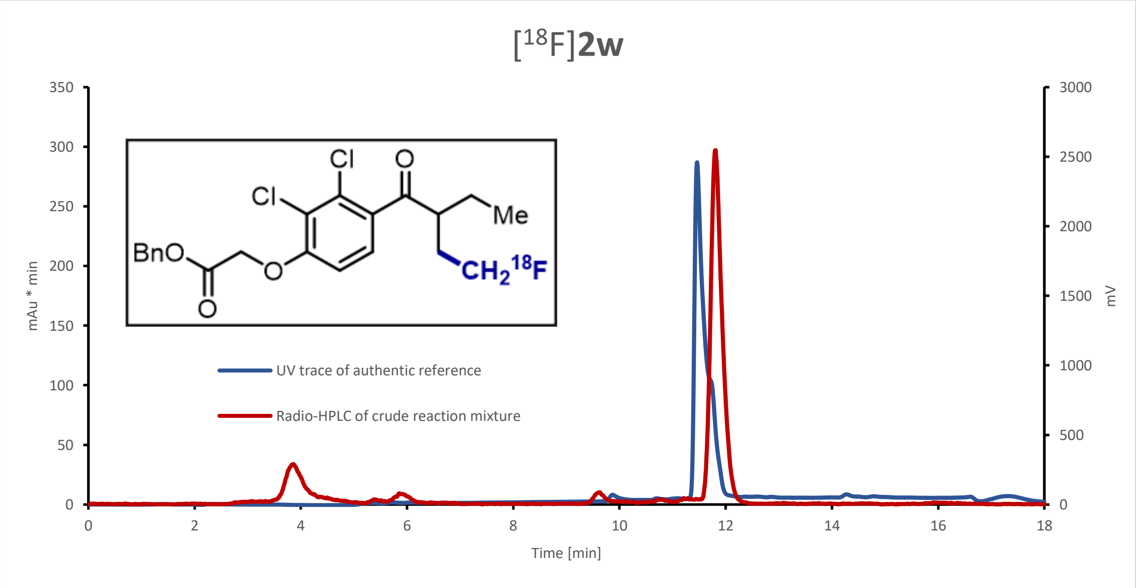

[18F]2w
Cl
Cl
BnO
Me
CH2 18F
UV trace of authentic reference
Radio-HPLC of crude reaction mixture
mAu * min
mV
Time [min]

12. Competition experiments

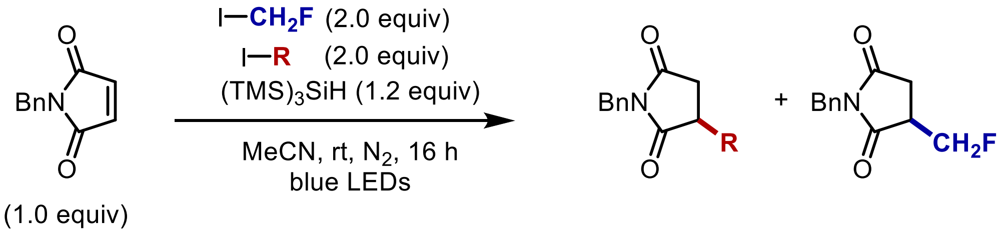

Me—I	(BDE ≈ 239 kJ mol^{-1})	**vs**	I—CH_2F	**5a**, 25%	**2o**, 74%
Et—I	(BDE ≈ 234 kJ mol^{-1})	**vs**	I—CH_2F	**6**, 43%	**2o**, 51%
*i*Pr—I	(BDE ≈ 235 kJ mol^{-1})	**vs**	I—CH_2F	**7**, 62%	**2o**, 36%
*t*Bu—I	(BDE ≈ 227 kJ mol^{-1})	**vs**	I—CH_2F	**8**, 73%	**2o**, 19%

General Procedure 1: To a 7 mL vial equipped with a magnetic stir bar, was added *N*-benzylmaleimide (93.5 mg, 0.50 mmol, 1.0 equiv), dry MeCN (3.0 mL), $(TMS)_3SiH$ (185 uL, 0.6 mmol, 1.2 equiv), CH_2FI (68 µL, 1.0 mmol, 2.0 equiv), and alkyl iodide (1.0 mmol, 2.0 equiv). The vial was sealed with a septum, degassed by nitrogen bubbling for 10 seconds and wrapped with parafilm. The reaction mixture was stirred in a EvoluChemTM PhotoRedOx Box with a 18W blue LED lamp (λ = 450 nm) at room temperature (using a fan) for 16 hours. The solvent was removed *in vacuo* and the residue was purified by column chromatography (silica, Et_2O in pentane 40/60) to yield the desired product(s).

1-benzyl-3-methylpyrrolidine-2,5-dione (5a)

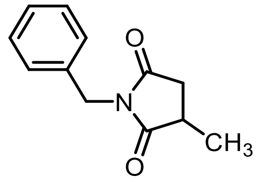

General procedure 1 was followed to obtain **5a** (25 mg, 0.12 mmol, 25%) as a colourless oil. **^{1}H NMR** (400 MHz, $CDCl_3$) δ 7.26 – 7.22 (m, 1H), 7.22 – 7.12 (m, 1H), 4.52 (d, J = 2.1 Hz, 1H), 2.85 – 2.67 (m, 1H), 2.19 (dd, J = 21.7, 13.6 Hz, 1H), 1.20 (d, J = 7.2 Hz, 1H); **^{13}C NMR** (101 MHz, $CDCl_3$) δ 180.3, 176.1, 136.0, 128.8, 128.7, 128.0, 42.5, 36.5, 34.8, 16.8; **MS** (ESI) m/z = 204.0 $[M+H]^+$. All data were in accordance with the literature.[8]

1-benzyl-3-ethylpyrrolidine-2,5-dione (6)

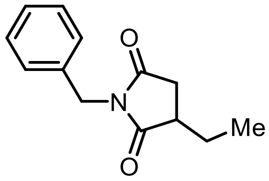

General procedure 1 was followed to obtain **6** (47 mg, 0.22 mmol, 43%) as a colourless oil. **^{1}H NMR** (400 MHz, $CDCl_3$) δ 7.40 – 7.19 (m, 5H), 4.62 (d, *J* = 1.5 Hz, 2H), 2.85 – 2.67 (m, 2H), 2.35 (dd, *J* = 13.6, 3.8 Hz, 1H), 1.95 – 1.81 (m, 1H), 1.56 (ddq, *J* = 13.7, 8.2, 7.3 Hz, 1H), 0.92 (t, *J* = 7.4 Hz, 3H); **^{13}C NMR** (101 MHz, $CDCl_3$) δ 179.6, 176.4, 136.0, 128.8, 128.7, 128.0, 42.4, 41.2, 33.9, 24.4, 10.9; **MS** (ESI) m/z = 218.0 $[M+H]^+$. All data were in accordance with the literature.[9]

1-benzyl-3-isopropylpyrrolidine-2,5-dione (7)

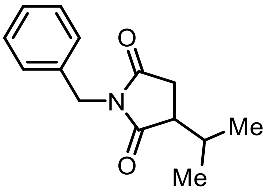

General procedure 1 was followed to obtain **7** (72 mg, 0.31 mmol, 62%) as a colourless oil. **^{1}H NMR** (400 MHz, $CDCl_3$) δ 7.35 – 7.16 (m, 5H), 4.58 (s, 2H), 2.72 (dt, *J* = 8.9, 4.3 Hz, 1H), 2.61 (dd, *J* = 18.2, 9.2 Hz, 1H), 2.39 (dd, *J* = 18.3, 4.4 Hz, 1H), 2.24 (hepd, *J* = 6.9, 4.3 Hz, 1H), 0.91 (d, *J* = 6.9 Hz, 3H), 0.75 (d, *J* = 6.8 Hz, 3zH); **^{13}C NMR** (101 MHz, $CDCl_3$) δ 179.1, 176.6, 136.0, 128.8, 128.6, 127.9, 45.7, 42.3, 30.4, 28.9, 20.0, 17.3; **HRMS** (ESI-TOF) calculated for $C_{14}H_{18}NO_2$ [M+H]+: 232.1332, found 232.1334; **IR** (neat) 2960, 1773, 1396, 1339, 1166, 1082, 695.

1-benzyl-3-(tert-butyl)pyrrolidine-2,5-dione (8)

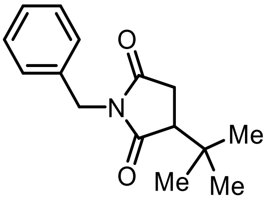

General procedure 1 was followed to obtain **8** (89 mg, 0.37 mmol, 73%) as a white solid. **^{1}H NMR** (400 MHz, $CDCl_3$) δ 7.34 – 7.15 (m, 5H), 4.58 (s, 2H), 2.70 – 2.41 (m, 3H), 0.95 (s, 9H); **^{13}C NMR** (101 MHz, $CDCl_3$) δ 178.2, 176.2, 136.1, 128.7, 128.6, 127.9, 49.8, 42.2, 33.5, 31.8, 27.2; **MS** (ESI) m/z = 246.0 $[M+H]^+$. All data were in accordance with the literature.[10]

13. Limitations

This hydrofluoromethylation protocol is best suited for electron-deficient alkenes. When the hydrofluoromethylation protocol was applied to a simple styrene the desired product was obtained in low yield.

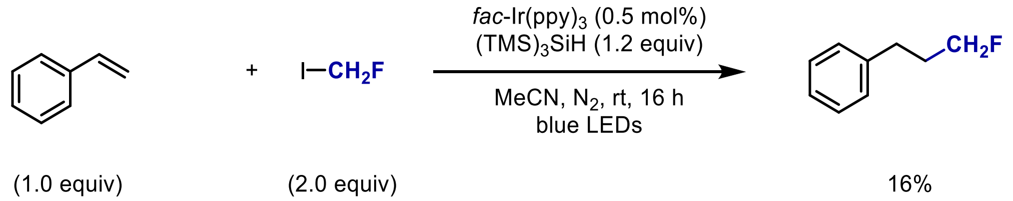

Similarly, a simple unactivated alkene only afforded 13% of the desired hydrofluoromethylated product. To be applicable to alkenes with different electronic profiles, we sought to investigate polarity reversal catalysis. We screened different HAD in the hope to further increase the yield of this transformation. However, the use of a thiol catalyst or other potential catalysts only resulted in trace amounts of the desired product.

Table S10: Screening of HAD for the hydrofluoromethylation of *N*-allyl-4-bromobenzamide.[a]

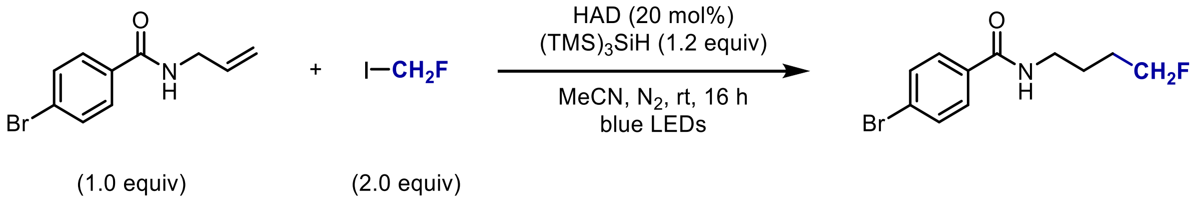

entry	HAD	yield[b]
**1**	No HAD, no $(TMS)_3SiH$	0%
**2**	-	13%
**3**	Hantzsch ester	0%
**4**	4-mercaptophenol	traces
**5**	cyclohexanethiol	traces
**6**	*N*-hydroxyphtalimide	9%

[a] Reaction conditions: *N*-allyl-4-bromobenzamide (0.1 mmol), fluoroiodomethane (0.2 mmol), HAD (20 mol%), $(TMS)_3SiH$ (0.12 mmol), MeCN (0.6 mL) under nitrogen atmosphere and blue light (λ_{max} = 470 nm) irradiation for 16 hours. [b] The yield was determined by quantitative ^{19}F NMR spectroscopy using *α*,*α*,*α*-trifluorotoluene as internal standard.

A further screening of solvents and reaction conditions revealed that using 4.0 equivalents of fluoroiodomethane, 2.0 equivalents of $(TMS)_3SiH$, 4CzIPN as an organophotocatalyst and $MeCN/H_2O$ (1:1) as solvent mixture, allowed to increase the yield to 21-26%.

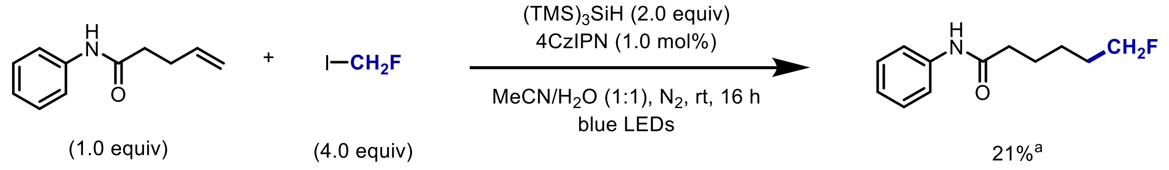

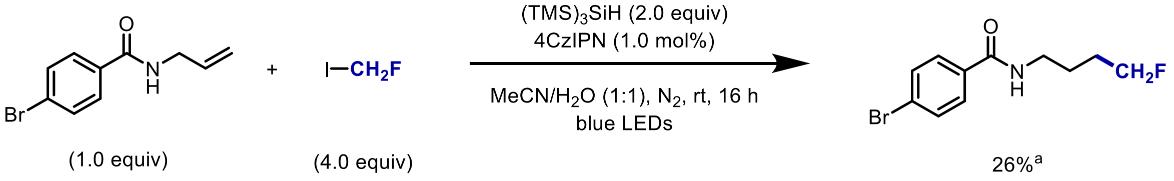

[a] The yield was determined by quantitative ^{19}F NMR spectroscopy using *α*,*α*,*α*-trifluorotoluene as internal standard.

14. Isolation of $(TMS)_3SiOH$

We noticed that $(TMS)_3SiOH$ was consistently formed as a by-product under our reaction conditions (upon purification by silica column chromatography). Considering its commercial value and synthetic utility we set out to recover it and were able to isolate it in high yield.

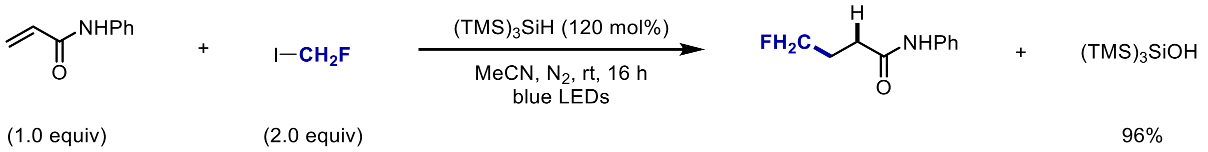

To a 7mL vial equipped with a stir bar was added *N*-phenylacrylamide (74.0 mg, 0.5 mmol, 1.0 equiv), MeCN (3.0 mL), $(TMS)_3SiH$ (185 µL, 0.6 mmol, 1.2 equiv), CH_2FI (68 µL, 1.0 mmol, 2.0 equiv). The vial was sealed with a septum, degassed by nitrogen bubbling for 10 seconds and wrapped with parafilm. The reaction mixture was stirred in a EvoluChemTM PhotoRedOx Box with a 18W blue LED lamp (λ = 450 nm) at room temperature (using a fan) for 16 hours. The reaction mixture was treated with an aqueous solution of saturated $NaHCO_3$ and extracted three times with DCM. The organic layers were dried (Na_2SO_4), filtered and concentrated *in vacuo*. The crude product was purified by column chromatography (silica, EtOAc in heptane 0/100 to 100/0) to yield the desired product (152 mg, 0.57 mmol, 96%, 114 mol%) as a colourless oil. **^{1}H NMR** (400 MHz, CD_3CN) δ 1.80 (s, 1H), 0.15 (s, 27 H); **^{13}C NMR** (101 MHz, CD_3CN) δ -0.25. All data were in accordance with the literature.[11]

15. General procedures

General procedure A: To a 7 mL vial equipped with a magnetic stir bar, was added alkene (0.50 mmol, 1.0 equiv), MeCN (dry; 3.0 mL), $(TMS)_3SiH$ (185 uL, 0.6 mmol, 1.2 equiv) and CH_2FI (68 µL, 1.0 mmol, 2.0 equiv). The vial was sealed with a septum, degassed by nitrogen bubbling for 10 seconds and wrapped with parafilm. The reaction mixture was stirred in a EvoluChemTM PhotoRedOx Box with a 18W blue LED lamp (λ = 450 nm) at room temperature (using a fan) for 16 hours. The solvent was removed *in vacuo* and the residue was purified by column chromatography (silica, EtOAc in heptane 0/100 to 100/0) to yield the desired product(s).

General procedure B: To a 7 mL vial equipped with a magnetic stir bar, was added alkene (0.50 mmol, 1.0 equiv), *fac*-$Ir(ppy)_3$ (1.6 mg, 0.0025 mmol, 0.5 mol%), MeCN (dry; 3.0 mL), $(TMS)_3SiH$ (185 uL, 0.6 mmol, 1.2 equiv) and CH_2FI (68 µL, 1.0 mmol, 2.0 equiv). The vial was sealed with a septum, degassed by nitrogen bubbling for 10 seconds and wrapped with parafilm. The reaction mixture was stirred in a EvoluChemTM PhotoRedOx Box with a 18W blue LED lamp (λ = 450 nm) at room temperature (using a fan) for 16 hours. The solvent was removed *in vacuo* and the residue was purified by column chromatography (silica, EtOAc in heptane 0/100 to 100/0) to yield the desired product(s).

General procedure C: To a 7 mL vial equipped with a magnetic stir bar, was added alkene (0.50 mmol, 1.0 equiv), $MesAcrBF_4$ (1.0 mg, 0.0025 mmol, 0.5 mol%), difluorobenzene (3.0 mL), $(TMS)_3SiH$ (463 uL, 1.5 mmol, 3.0 equiv) and MeI (125 µL, 2.0 mmol, 4.0 equiv). The vial was sealed with a septum, degassed by nitrogen bubbling for 10 seconds and wrapped with parafilm. The reaction mixture was stirred in a EvoluChemTM PhotoRedOx Box with a 18W blue LED lamp (λ = 450 nm) at room temperature (using a fan) for 16 hours. The solvent was removed *in vacuo* and the residue was purified by column chromatography (silica, EtOAc in heptane 0/100 to 100/0) to yield the desired product(s).

16. Characterization

16.1. Hydrofluoromethylation of electron-deficient alkenes

4-fluoro-*N*-phenylbutanamide (2a)

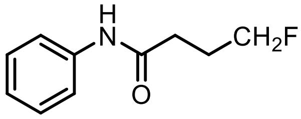

General procedure A was followed to obtain **2a** (64 mg, 0.36 mmol, 71%) as a white solid. **^{1}H NMR** (400 MHz, $CDCl_3$) δ 7.57 (s, 1H), 7.50 (d, *J* = 7.9 Hz, 2H), 7.30 (t, *J* = 7.9 Hz, 2H), 7.10 (t, *J* = 7.4 Hz, 1H), 4.52 (dt, *J* = 47.3, 5.7 Hz, 2H), 2.50 (t, *J* = 7.3 Hz, 2H), 2.19 – 2.01 (m, 2H); **^{13}C NMR** (101 MHz, $CDCl_3$) δ 170.5, 137.9, 129.1, 124.5, 120.1, 83.3 (d, $J_{C\text{-}F}$ = 164.6 Hz), 33.1 (d, $J_{C\text{-}F}$ = 4.3 Hz), 26.3 (d, $J_{C\text{-}F}$ = 20.0 Hz); **^{19}F NMR** (377 MHz, $CDCl_3$) δ -220.5 (tt, *J* = 47.4, 27.0 Hz); **HRMS** (ESI-TOF) calculated for $C_{10}H_{13}FNO$ $[M+H]^+$: 182.0976, found 182.0977; **IR** (neat) 1656, 1620, 1597, 1542, 1502, 1489, 1441, 1393, 1374, 1331, 1297, 1258, 1035, 901, 758, 694, 634; **m.p.**: 76 – 78 °C.

***N*-(4-bromophenyl)-4-fluorobutanamide (2b)**

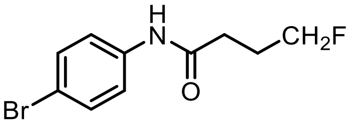

General procedure B was followed to obtain **2b** (109 mg, 0.42 mmol, 84%) as a white solid. **^{1}H NMR** (400 MHz, $CDCl_3$) δ 7.45 – 7.38 (m, 4H), 7.32 (s, 1H), 4.53 (dt, *J* = 47.3, 5.6 Hz, 2H), 2.51 (t, *J* = 7.2 Hz, 2H), 2.19 – 2.03 (m, 2H); **^{13}C NMR** (101 MHz, $CDCl_3$) δ 170.4, 136.9, 132.1, 121.5, 117.1, 83.2 (d, $J_{C\text{-}F}$ = 164.8 Hz), 33.2 (d, $J_{C\text{-}F}$ = 4.1 Hz), 26.2 (d, $J_{C\text{-}F}$ = 19.9 Hz); **^{19}F NMR** (377 MHz, $CDCl_3$) δ -220.7 (tt, *J* = 47.5, 27.3 Hz). **HRMS** (ESI-TOF) calculated for $C_{10}H_{12}{}^{79}BrFNO$ $[M+H]^+$: 260.0081, found 260.0083; **IR** (neat) 1653, 1589, 1528, 1488, 1441, 1396, 1336, 1297, 1281, 1254, 1177, 1099, 1072, 1032, 1011, 980, 896, 849, 819, 785, 692, 645; **m.p.**: 87 – 89 °C

N-(4-cyanophenyl)-4-fluorobutanamide (2c)

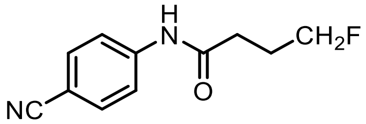

General procedure B was followed to obtain **2c** (53 mg, 0.26 mmol, 51%) as a white solid. **^{1}H NMR** (400 MHz, $CDCl_3$) δ 8.04 (s, 1H), 7.71 – 7.66 (m, 2H), 7.61 – 7.56 (m, 2H), 4.53 (dt, J = 47.2, 5.6 Hz, 2H), 2.56 (t, J = 7.3 Hz, 2H), 2.19 – 2.03 (m, 2H); **^{13}C NMR** (101 MHz, $CDCl_3$) δ 171.1, 142.3, 133.4, 119.7, 119.1, 106.8, 83.1 (d, $J_{C\text{-}F}$ = 164.9 Hz), 33.2 (d, $J_{C\text{-}F}$ = 4.2 Hz), 26.0 (d, $J_{C\text{-}F}$ = 19.9 Hz); **^{19}F NMR** (377 MHz, $CDCl_3$) δ -220.6 (tt, J = 47.3, 27.1 Hz); **HRMS** (ESI-TOF) calculated for $C_{11}H_{10}FN_2O$ $[M-H]^-$: 205.0783, found 205.0777; **IR** (neat) 2237, 2220, 1703, 1677, 1596, 1523, 1437, 1409, 1377, 1324, 1254, 1174, 1174, 1079, 1061, 1035, 967, 899, 842, 733; **m.p.**: 122 – 124 °C.

4-fluoro-N-(4-methoxyphenyl)butanamide (2d)

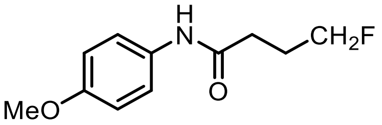

General procedure B was followed to obtain **2d** (97 mg, 0.46 mmol, 92%) as a white solid. **^{1}H NMR** (400 MHz, $CDCl_3$) δ 7.47 (s, 1H), 7.42 – 7.35 (m, 2H), 6.87 – 6.80 (m, 2H), 4.52 (dt, J = 47.2, 5.7 Hz, 2H), 3.77 (s, 3H), 2.47 (t, J = 7.3 Hz, 2H), 2.19 – 2.01 (m, 2H); **^{13}C NMR** (101 MHz, $CDCl_3$) δ 170.2, 156.6, 131.0, 122.0, 114.3, 83.3 (d, $J_{C\text{-}F}$ = 164.7 Hz), 55.6, 32.9 (d, $J_{C\text{-}F}$ = 4.4 Hz), 26.4 (d, J = 19.9 Hz); **^{19}F NMR** (377 MHz, $CDCl_3$) δ -220.5 (tt, J = 47.3, 27.0 Hz); **HRMS** (ESI-TOF) calculated for $C_{11}H_{15}FNO_2$ $[M+H]^+$: 212.1081, found 212.1084; **IR** (neat) 1650, 1535, 1510, 1412, 1377, 1341, 1304, 1239, 1186, 1169, 1031, 981, 899, 825, 796, 759, 728, 704, 680, 606; **m.p.**: 106 – 108 °C.

4-fluoro-*N*-(pyridin-3-yl)butanamide (2e)

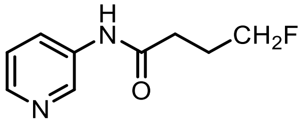

General procedure A was followed to obtain **2e** (52 mg, 0.29 mmol, 57%) as a white solid. **^{1}H NMR** (400 MHz, $CDCl_3$) δ 8.60 (s, 1H), 8.31 (d, J = 4.2 Hz, 1H), 8.19 (d, J = 8.2 Hz, 1H), 7.28 (dd, J = 8.2, 4.6 Hz, 1H), 4.52 (dt, J = 47.3, 5.7 Hz, 2H), 2.55 (t, J = 7.2 Hz, 2H), 2.18 – 2.03 (m, 2H); **^{13}C NMR** (101 MHz, $CDCl_3$) δ 171.4, 144.8, 141.0, 135.4, 127.7, 124.1, 83.2 (d, $J_{C\text{-}F}$ = 164.8 Hz), 32.9 (d, $J_{C\text{-}F}$ = 4.3 Hz), 26.1 (d, $J_{C\text{-}F}$ = 20.0 Hz); **^{19}F NMR** (377 MHz, $CDCl_3$) δ -220.5 (tt, J = 47.3, 27.1 Hz); **HRMS** (ESI-TOF) calculated for $C_9H_{12}FN_2O$ $[M+H]^+$: 183.0928; found 183.0929; **IR** (neat) 1688, 1610, 1584, 1549, 1476, 1422, 1375, 1330, 1271, 1248, 1194, 1172, 1150, 1130, 1103, 1077, 1032, 967, 896, 855, 808, 750, 704, 624; **m.p.**: 70 – 72 °C.

***N*-(benzo[d]thiazol-2-yl)-4-fluorobutanamide (2f)**

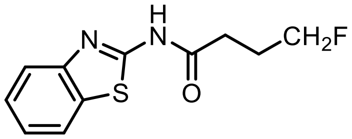

General procedure A was followed to obtain **2f** (82 mg, 0.34 mmol, 69%) as a white solid. **^{1}H NMR** (400 MHz, Acetone-d_6) δ 11.17 (s, 1H), 7.95 – 7.90 (m, 1H), 7.73 – 7.68 (m, 1H), 7.45 – 7.39 (m, 1H), 7.33 – 7.27 (m, 1H), 4.56 (dt, J = 47.4, 6.0 Hz, 2H), 2.77 (t, J = 7.3 Hz, 2H), 2.21 – 2.07 (m, 2H); **^{13}C NMR** (101 MHz, Acetone-d_6) δ 172.0, 158.7, 149.9, 133.1, 126.8, 124.4, 122.2, 121.7, 83.8 (d, $J_{C\text{-}F}$ = 163.5 Hz), 32.1 (d, $J_{C\text{-}F}$ = 5.5 Hz), 26.4 (d, $J_{C\text{-}F}$ = 20.2 Hz); **^{19}F NMR** (377 MHz, Acetone-d_6) δ -220.5 (tt, J = 47.6, 25.2 Hz); **HRMS** (ESI-TOF) calculated for $C_{11}H_{12}FN_2O^{32}S$ $[M+H]^+$: 239.0649; found 239.0650; **IR** (neat) 1697, 1599, 1548, 1445, 1419, 1380, 1342, 1267, 1254, 1190, 1166, 1097, 1082, 1039, 1022, 994, 939, 906, 867, 782, 758, 683; **m.p.**: 176 – 178 °C.

((3-fluoropropyl)sulfonyl)benzene (2g)

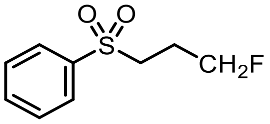

General procedure A was followed to obtain **2g** (71 mg, 0.34 mmol, 68%) as a pale yellow oil. **^{1}H NMR** (400 MHz, $CDCl_3$) δ 7.94 – 7.88 (m, 2H), 7.70 – 7.63 (m, 1H), 7.61 – 7.53 (m, 2H), 4.50 (dt, J = 46.9, 5.7 Hz, 2H), 3.26 – 3.19 (m, 2H), 2.21 – 2.04 (m, 2H); **^{13}C NMR** (101 MHz, $CDCl_3$) δ 139.0, 134.0, 129.5, 128.1, 81.6 (d, $J_{C\text{-}F}$ = 167.8 Hz), 52.6 (d, $J_{C\text{-}F}$ = 4.2 Hz), 24.2 (d, $J_{C\text{-}F}$ = 20.8 Hz); **^{19}F NMR** (377 MHz, $CDCl_3$) δ -220.5 (tt, J = 47.1, 25.9 Hz); **HRMS** (ESI-TOF) calculated for $C_9H_{12}FO_2{}^{32}S$ $[M+H]^+$: 203.0537; found 203.0539; **IR** (neat) 1477, 1307, 1142, 1087, 1058, 1022, 889, 732, 689, 625.

Benzyl 4-fluorobutanoate (2h)

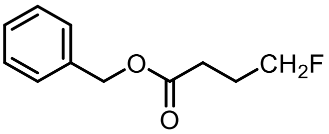

General procedure A was followed to obtain **2h** (64 mg, 0.33 mmol, 65%) as a colourless oil. **^{1}H NMR** (400 MHz, $CDCl_3$) δ 7.41 – 7.30 (m, 5H), 5.14 (s, 2H), 4.49 (dt, J = 47.1, 5.8 Hz, 2H), 2.52 (t, J = 7.4 Hz, 2H), 2.13 – 1.98 (m, 2H); **^{13}C NMR** (101 MHz, $CDCl_3$) δ 172.8, 136.0, 128.7, 128.4, 128.4, 83.0 (d, $J_{C\text{-}F}$ = 165.4 Hz), 66.5, 30.1 (d, $J_{C\text{-}F}$ = 5.1 Hz), 25.9 (d, $J_{C\text{-}F}$ = 20.3 Hz); **^{19}F NMR** (377 MHz, $CDCl_3$) δ -220.6 (tt, J = 47.2, 26.0 Hz). **GC-MS** (EI-TOF) calculated for $C_{11}H_{13}FO_2$ $[M]^+$: 196.0894; found 196.0891; **IR** (neat) 1734, 1498, 1456, 1422, 1384, 1351, 1317, 1247, 1213, 1165, 1083, 1037, 909, 839, 738, 697, 622.

The product coeluted with silane by-products (δ: 0.19 ppm).

Benzyl 4-fluorobutanoate-2,3,3-*d*$_3$ (2i)

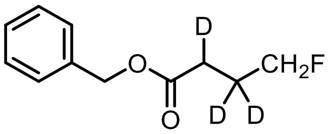

General procedure B was followed to obtain **2i** (77 mg, 0.39 mmol, 77%) as a colourless oil. ^{1}**H NMR** (400 MHz, $CDCl_3$) δ 7.42 – 7.29 (m, 5H), 5.14 (s, 2H), 4.48 (d, J = 47.1 Hz, 2H), 2.49 (s, 1H). ^{13}**C NMR** (101 MHz, $CDCl_3$) δ 172.8, 136.0, 128.7, 128.4, 128.3, 82.9 (d, J_{C-F} = 165.3 Hz), 66.5, 30.2 – 29.1 (m), 26.0 – 24.2 (m). ^{19}**F NMR** (377 MHz, $CDCl_3$) δ -221.1 (tdt, J = 47.0, 7.8, 3.9 Hz); **HRMS** (ESI-TOF) calculated for $C_{11}H_{10}D_3FNaO_2$ $[M+Na]^+$: 222.0980; found 222.0982; **IR** (neat) 2962, 2904, 2161, 1732, 1498, 1456, 1379, 1328, 1199, 1174, 1110, 1016, 912, 840, 738, 697.

Benzyl 4-fluoro-2-methylbutanoate (2j)

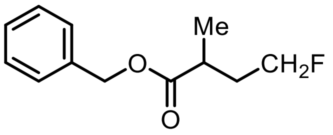

General procedure A was followed to obtain **2j** (66 mg, 0.32 mmol, 63%) as a colourless oil. ^{1}**H NMR** (400 MHz, $CDCl_3$) δ 7.43 – 7.29 (m, 5H), 5.13 (s, 2H), 4.61 – 4.36 (m, 2H), 2.72 (dq, J = 14.3, 7.1 Hz, 1H), 2.22 – 2.03 (m, 1H), 1.91 – 1.71 (m, 1H), 1.24 (d, J = 7.0 Hz, 3H); ^{13}**C NMR** (101 MHz, $CDCl_3$) δ 175.9, 136.1, 128.7, 128.4, 128.2, 82.0 (d, J_{C-F} = 165.0 Hz), 66.5, 36.0 (d, J_{C-F} = 4.4 Hz), 34.2 (d, J_{C-F} = 19.9 Hz), 17.2; ^{19}**F NMR** (377 MHz, $CDCl_3$) δ 7.3 (tt, J = 47.2, 26.1 Hz); **HRMS** (ESI-TOF) calculated for $C_{12}H_{15}FNaO_2$ $[M+Na]^+$: 233.0948; found 233.0951; **IR** (neat) 2919, 1732, 1498, 1456, 1384, 1257, 1170, 1136, 1046, 992, 849, 751, 698.

***N*-benzyl-2-(fluoromethyl)cyclobutane-1-carboxamide (2k)**

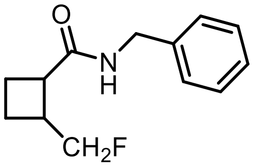

General procedure B was followed to obtain **2k** (60 mg, 0.27 mmol, 54%, d.r.: 68/32) as a pale yellow oil. **^{1}H NMR** (400 MHz, $CDCl_3$) δ 7.37 – 7.26 (m, 5H), 5.98 (s, 0.32H), 5.93 (s, 0.63H), 4.72 – 4.28 (m, 4H), 3.24 – 3.15 (m, 0.65H), 3.08 – 2.83 (m, 1.36H), 2.47 – 2.37 (m, 0.64H), 2.18 – 2.03 (m, 2H), 2.00 – 1.77 (m, 1.36H); **^{13}C NMR** (101 MHz, $CDCl_3$) δ 173.6, 172.4, 138.5, 138.4, 128.8, 128.8, 127.9, 127.7, 127.5, 127.5, 85.9 (d, J = 165.7 Hz), 84.1 (d, $J_{C\text{-}F}$ = 166.0 Hz), 43.6 (d, $J_{C\text{-}F}$ = 11.9 Hz), 42.0 (d, $J_{C\text{-}F}$ = 4.4 Hz), 40.8 (d, $J_{C\text{-}F}$ = 5.3 Hz), 38.9 (d, $J_{C\text{-}F}$ = 19.0 Hz), 37.5 (d, $J_{C\text{-}F}$ = 18.3 Hz), 21.4 (d, $J_{C\text{-}F}$ = 17.5 Hz), 20.4 (d, $J_{C\text{-}F}$ = 9.6 Hz), 19.2 (d, $J_{C\text{-}F}$ = 8.4 Hz); **^{19}F NMR** (377 MHz, $CDCl_3$) δ -219.7 (td, J = 47.2, 18.9 Hz), -222.2 (td, J = 47.9, 22.0 Hz); **HRMS** (ESI-TOF) calculated for $C_{13}H_{17}FNO$ $[M+H]^+$: 222.1289; found 222.1290; **IR** (neat) 1642, 1539, 1497, 1454, 1381, 1355, 1325, 1247, 1079, 1029, 992, 733, 698.

Cis- and *trans*- products could not be separated upon purification and were isolated as a mixture of products (d.r.: 68/32).

2-(2-fluoro-1-phenylethyl)malononitrile (2l)

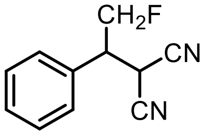

General procedure B was followed to obtain **2l** (54 mg, 0.29 mmol, 57%) as a pale yellow oil. **^{1}H NMR** (400 MHz, $CDCl_3$) δ 7.45 (s, 3H), 7.39 – 7.34 (m, 2H), 4.87 (ddd, J = 46.3, 6.2, 2.6 Hz, 2H), 4.29 (d, J = 6.3 Hz, 1H), 3.61 (dddd, J = 17.0, 7.4, 6.4, 5.1 Hz, 1H); **^{13}C NMR** (101 MHz, $CDCl_3$) δ 132.6 (d, $J_{C\text{-}F}$ = 6.2 Hz), 129.9, 129.7, 128.3, 111.3 (d, $J_{C\text{-}F}$ = 34.8 Hz), 82.3 (d, $J_{C\text{-}F}$ = 176.7 Hz), 46.8 (d, $J_{C\text{-}F}$ = 18.3 Hz), 26.2 (d, $J_{C\text{-}F}$ = 5.0 Hz); **^{19}F NMR** (377 MHz, $CDCl_3$) δ -220.9 (td, J = 46.7, 17.0 Hz); **HRMS** (ESI-TOF) calculated for $C_{11}H_8FN$ $[M-H]^-$: 187.0677; found 187.0666; **IR** (neat) 2912, 2630, 1498 1456, 1160, 1068, 1012, 947, 815, 763, 700, 653, 620.

(3-fluoropropane-1,1-diyl)dibenzene (2m)

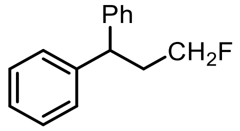

General procedure B was followed to obtain **2m** (33 mg, 0.16 mmol, 31%) as a colourless oil. ^{1}**H NMR** (400 MHz, $CDCl_3$) δ 7.43 – 7.21 (m, 0H), 4.46 (dt, J = 47.0, 6.0 Hz, 1H), 4.24 (t, J = 8.0 Hz, 1H), 2.51 (ddt, J = 24.0, 7.9, 6.0 Hz, 1H); ^{13}**C NMR** (101 MHz, $CDCl_3$) δ 144.0, 128.7, 128.0, 126.6, 82.2 (d, $J_{C\text{-}F}$ = 164.7 Hz), 46.6 (d, $J_{C\text{-}F}$ = 5.1 Hz), 36.2 (d, $J_{C\text{-}F}$ = 19.8 Hz); ^{19}**F NMR** (377 MHz, $CDCl_3$) δ -220.8 (tt, J = 47.4, 24.0 Hz). All data were in accordance with the literature.[12]

Note: The product was purified by column chromatography with an alternative eluent to facilitate separation (silica, toluene in pentane, 0/100 to 20/80).

4-(3-fluoropropyl)pyridine (2n)

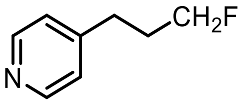

General procedure B was followed to obtain **2n** (29 mg, 0.21 mmol, 42%) as a colourless oil. ^{1}**H NMR** (400 MHz, $CDCl_3$) δ 8.53 (s, 2H), 7.15 (d, J = 4.4 Hz, 2H), 4.46 (dt, J = 47.1, 5.8 Hz, 2H), 2.80 – 2.72 (m,2H), 2.11 – 1.93 (m, 2H); ^{13}**C NMR** (101 MHz, $CDCl_3$) δ 150.1, 150.0, 124.0, 82.8 (d, J = 165.7 Hz), 31.0 - 30.8 (m); ^{19}**F NMR** (377 MHz, $CDCl_3$) δ -220.4 (tt, J = 46.9, 25.7 Hz) All data were in accordance with the literature.[13]

Note: The product was purified by column chromatography with an alternative eluent to facilitate separation (silica, methanol in DCM, 2/98 + 1% NEt_3).

1-benzyl-3-(fluoromethyl)pyrrolidine-2,5-dione (2o)

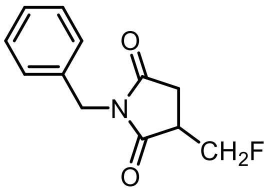

General procedure A was followed to obtain **2o** (99 mg, 0.45 mmol, 90%) as a pale yellow oil. **^{1}H NMR** (400 MHz, $CDCl_3$) δ 7.27 – 7.09 (m, 5H), 4.75 (ddd, J = 46.4, 9.3, 3.5 Hz, 1H), 4.59 – 4.36 (m, 2H), 4.54 (d, J = 5.5 Hz, 1H), 2.98 – 2.83 (m, 1H), 2.79 – 2.58 (m, 2H); **^{19}F NMR** (377 MHz, $CDCl_3$) δ -230.1 (td, J = 46.8, 33.3 Hz); **^{13}C NMR** (101 MHz, $CDCl_3$) δ 176.2 (d, J_{C-F} = 5.4 Hz), 175.4, 135.6, 128.8, 128.6, 128.1, 81.3 (d, J_{C-F} = 172.4 Hz), 42.8, 41.6 (d, J_{C-F} = 21.4 Hz), 30.9 (d, J = 4.0 Hz), 28.3; Mass not found, **HRMS** (ESI-TOF) calculated for $C_{12}H_{13}FNO_2$ $[M+H]^+$: 222.0925; found 222.0927; **IR** (neat) 1698, 1431, 1401, 1342, 1168, 1022, 938, 900, 755, 708, 696, 634.

(8*R*,9*S*,13*S*,14*S*)-13-methyl-17-oxo-7,8,9,11,12,13,14,15,16,17-decahydro-6*H*-cyclopenta[*a*]phenanthren-3-yl 4-fluorobutanoate (2t)

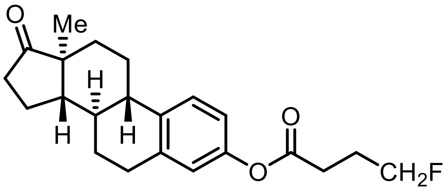

General procedure A was followed to obtain **2t** (91 mg, 0.26 mmol, 51%) as a white solid. **^{1}H NMR** (400 MHz, $CDCl_3$) δ 7.28 (d, J = 9.7 Hz, 1H), 6.85 (dd, J = 8.5, 2.6 Hz, 1H), 6.82 – 6.80 (m, 1H), 4.56 (dt, J = 47.1, 5.7 Hz, 2H), 2.94 – 2.88 (m, 2H), 2.71 (t, J = 7.3 Hz, 2H), 2.51 (dd, J = 19.0, 8.4 Hz, 1H), 2.45 – 2.23 (m, 2H), 2.21 – 1.93 (m, 6H), 1.69 – 1.40 (m, 6H), 0.91 (s, 3H); **^{13}C NMR** (101 MHz, $CDCl_3$) δ 220.9, 171.9, 148.6, 138.2, 137.6, 126.6, 121.6, 118.8, 83.0 (d, $J_{C\text{-}F}$ = 165.5 Hz), 50.6, 48.1, 44.3, 38.1, 36.0, 31.7, 30.3 (d, $J_{C\text{-}F}$ = 4.9 Hz), 29.5, 26.5, 25.9 (d, $J_{C\text{-}F}$ = 20.3 Hz), 25.9, 21.7, 14.0; **^{19}F NMR** (377 MHz, $CDCl_3$) δ -220.7 (tt, J = 47.1, 26.3 Hz); **HRMS** (ESI-TOF) calculated for $C_{22}H_{27}FNaO_3$ $[M+Na]^+$: 381.1836; found 381.1838; **IR** (neat) 2919, 2853, 1740, 1603, 1491, 1452, 1438, 1377, 1315, 1251, 1223, 1555, 1141, 1086, 1055, 1036, 1007, 953, 9090, 875, 824, 784, 719; **m.p.**: 94 – 96 °C.

(*R*)-1-(3-(4-amino-3-(4-phenoxyphenyl)-1*H*-pyrazolo[3,4-*d*]pyrimidin-1-yl)piperidin-1-yl)-4-fluorobutan-1-one (2u)

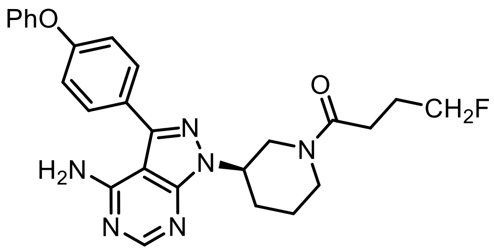

General procedure B was followed to obtain **2u** (137 mg, 0.29 mmol, 58%) as a yellow oil. **^{1}H NMR** (400 MHz, $CDCl_3$) δ 8.33 (d, J = 15.2 Hz, 1H), 7.63 (dd, J = 8.4, 4.0 Hz, 2H), 7.38 (t, J = 7.7 Hz, 2H), 7.20 – 7.11 (m, 3H), 7.07 (d, J = 8.0 Hz, 2H), 6.04 (s, 2H), 4.89 – 4.76 (m, 1.5H), 4.62 – 4.51 (m, 1.5H), 4.44 (dt, J = 12.6, 5.7 Hz, 1H), 4.07 (dd, J = 13.4, 4.2 Hz, 0.5H), 3.91 (d, J = 13.6 Hz, 0.5H), 3.69 (dd, J = 13.2, 10.5 Hz, 0.5H), 3.23 (dt, J = 61.6, 12.5 Hz, 2H), 2.79 (t, J = 11.7 Hz, 0.5H), 2.57 – 2.18 (m, 3H), 2.13 – 1.90 (m, 3H), 1.78 – 1.60 (m, 1H); **^{13}C NMR** (101 MHz, $CDCl_3$) δ 170.7, 170.6, 158.8, 158.8, 157.7, 157.4, 156.4, 156.4, 155.0, 154.3, 154.2, 153.9, 144.4, 130.1, 130.0, 127.6, 127.5, 124.2, 124.2, 119.7, 119.2, 98.6, 98.4, 83.6 (d, $J_{C\text{-}F}$ = 163.9 Hz), 53.5, 52.8, 49.8, 45.9, 45.6, 41.9, 30.3, 30.1, 28.8 (d, $J_{C\text{-}F}$ = 4.0 Hz), 26.2 (d, $J_{C\text{-}F}$ = 19.7 Hz), 26.1 (d, $J_{C\text{-}F}$ = 19.8 Hz), 25.2, 24.1; **^{19}F NMR** (377 MHz, $CDCl_3$) δ -220.2 (dtt, J = 128.4, 47.3, 27.3 Hz); **HRMS** (ESI-TOF) calculated for $C_{26}H_{28}FN_6O_2$ $[M+H]^+$: 475.2252; found 475.2249; **IR** (neat) 2924, 2856, 1626, 1587, 1567, 1521, 1489, 1440, 1285, 1236, 1167, 1135, 1104, 1070, 1025, 869, 803, 803, 757, 696.

Methyl (4-fluorobutanoyl)-*L*-tyrosinate (2v)

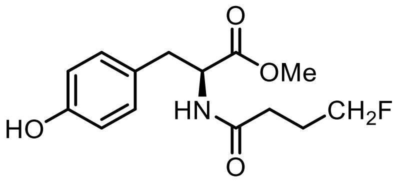

General procedure B was followed to obtain **2v** (93 mg, 0.33 mmol, 66%) as a pale yellow oil; **^{1}H NMR** (400 MHz, $CDCl_3$) δ 6.93 (d, *J* = 8.5 Hz, 2H), 6.72 (d, *J* = 8.5 Hz, 2H), 6.20 (br d, *J* = 8.1 Hz, 1H), 4.91 – 4.82 (m, 1H), 4.52 – 4.27 (m, 2H), 3.73 (s, 3H), 3.07 (dd, *J* = 14.1, 5.5 Hz, 1H), 2.95 (dd, *J* = 14.1, 6.6 Hz, 1H), 2.31 (dd, *J* = 7.9, 6.7 Hz, 2H), 2.06 – 1.86 (m, 2H); **^{13}C NMR** (101 MHz, $CDCl_3$) δ 172.4, 155.7, 130.3, 127.0, 115.7, 83.1 (d, *J* = 164.9 Hz), 53.4, 52.6, 37.3, 32.0 (d, *J* = 4.6 Hz), 26.2 (d, *J* = 20.0 Hz) (carbonyl peaks overlapping); **^{19}F NMR** (377 MHz, $CDCl_3$) δ -220.1 – -220.9 (m); **HRMS** (ESI-TOF) calculated for $C_{14}H_{17}FNO_4$ $[M+H]^+$: 284.1293; found 282.1292; **IR** (neat) 3297, 2956, 2361, 1736, 1649, 1614, 1596, 1537, 1515, 1440, 1369, 1219, 1174, 1124, 1105, 1033, 905, 829, 802, 668.

Benzyl 2-(2,3-dichloro-4-(2-ethyl-4-fluorobutanoyl)phenoxy)acetate (2w)

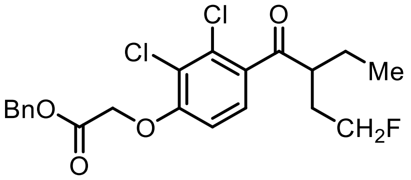

General procedure B was followed to obtain **2w** (62 mg, 0.29 mmol, 57%) as a colourless oil. **^{1}H NMR** (400 MHz, $CDCl_3$) δ 7.40 – 7.32 (m, 5H), 7.30 (d, *J* = 8.6 Hz, 1H), 6.72 (d, *J* = 8.7 Hz, 1H), 5.25 (s, 2H), 4.79 (s, 2H), 4.68 – 4.33 (m, 1H), 3.49 – 3.35 (m, 1H), 2.31 – 2.09 (m, 1H), 2.00 – 1.80 (m, 1H), 1.76 (ddd, *J* = 14.1, 7.7, 6.6 Hz, 1H), 1.65 – 1.47 (m, 2H), 0.91 (t, *J* = 7.4 Hz, 3H); **^{13}C NMR** (101 MHz, $CDCl_3$) δ 204.2, 167.6, 156.2, 134.9, 134.5, 131.9, 128.9, 128.8, 128.7, 127.4, 124.2, 110.8, 82.3 (d, *J* = 164.5 Hz), 67.5, 66.3, 47.8 (d, *J* = 3.6 Hz), 30.9 (d, *J* = 19.7 Hz), 24.8, 11.3; **^{19}F NMR** (377 MHz, $CDCl_3$) δ -219.1 (tdd, *J* = 47.4, 30.8, 22.7 Hz); **HRMS** (ESI-TOF) calculated for $C_{21}H_{22}{}^{35}Cl_2FO_4$ $[M+H]^+$: 427.0874; found 427.0873; **IR** (neat) 2965, 1757, 1694, 1583, 1498, 1466, 1385, 1303, 1264, 1191, 1121, 1076, 996, 894, 811, 751, 698, 645.

16.2. Hydro(poly)fluoroalkylation of alkenes

1-benzyl-3-(2-fluoroethyl)pyrrolidine-2,5-dione (3a)

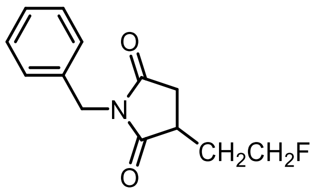

General procedure A was followed to obtain **3a** (86 mg, 0.37 mmol, 73%) as a pale-yellow oil. **^{1}H NMR** (400 MHz, $CDCl_3$) δ 7.34 – 7.27 (m, 2H), 7.27 – 7.17 (m, 3H), 4.66 – 4.38 (m, 4H), 2.96 – 2.79 (m, 2H), 2.51 – 2.36 (m, 1H), 2.34 – 2.12 (m, 1H), 1.94 – 1.73 (m, 1H); **^{13}C NMR** (101 MHz, $CDCl_3$) δ 178.9, 175.8, 135.8, 128.7, 128.7, 128.0, 81.8 (d, $J_{C\text{-}F}$ = 166.5 Hz), 42.5, 37.1 (d, $J_{C\text{-}F}$ = 3.5 Hz), 34.5, 31.8 (d, $J_{C\text{-}F}$ = 19.6 Hz); **^{19}F NMR** (377 MHz, $CDCl_3$) δ -220.6 (tdd, J = 47.1, 28.7, 24.3 Hz); **HRMS** (ESI-TOF) calculated for $C_{13}H_{15}FNO_2$ $[M+H]^+$: 236.1081; found 236.1084; **IR** (neat) 1174, 1696, 1497, 1431, 1397, 1342, 1314, 1168, 1081, 1032, 1001, 949, 929, 897, 857, 707, 635.

1-benzyl-3-(2,2-difluoroethyl)pyrrolidine-2,5-dione (3b)

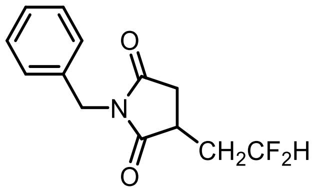

General procedure A was followed to obtain **3b** (38 mg, 0.15 mmol, 30%) as a pale-yellow oil. **^{1}H NMR** (400 MHz, $CDCl_3$) δ 7.40 – 7.27 (m, 5H), 6.06 (tdd, J = 55.9, 4.8, 3.1 Hz, 1H), 4.66 (s, 2H), 3.10 – 2.92 (m, 2H), 2.62 – 2.41 (m, 2H), 2.10 – 1.92 (m, 1H); **^{13}C NMR** (101 MHz, $CDCl_3$) δ 178.1, 175.4, 135.6, 129.0, 128.9, 128.3, 115.6 (t, $J_{C\text{-}F}$ = 240.0 Hz), 42.8, 35.2 (t, $J_{C\text{-}F}$ = 21.7 Hz), 35.0, 34.5 (t, $J_{C\text{-}F}$ = 4.0 Hz); **^{19}F NMR** (377 MHz, $CDCl_3$) δ -115.2 (dddd, J = 285.1, 55.7, 18.7, 12.4 Hz), -118.0 (ddt, J = 285.0, 56.1, 19.3 Hz); **HRMS** (ESI-TOF) calculated for $C_{13}H_{14}F_2NO_2$ $[M+H]^+$: 254.0987; found 254.0987; **IR** (neat) 2937, 1777, 1698, 1586, 1456, 1432, 1399, 1344, 1314, 1292, 1170, 1122, 1088, 1053, 978, 964, 930, 906, 864, 825, 755, 708, 696, 650, 631.

2-(3-fluoro-1-phenylpropyl)malononitrile (3d)

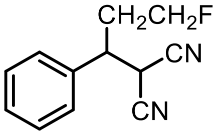

General procedure A was followed to obtain **3d** (50 mg, 0.25 mmol, 50%) as a colourless oil. **^{1}H NMR** (400 MHz, $CDCl_3$) δ 7.49 – 7.30 (m, 5H), 4.54 (ddt, J = 46.3, 9.2, 4.5 Hz, 1H), 4.28 (dtd, J = 47.4, 9.6, 3.4 Hz, 1H), 4.04 (d, J = 6.2 Hz, 1H), 3.51 (dt, J = 10.8, 5.6 Hz, 1H), 2.57 – 2.42 (m, 1H), 2.36 – 2.17 (m, 1H); **^{13}C NMR** (101 MHz, $CDCl_3$) δ 135.6, 129.6, 129.4, 128.0, 111.8, 111.7, 80.7 (d, $J_{C\text{-}F}$ = 167.2 Hz), 43.0 (d, $J_{C\text{-}F}$ = 3.5 Hz), 33.0 (d, $J_{C\text{-}F}$ = 20.0 Hz), 30.0 (d, $J_{C\text{-}F}$ = 1.8 Hz); **^{19}F NMR** (377 MHz, $CDCl_3$) δ -221.4 (tdd, J = 46.8, 34.7, 17.0 Hz); **HRMS** (ESI-TOF) calculated for $C_{12}H_{10}FN_2$ $[M\text{-}H]^-$: 201.0834; found 201.0826; **IR** (neat) 2909, 2256, 1497, 1456, 1435, 1393, 1220, 1025, 897, 877, 761, 701, 619.

2-(4-fluoro-1-phenylbutyl)malononitrile (3e)

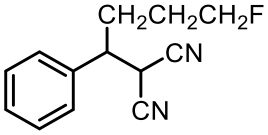

General procedure A was followed to obtain **3e** (77 mg, 0.36 mmol, 71%) as a pale yellow oil. **^{1}H NMR** (400 MHz, $CDCl_3$) δ 7.48 – 7.27 (m, 5H), 4.43 (dtd, J = 47.2, 5.8, 2.3 Hz, 2H), 3.92 (d, J = 6.3 Hz, 1H), 3.33 – 3.19 (m, 1H), 2.28 – 1.97 (m, 2H), 1.71 – 1.53 (m, 2H); **^{13}C NMR** (101 MHz, $CDCl_3$) δ 136.2, 129.5, 129.2, 127.9, 111.9, 111.9, 83.2 (d, J = 166.1 Hz), 46.3, 30.4, 28.3 (d, J = 4.2 Hz), 27.9 (d, J = 20.2 Hz); **^{19}F NMR** (377 MHz, $CDCl_3$) δ -219.20 (tt, J = 47.3, 26.4 Hz). **HRMS** (ESI-TOF) calculated for $C_{13}H_{12}FN_2$ $[M\text{-}H]^-$: 215.0990; found 215.0984; **IR** (neat) 2256, 1979, 1604, 1498, 1456, 1391, 1261, 1184, 1039, 1003, 914, 847, 801, 762, 737, 701, 618.

Benzyl 5-fluoro-2-methylpentanoate (3f)

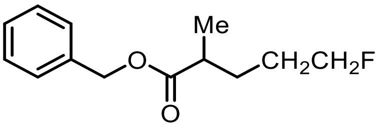

General procedure A was followed to obtain **3f** (45 mg, 0.20 mmol, 40%) as a colourless oil. **^{1}H NMR** (400 MHz, $CDCl_3$) δ 7.41 – 7.29 (m, 5H), 5.13 (s, 2H), 4.54 – 4.30 (m, 2H), 2.55 (h, J = 7.0 Hz, 1H), 1.88 – 1.51 (m, 4H), 1.21 (d, J = 7.1 Hz, 3H); **^{13}C NMR** (101 MHz, $CDCl_3$) δ 176.2, 136.2, 128.7, 128.3, 128.2, 83.8 (d, $J_{C\text{-}F}$ = 165.0 Hz), 66.3, 39.2, 29.5 (d, $J_{C\text{-}F}$ = 5.2 Hz), 28.2 (d, $J_{C\text{-}F}$ = 19.8 Hz), 17.2; **^{19}F NMR** (377 MHz, $CDCl_3$) δ -218.7 (tt, J = 47.4, 24.7 Hz). **HRMS** (ESI-TOF) calculated for $C_{13}H_{17}FNaO_2$ $[M+Na]^+$: 247.1105; found 247.1107; **IR** (neat) 2968, 1731, 1498, 1456, 1385, 1351, 1212, 1166, 1138, 1079, 1055, 1029, 997, 895, 842, 751, 697.

Benzyl 6-fluoro-2-methylhexanoate (3g)

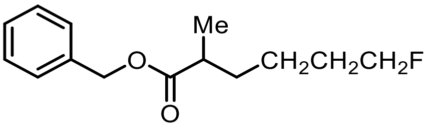

General procedure A was followed to obtain **3g** (49 mg, 0.21 mmol, 41%) as a colourless oil. **^{1}H NMR** (400 MHz, $CDCl_3$) δ 7.41 – 7.29 (m, 5H), 5.13 (s, 2H), 4.40 (dt, J = 47.3, 6.1 Hz, 2H), 2.51 (h, J = 7.0 Hz, 1H), 1.80 – 1.58 (m, 3H), 1.54 – 1.31 (m, 3H), 1.19 (d, J = 7.0 Hz, 3H); **^{13}C NMR** (101 MHz, $CDCl_3$) δ 176.5, 136.3, 128.7, 128.3, 83.9 (d, J = 164.7 Hz), 66.2, 39.6, 33.4, 30.4 (d, J = 19.6 Hz), 23.1 (d, J = 5.3 Hz), 17.2; **^{19}F NMR** (377 MHz, $CDCl_3$) δ -218.4 (tt, J = 47.4, 25.3 Hz); **HRMS** (ESI-TOF) calculated for $C_{14}H_{20}FO_2$ $[M+H]^+$: 239.1442; found 239.1443; **IR** (neat) 3035, 2941, 2160, 1978, 1732, 1607, 1498, 1456, 1385, 1354, 1246, 1161, 1138, 1081, 1066, 1041, 1029, 1003, 931, 826, 737, 697.

((4-fluorobutyl)sulfonyl)benzene (3h)

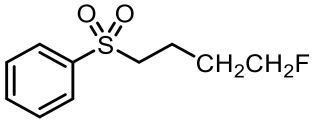

General procedure A was followed to obtain **3h** (45 mg, 0.20 mmol, 38%) as a colourless oil. ^{1}**H NMR** (400 MHz, $CDCl_3$) δ 7.93 – 7.88 (m, 2H), 7.69 – 7.63 (m, 1H), 7.61 – 7.54 (m, 2H), 4.42 (dt, J = 47.4, 5.5 Hz, 2H), 3.19 – 3.10 (m, 2H), 1.94 – 1.69 (m, 4H); ^{13}**C NMR** (101 MHz, $CDCl_3$) δ 139.1, 133.9, 129.5, 128.2, 83.3 (d, $J_{C\text{-}F}$ = 166.0 Hz), 55.9, 29.1 (d, $J_{C\text{-}F}$ = 19.9 Hz), 19.4 (d, $J_{C\text{-}F}$ = 4.6 Hz); ^{19}**F NMR** (377 MHz, $CDCl_3$) δ -219.5 (tt, J = 47.0, 26.3 Hz); **HRMS** (ESI-TOF) calculated for $C_{10}H_{13}FNaO_2{}^{32}S$ $[M+Na]^+$: 239.0512; found 239.0519; **IR** (neat) 2969, 1447, 1405, 1297, 1222, 1141, 1086, 1043, 1023, 927, 813, 750, 729, 689.

((5-Fluoropentyl)sulfonyl)benzene (3i)

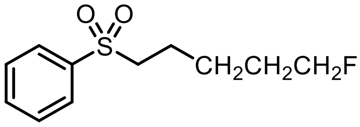

General procedure A was followed to obtain **3i** (51 mg, 0.22 mmol, 44%) as a pale yellow oil. ^{1}**H NMR** (400 MHz, $CDCl_3$) δ 7.93 – 7.87 (m, 2H), 7.69 – 7.62 (m, 1H), 7.61 – 7.52 (m, 2H), 4.39 (dt, J = 47.2, 5.9 Hz, 2H), 3.15 – 3.04 (m, 2H), 1.84 – 1.56 (m, 4H), 1.55 – 1.42 (m, 2H); ^{13}**C NMR** (101 MHz, $CDCl_3$) δ 139.2, 133.8, 129.4, 128.1, 83.5 (d, $J_{C\text{-}F}$ = 165.1 Hz), 56.2, 29.9 (d, $J_{C\text{-}F}$ = 19.9 Hz), 24.3 (d, $J_{C\text{-}F}$ = 5.0 Hz), 22.5; ^{19}**F NMR** (377 MHz, $CDCl_3$) δ -219.0 (tt, J = 47.1, 25.8 Hz); **HRMS** (ESI-TOF) calculated for $C_{11}H_{16}FO_2{}^{32}S$ $[M+H]^+$: 231.0851; found 231.0850; **IR** (neat) 2948, 2160, 1977, 1586, 1479, 1447, 1405, 1304, 1214, 1142, 1086, 1055, 1036, 999, 975, 950, 888, 854, 792, 746, 729, 689.

16.3. Hydrohalomethylation of electron-deficient alkenes

1-benzyl-3-(iodomethyl)pyrrolidine-2,5-dione (4a)

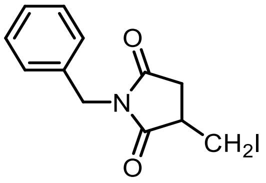

This product was purified by PREP TLC (Pentane/Et_2O, 70/30) due to instability on silica gel column chromatography. **General procedure A** was followed to obtain **4a** (102 mg, 0.27 mmol, 62%) as a waxy solid. **^{1}H NMR** (400 MHz, $CDCl_3$) δ 7.35 – 7.17 (m, 5H), 4.66 – 4.54 (m, 2H), 3.50 (dd, J = 10.3, 5.6 Hz, 1H), 3.32 (dd, J = 10.3, 3.7 Hz, 1H), 3.04 (dtd, J = 8.9, 5.2, 3.6 Hz, 1H), 2.82 (dd, J = 18.4, 8.9 Hz, 1H), 2.57 (dd, J = 18.4, 4.8 Hz, 1H); **^{13}C NMR** (101 MHz, $CDCl_3$) δ 177.0, 175.5, 135.3, 128.9, 128.7, 128.1, 42.9, 41.1, 35.7, 4.9; **HRMS** (ESI-TOF) calculated for $C_{12}H_{13}INO_2$ $[M+H]^+$: 329.9985, found 329.9985; **IR** (neat) 2922, 1775, 1697, 1605, 1586, 1496, 1455, 1429, 1396, 1342, 1312, 1290, 1243, 1208, 1167, 1080, 1029, 977, 929, 870, 838, 758, 707, 695, 634.

1-benzyl-3-(bromomethyl)pyrrolidine-2,5-dione (4b)

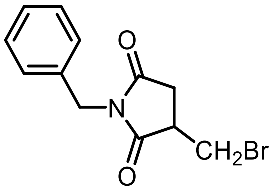

This product was purified by PREP TLC (Pentane/Et_2O, 70/30). **From CH_2Br_2: General procedure A** was followed to obtain **4b** (73 mg, 0.26 mmol, 52%) as a waxy solid. **From CH_2BrI: General procedure A** was followed to obtain **4b** (87 mg, 0.31 mmol, 62%) as a waxy solid. **^{1}H NMR** (400 MHz, $CDCl_3$) δ 7.35 – 7.16 (m, 5H), 4.60 (dd, J = 14.2 Hz, 2H), 3.77 (dd, J = 10.5, 4.9 Hz, 1H), 3.53 (dd, J = 10.5, 3.5 Hz, 1H), 3.20 (dtd, J = 8.6, 5.0, 3.4 Hz, 1H), 2.79 (dd, J = 18.3, 8.9 Hz, 1H), 2.68 (dd, J = 18.3, 5.1 Hz, 1H); **^{13}C NMR** (101 MHz, $CDCl_3$) δ 176.4, 175.1, 135.5, 128.8, 128.7, 128.1, 42.8, 41.3, 33.3, 31.8; **HRMS** (ESI-TOF) calculated for $C_{12}H_{13}{}^{79}BrNO_2$ $[M+H]^+$: 282.0124, found 282.0104; **IR** (neat) 2917, 2849, 1777, 1698, 1586, 1497, 1455, 1430, 1397, 1342, 1312, 1292, 1254, 1235, 1167, 1081, 1029, 984, 933, 869, 822, 761, 708, 695, 634.

1-benzyl-3-(chloromethyl)pyrrolidine-2,5-dione (4c)

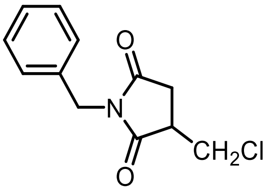

This product was purified by PREP TLC (Pentane/Et_2O, 70/30) due to instability on silica gel column chromatography. **General procedure A** was followed to obtain **4c** (64 mg, 0.27 mmol, 54%) as a waxy solid. **^{1}H NMR** (400 MHz, $CDCl_3$) *δ* 7.33 – 7.17 (m, 5H), 4.61 (dd, *J* = 14.8, 13.4 Hz, 2H), 3.95 (dd, *J* = 11.3, 4.6 Hz, 1H), 3.68 (dd, *J* = 11.3, 3.4 Hz, 1H), 3.16 (dtd, *J* = 8.6, 4.8, 3.4 Hz, 1H), 2.80 (dd, *J* = 18.3, 8.7 Hz, 1H), 2.72 (dd, *J* = 18.3, 5.3 Hz, 1H); **^{13}C NMR** (101 MHz, $CDCl_3$) *δ* 176.3, 175.3, 135.5, 128.7, 128.7, 128.1, 43.4, 42.8, 41.8, 32.0; **HRMS** (ESI-TOF) calculated for $C_{12}H_{13}{}^{35}ClNO_2$ $[M+H]^+$: 238.0629, found 238.0630; **IR** (neat) 2918, 1777, 1698, 1586, 1497, 1455, 1432, 1398, 1341, 1313, 1244, 1168, 1082, 1029, 991, 940, 886, 790, 764, 709, 696, 673, 633, 608.

1-benzyl-3-(bromofluoromethyl)pyrrolidine-2,5-dione (4d)

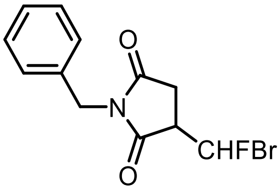

This product was purified by PREP TLC (Pentane/Et_2O, 70/30). **General procedure A** was followed to obtain **4d** (66 mg, 0.26 mmol, 51%, d.r: 1/1) as a colourless oil. The compound coeluted with traces of a vinyl fluoride side product, likely stemming from HBr elimination of the desired product. **^{1}H NMR** (400 MHz, $CDCl_3$) *δ* 6.92 – 6.88 (m, 5H), 6.93 – 6.87 (m, 0.5H), 6.81 – 6.75 (m, 0.5H), 4.64 – 4.52 (m, 2H), 3.59 – 3.49 (m, 0.5H), 3.35 (dddd, *J* = 31.7, 9.3, 5.6, 1.8 Hz, 0.5H), 3.07 – 2.70 (m, 2H); **^{13}C NMR** (101 MHz, $CDCl_3$) *δ* 174.5, 174.1, 173.2, 172.9 (d, $J_{C\text{-}F}$ = 14.4 Hz), 135.2, 135.1, 128.9, 128.8, 128.7, 128.7, 128.2, 93.2 (d, $J_{C\text{-}F}$ = 256.2 Hz), 90.1 (d, $J_{C\text{-}F}$ = 254.1 Hz), 49.8 (d, $J_{C\text{-}F}$ = 21.4 Hz), 47.5 (d, $J_{C\text{-}F}$ = 22.6 Hz), 42.9 (d, $J_{C\text{-}F}$ = 6.9 Hz), 30.7, 29.8; **^{19}F NMR** (377 MHz, $CDCl_3$) *δ* -139.5 (dd, *J* = 48.0, 7.1 Hz), -147.0 (dd, *J* = 49.3, 31.8 Hz); The compound did not ionize; **IR** (neat) 1180, 1705, 1497, 1432, 1399, 1345, 1249, 1170, 1119, 1083, 1028, 951; **m.p.**: 64 – 66 °C. 5% of elimination product (1-benzyl-3-(fluoromethylene)pyrrolidine-2,5-dione) was observed by quantitative ^{19}F NMR.

4-chloro-*N*-phenylbutanamide (4e)

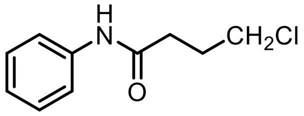

General procedure A was followed to obtain **4e** (32 mg, 0.16 mmol, 32%) as a white solid. **^{1}H NMR** (400 MHz, $CDCl_3$) δ 7.50 (d, J = 7.8 Hz, 1H), 7.32 (t, J = 7.9 Hz, 1H), 3.65 (t, J = 6.1 Hz, 1H), 2.55 (t, J = 7.1 Hz, 1H), 2.20 (p, J = 6.6 Hz, 1H); **^{13}C NMR** (101 MHz, $CDCl_3$) δ 170.1, 137.8, 129.2, 124.6, 120.0, 44.6, 34.3, 28.1. All data were in accordance with the literature.[14]

Benzyl 4-chloro-2-methylbutanoate (4f)

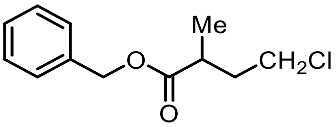

General procedure A was followed to obtain **4f** (50 mg, 0.22 mmol, 44%) as a colourless oil. **^{1}H NMR** (400 MHz, $CDCl_3$) δ 7.43 – 7.29 (m, 5H), 5.14 (d, J = 1.0 Hz, 2H), 3.55 (td, J = 6.6, 1.1 Hz, 2H), 2.79 (h, J = 7.1 Hz, 1H), 2.27 – 2.15 (m, 1H), 1.92 – 1.80 (m, 1H), 1.23 (d, J = 7.1 Hz, 3H); **^{13}C NMR** (101 MHz, $CDCl_3$) δ 175.6, 136.1, 128.7, 128.4, 128.2, 66.5, 42.6, 36.9, 36.1, 16.9. The compound did not ionize. **IR** (neat) 2973, 2161, 1731, 1498, 1455, 1384, 1355, 1284, 1236, 1155, 1123, 1091, 1058, 1029, 1004, 966, 890, 839, 789, 749, 697, 659.

Benzyl 2-(2,3-dichloro-4-(4-chloro-2-ethylbutanoyl)phenoxy)acetate (4g)

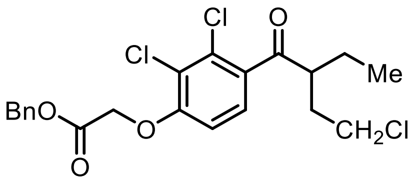

The reaction was performed on 100 mg of ethacrynic acid benzyl ester. **General procedure A** was followed to obtain **4g** (56 mg, 0.13 mmol, 50%) as a colourless oil. **^{1}H NMR** (400 MHz, $CDCl_3$) δ 7.41 – 7.31 (m, 6H), 6.73 (d, *J* = 8.7 Hz, 1H), 5.25 (s, 2H), 4.80 (s, 2H), 3.70 – 3.62 (m, 1H), 3.59 – 3.45 (m, 2H), 2.33 (ddt, *J* = 14.2, 8.6, 5.6 Hz, 1H), 1.90 (dddd, *J* = 14.4, 8.4, 6.1, 4.8 Hz, 1H), 1.81 – 1.66 (m, 1H), 1.59 – 1.45 (m, 1H), 0.90 (t, *J* = 7.5 Hz, 3H); **^{13}C NMR** (101 MHz, $CDCl_3$) *δ* 203.8, 167.5, 156.3, 134.9, 134.4, 132.0, 128.9, 128.8, 128.7, 127.4, 124.3, 110.8, 67.5, 66.3, 48.8, 43.3, 32.6, 24.6, 11.3; **HRMS** (ESI-TOF) calculated for $C_{21}H_{22}{}^{35}Cl_3O_4$ $[M+H]^+$: 443.0578, found 443.0579; **IR** (neat) 1757, 1693, 1583, 1466, 1386, 1191, 1077, 811, 751, 697, 666.

15.4. Hydromethylation of electron-deficient alkenes

1-benzyl-3-methylpyrrolidine-2,5-dione (5a)

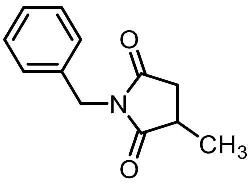

General procedure C was followed to obtain **5a** (94 mg, 0.46 mmol, 93%) as a colourless oil. **^{1}H NMR** (400 MHz, $CDCl_3$) δ 7.26 – 7.22 (m, 1H), 7.22 – 7.12 (m, 1H), 4.52 (d, J = 2.1 Hz, 1H), 2.85 – 2.67 (m, 1H), 2.19 (dd, J = 21.7, 13.6 Hz, 1H), 1.20 (d, J = 7.2 Hz, 1H); **^{13}C NMR** (101 MHz, $CDCl_3$) δ 180.3, 176.1, 136.0, 128.8, 128.7, 128.0, 42.5, 36.5, 34.8, 16.8; **MS** (ESI) m/z = 204.0 $[M+H]^+$. All data were in accordance with the literature.[8]

2-(1-phenylethyl)malononitrile (5b)

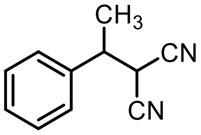

General procedure C was followed to obtain **5b** (53 mg, 0.31 mmol, 62%) as a colourless oil. **^{1}H NMR** (400 MHz, $CDCl_3$) δ 7.48 – 7.27 (m, 5H), 3.85 (d, *J* = 6.1 Hz, 1H), 3.46 (p, *J* = 6.9 Hz, 1H), 1.66 (d, *J* = 7.0 Hz, 3H); **^{13}C NMR** (101 MHz, $CDCl_3$) δ 138.3, 129.4, 129.0, 127.4, 112.1, 111.8, 41.4, 31.4, 17.9. All data were in accordance with the literature.[15]

Benzyl butyrate (5c)

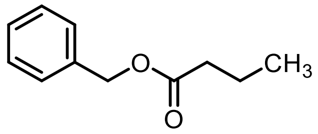

General procedure C was followed to obtain **5c** (60 mg, 0.34 mmol, 67%) as a colourless oil. **^{1}H NMR** (400 MHz, $CDCl_3$) δ 7.42 – 7.28 (m, 5H), 5.13 (s, 2H), 2.35 (t, J = 7.4 Hz, 2H), 1.69 (h, J = 7.4 Hz, 2H), 0.96 (t, J = 7.4 Hz, 3H); **^{13}C NMR** (101 MHz, $CDCl_3$) δ 173.7, 136.3, 131.2, 128.7, 128.3, 66.2, 36.4, 29.9, 18.6, 13.8. All data were in accordance with the literature.[16]

(Propylsulfonyl)benzene (5d)

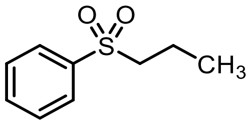

General procedure C was followed to obtain **5d** (53 mg, 0.31 mmol, 55%) as a colourless oil. **^{1}H NMR** (400 MHz, $CDCl_3$) δ 7.95 – 7.87 (m, 2H), 7.69 – 7.62 (m, 1H), 7.60 – 7.54 (m, 2H), 3.10 – 3.03 (m, 2H), 1.81 – 1.68 (m, 2H), 0.99 (t, J = 7.5 Hz, 3H); **^{13}C NMR** (101 MHz, $CDCl_3$) δ 139.4, 133.7, 129.4, 128.2, 58.1, 16.7, 13.1. All data were in accordance with the literature.[17]

Benzyl 2-methylbutanoate (5e)

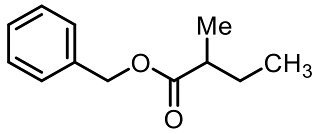

General procedure C was followed to obtain **5e** (52 mg, 0.27 mmol, 54%) as a colourless oil. ^{1}**H NMR** (400 MHz, $CDCl_3$) *δ* 7.42 – 7.28 (m, 5H), 5.13 (s, 2H), 2.44 (h, *J* = 7.0 Hz, 1H), 1.78 – 1.63 (m, 1H), 1.57 – 1.43 (m, 1H), 1.18 (d, *J* = 7.0 Hz, 3H), 0.91 (t, *J* = 7.5 Hz, 3H); ^{13}**C NMR** (101 MHz, $CDCl_3$) δ 176.7, 136.4, 128.6, 128.2, 128.2, 66.1, 41.2, 26.9, 16.7, 11.7. All data were in accordance with the literature.[18]

***N*-benzyl-2-methylcyclobutane-1-carboxamide (5f)**

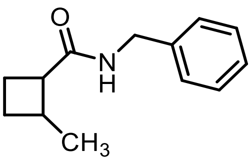

General procedure C was followed to obtain **5f** (16 mg, 0.08 mmol, 16%, d.r.: 60/40) as a white solid (mixture of both diastereoisomers). ^{1}**H NMR** (400 MHz, $CDCl_3$) *δ* 7.38 – 7.25 (m, 5H), 5.60 (s, 1H), 4.49 – 4.42 (m, 2H), 3.07 (q, *J* = 8.2 Hz, 0.6H), 2.77 – 2.67 (m, 0.6H), 2.65 – 2.55 (m, 0.4H), 2.54 – 2.46 (m, 0.4H), 2.43 – 2.30 (m, 0.6H), 2.19 – 2.07 (m, 1H), 2.06 – 1.94 (m, 1.4H), 1.68 – 1.58 (m, 0.6H), 1.57 – 1.45 (m, 0.4H), 1.12 (d, *J* = 6.6 Hz, 1.2H), 1.08 (d, *J* = 7.1 Hz, 1.8H); ^{13}**C NMR** (101 MHz, $CDCl_3$) *δ* 174.3, 173.1, 138.7, 138.6, 128.9, 128.8, 128.1, 127.9, 127.6, 48.1, 43.7, 43.2, 35.1, 32.9, 29.8, 26.4, 26.3, 21.6, 21.5, 20.5, 16.7; **HRMS** (ESI-TOF) calculated for $C_{13}H_{18}NO$ $[M+H]^+$: 204.1383, found 204.1385; **IR** (neat) 3289, 3065, 2951, 2865, 1643, 1540, 1497, 1454, 1356, 1241, 1029, 845, 730, 697; **m.p.**: 41 – 42 °C.

***N*-benzyl-2-(methyl-*d*$_3$)cyclobutane-1-carboxamide (5g)**

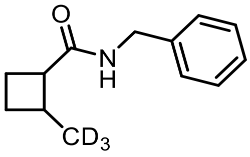

General procedure C was followed to obtain **5g** (20 mg, 0.10 mmol, 19%, d.r.: 60/40) as a white solid (mixture of both diastereoisomers). **^{1}H NMR** (400 MHz, $CDCl_3$) δ 7.37 – 7.24 (m, 5H), 5.63 (s, 1H), 4.45 (dd, J = 8.1, 5.5 Hz, 2H), 3.07 (q, J = 8.1 Hz, 0.6H), 2.73 – 2.65 (m, 0.6H), 2.64 – 2.55 (m, 0.4H), 2.54 – 2.47 (m, 0.4H), 2.44 – 2.31 (m, 0.6H), 2.19 – 2.06 (m, 1.0H), 2.05 – 1.94 (m, 1.4H), 1.67 – 1.57 (m, 0.6H), 1.56 – 1.46 (m, 0.4H); **^{13}C NMR** (101 MHz, $CDCl_3$) δ 174.3, 173.1, 138.7, 138.6, 128.8, 128.8, 128.1, 127.9, 127.6, 48.0, 43.6, 43.6, 43.1, 34.9, 32.7, 29.8, 26.3, 26.1, 21.6, 20.5, 16.3 – 15.5 (m); **HRMS** (ESI-TOF) calculated for $C_{13}H_{15}D_3NO$ $[M+H]^+$: 207.1571, found 207.1572; **IR** (neat) 2936, 2863, 2360, 2212, 1636, 1538, 1498, 1454, 1380, 1354, 1325, 1255, 1238, 1226, 1158, 1130, 1080, 1049, 1029, 952, 817, 744, 695; **m.p.**: 41 – 42 °C.

Benzyl 2-(2,3-dichloro-4-(2-ethylbutanoyl)phenoxy)acetate (5h)

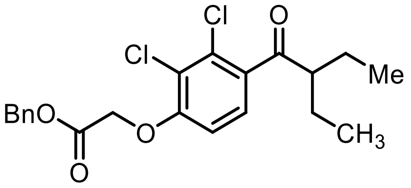

The reaction was performed on 100 mg of ethacrynic acid benzyl ester. **General procedure C** was followed to obtain **5h** (47 mg, 0.11 mmol, 45%) as a colourless oil. **^{1}H NMR** (400 MHz, $CDCl_3$) δ 7.41 – 7.30 (m, 5H), 7.23 (d, J = 8.6 Hz, 1H), 6.72 (d, J = 8.6 Hz, 1H), 5.24 (s, 2H), 4.79 (s, 2H), 3.10 (ddd, J = 12.8, 7.1, 5.8 Hz, 1H), 1.77 (dt, J = 13.8, 7.3 Hz, 2H), 1.59 – 1.44 (m, 2H), 0.91 (t, J = 7.5 Hz, 6H); **^{13}C NMR** (101 MHz, $CDCl_3$) δ 205.4, 167.6, 156.0, 135.4, 135.0, 131.7, 128.9, 128.8, 128.7, 127.1, 124.0, 110.8, 67.5, 66.3, 53.5, 23.7, 11.7; **HRMS** (ESI-TOF) calculated for $C_{21}H_{23}{}^{35}Cl_2O_4$ $[M+H]^+$: 409.0968, found 409.0968; **IR** (neat) 2964, 2933,

2876, 2159, 1758, 1693, 1583, 1559, 1498, 1459, 1383, 1303, 1264, 1192, 1123, 1076, 1028, 894, 837, 810, 739, 697, 644.

Benzyl 2-(2,3-dichloro-4-(2-ethylbutanoyl-4,4,4-*d*3)phenoxy)acetate (5i)

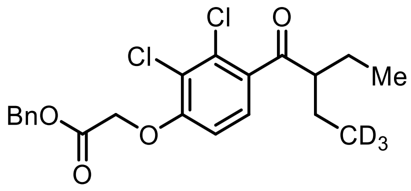

The reaction was performed on 100 mg of ethacrynic acid benzyl ester. **General procedure C** was followed to obtain **5i** (62 mg, 0.15 mmol, 59%) as a colourless oil. **^{1}H NMR** (500 MHz, $CDCl_3$) *δ* 7.40 – 7.30 (m, 5H), 7.23 (d, *J* = 8.6 Hz, 1H), 6.72 (d, *J* = 8.6 Hz, 1H), 5.24 (s, 2H), 4.79 (s, 2H), 3.09 (ddd, *J* = 12.8, 7.1, 5.8 Hz, 1H), 1.82 – 1.70 (m, 2H), 1.57 – 1.45 (m, 2H), 0.91 (t, *J* = 7.4 Hz, 3H); **^{13}C NMR** (126 MHz, $CDCl_3$) *δ* 205.4, 167.6, 156.0, 135.3, 134.9, 131.7, 128.9, 128.8, 128.7, 127.1, 124.0, 110.9 – 110.7 (m), 67.7 – 67.3 (m), 66.6 – 66.1 (m), 53.6 – 53.3 (m), 23.9 – 23.1 (m), 11.9 – 11.6 (m), 11.3 – 10.3 (m); **HRMS** (ESI-TOF) calculated for $C_{21}H_{20}D_3{}^{35}Cl_2O_4$ $[M+H]^+$: 412.1156, found 412.1156; **IR (neat)** 2931, 2219, 1758, 1692, 1582, 1559, 1498, 1465, 1383, 1302, 1259, 1190, 1118, 1076, 1025, 887, 809, 738, 697, 661, 639.

Benzyl 2-(2,3-dichloro-4-(2-ethylbutanoyl-4-^{13}C)phenoxy)acetate (5j)

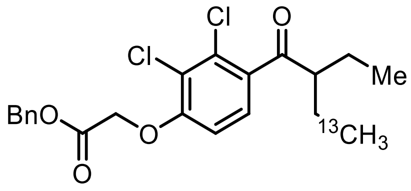

The reaction was performed on 100 mg of ethacrynic acid benzyl ester. **General procedure C** was followed to obtain **5j** (46 mg, 0.11 mmol, 44%) as a colourless oil. **^{1}H NMR** (500 MHz, $CDCl_3$) *δ* 7.40 – 7.31 (m, 5H), 7.23 (d, *J* = 8.6 Hz, 1H), 6.72 (d, *J* = 8.7 Hz, 1H), 5.25 (s, 2H), 4.79 (s, 2H), 3.13 – 3.06 (m, 1H), 1.83 – 1.70 (m, 2H), 1.59 – 1.46 (m, 2H), 0.91 (t, *J* = 7.4 Hz, 3H), 0.91 (dt, *J* = 125.6, 7.4 Hz, 3H); **^{13}C NMR** (126 MHz, $CDCl_3$) *δ* 205.4 – 205.4 (m), 167.7, 156.0, 135.4, 135.0, 131.7, 128.9, 128.8, 128.7, 127.2, 124.0, 110.8, 67.5, 66.3, 53.5, 29.8, 24.0 – 23.5 (m), 11.7 (br); **HRMS** (ESI-TOF) calculated for $C_{20}{}^{13}CH_{23}{}^{35}Cl_2O_4$ $[M+H]^+$: 410.1001,

found 410.1001; **IR (neat)** 2934, 2872, 2362, 2342, 2158, 2020, 1976, 1760, 1695, 1584, 1559, 1498, 1466, 1385, 1303, 1263, 1194, 1121, 1078, 1025, 893, 810, 753, 698, 668, 652.

Benzyl 2-(2,3-dichloro-4-(2-ethylbutanoyl-4-*d*)phenoxy)acetate (5k)

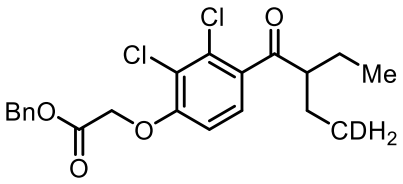

The reaction was performed on 100 mg of ethacrynic acid benzyl ester. **General procedure C** was followed to obtain **5k** (71 mg, 0.17 mmol, 68%) as a pale yellow oil. **^{1}H NMR** (400 MHz, $CDCl_3$) δ 7.41 – 7.30 (m, 5H), 7.23 (d, J = 8.6 Hz, 1H), 6.72 (d, J = 8.6 Hz, 1H), 5.24 (s, 2H), 4.78 (s, 2H), 3.09 (tt, J = 7.1, 5.7 Hz, 1H), 1.82 – 1.69 (m, 2H), 1.59 – 1.45 (m, 2H), 0.95 – 0.85 (m, 5H); **^{13}C NMR** (101 MHz, $CDCl_3$) δ 205.3, 167.6, 155.9, 135.3, 134.9, 131.6, 128.8, 128.8, 128.6, 127.1, 123.9, 110.8, 67.5, 66.3, 53.5, 23.7, 23.6, 11.7, 11.6 – 11.2 (m); **HRMS** (ESI-TOF) calculated for $C_{21}H_{22}D^{35}Cl_2O_4$ [M+H]$^+$: 410.1015, found 410.1031; **IR** (neat) 2963, 2934, 2875, 2361, 2174, 1757, 1693, 1583, 1559, 1498, 1465, 1384, 1302, 1263, 1190, 1120, 1076, 1022, 962, 891, 810, 752, 697, 643.

Benzyl 2-(2,3-dichloro-4-(2-ethylbutanoyl-4,4-*d*$_2$)phenoxy)acetate (5l)

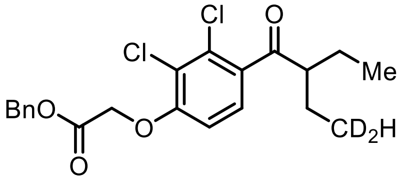

The reaction was performed on 100 mg of ethacrynic acid benzyl ester. **General procedure D** was followed to obtain **5l** (62 mg, 0.15 mmol, 59%) as a pale yellow oil. **^{1}H NMR** (400 MHz, $CDCl_3$) δ 7.41 – 7.30 (m, 5H), 7.22 (d, J = 8.6 Hz, 1H), 6.72 (d, J = 8.6 Hz, 1H), 5.24 (s, 2H), 4.79 (s, 2H), 3.14 – 3.04 (m, 1H), 1.84 – 1.69 (m, 2H), 1.58 – 1.45 (m, 2H), 0.97 – 0.83 (m, 4H); **^{13}C NMR** (101 MHz, $CDCl_3$) δ 205.3, 167.6, 155.9, 135.3, 134.9, 131.6, 128.8, 128.8, 128.6, 127.1, 123.9, 110.8, 67.5, 66.3, 53.4, 23.7, 23.5, 11.7, 11.1 (p, J = 19.2 Hz); **HRMS** (ESI-TOF) calculated for $C_{21}H_{21}D_2{}^{35}Cl_2O_4$ [M+H]$^+$: 411.1093, found 410.1094; **IR** (neat) 2935, 2875, 2216,

1757, 1693, 1583, 1559, 1498, 1465, 1384, 1302, 1263, 1190, 1119,1076, 1026, 994, 887, 810, 752, 697, 641.

enzyl 2-(2,3-dichloro-4-(2-ethylbutanoyl-4-^{13}C-4,4,4-*d*$_3$)phenoxy)acetate (5m)

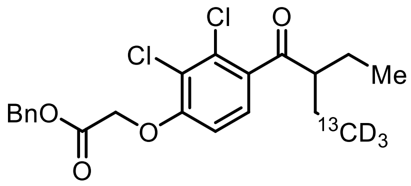

The reaction was performed on 100 mg of ethacrynic acid benzyl ester. **General procedure D** was followed to obtain **5m** (55 mg, 0.13 mmol, 52%) as a pale yellow oil. **^{1}H] NMR** (400 MHz, $CDCl_3$) δ 7.41 – 7.31 (m, 4H), 7.23 (d, *J* = 8.6 Hz, 1H), 6.72 (d, *J* = 8.6 Hz, 1H), 5.24 (s, 2H), 4.79 (s, 2H), 3.15 – 3.04 (m, 1H), 1.83 – 1.69 (m, 2H), 1.58 – 1.46 (m, 2H), 0.91 (t, *J* = 7.5 Hz, 3H); **^{13}C NMR** (101 MHz, $CDCl_3$) *δ* 205.4 – 205.3 (m), 167.6, 155.9, 135.3, 134.9, 131.7, 128.8, 128.8, 128.6, 127.1, 124.0, 110.8, 67.5, 66.3, 53.4, 23.9 – 23.1 (m), 23.7 – 23.2 (m), 11.7, 11.5 – 10.1 (m); **HRMS** (ESI-TOF) calculated for $C_{20}{}^{13}CH_{20}D_3{}^{35}Cl_2O_4$ $[M+H]^+$: 413.1190, found 413.1190; **IR** (neat) 2963, 2932, 2875, 2203, 2032, 1758, 1692, 1582, 1559, 1498, 1465, 1384, 1302, 1259, 1190, 1119, 1076, 1025, 887, 809, 753, 738, 697, 660, 638.

17. References

[1] X. J. Tang, W. R. Dolbier, *Angew. Chem. Int. Ed.* **2015**, *54*, 4246–4249.

[2] P. Rai, K. Maji, B. Maji, *Org. Lett.* **2019**, *21*, 3755–3759.

[3] J. Eriksson, O. Åberg, B. Långström, *Eur. J. Org. Chem.* **2007**, 455–461.

[4] O. A. Al-Fulaij, A. Z. A. Elassar, A. F. J. El-Asmy, *Appl. Polym. Sci.* **2015**, *132*, 1–14.

[5] T. J. Saiegh, H. Chedotal, C. Meyer, J. Cossy, *Org. Lett*. **2019**, *21*, 8364−8368.

[6] F. Wang, D. Fan, J. Qian, Z. Zhang, J. Zhu, J. Chen, *Medicinal Chemistry*. **2015**, *11*, 649−655.

[7] S. M. Hell, C. F. Meyer, G. Laudadio, A. Misale, M. C. Willis, T. Noël, A. A. Trabanco, V. Gouverneur, *J. Am. Chem. Soc.* **2020**, *142*, 720–725.

[8] M. D. Barker, R. A. Dixon, S. Jones, B. J. Marsh, *Chem. Commun.* **2008**, 2218–2220.

[9] E. Dardennes, S. Labano, N. S. Simpkins, C. Wilson, *Tetrahedron Lett.* **2007**, *48*, 6380–6383.

[10] Y. X. Li, W. Y. Li, Y. Y. Jiang, L. Yang, *Tetrahedron Lett.* **2018**, *59*, 2934–2937.

[11] P. Zhang, C. C. Le, D. W. C. Macmillan, *J. Am. Chem. Soc.* **2016**, *138*, 8084-8087.

[12] W. Wang, C. Ding, Y. Li, Z. Li, Y. Li, L. Peng, G. Yin, *Angew. Chem. Int. Ed.* **2019**, *58*, 4612 –4616.

[13] J. Guo, C. Kuang, J. Rong, L. Li, C. Ni, J. Hu, *Chem. Eur. J.* **2019**, *25*, 7259 –7264.

[14] E. Massolo, M. Pirola, A. Puglisi, S. Rossi, M. Benaglia, *RSC Adv.* **2020**, *10*, 4040-4044.

[15] G. Laudadio, Y. Deng, K. Van der Wal, D. Ravelli, M. Nuño, M. Fagnoni, D. Guthrie, Y. Sun, T. Noël, *Science* **2020**, *369*, 92–96.

[16] J. Dong, X. Wang, Z. Wang, H. Song, Y. Liu, Q. Wang, *Chem. Commun.*, **2019**, *55*, 11707-11710.

[17] Q. Zhu, D. G. Nocera, *J. Am. Chem. Soc.* **2020**, *142*, 17913−17918.

[18] J. Q. Li, X. Quan, P. G. Andersson, *Chem. Eur. J.* **2012**, *18*, 10609 – 10616.

18. Spectra

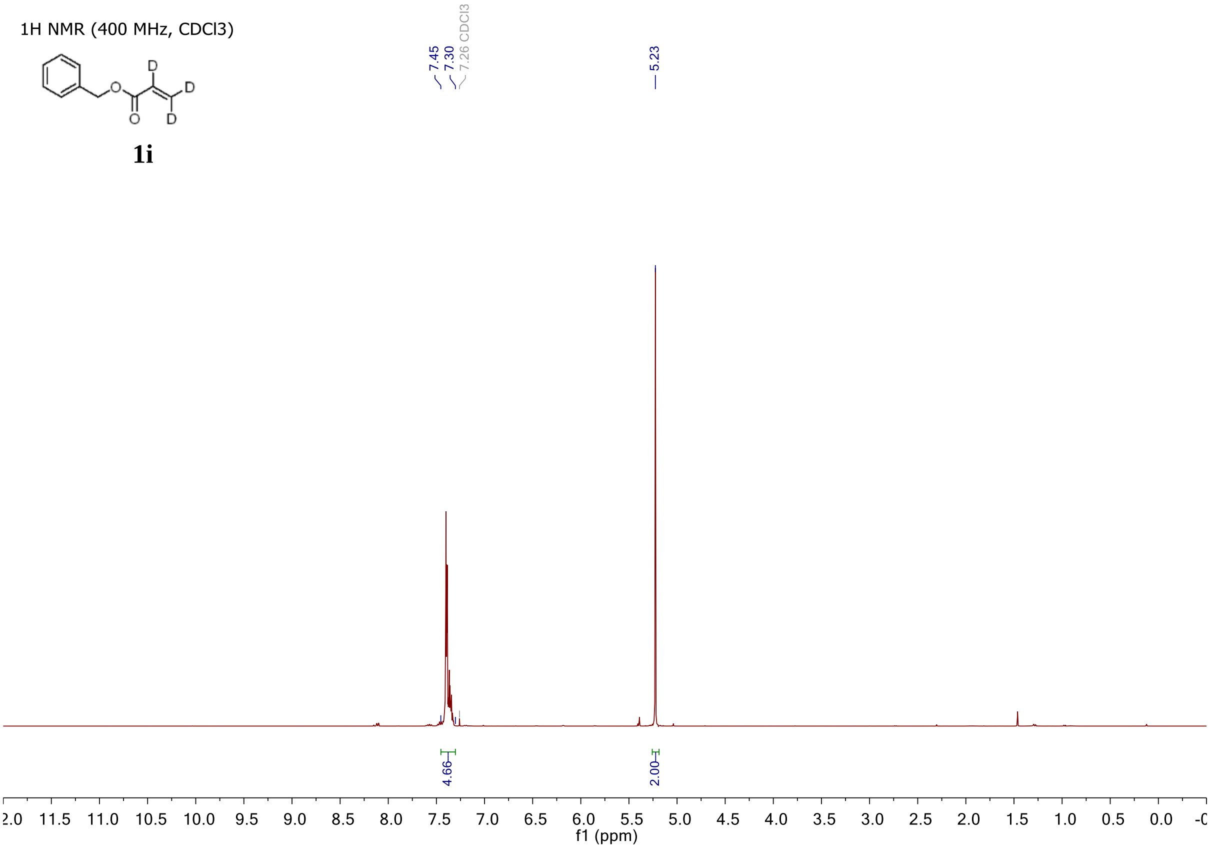

1H NMR (400 MHz, CDCl3)
1i
7.45
7.30
7.26 CDCl3
5.23
4.66
2.00
f1 (ppm)

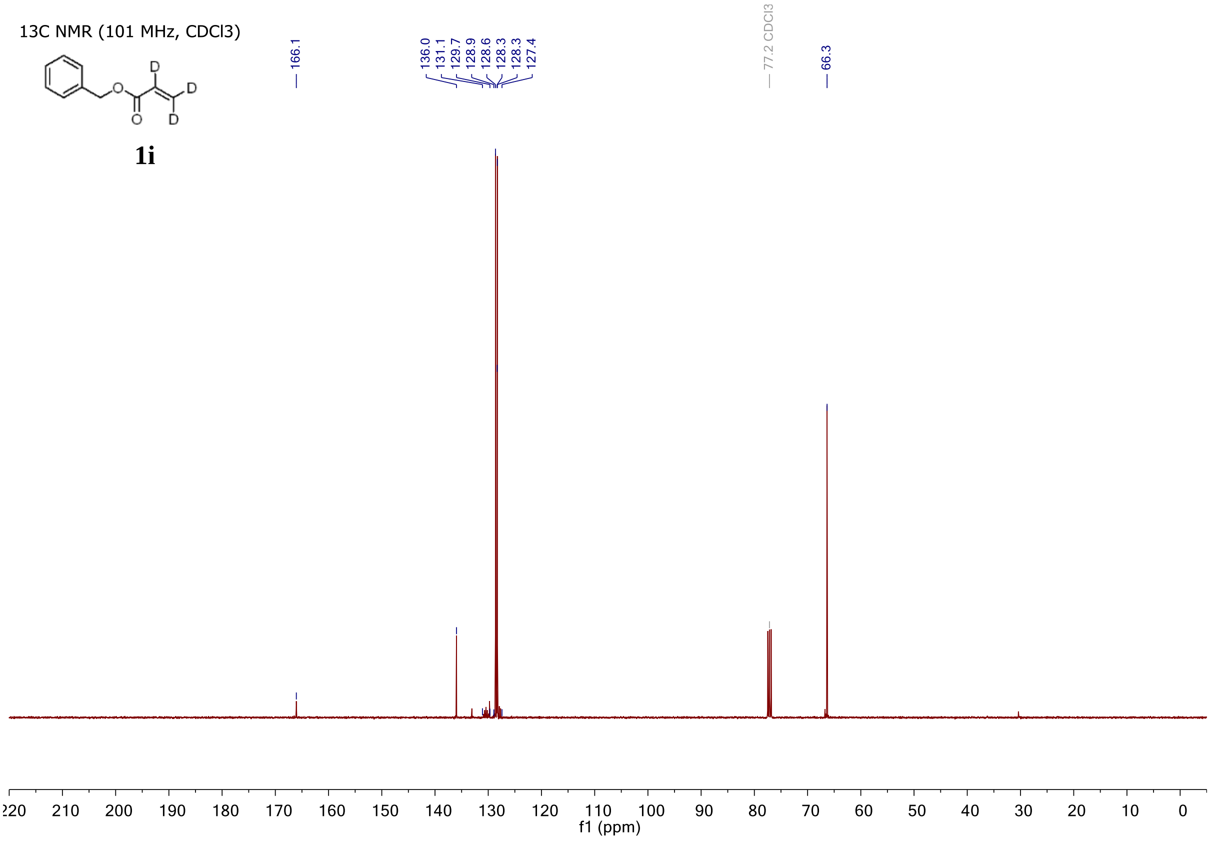

13C NMR (101 MHz, CDCl3)
1i
166.1
136.0
131.1
129.7
128.9
128.6
128.3
128.3
127.4
77.2 CDCl3
66.3
f1 (ppm)

1H NMR (400 MHz, CDCl3)

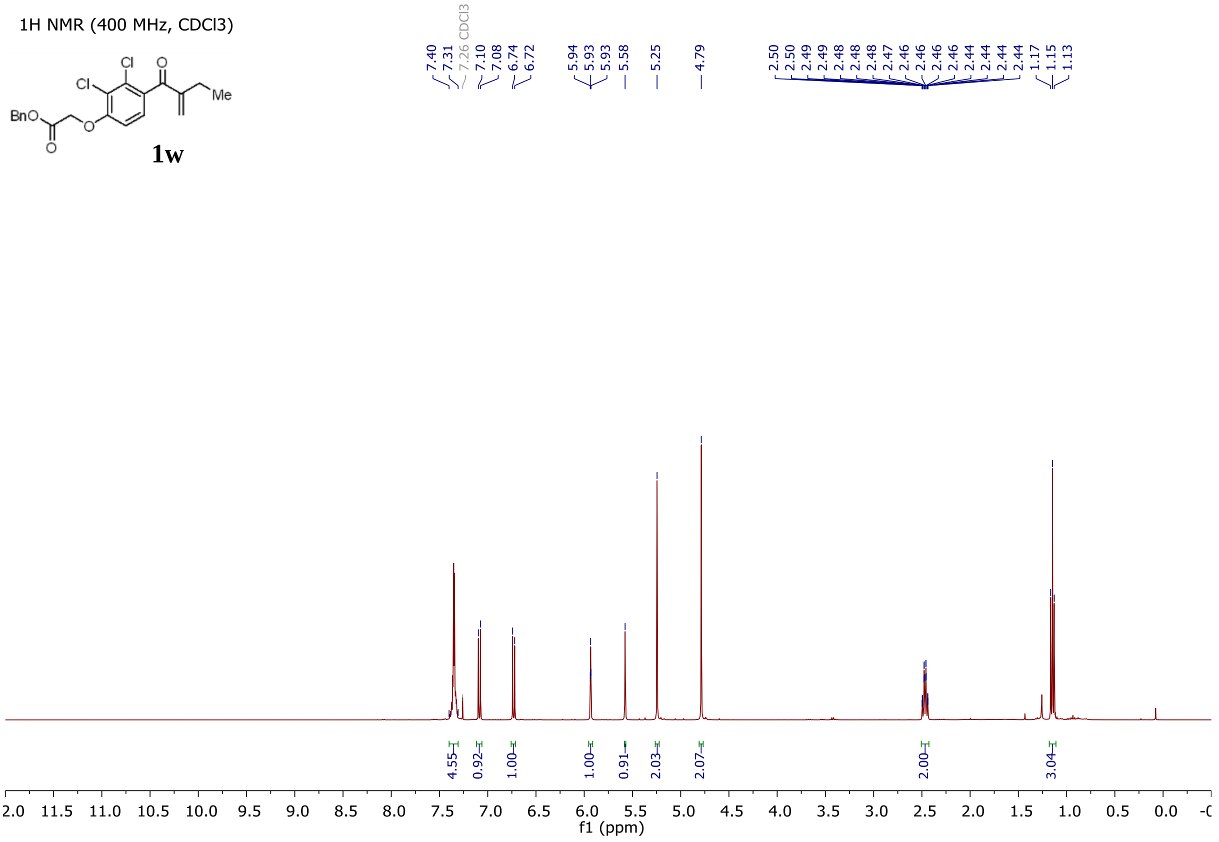

13C NMR (101 MHz, CDCl3)

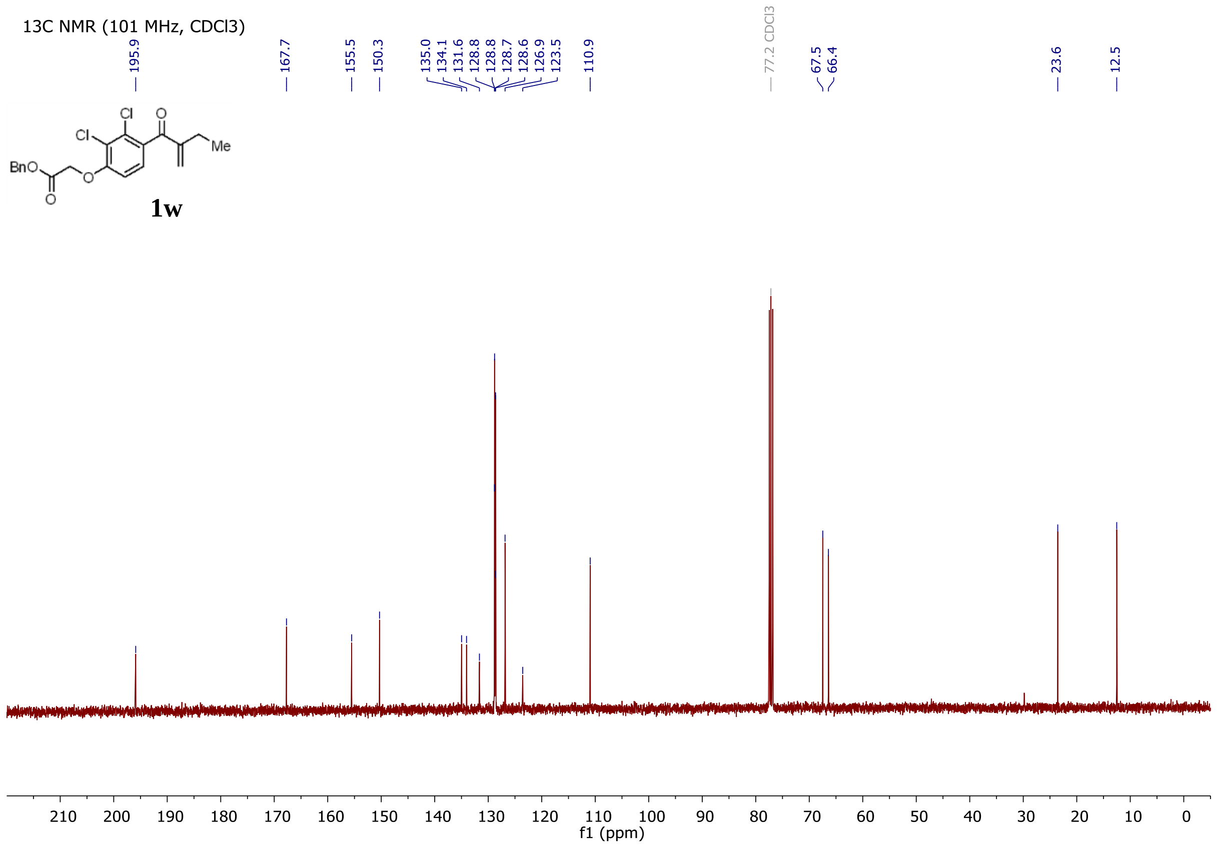

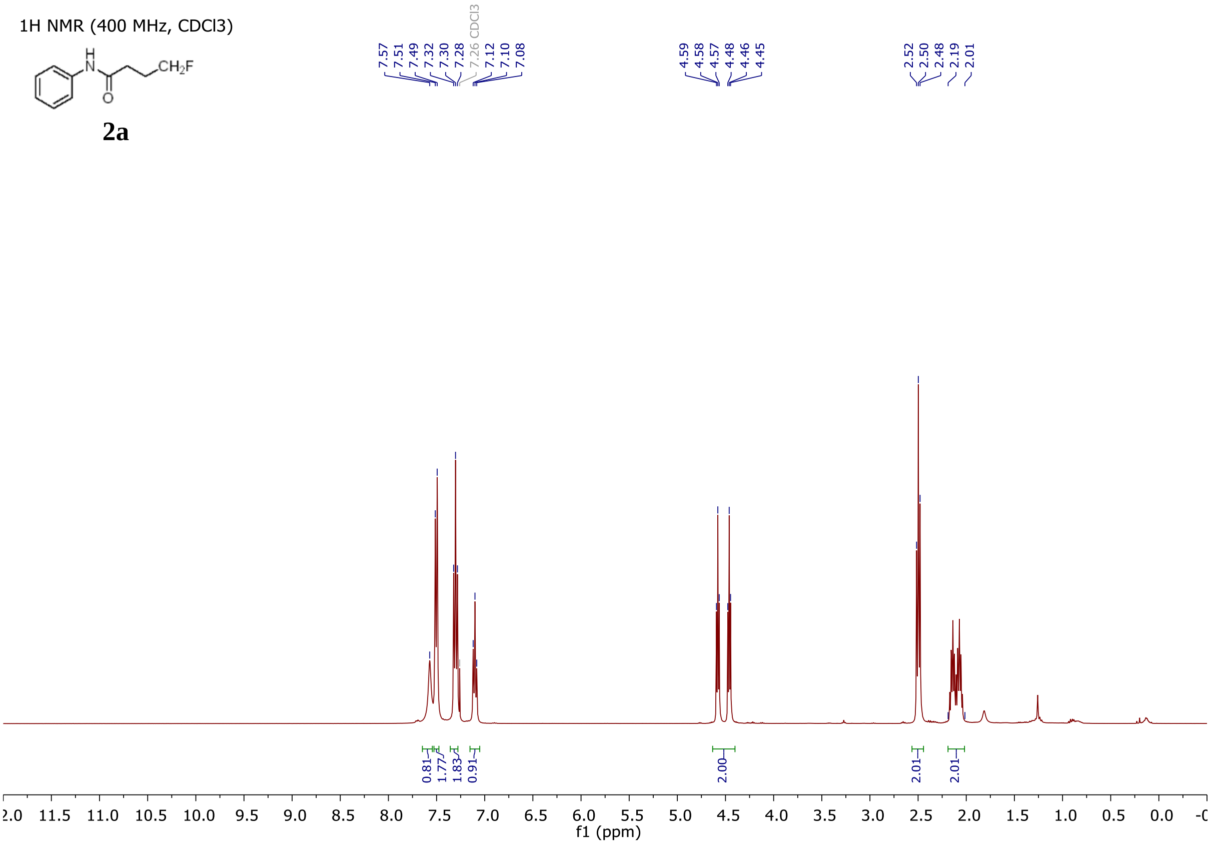
1H NMR (400 MHz, CDCl3)
CH2F
2a
7.57
7.51
7.49
7.32
7.30
7.28
7.26 CDCl3
7.12
7.10
7.08
4.59
4.58
4.57
4.48
4.46
4.45
2.52
2.50
2.48
2.19
2.01
0.81
1.77
1.83
0.91
2.00
2.01
2.01
f1 (ppm)

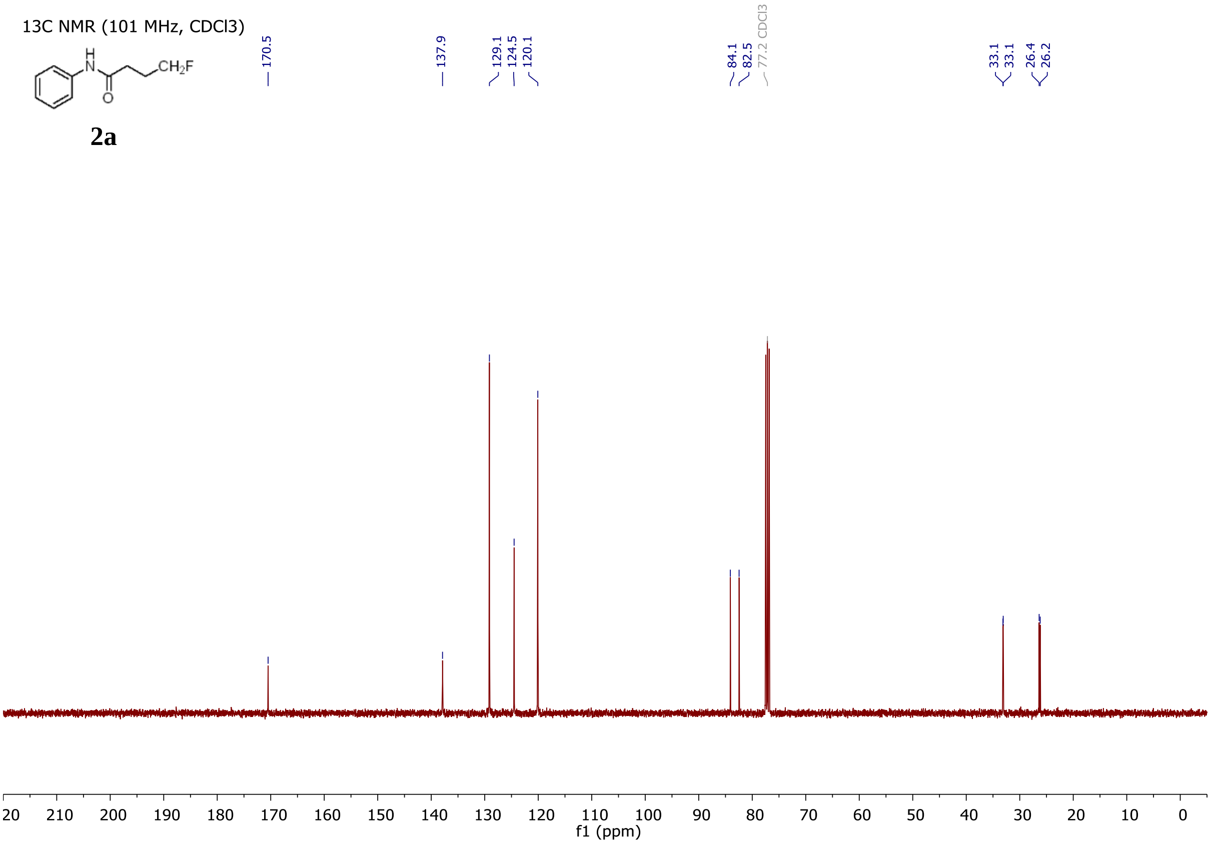
13C NMR (101 MHz, CDCl3)
CH2F
2a
170.5
137.9
129.1
124.5
120.1
84.1
82.5
77.2 CDCl3
33.1
33.1
26.4
26.2
f1 (ppm)

19F NMR (377 MHz, CDCl3)

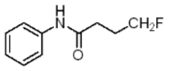

2a

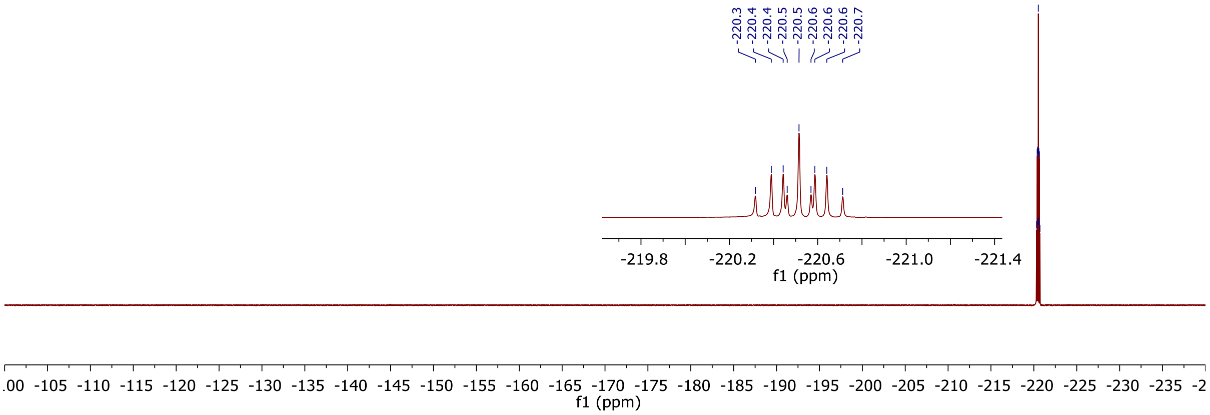

1H NMR (400 MHz, CDCl3)

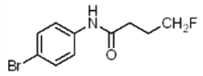

2b

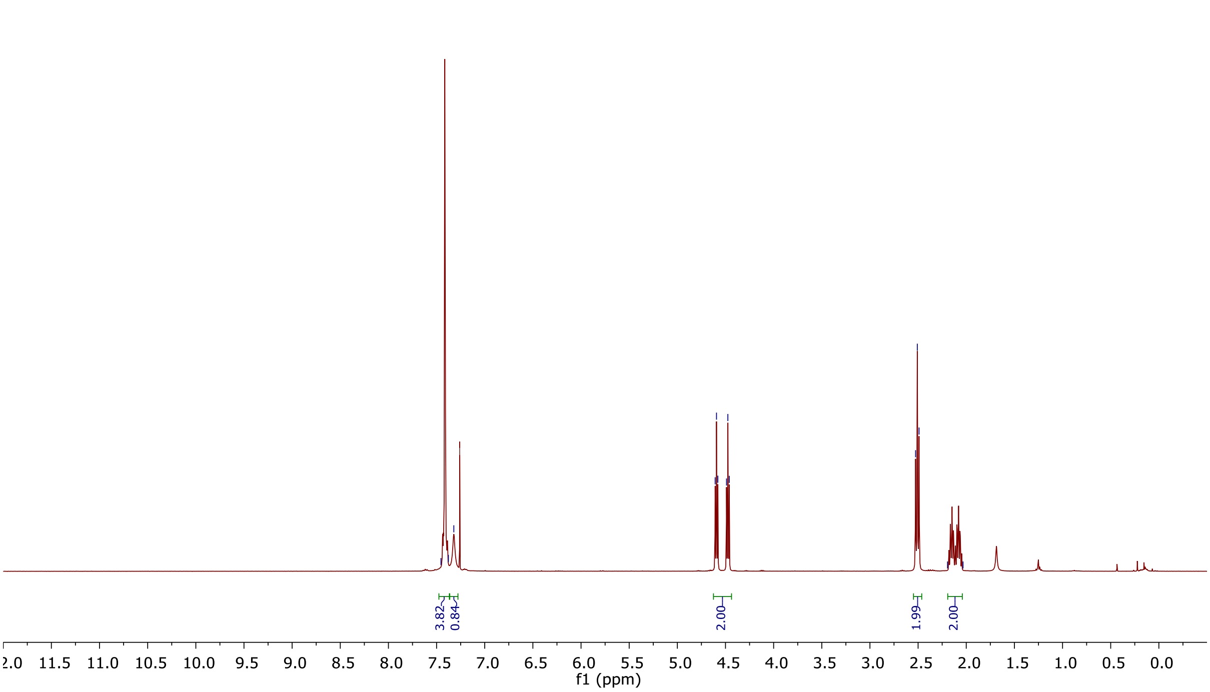

13C NMR (101 MHz, CDCl3)

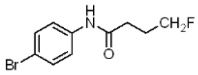

2b

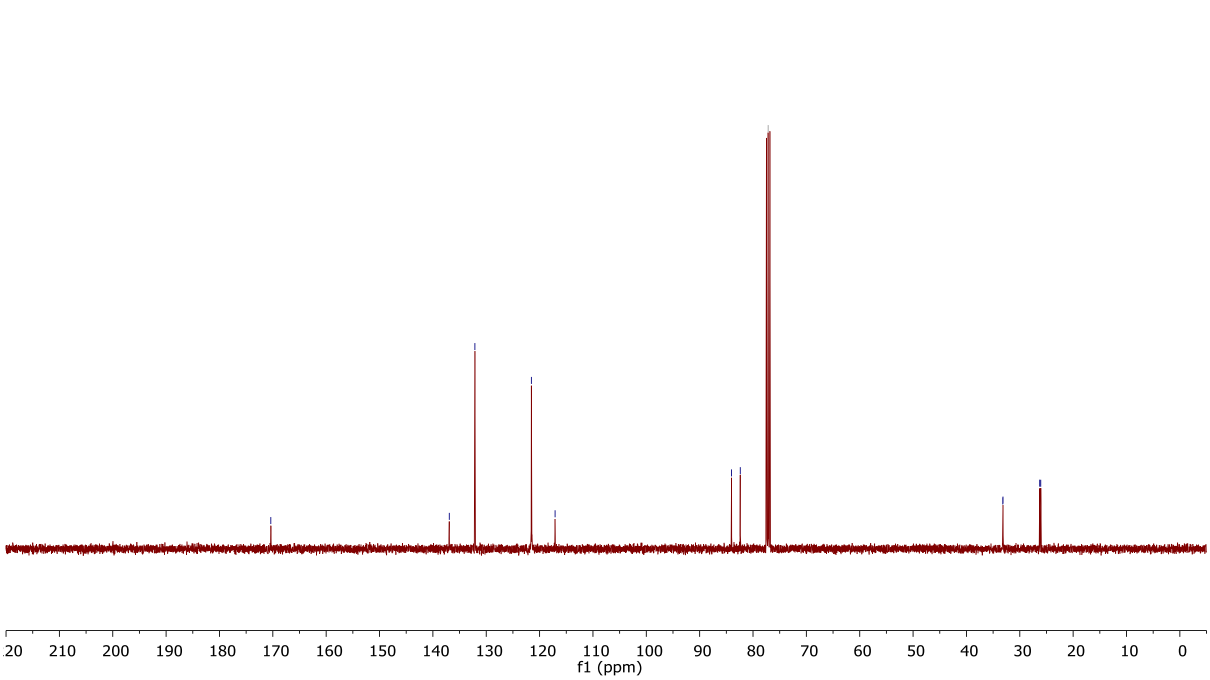

19F NMR (377 MHz, CDCl3)

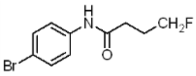

2b

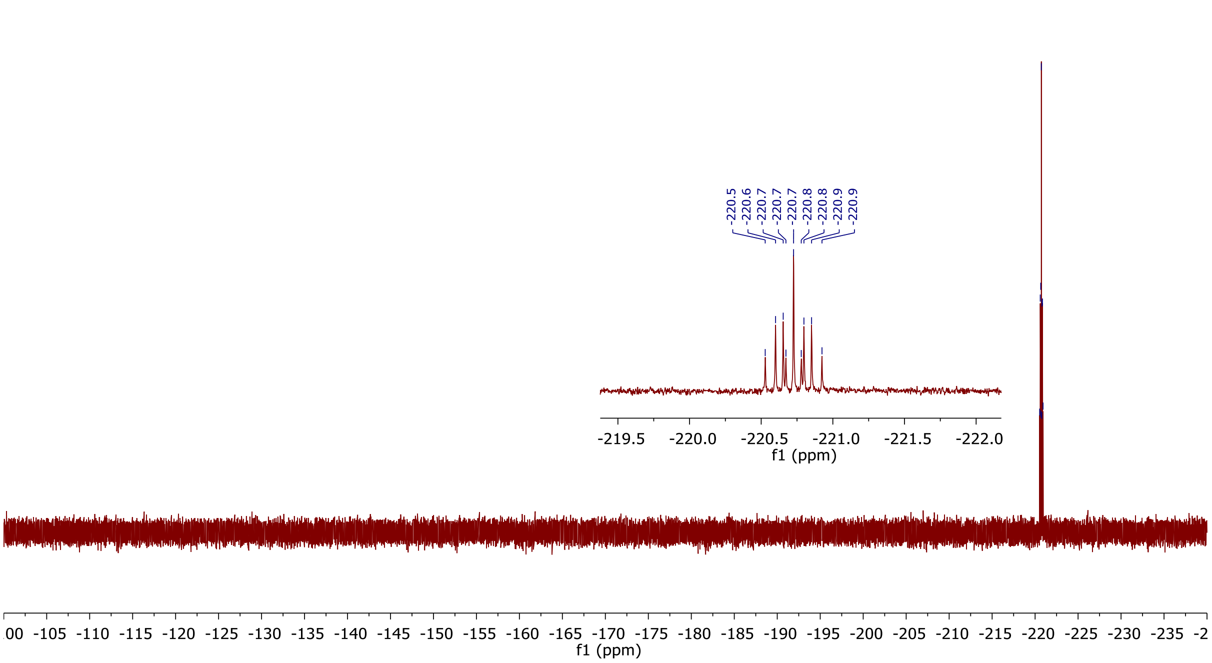

1H NMR (400 MHz, CDCl3)

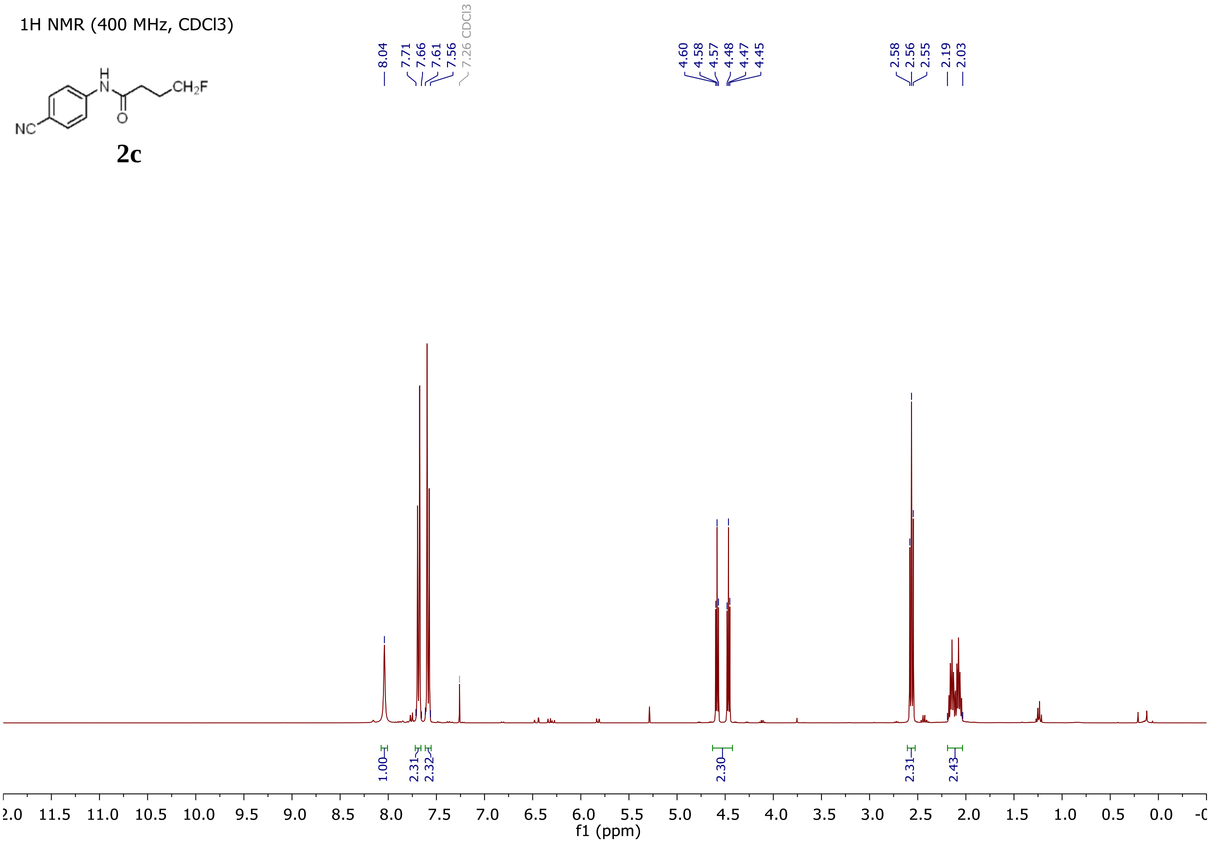

13C NMR (101 MHz, CDCl3)

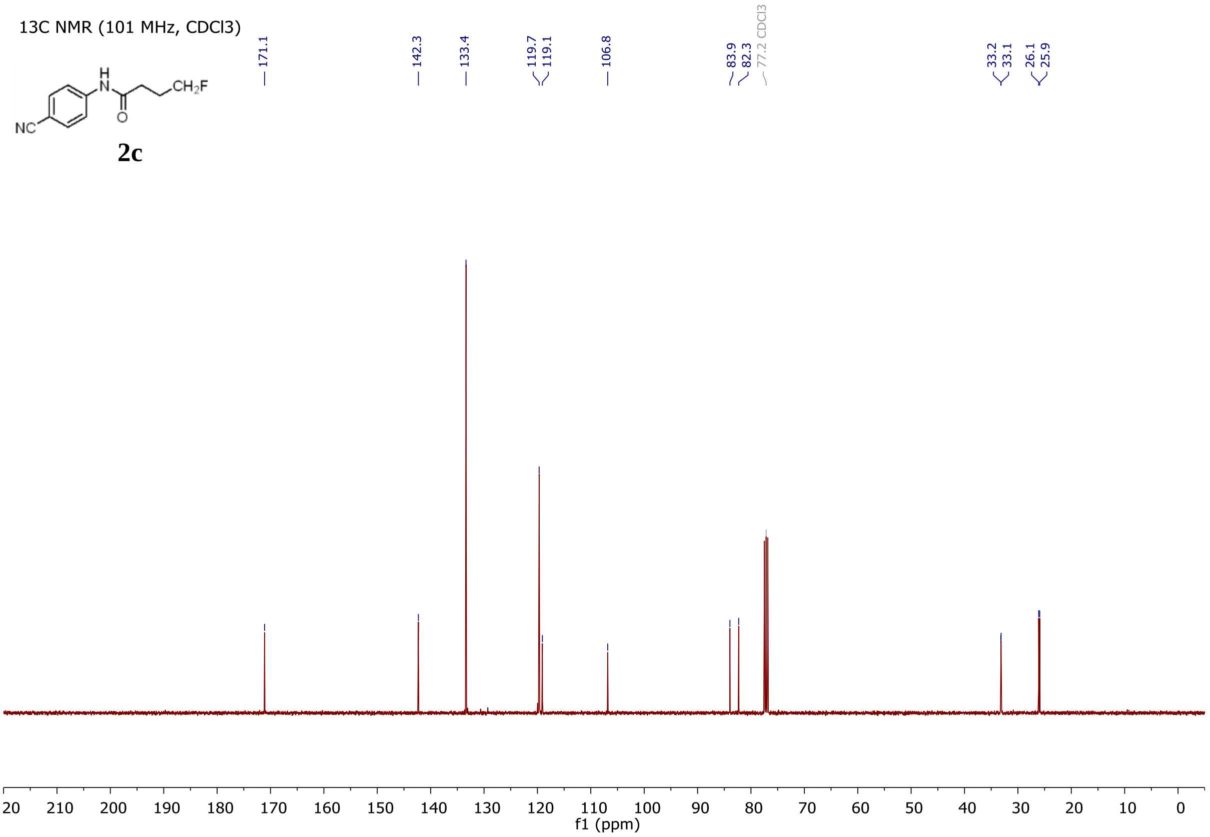

19F NMR (377 MHz, CDCl3)

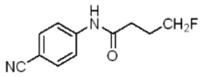

2c

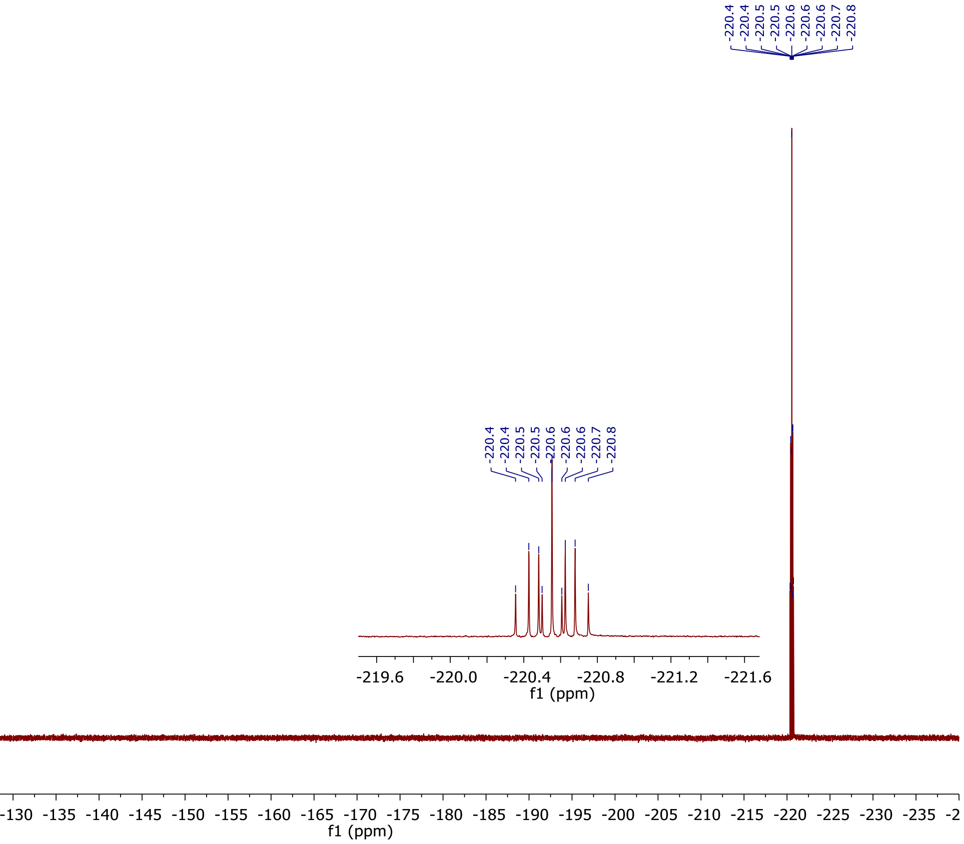

1H NMR (400 MHz, CDCl3)

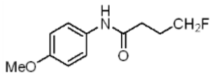

2d

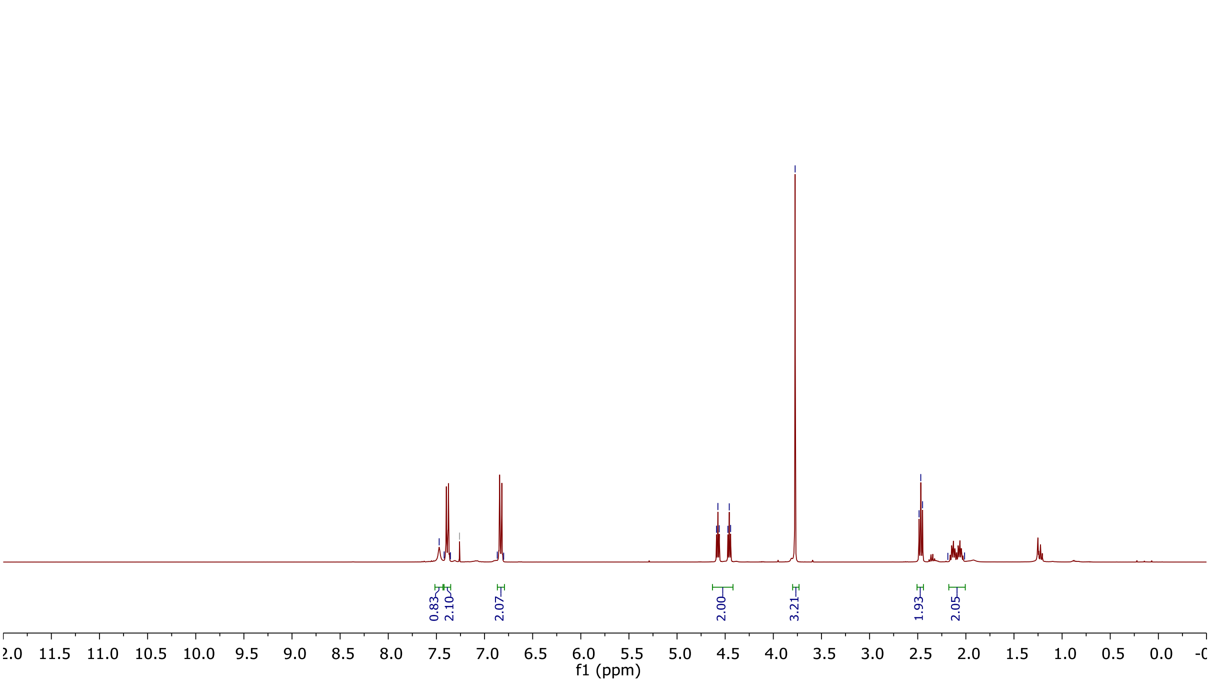

13C NMR (101 MHz, CDCl3)

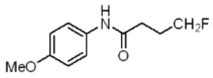

2d

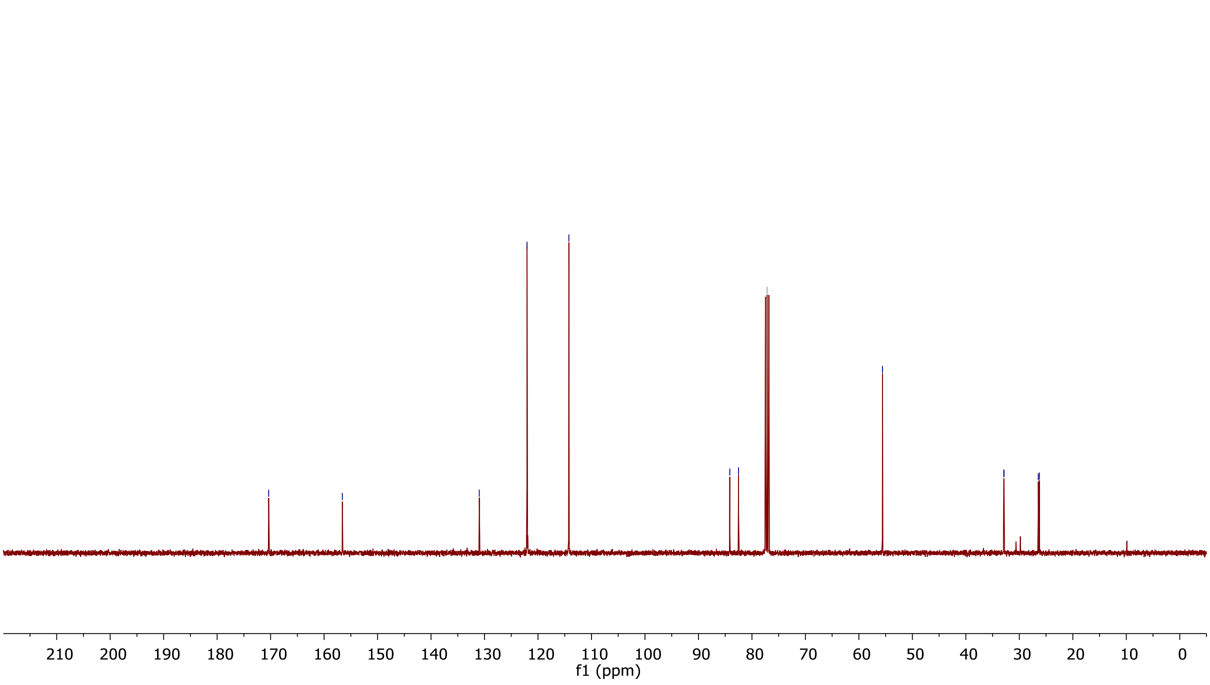

19F NMR (377 MHz, CDCl3)

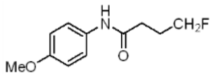

2d

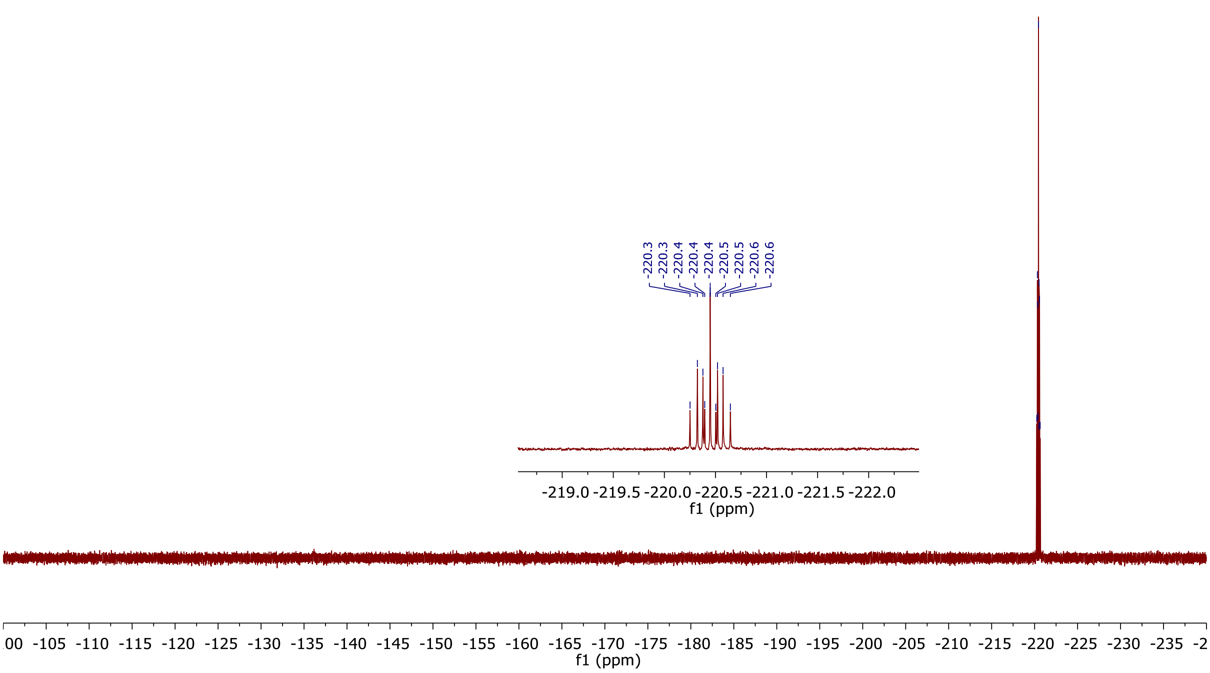

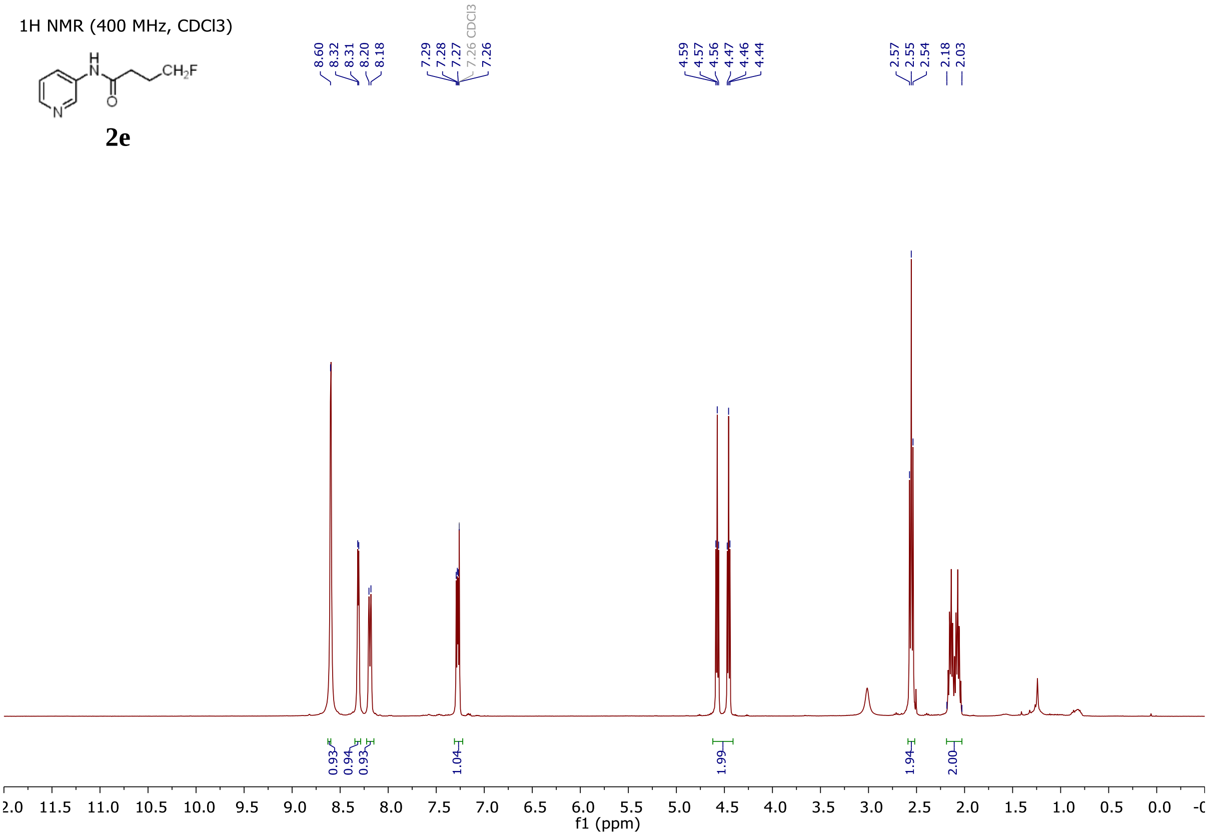
1H NMR (400 MHz, CDCl3)
2e
8.60 8.32 8.31 8.20 8.18 7.29 7.28 7.27 7.26 CDCl3 7.26
4.59 4.57 4.56 4.47 4.46 4.44
2.57 2.55 2.54 2.18 2.03
0.93 0.94 0.93 1.04 1.99 1.94 2.00
f1 (ppm)

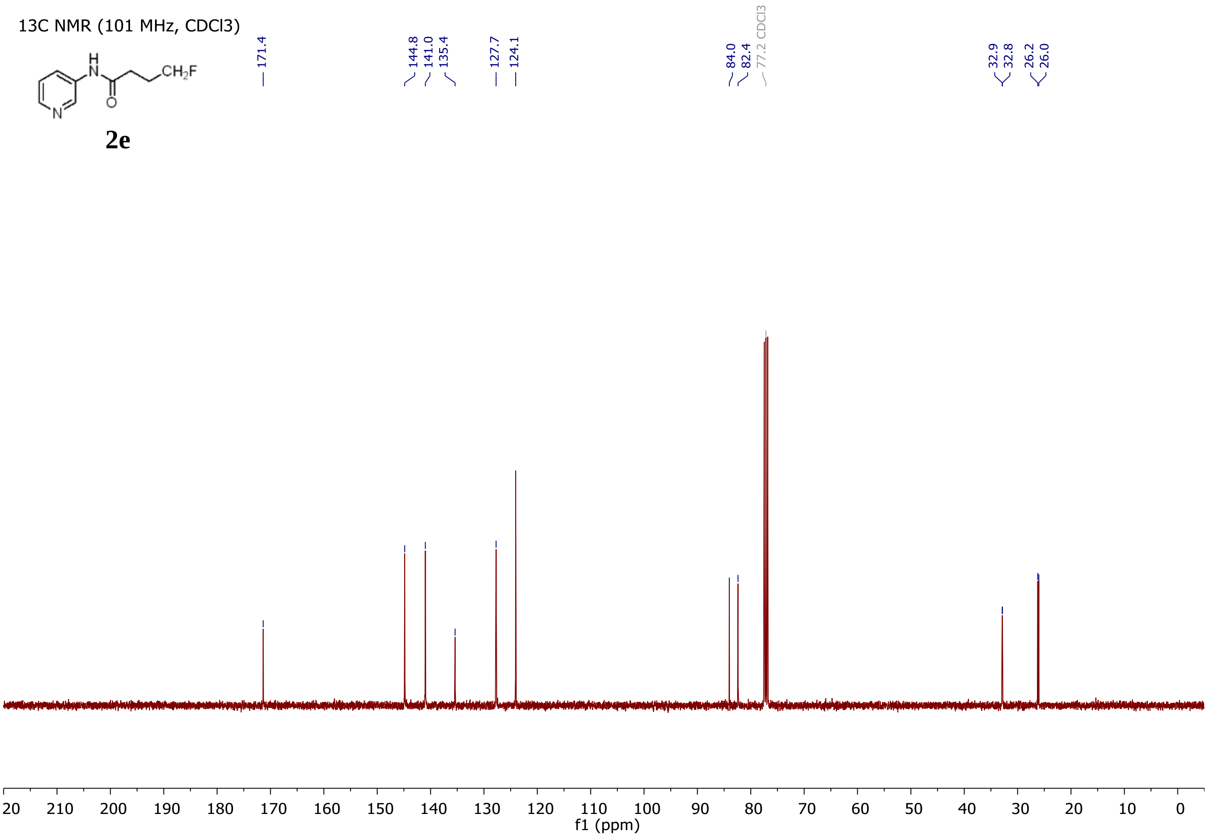
13C NMR (101 MHz, CDCl3)
2e
171.4
144.8 141.0 135.4 127.7 124.1
84.0 82.4 77.2 CDCl3
32.9 32.8 26.2 26.0
f1 (ppm)

19F NMR (377 MHz, CDCl3)

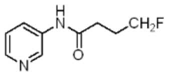

2e

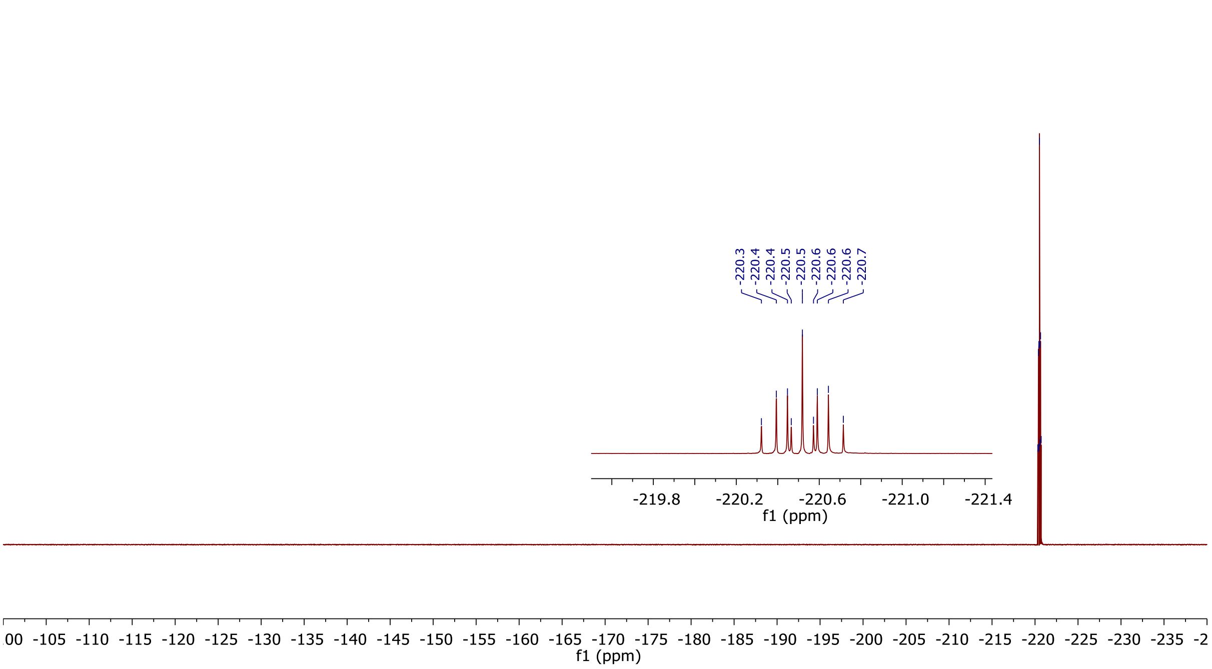

1H NMR (400 MHz, Acetone)

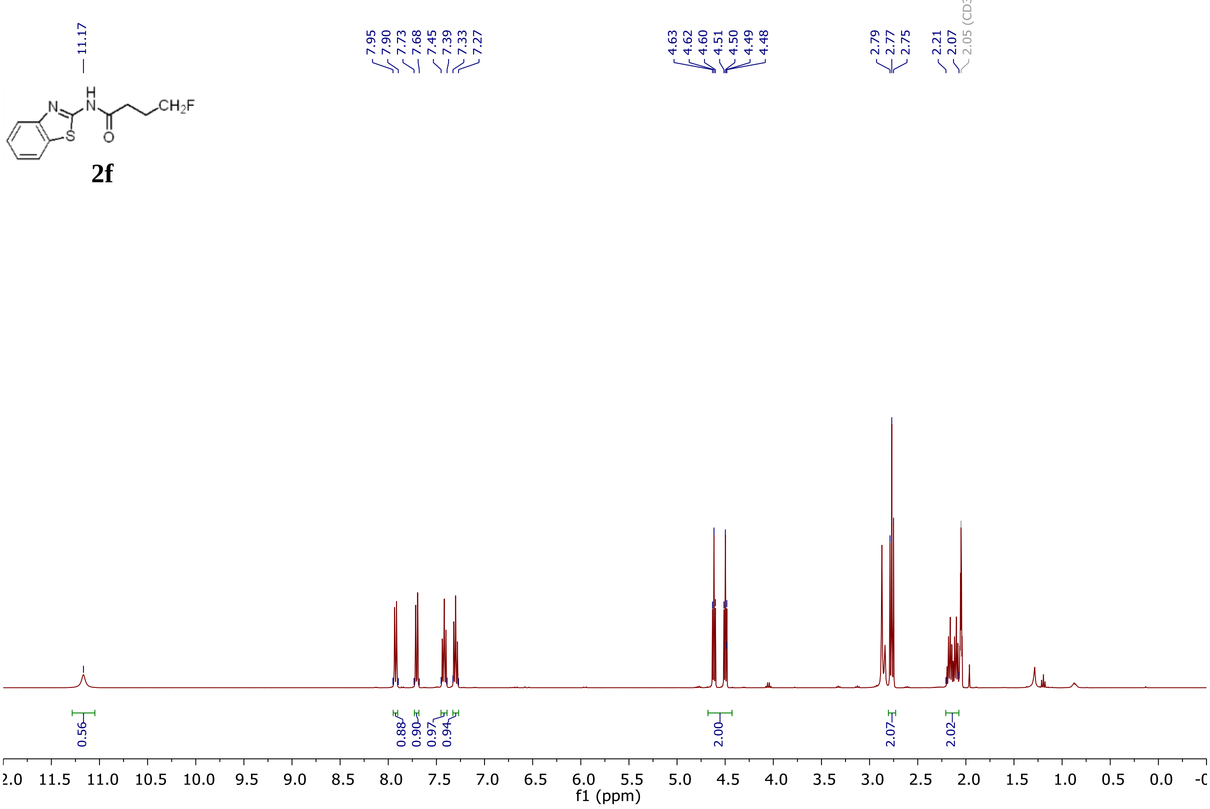

13C NMR (101 MHz, Acetone)

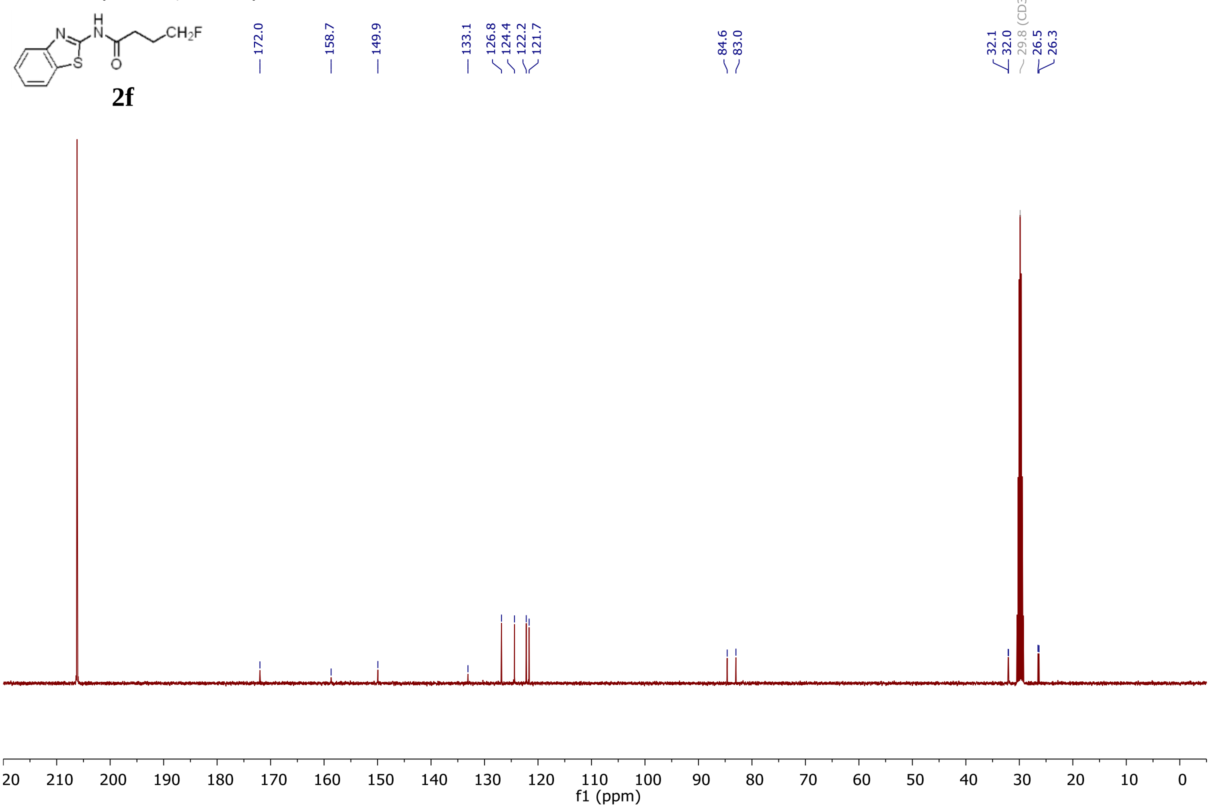

19F NMR (377 MHz, Acetone)

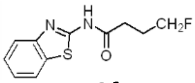

2f

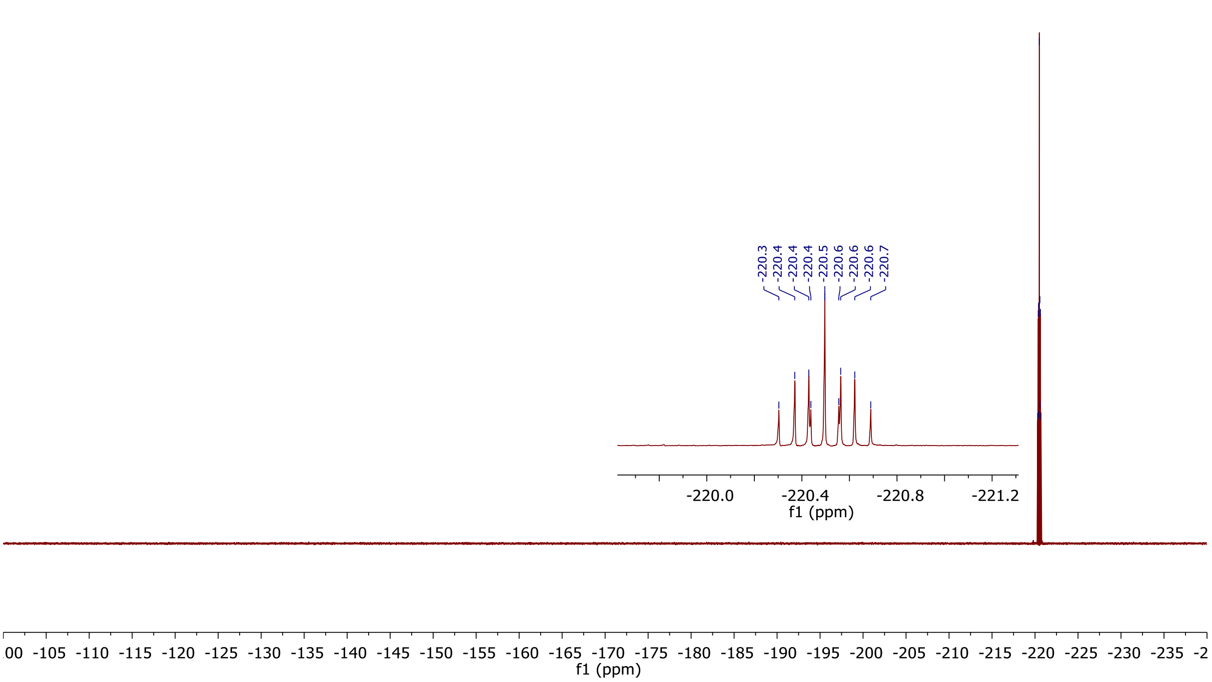

1H NMR (400 MHz, CDCl3)

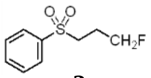

2g

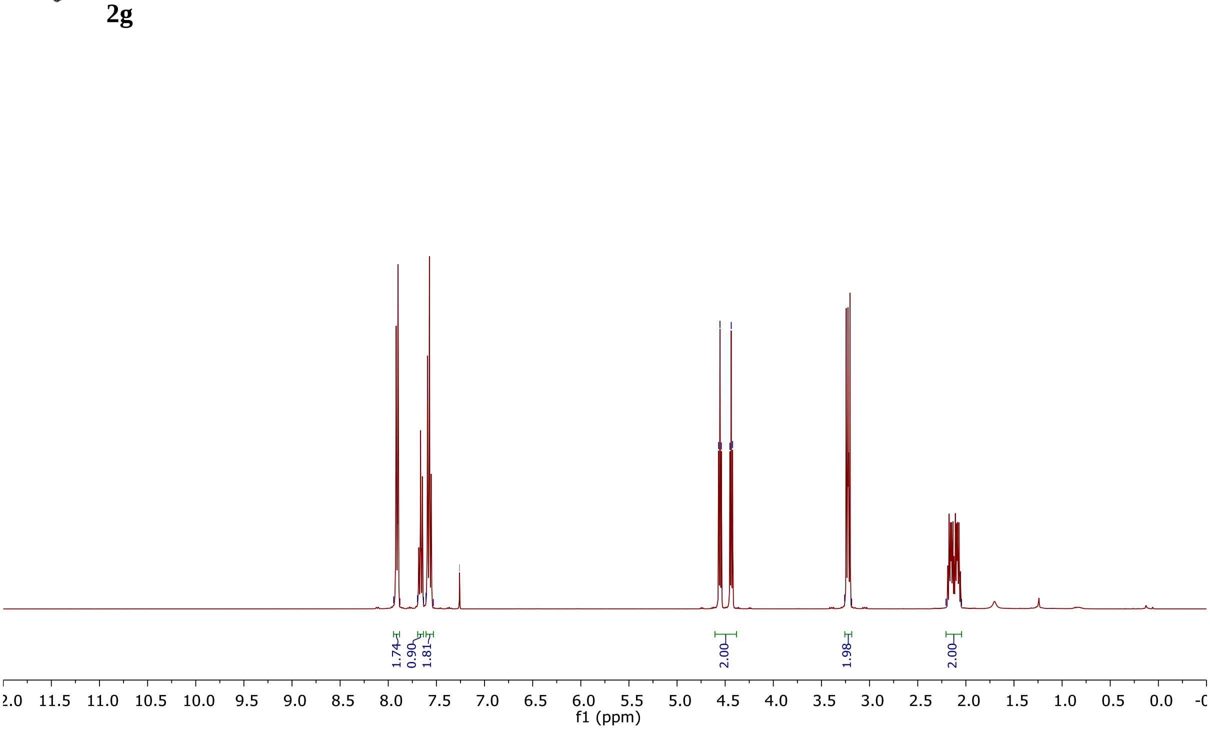

13C NMR (101 MHz, CDCl3)

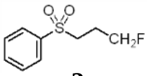

2g

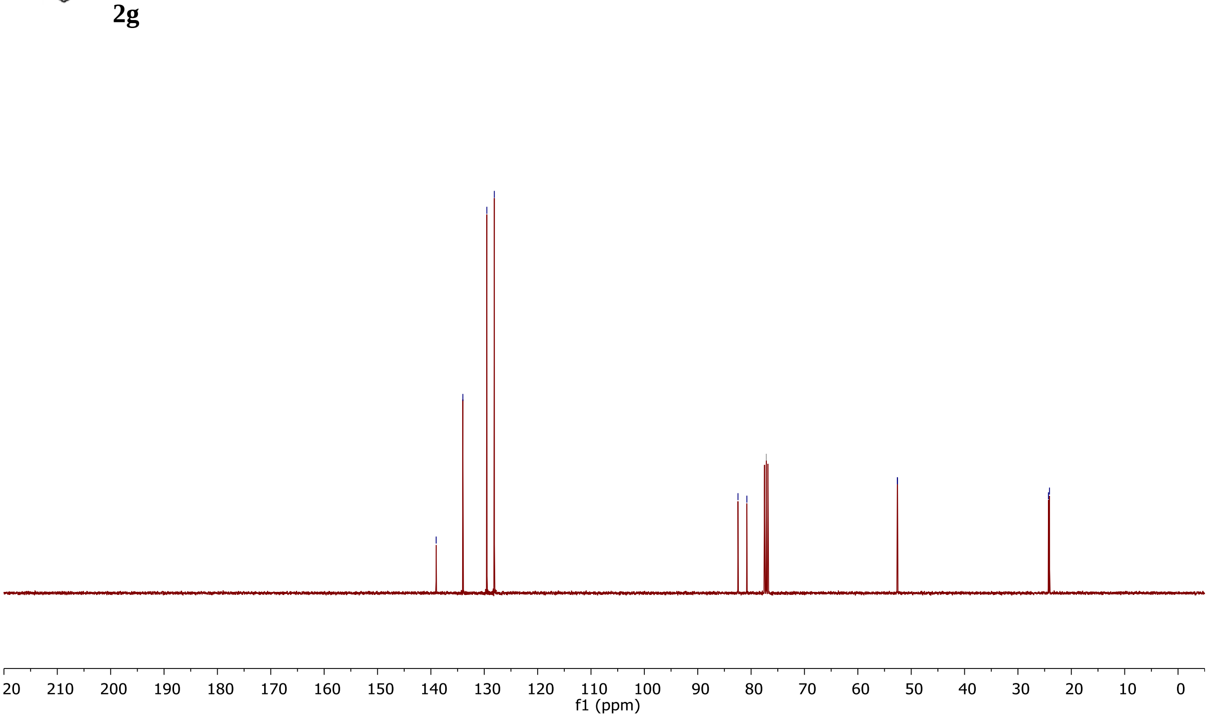

19F NMR (377 MHz, CDCl3)

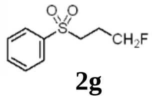

2g

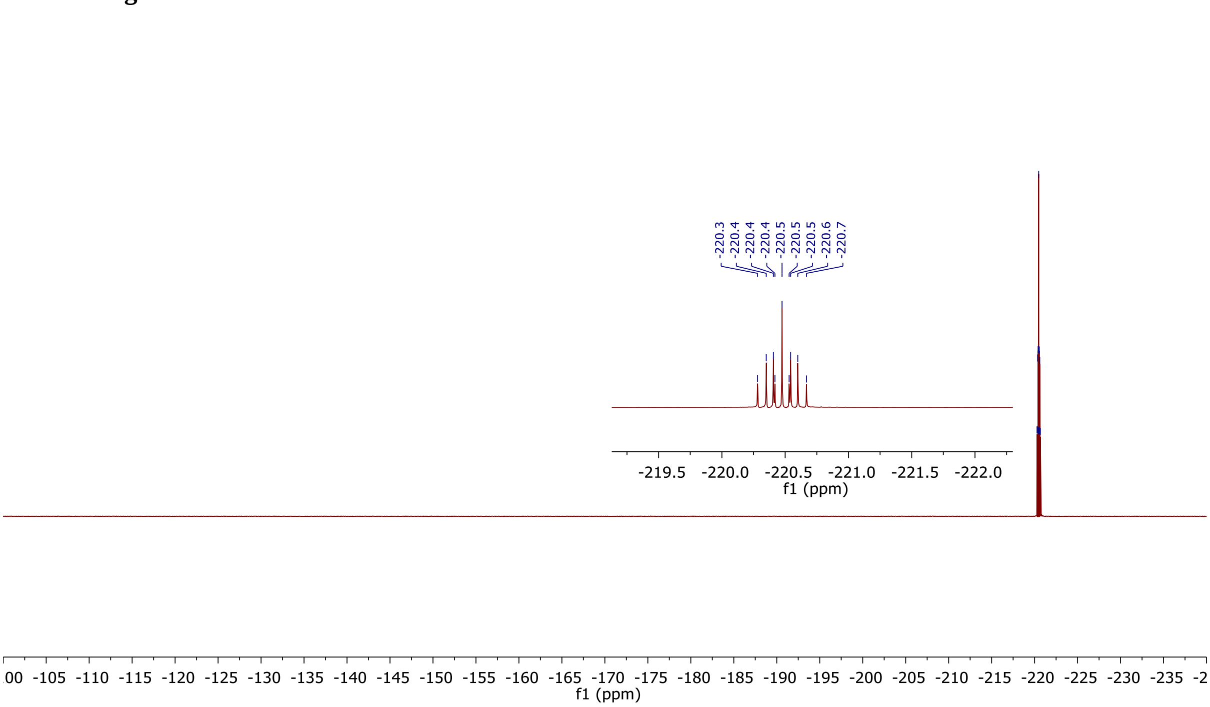

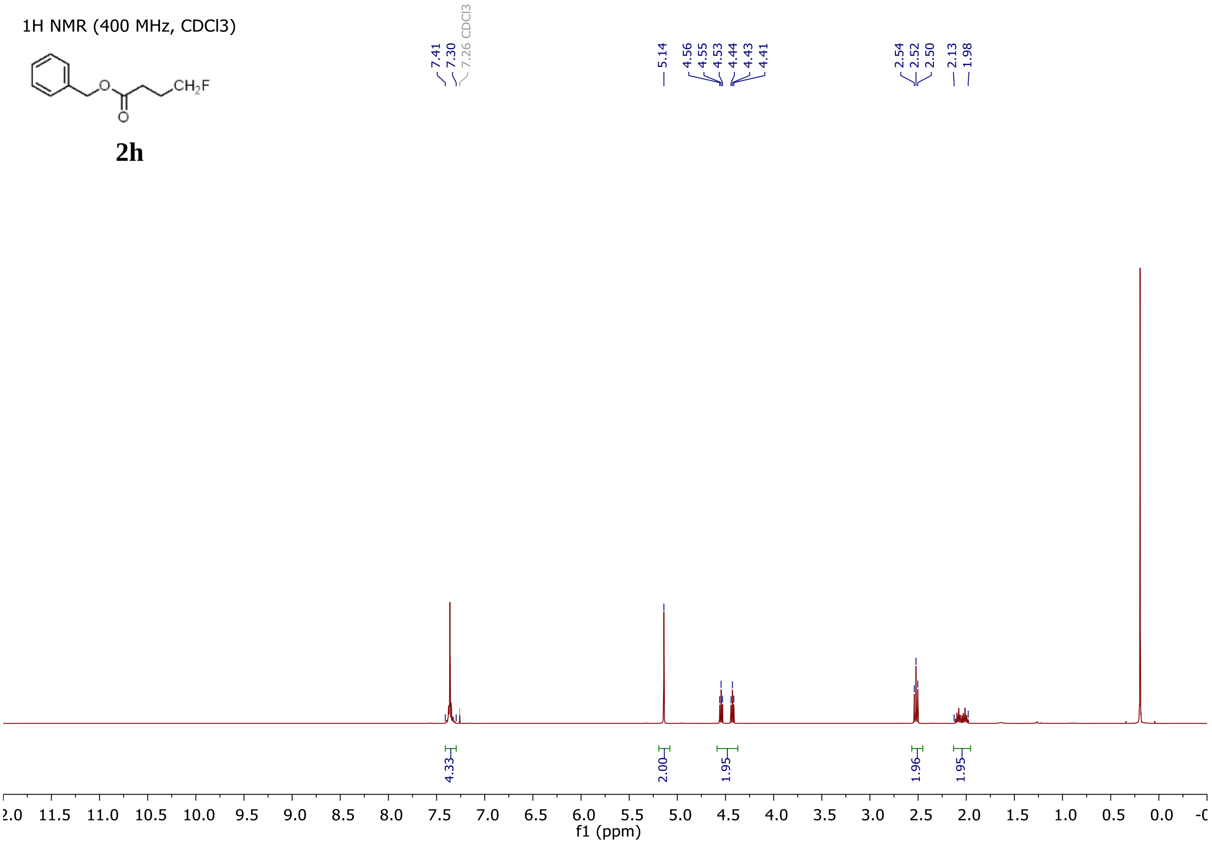

The product coeluted with silane by-products (δ: 0.19 ppm).

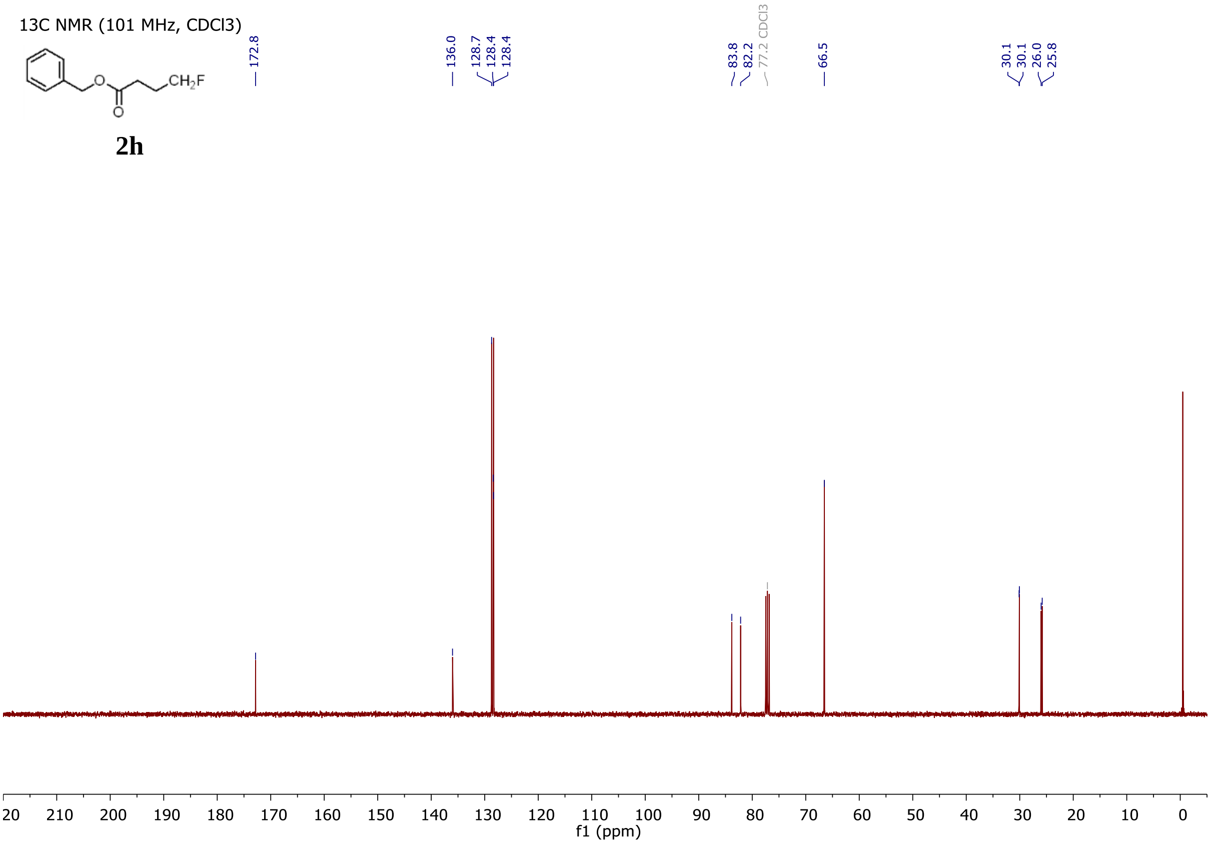

19F NMR (377 MHz, CDCl3)

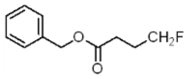

2h

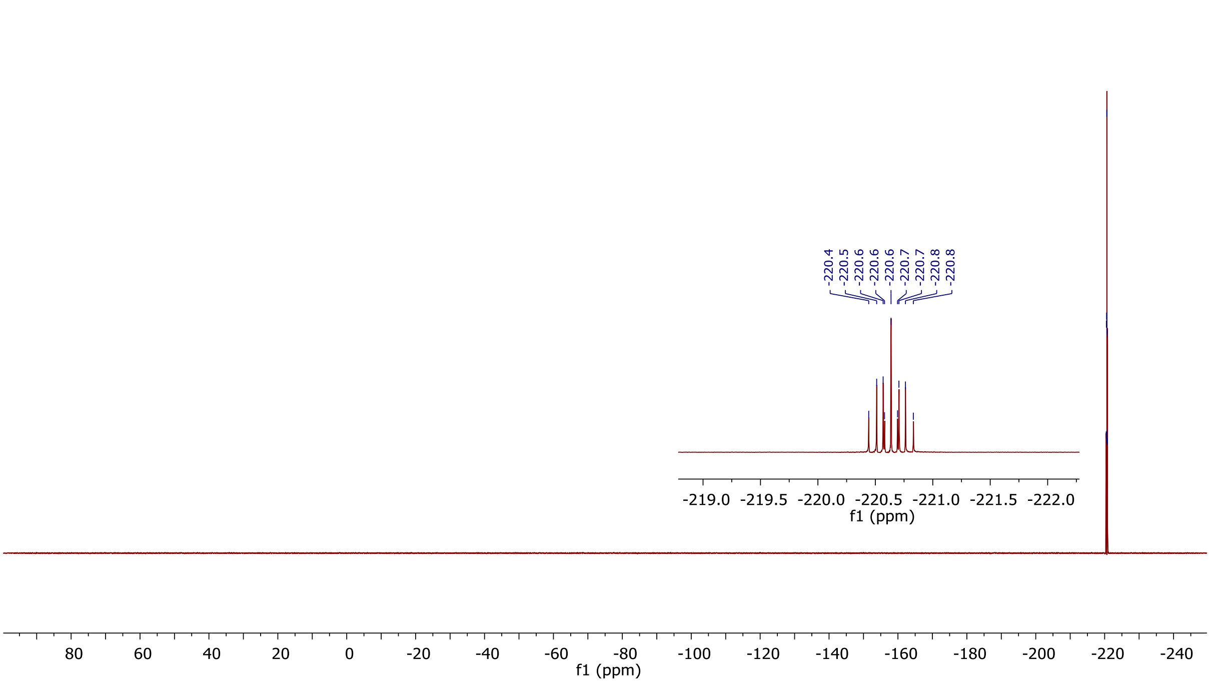

1H NMR (400 MHz, CDCl3)

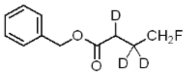

2i

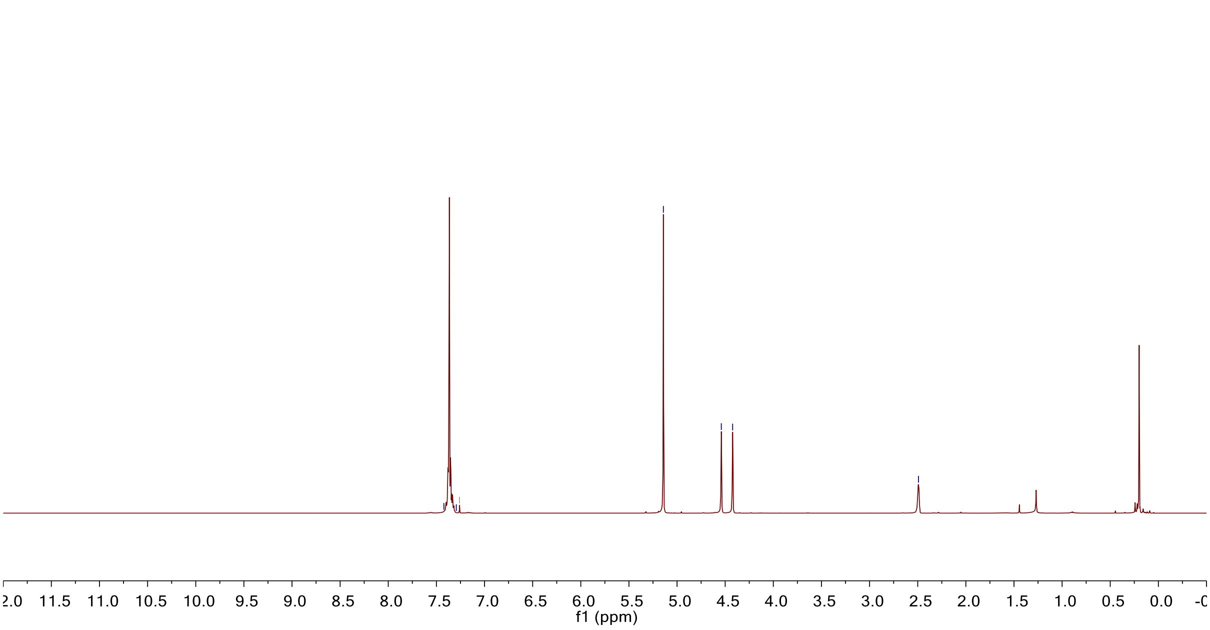

13C NMR (101 MHz, CDCl3)

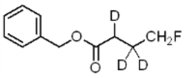

2i

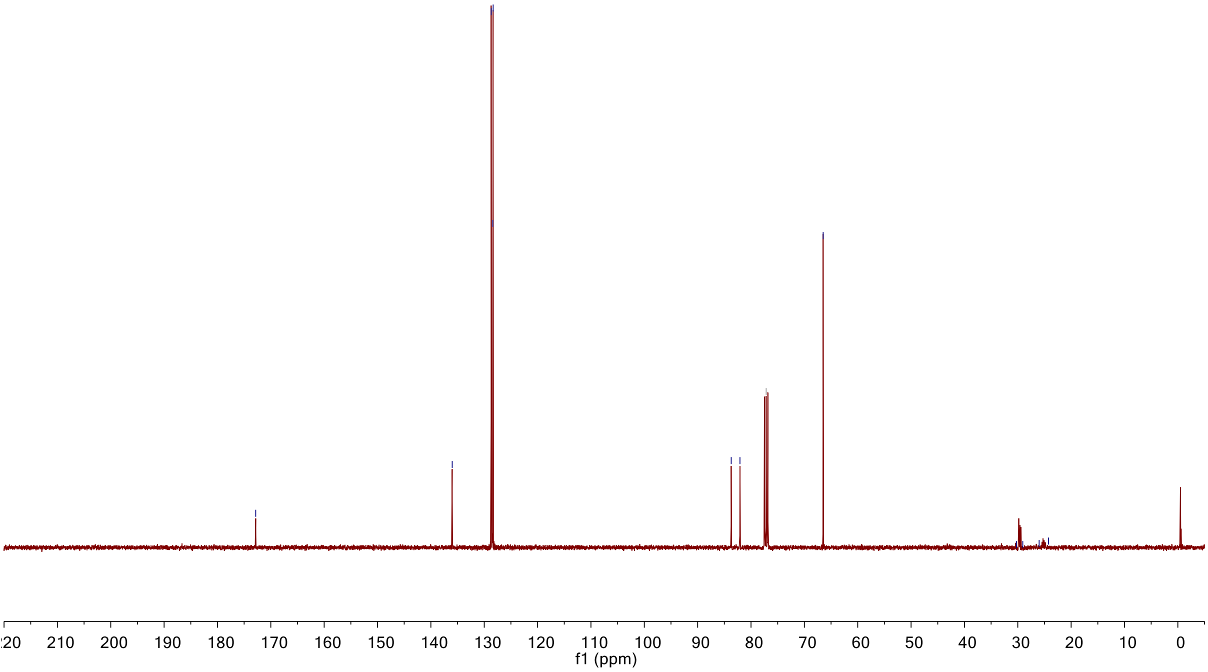

19F NMR (377 MHz, CDCl3)

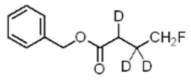

2i

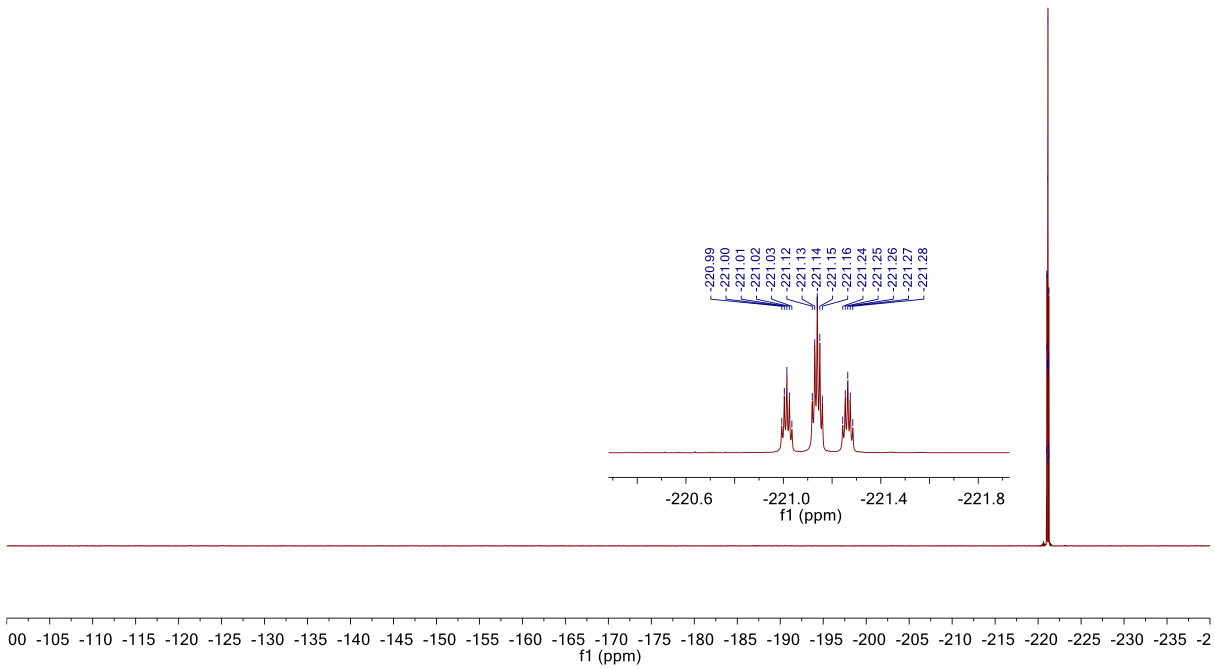

1H NMR (400 MHz, CDCl3)

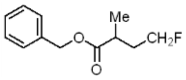

2j

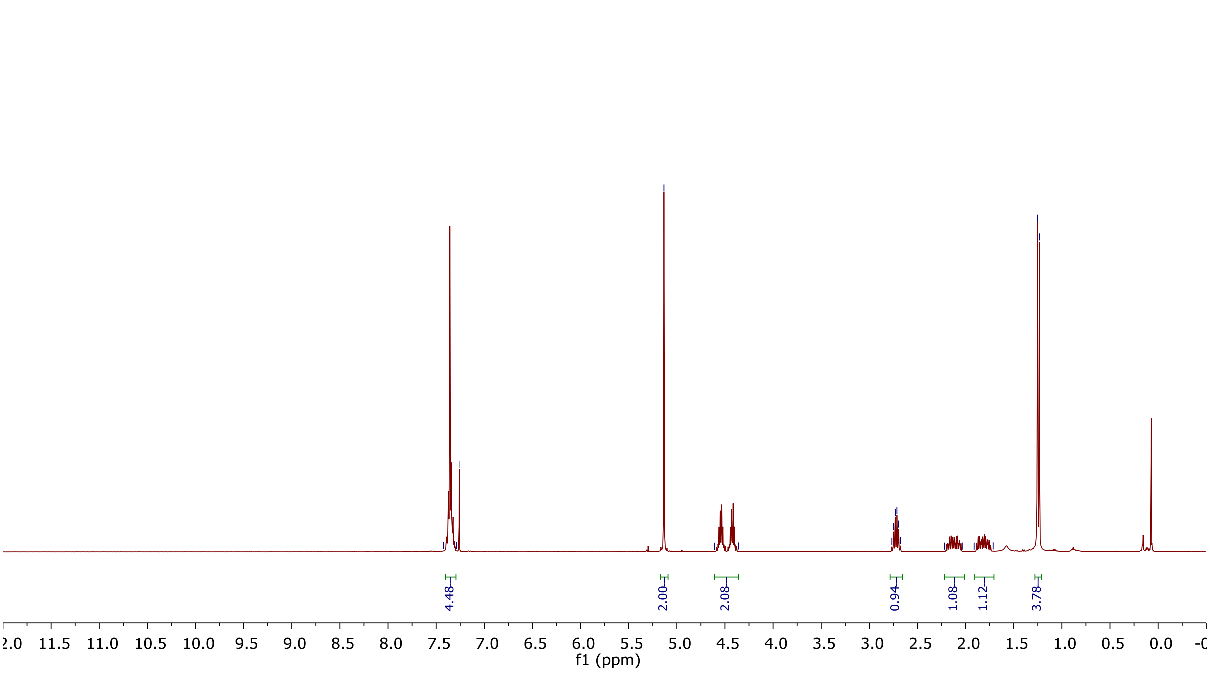

13C NMR (101 MHz, CDCl3)

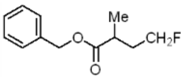

2j

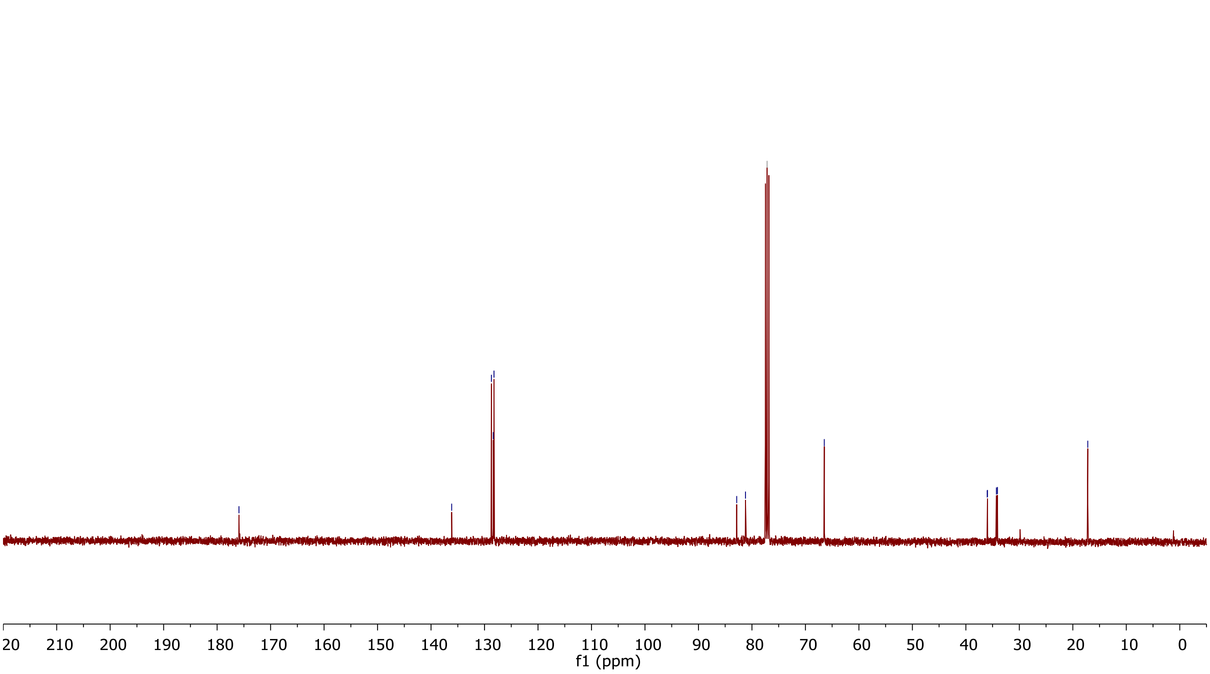

19F NMR (377 MHz, CDCl3)

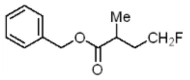

2j

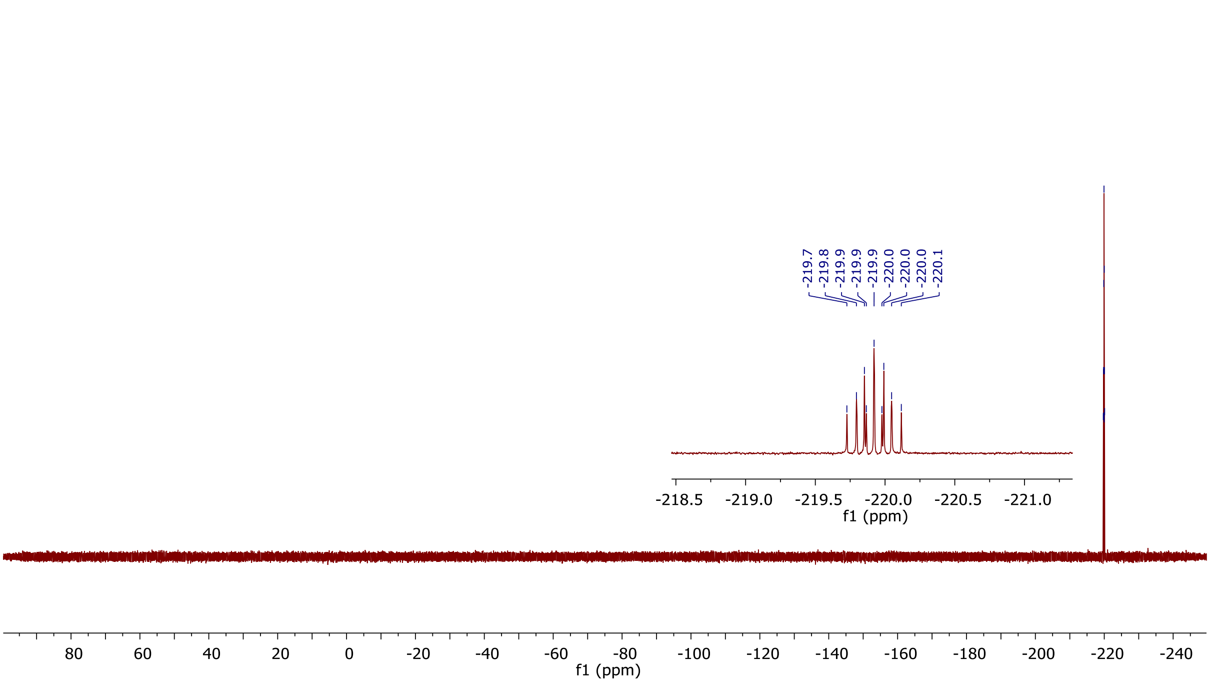

1H NMR (400 MHz, CDCl3)

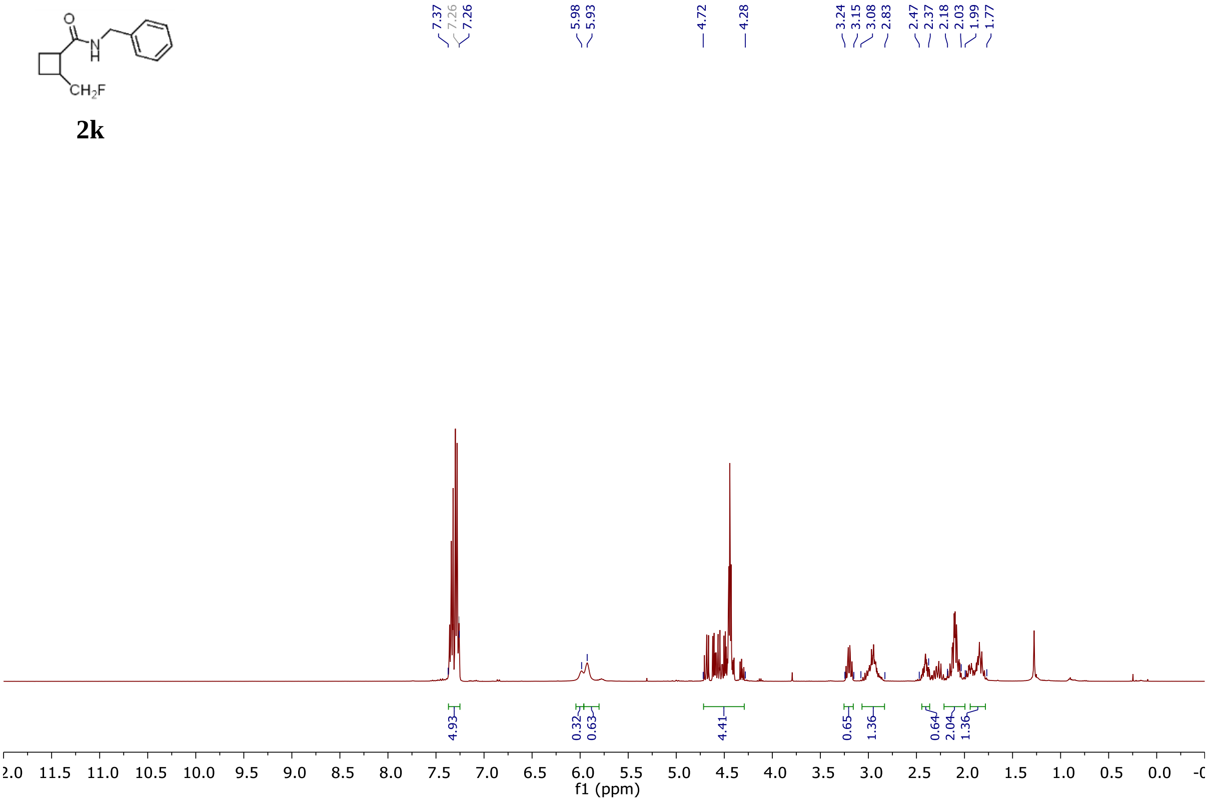

13C NMR (101 MHz, CDCl3)

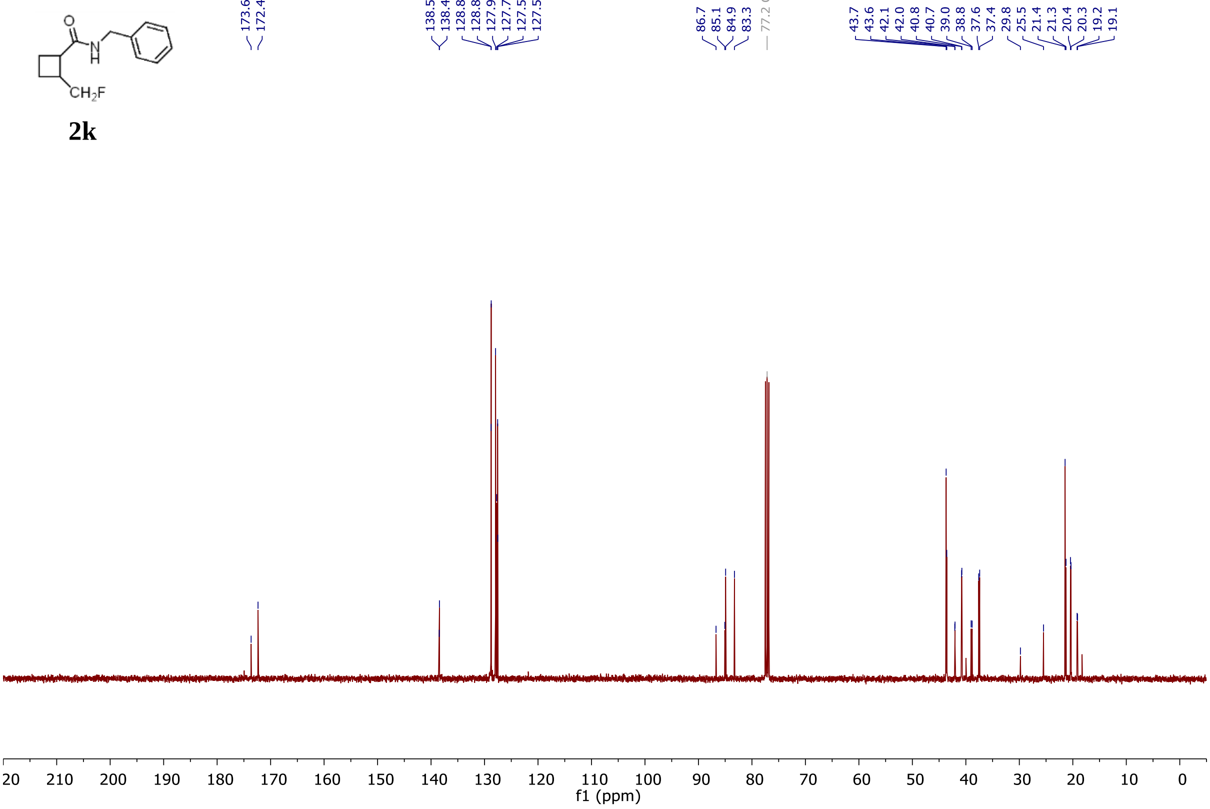

19F NMR (377 MHz, CDCl3)

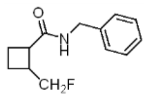

2k

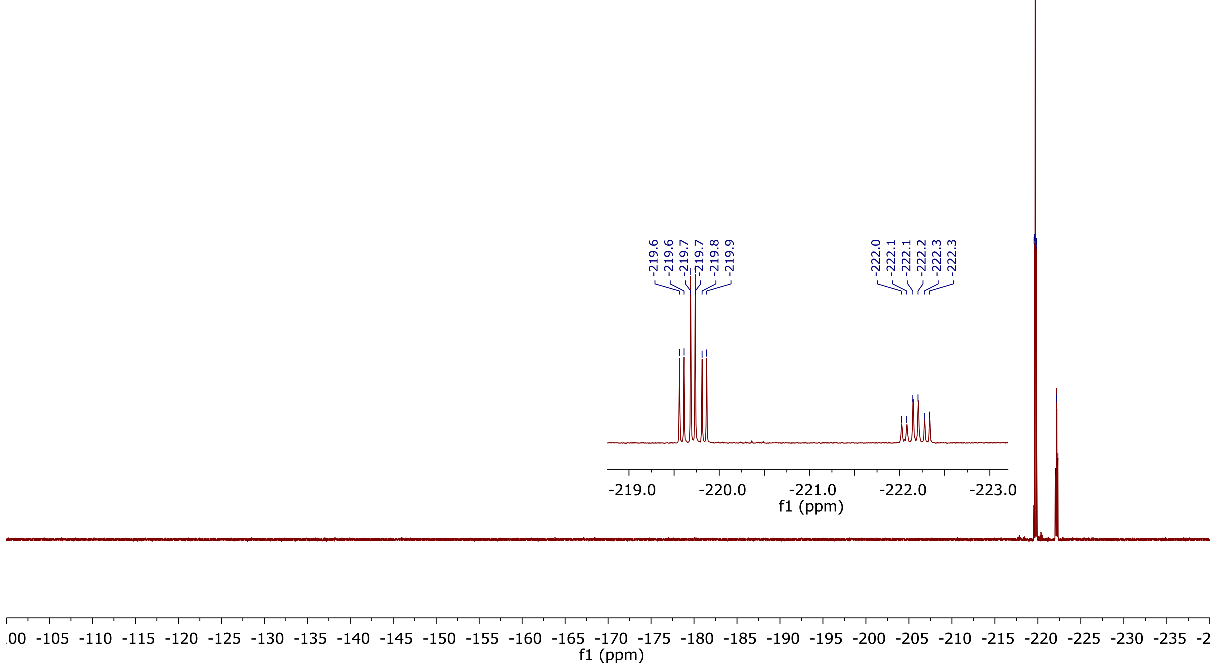

1H NMR (400 MHz, CDCl3)

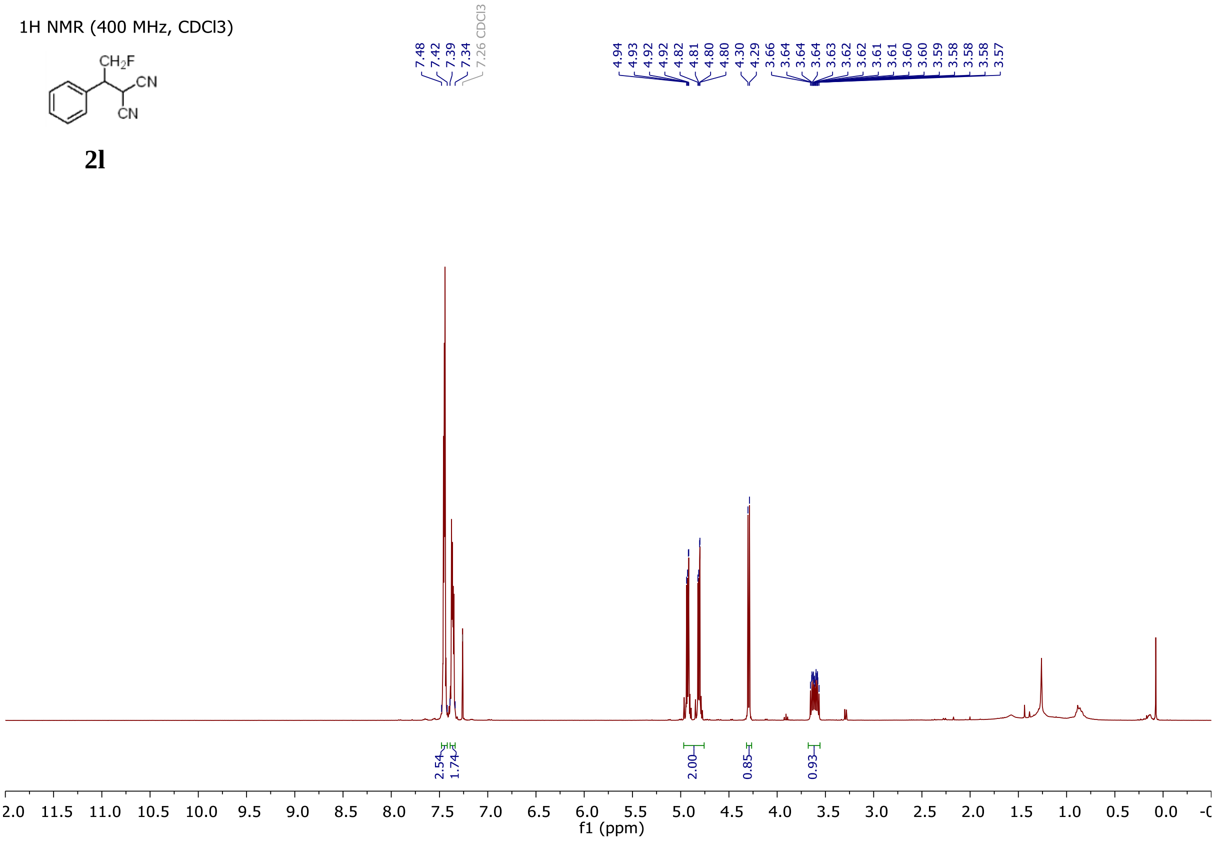

2l

13C NMR (101 MHz, CDCl3)

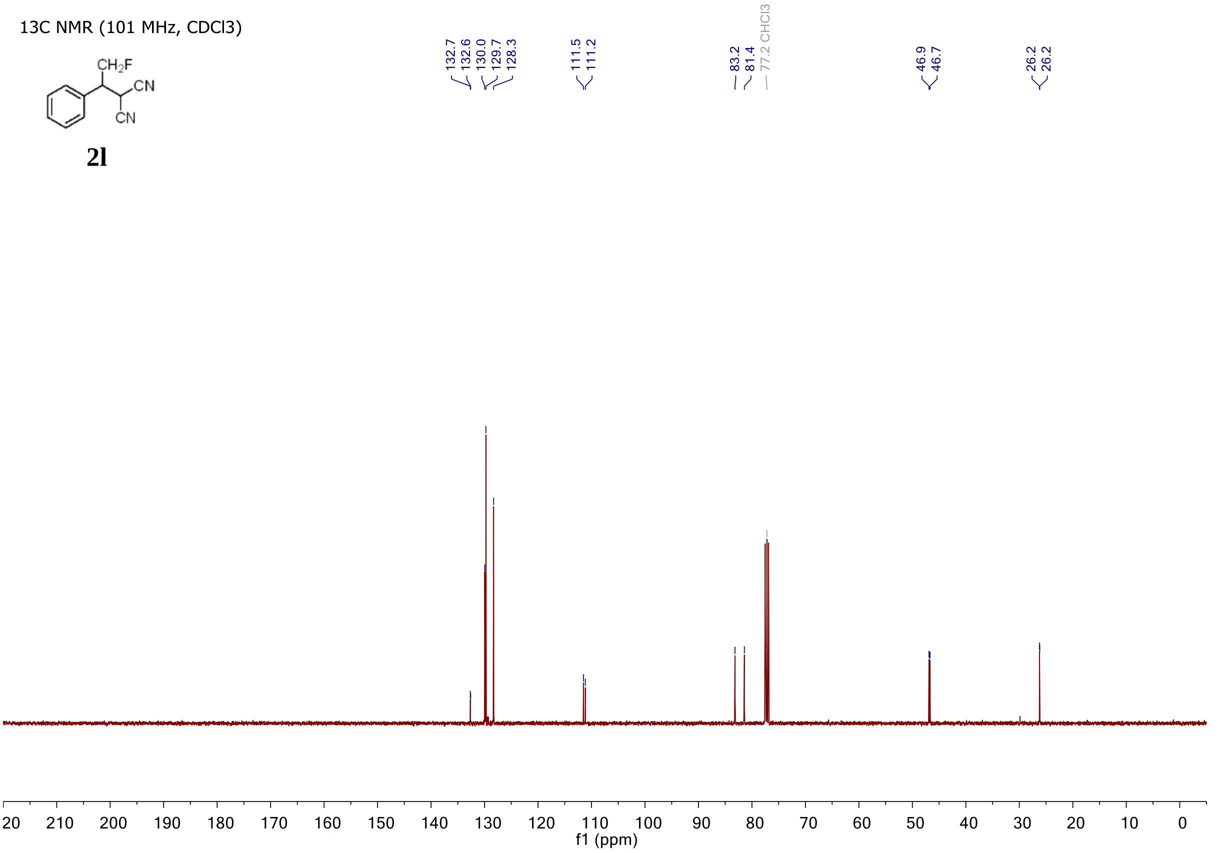

2l

19F NMR (377 MHz, CDCl3)

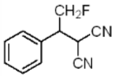

2l

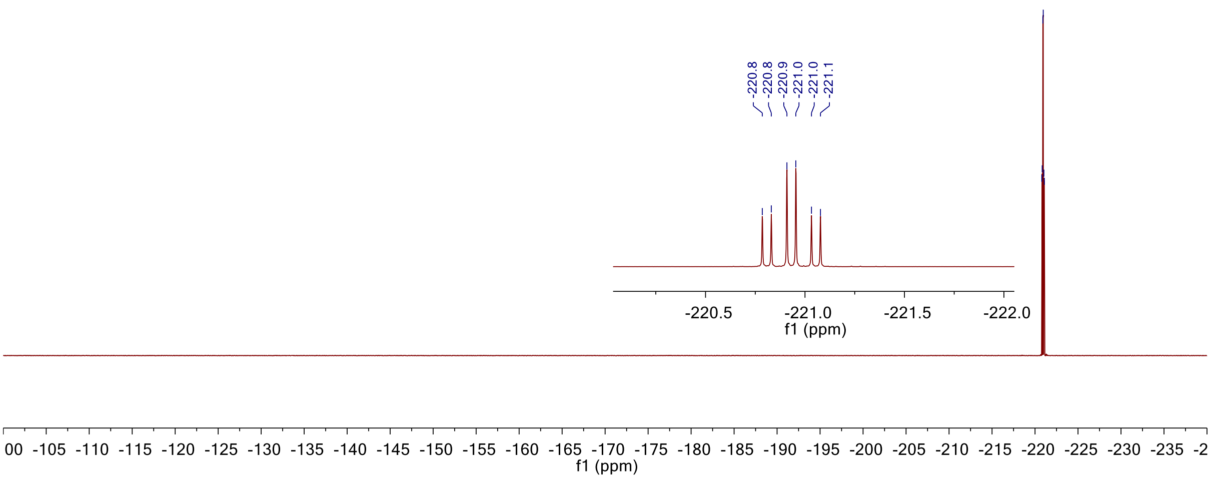

1H NMR (400 MHz, CDCl3)

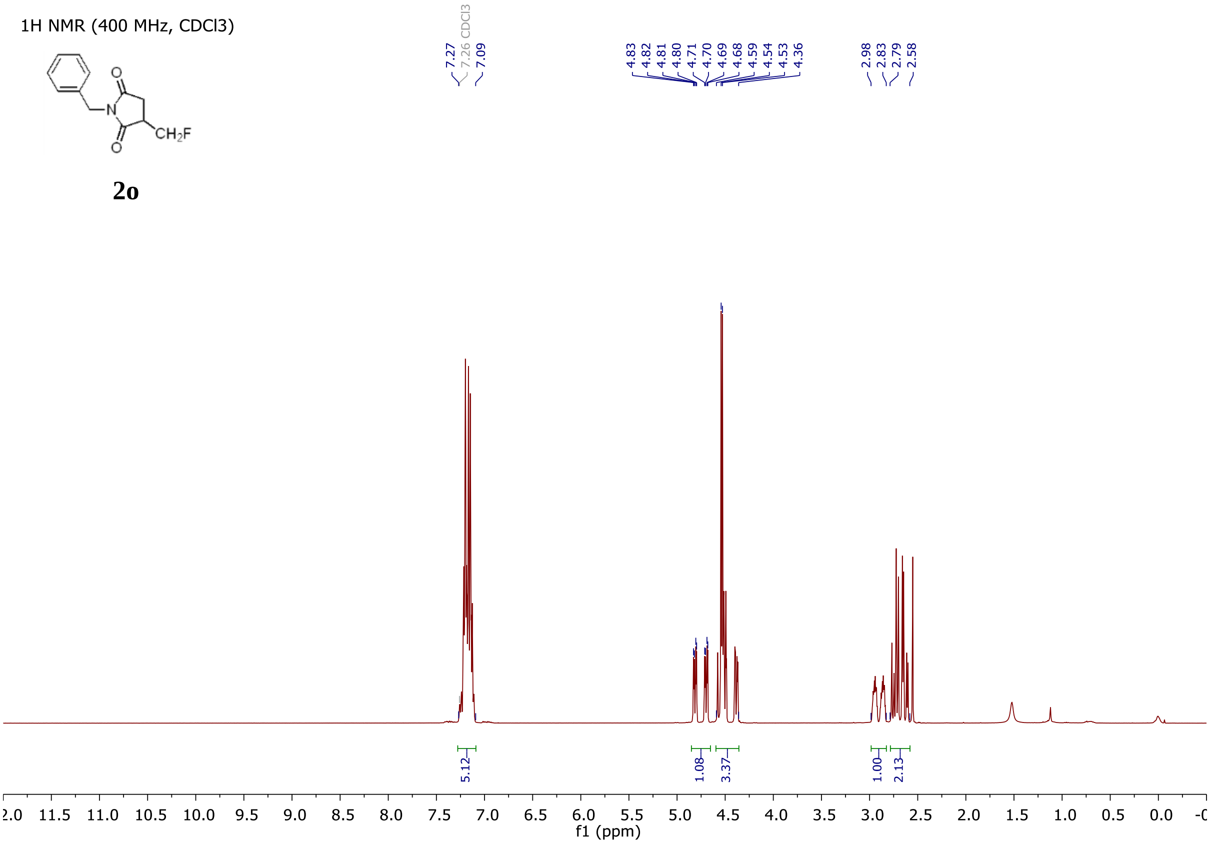

2o

13C NMR (101 MHz, CDCl3)

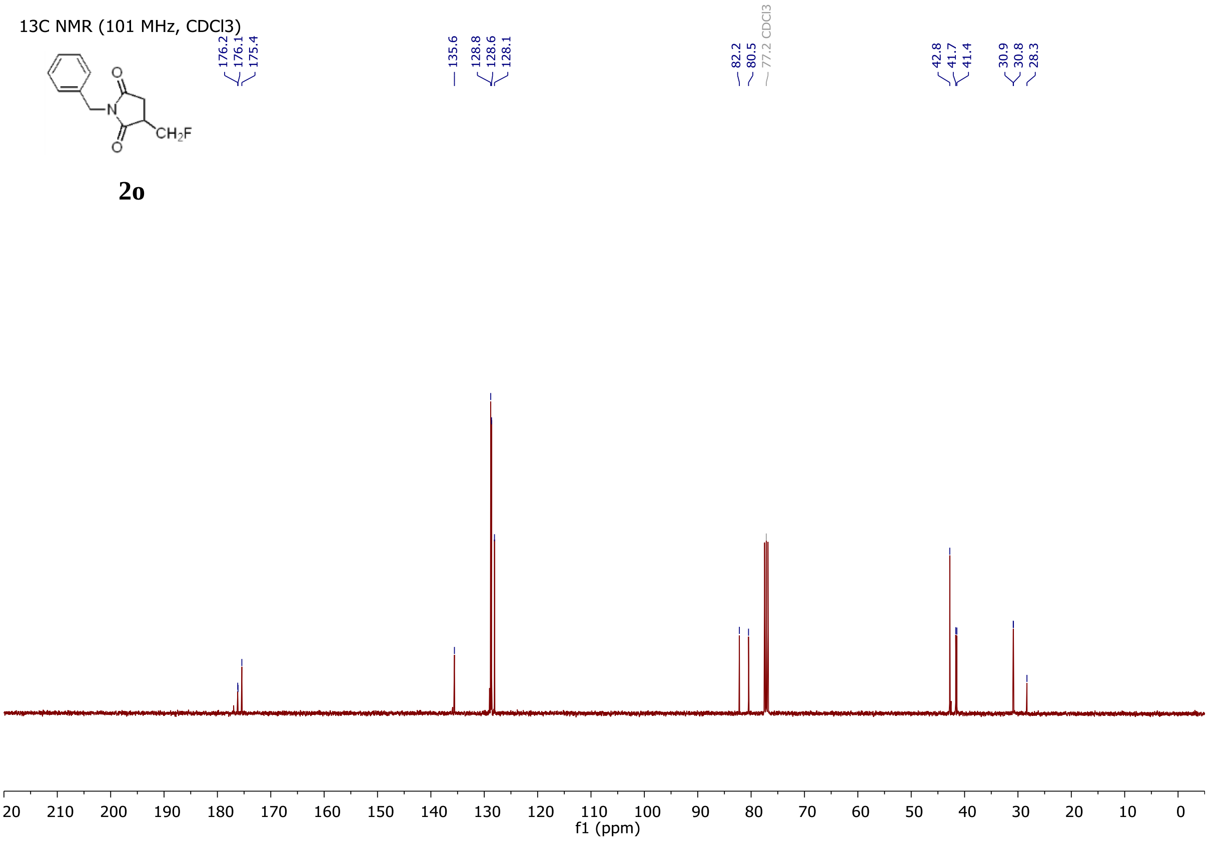

2o

19F NMR (377 MHz, CDCl3)

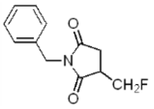

2o

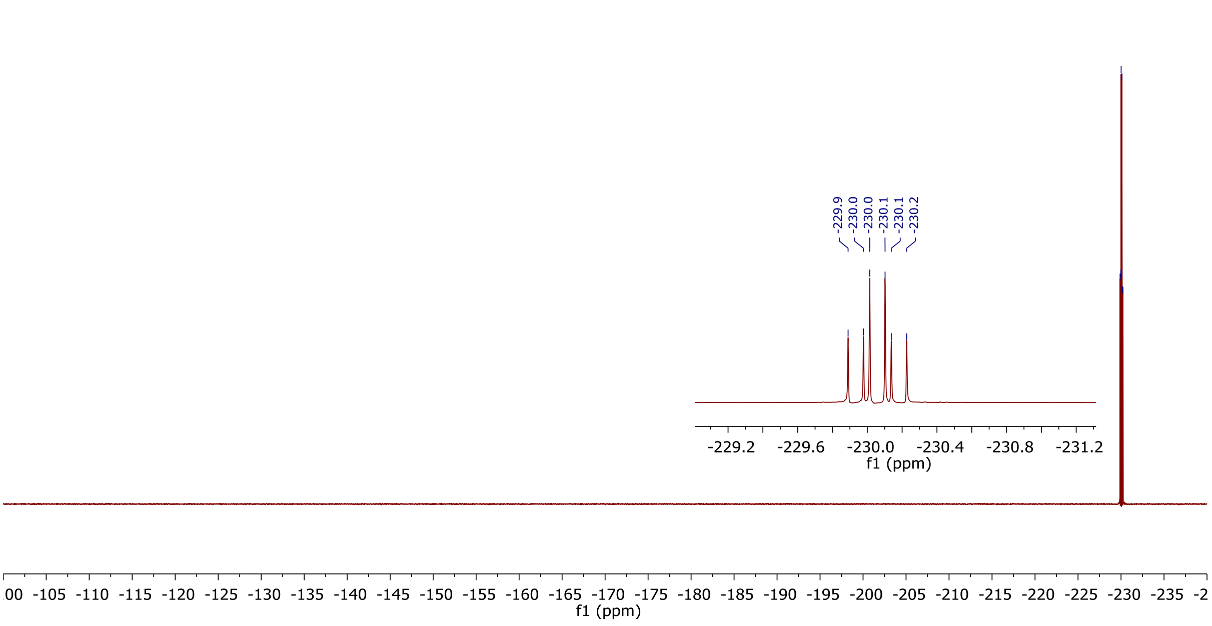

1H NMR (400 MHz, CDCl3)

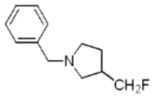

2p

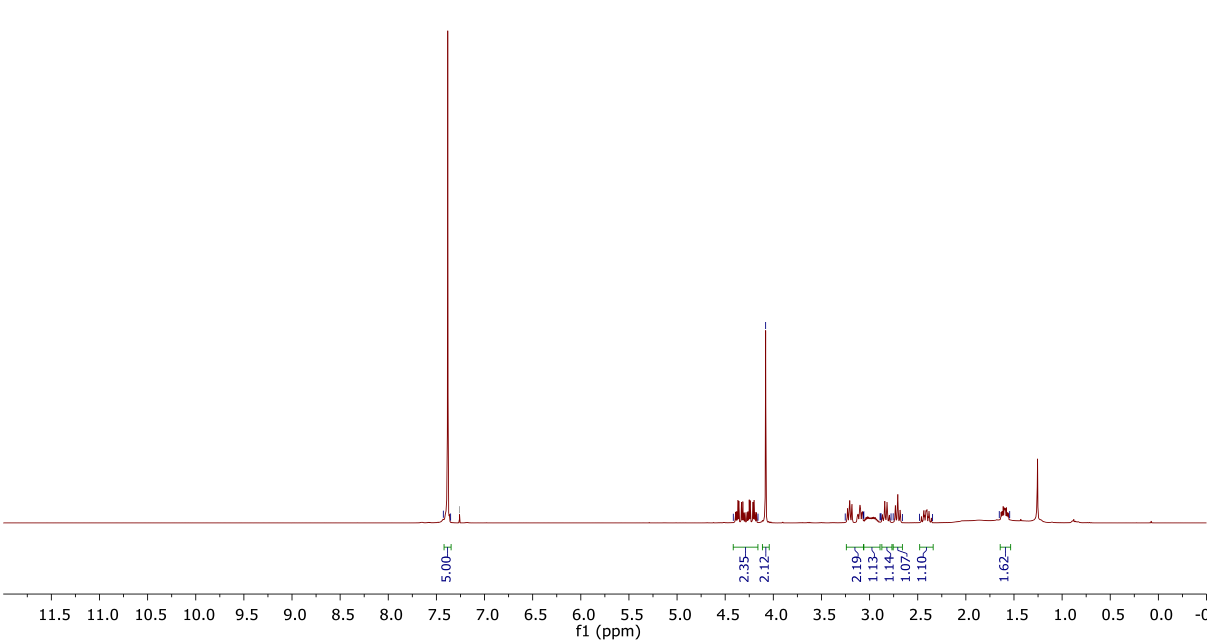

13C NMR (101 MHz, CDCl3)

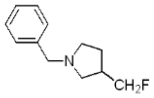

2p

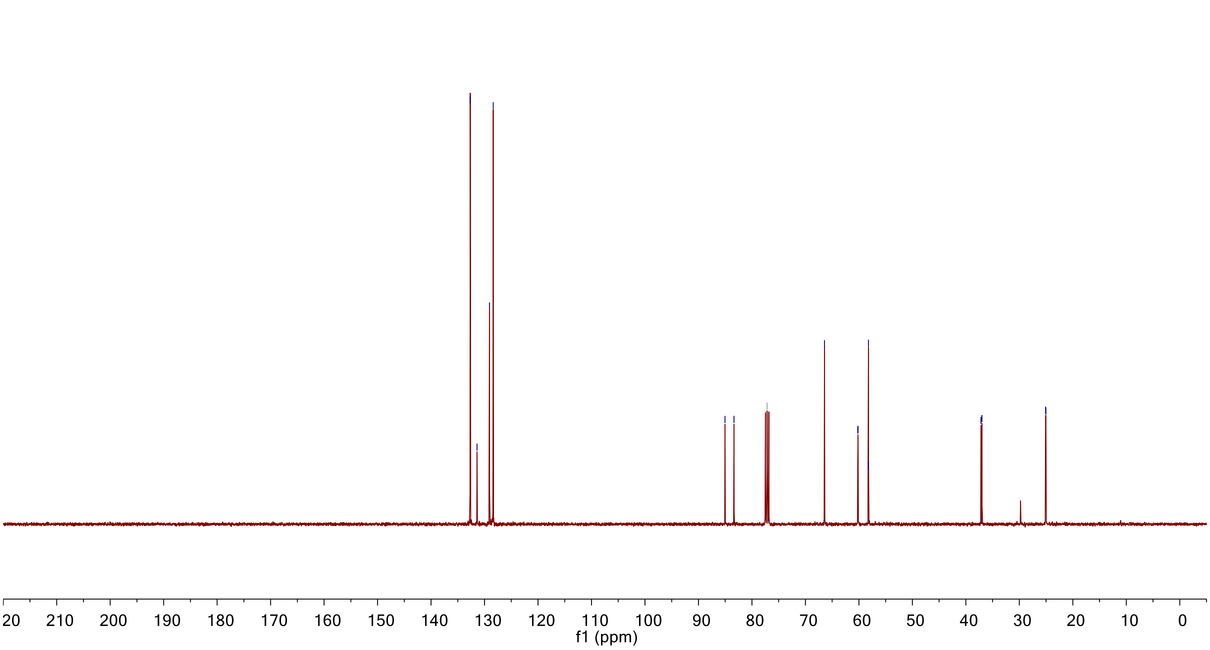

19F NMR (377 MHz, CDCl3)

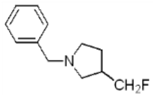

2p

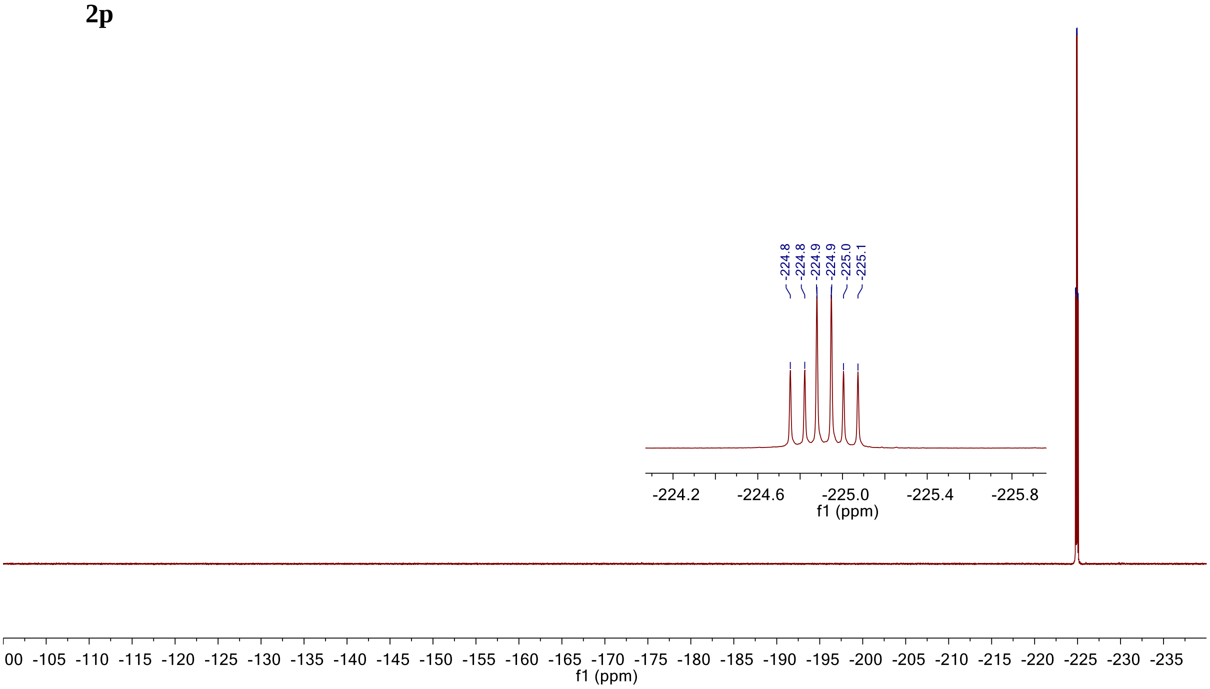

1H NMR (400 MHz, CDCl3)

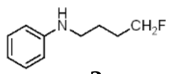

2q

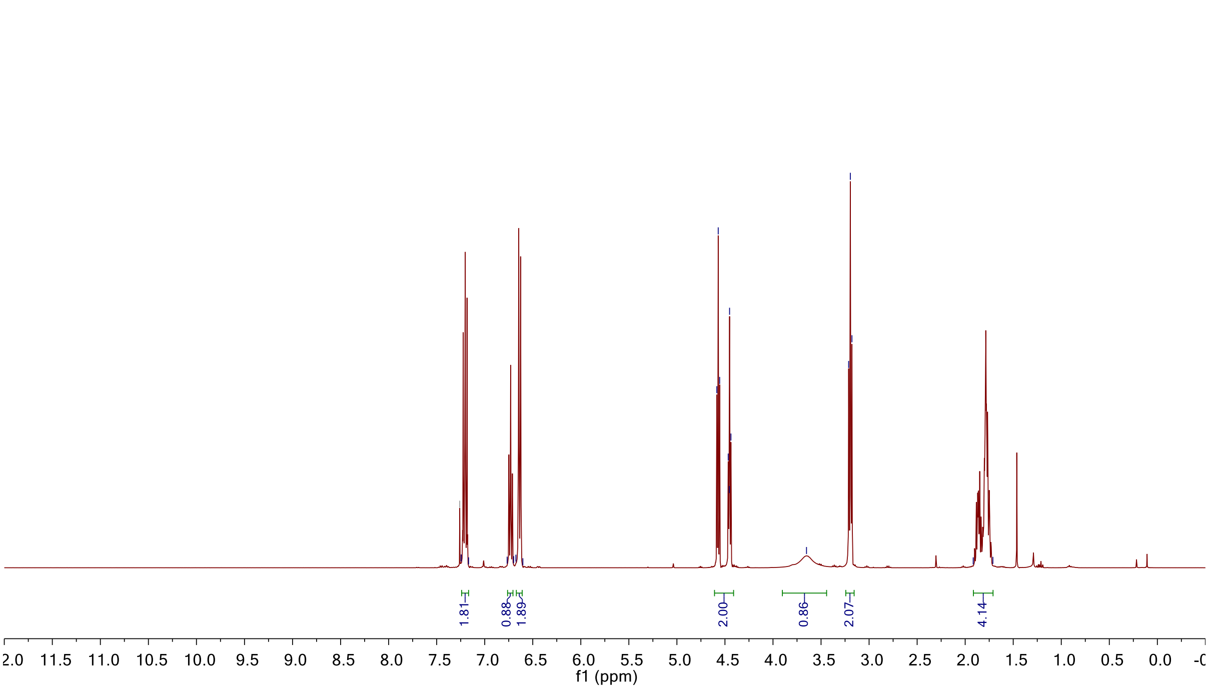

13C NMR (101 MHz, CDCl3)

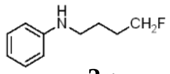

2q

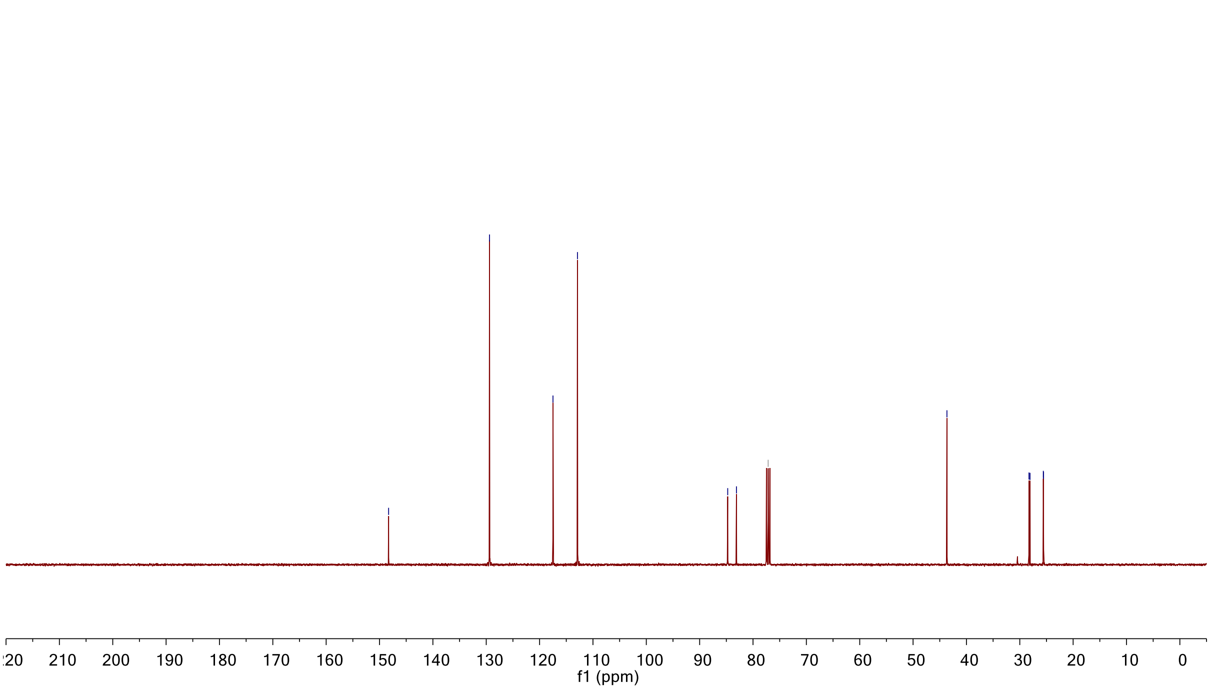

19F NMR (377 MHz, CDCl3)

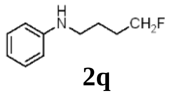

2q

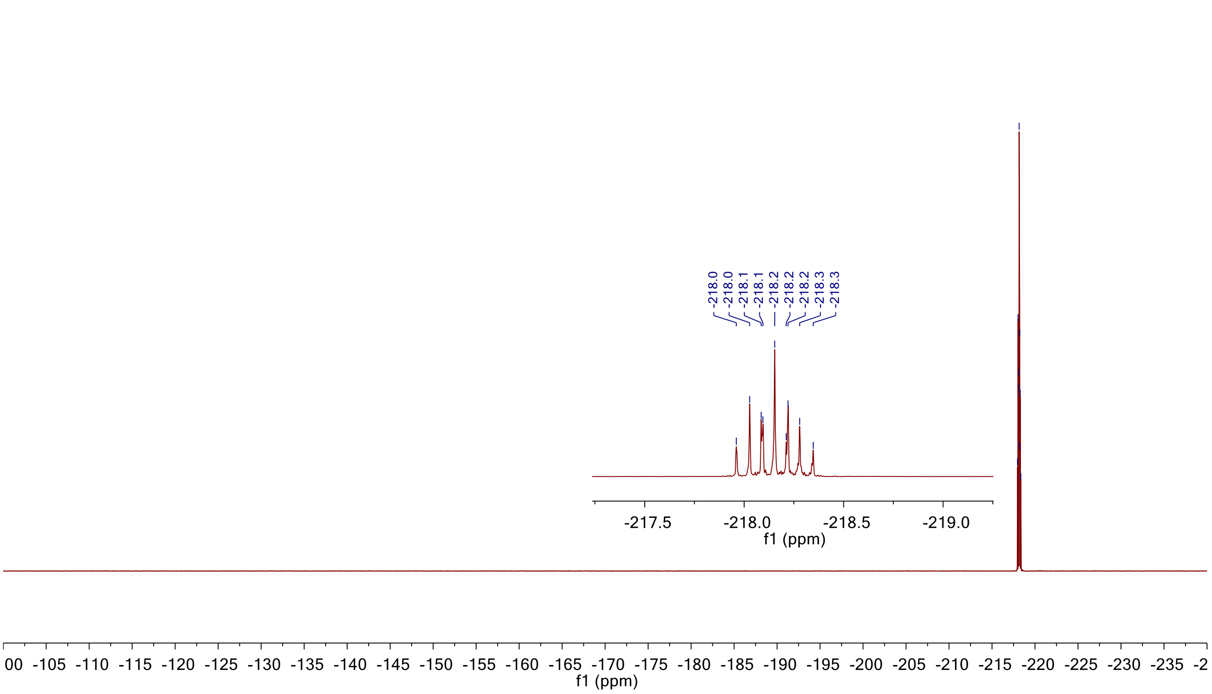

1H NMR (400 MHz, MeOD)

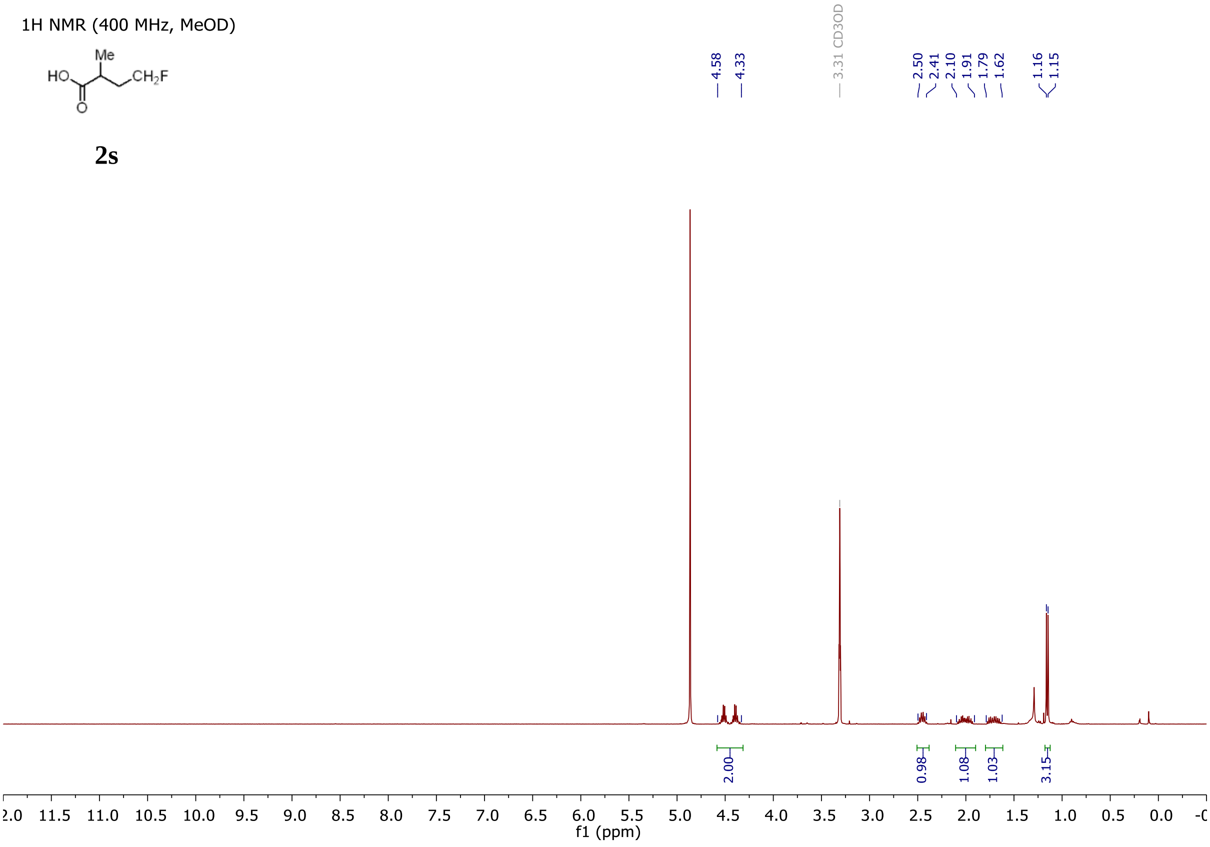

19F NMR (377 MHz, MeOD)

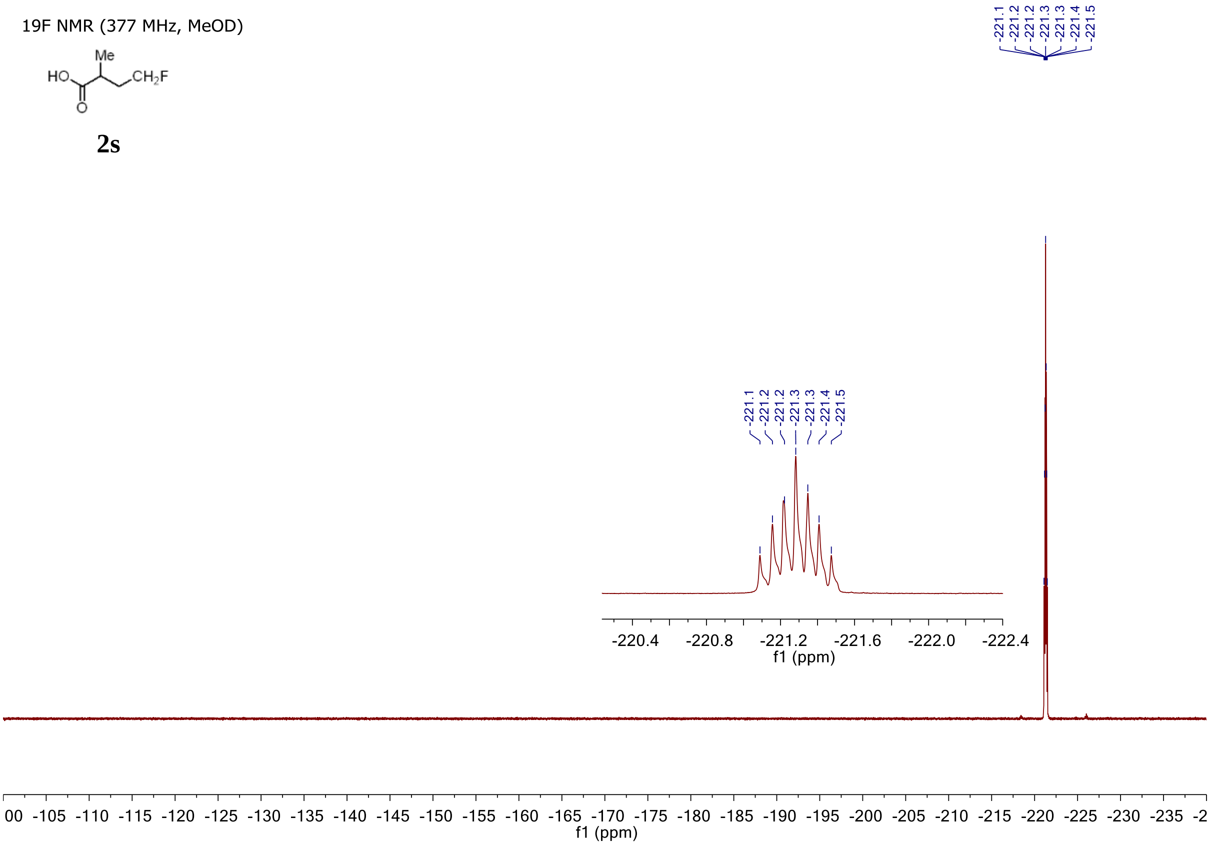

1H NMR (400 MHz, CDCl3)

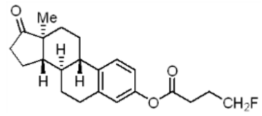

2t

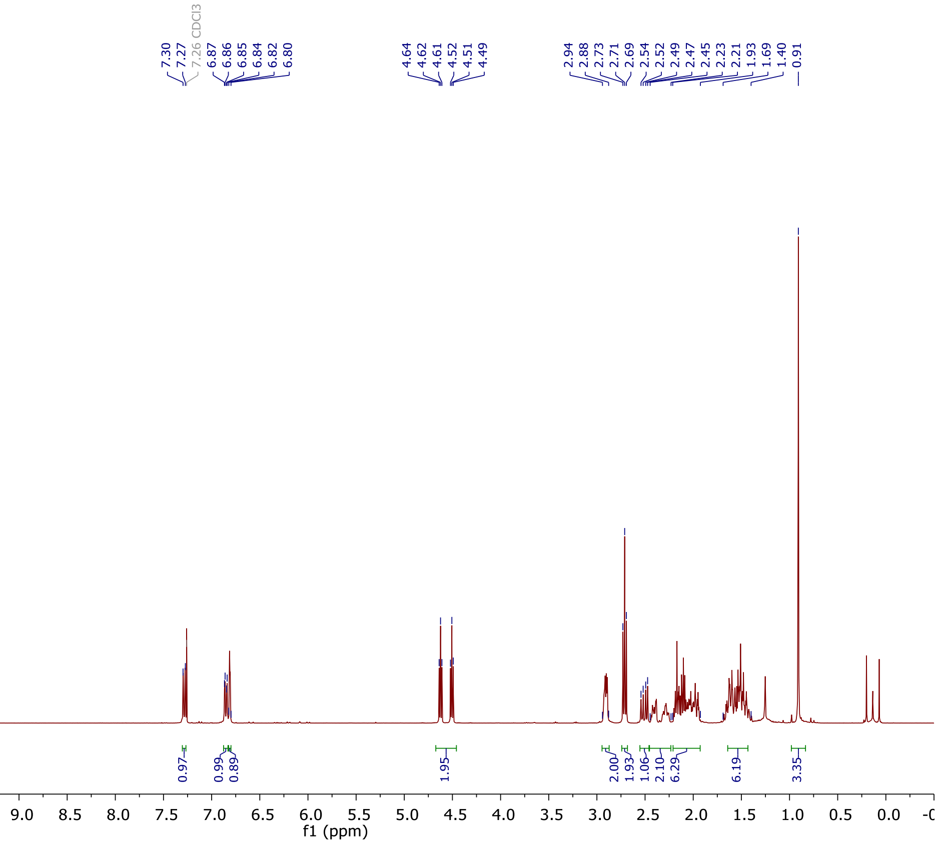

13C NMR (101 MHz, CDCl3)

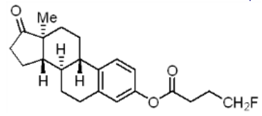

2t

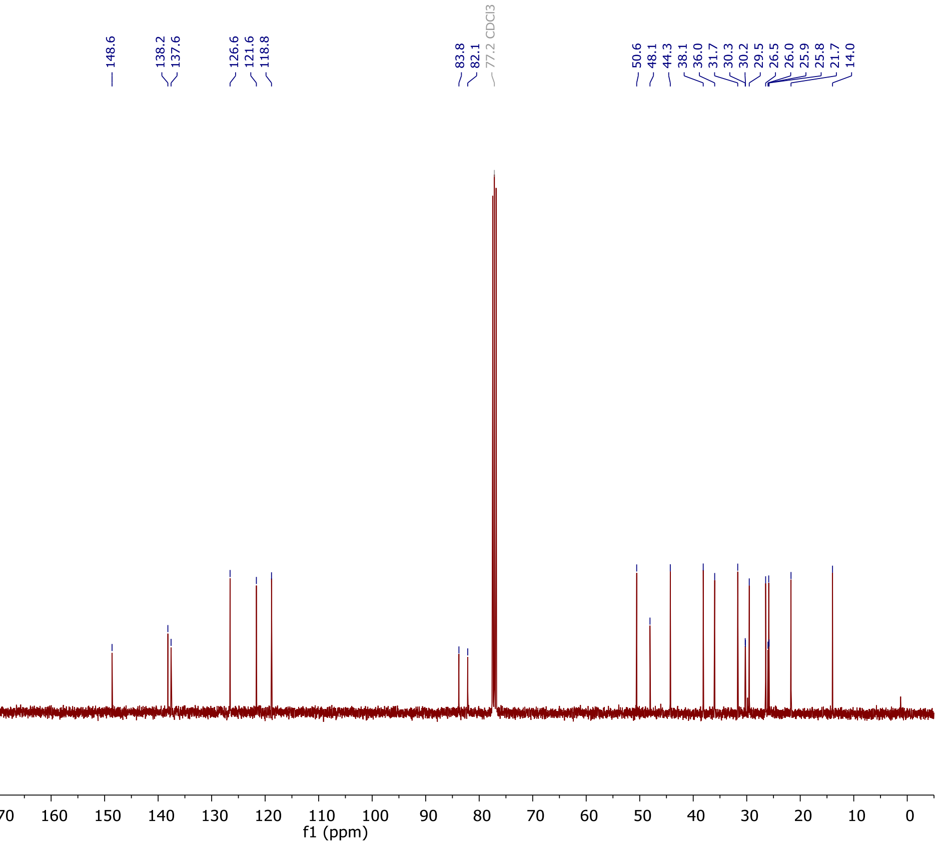

19F NMR (377 MHz, CDCl3)

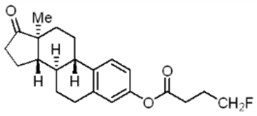

2t

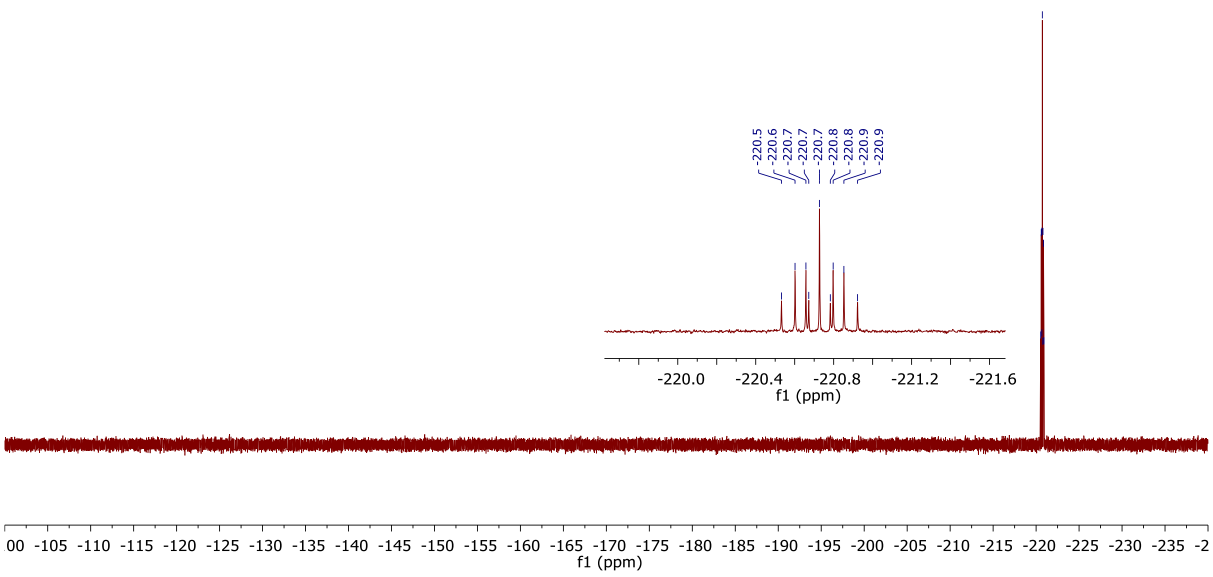

1H NMR (400 MHz, CDCl3)

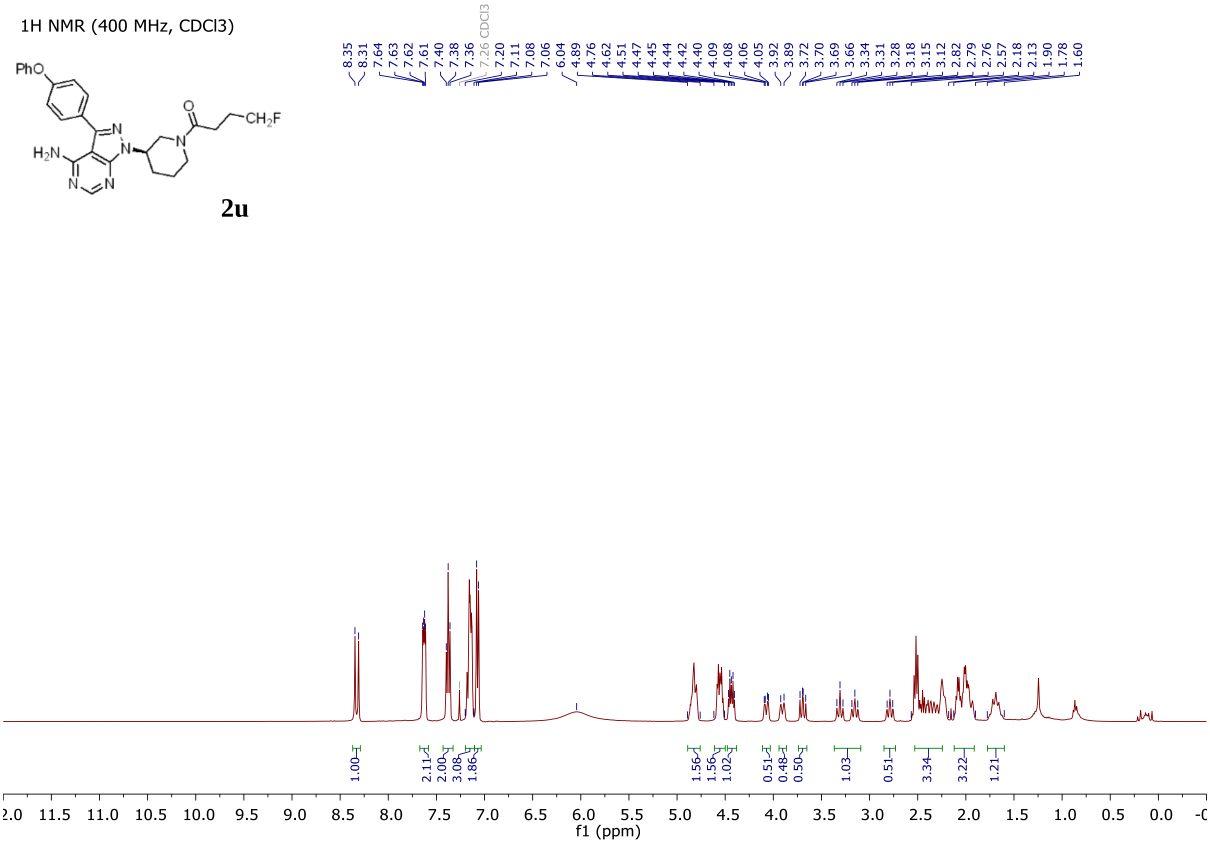

2u

13C NMR (101 MHz, CDCl3)

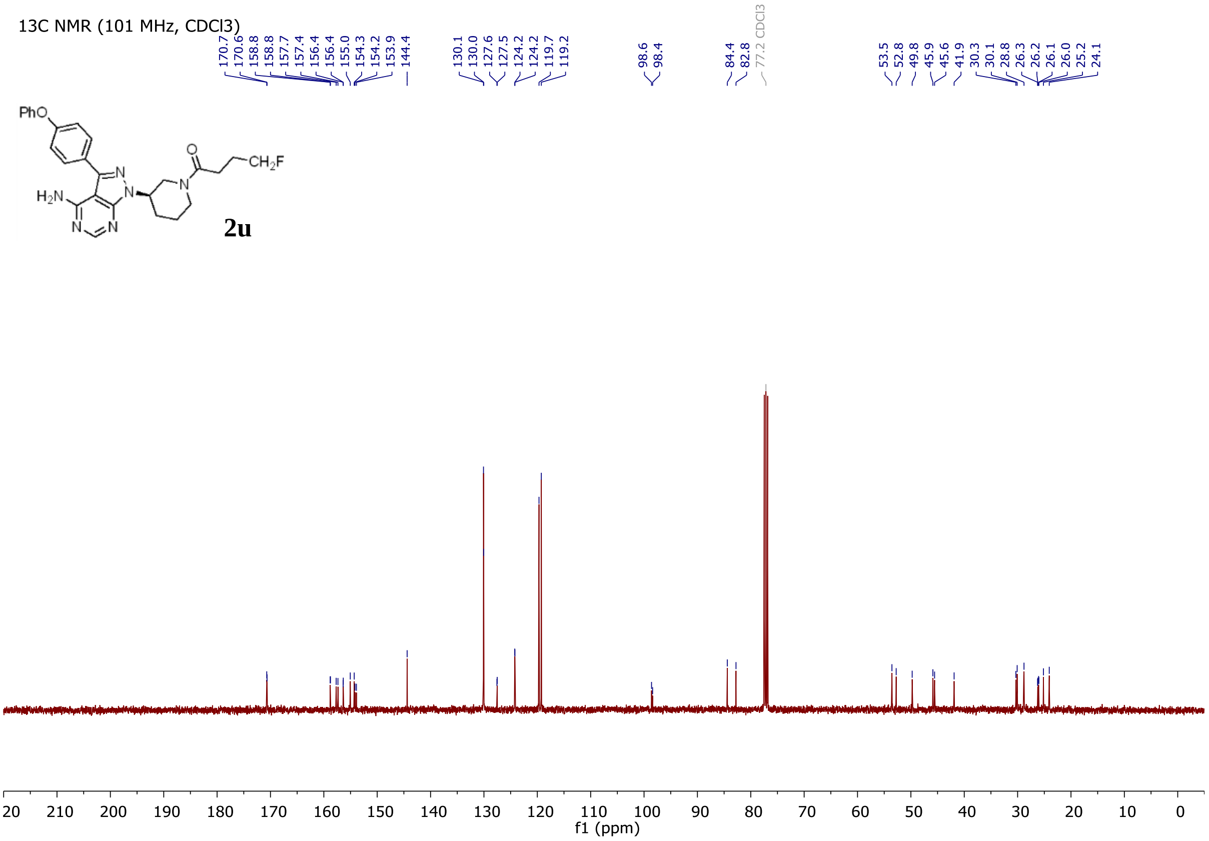

2u

19F NMR (377 MHz, CDCl3)

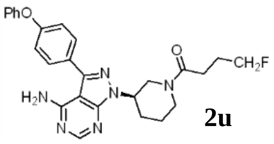

2u

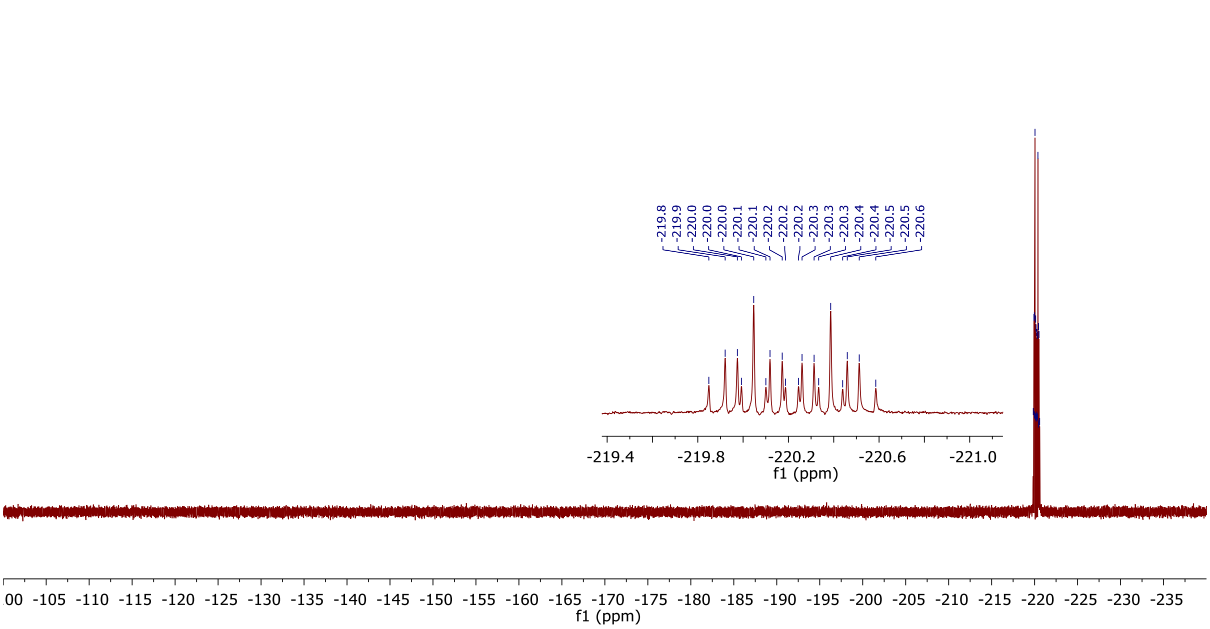

1H NMR (400 MHz, CDCl3)

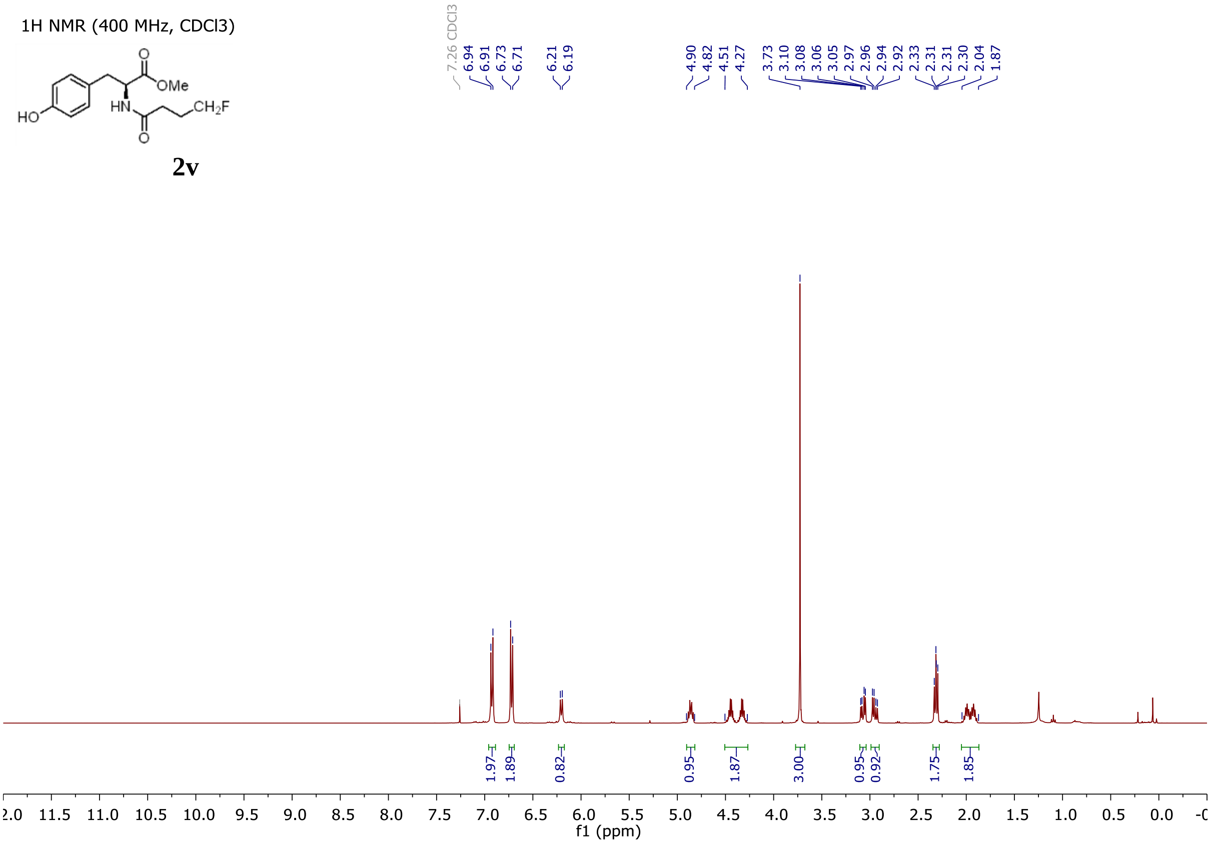

2v

13C NMR (101 MHz, CDCl3)

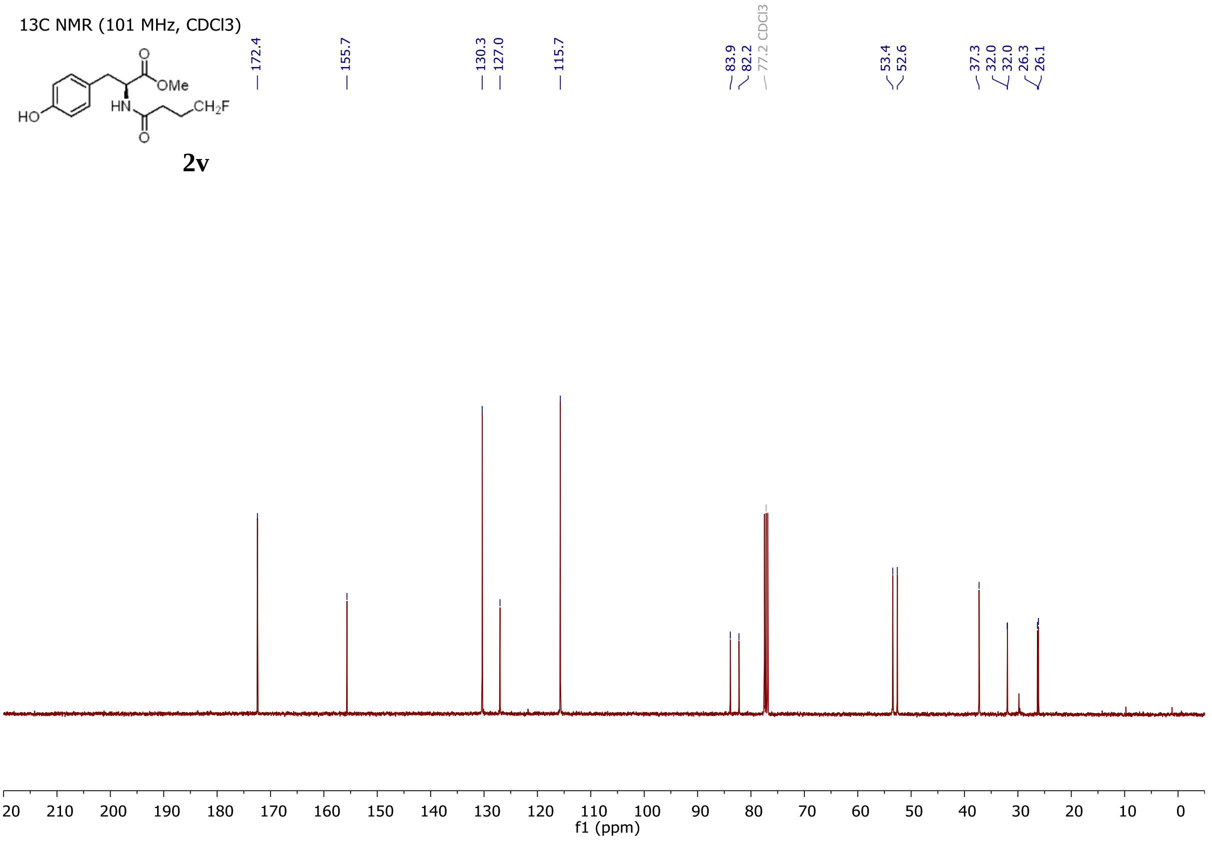

2v

19F NMR (377 MHz, CDCl3)

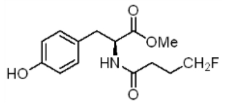

2v

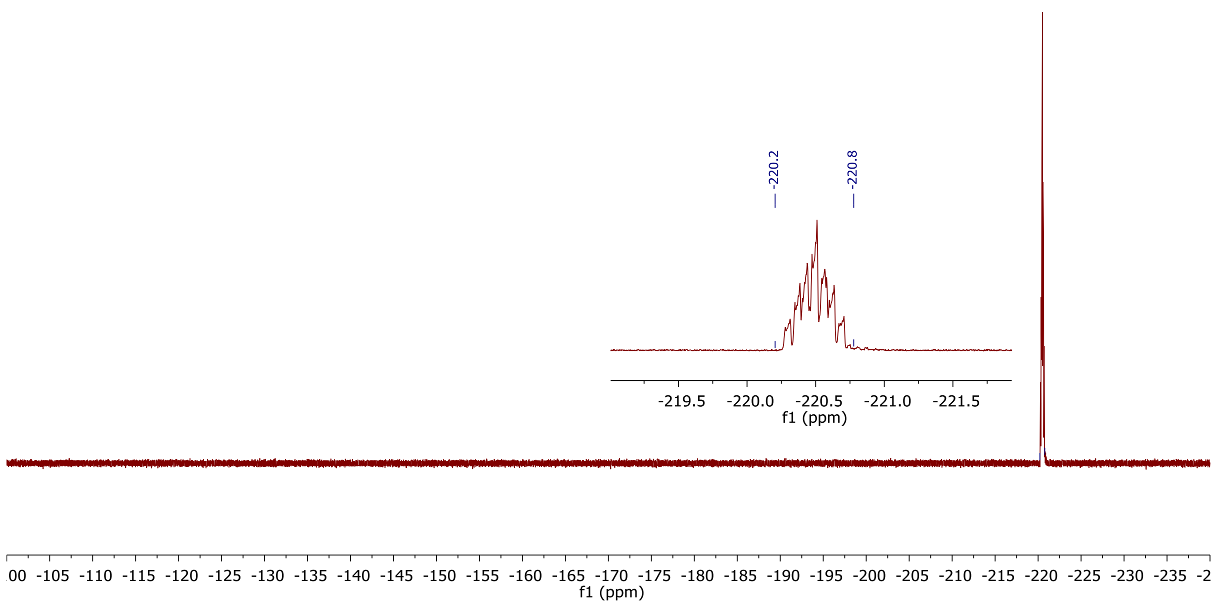

1H NMR (400 MHz, CDCl3)

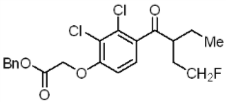

2w

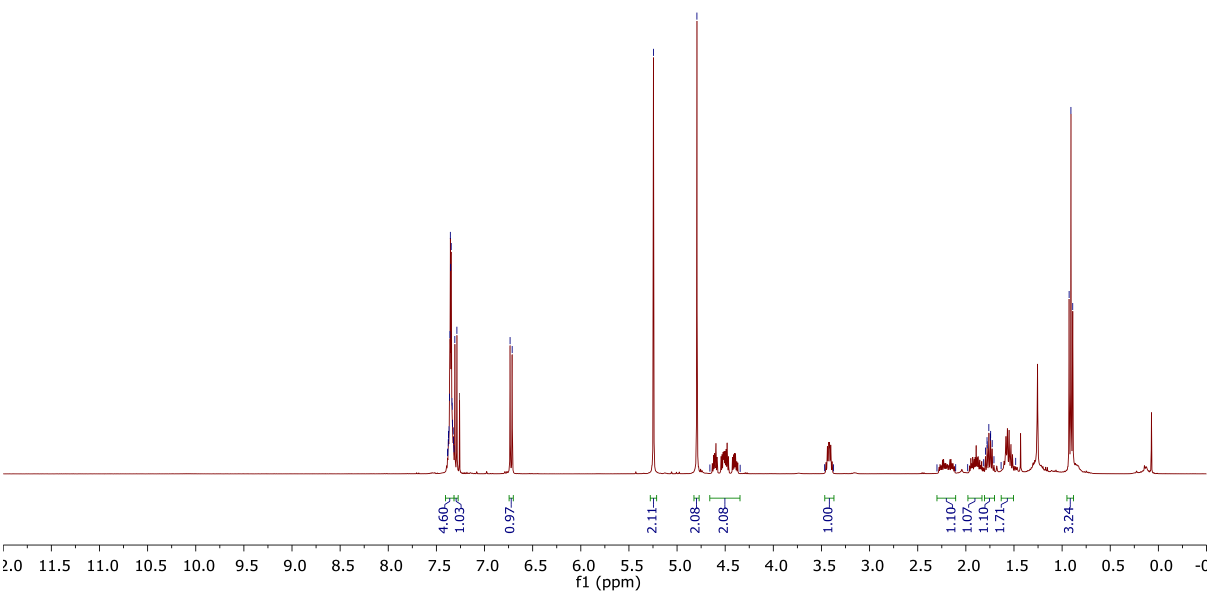

13C NMR (101 MHz, CDCl3)

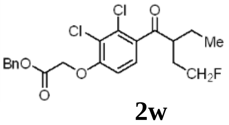

2w

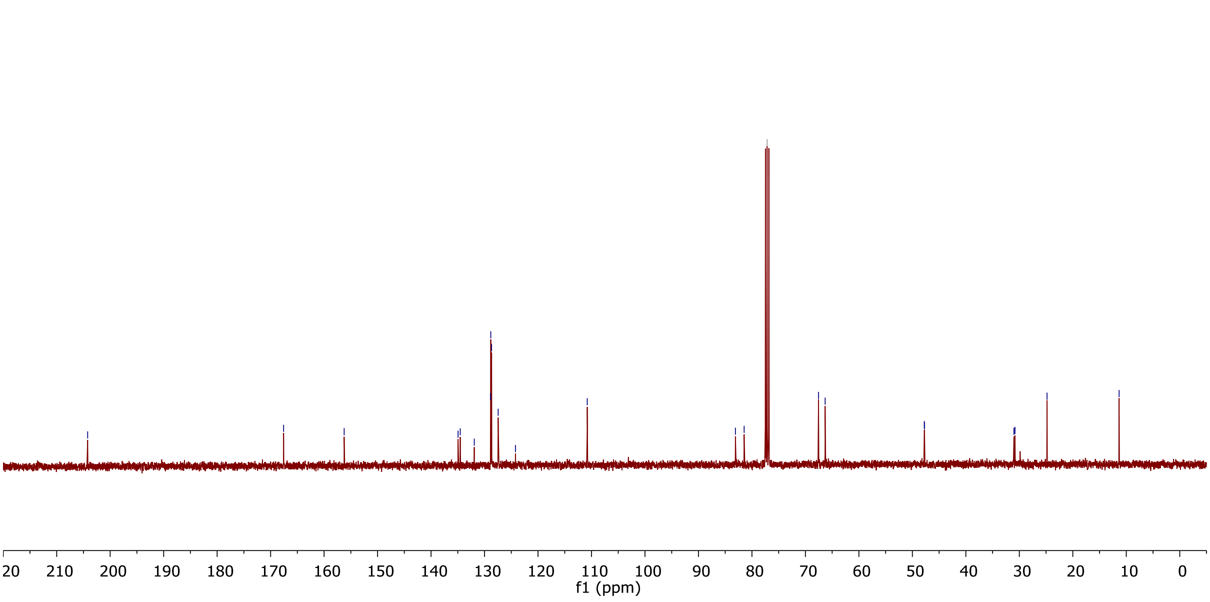

19F NMR (377 MHz, CDCl3)

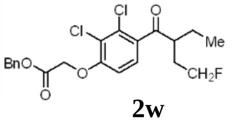

2w

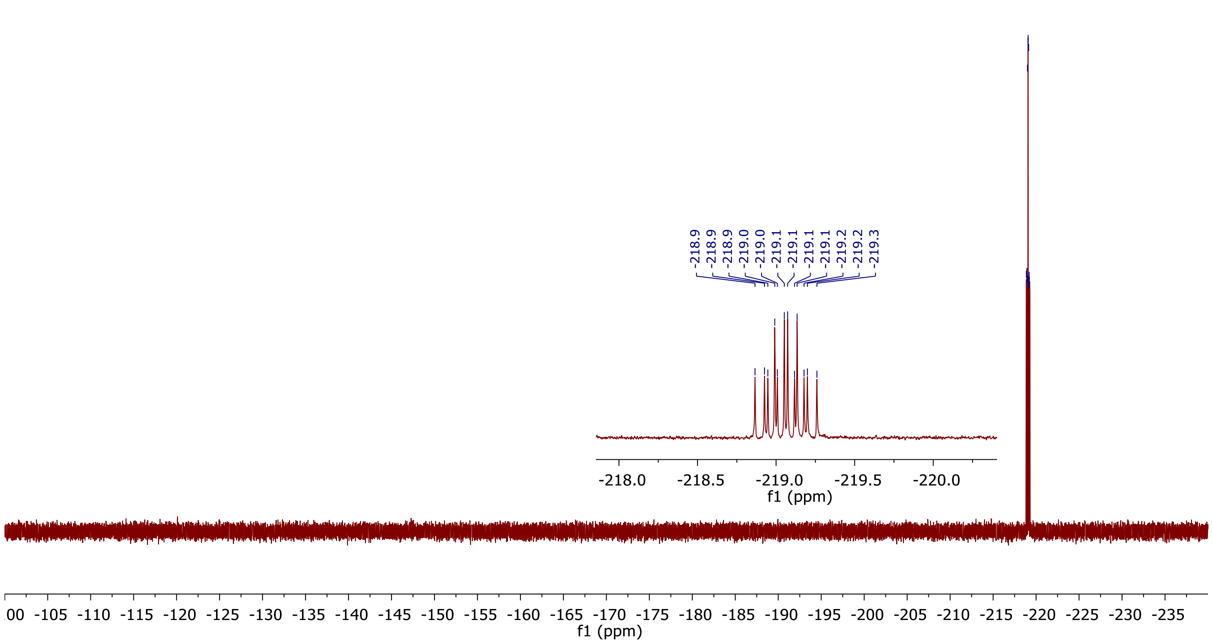

1H NMR (400 MHz, CDCl3)

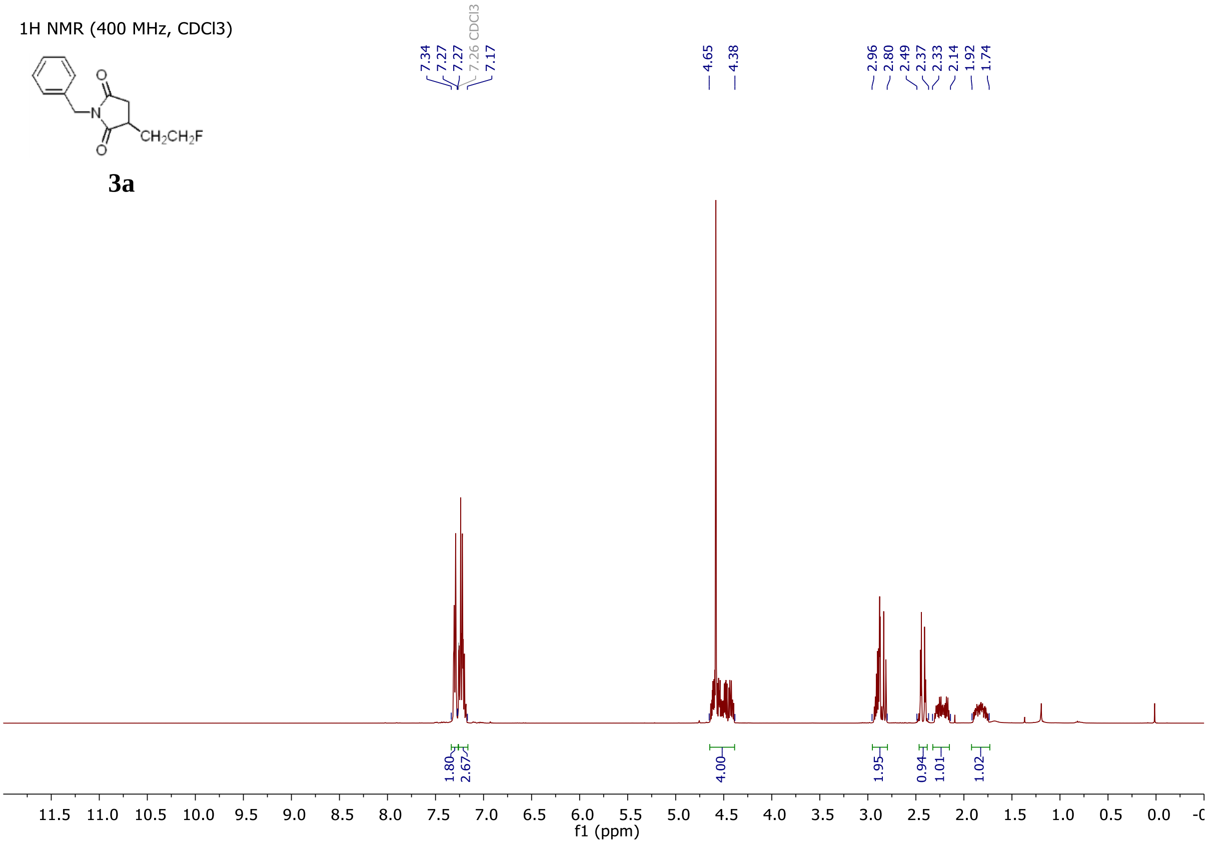

13C NMR (101 MHz, CDCl3)

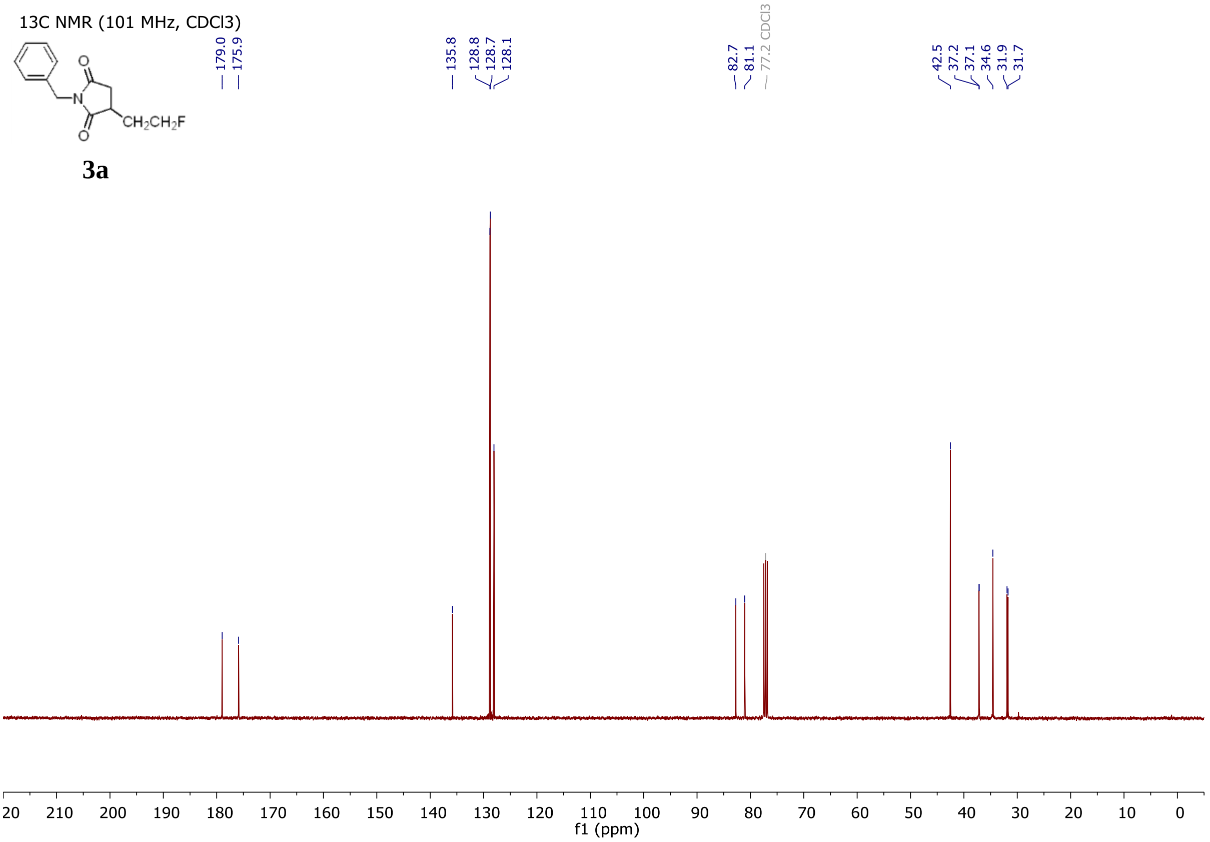

19F NMR (377 MHz, CDCl3)

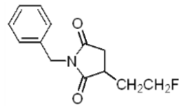

3a

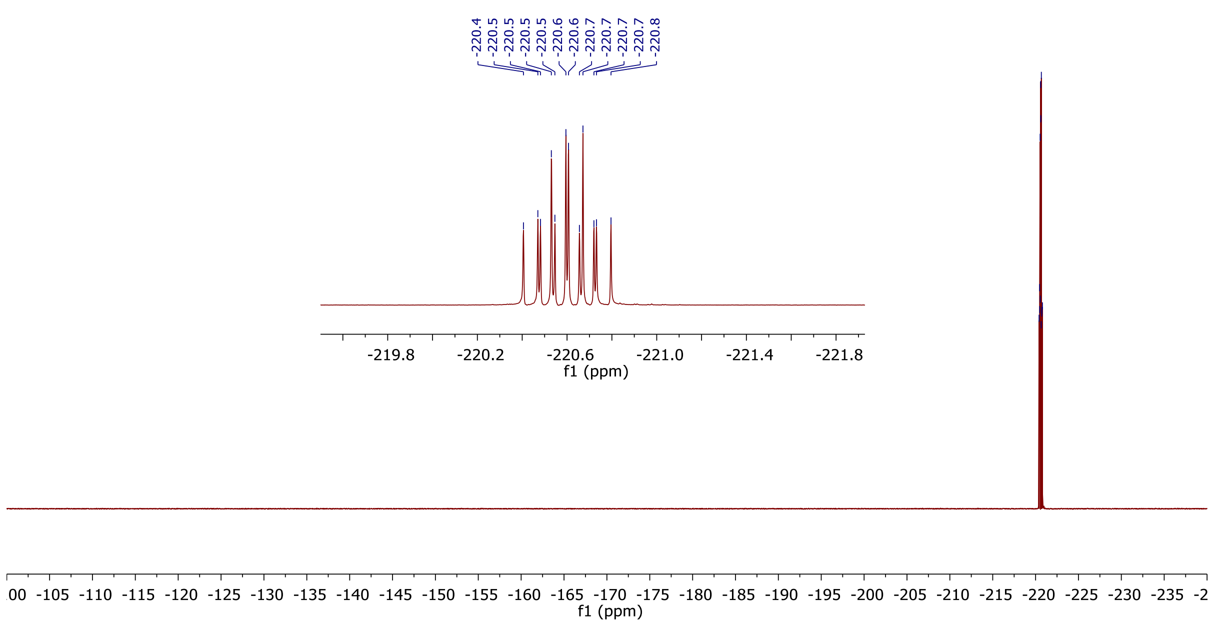

1H NMR (400 MHz, CDCl3)

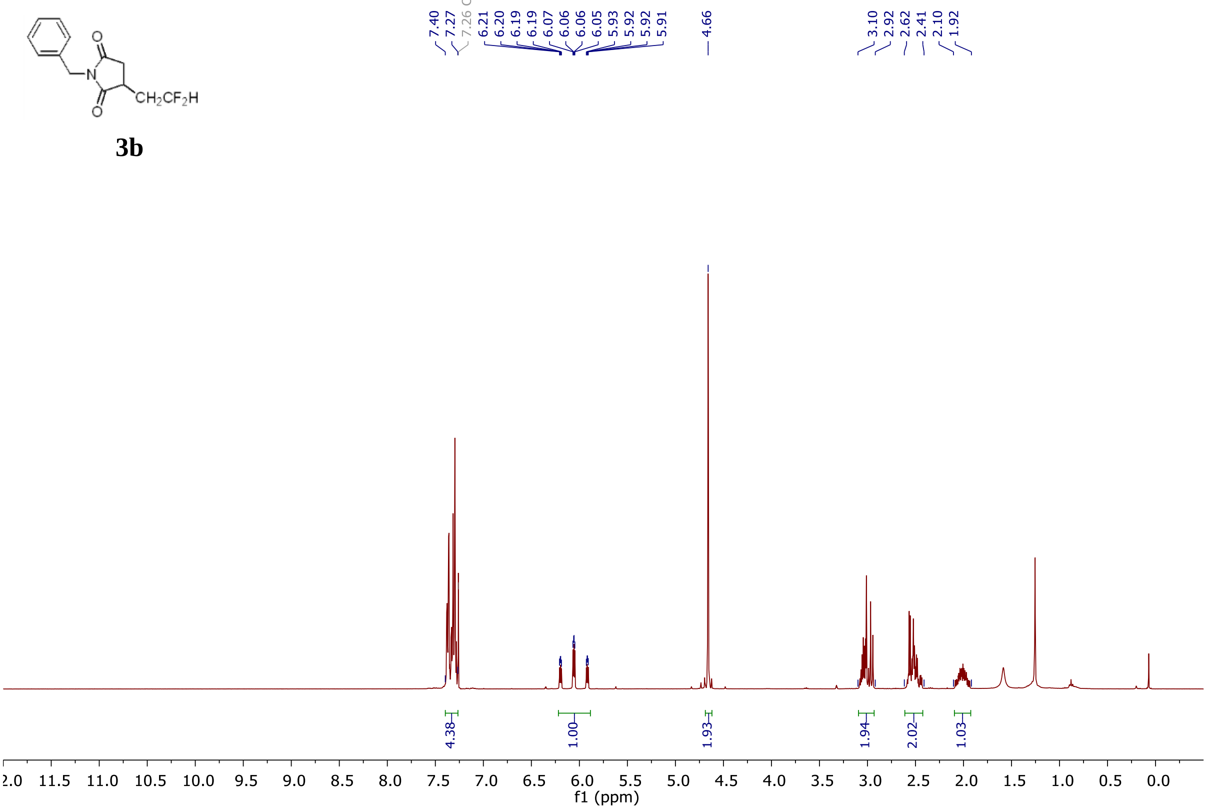

13C NMR (101 MHz, CDCl3)

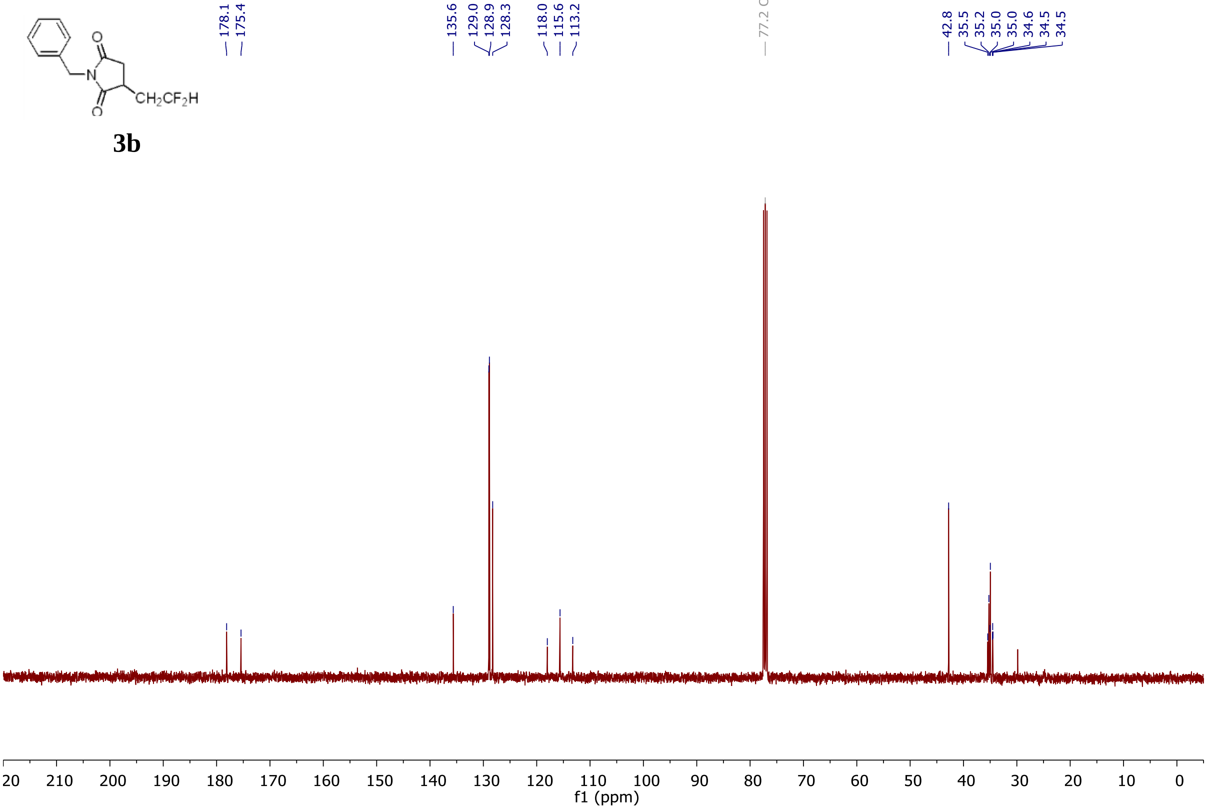

19F NMR (377 MHz, CDCl3)

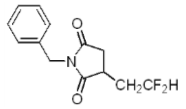

3b

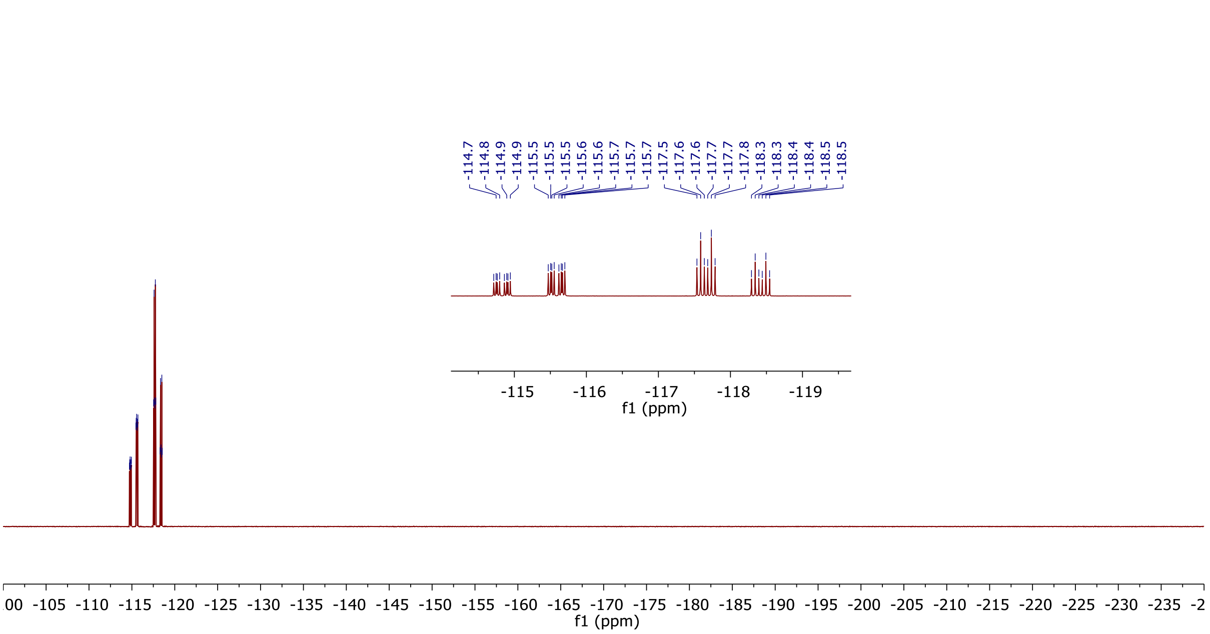

1H NMR (400 MHz, CDCl3)

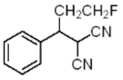

3d

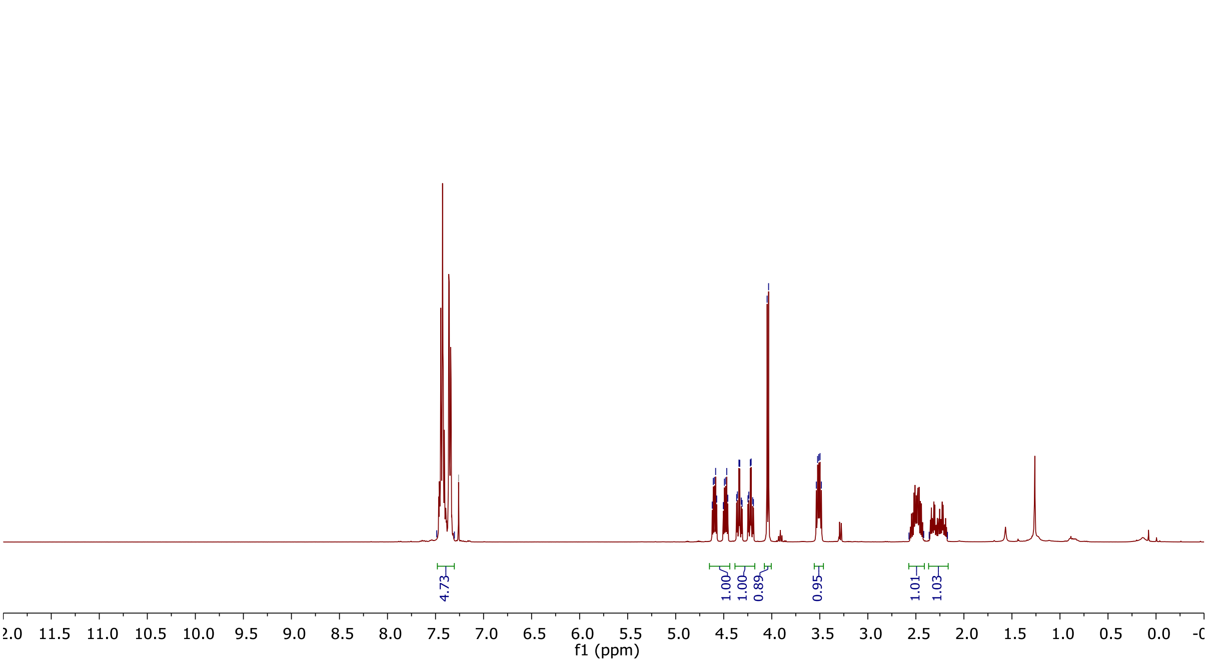

13C NMR (101 MHz, CDCl3)

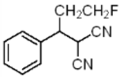

3d

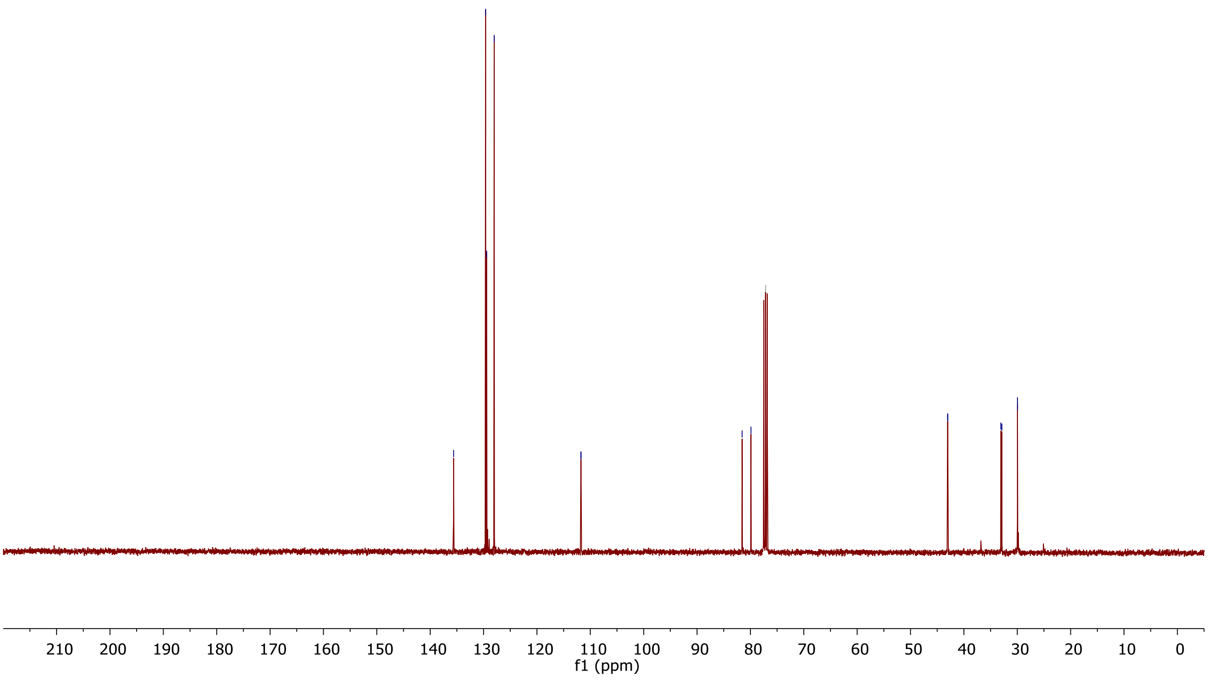

19F NMR (377 MHz, CDCl3)

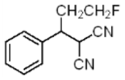

3d

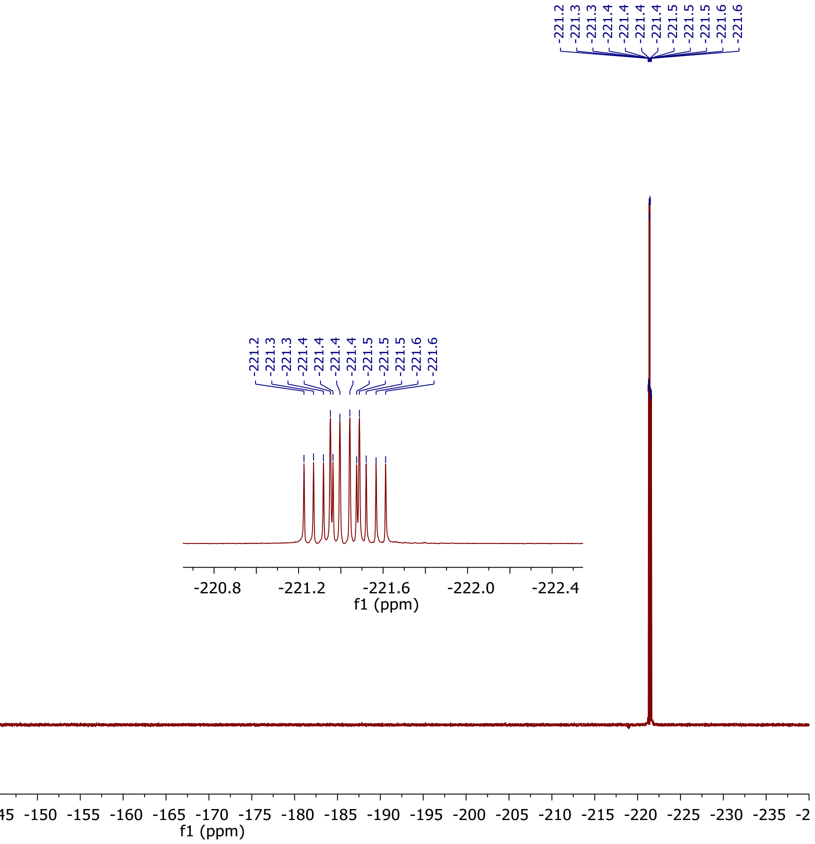

1H NMR (400 MHz, CDCl3)

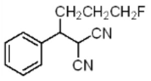

3e

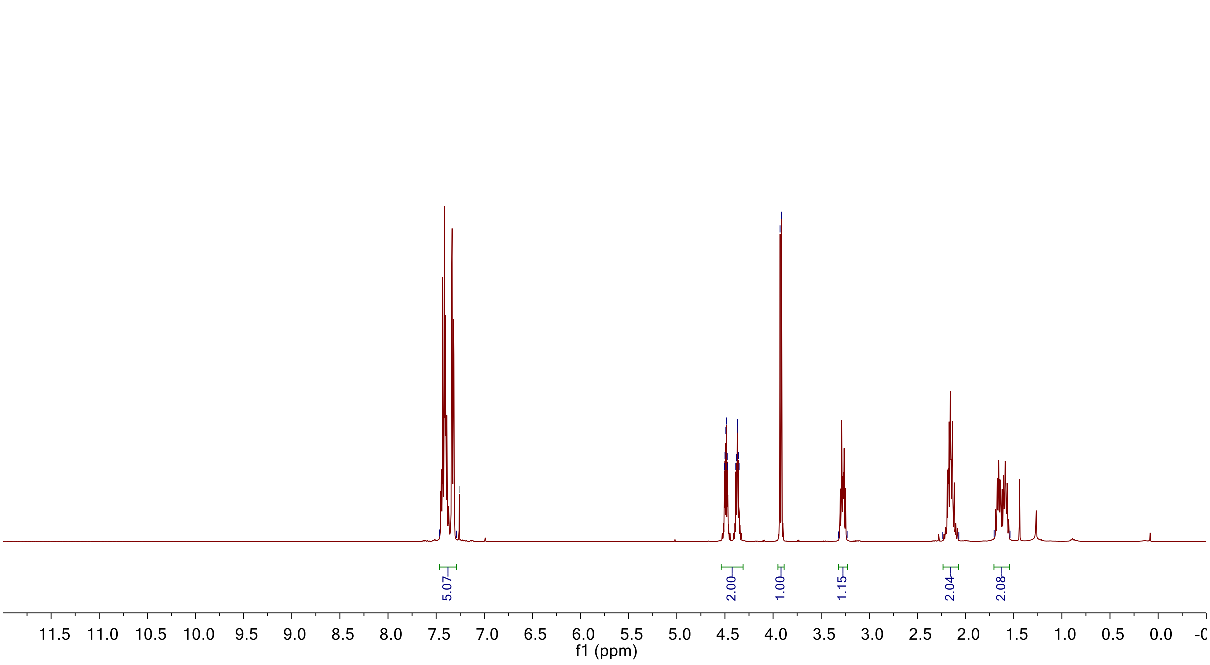

13C NMR (101 MHz, CDCl3)

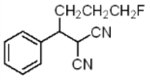

3e

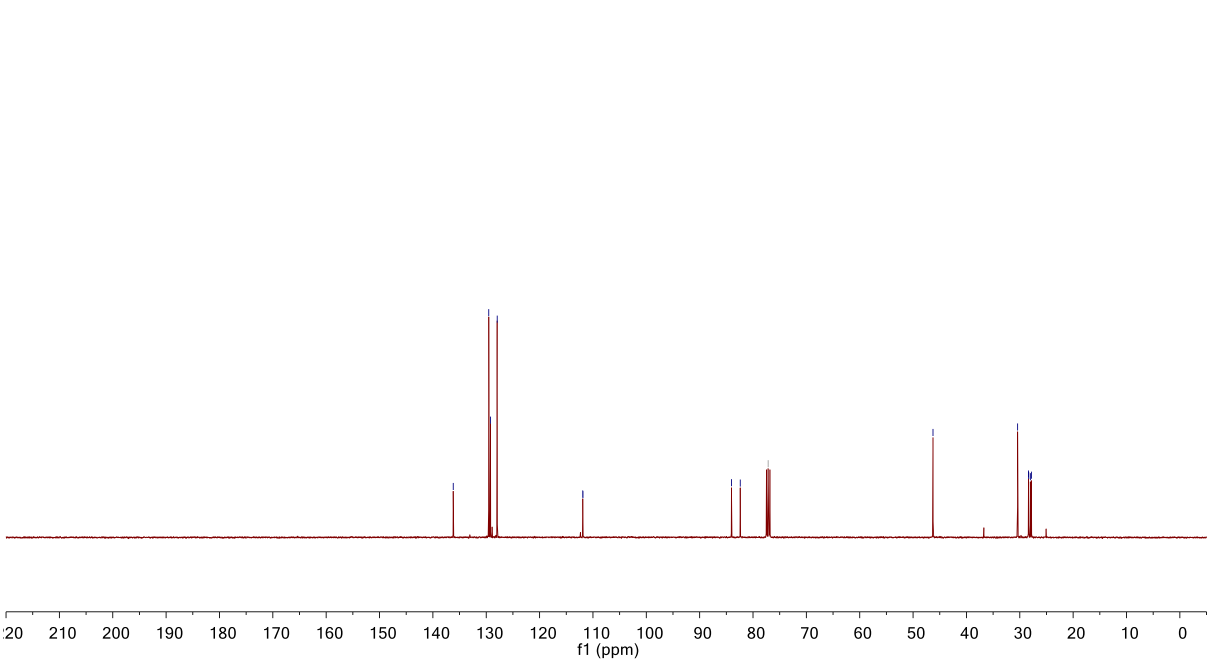

19F NMR (377 MHz, CDCl3)

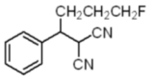

3e

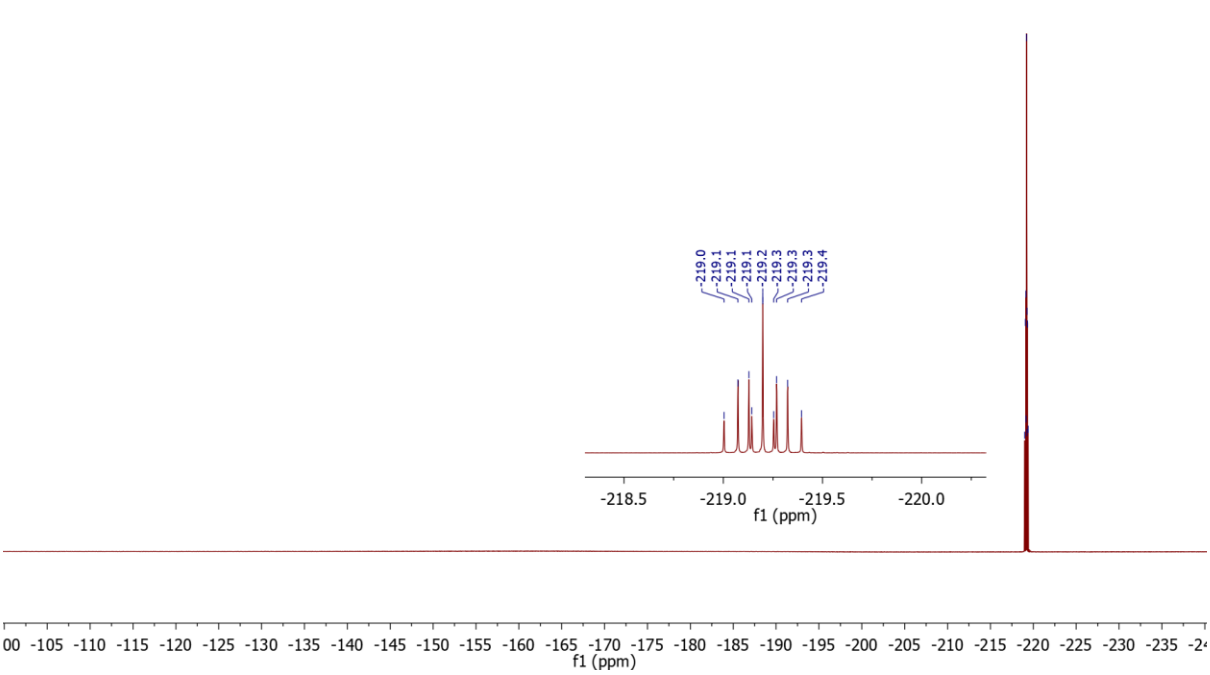

1H NMR (400 MHz, CDCl3)

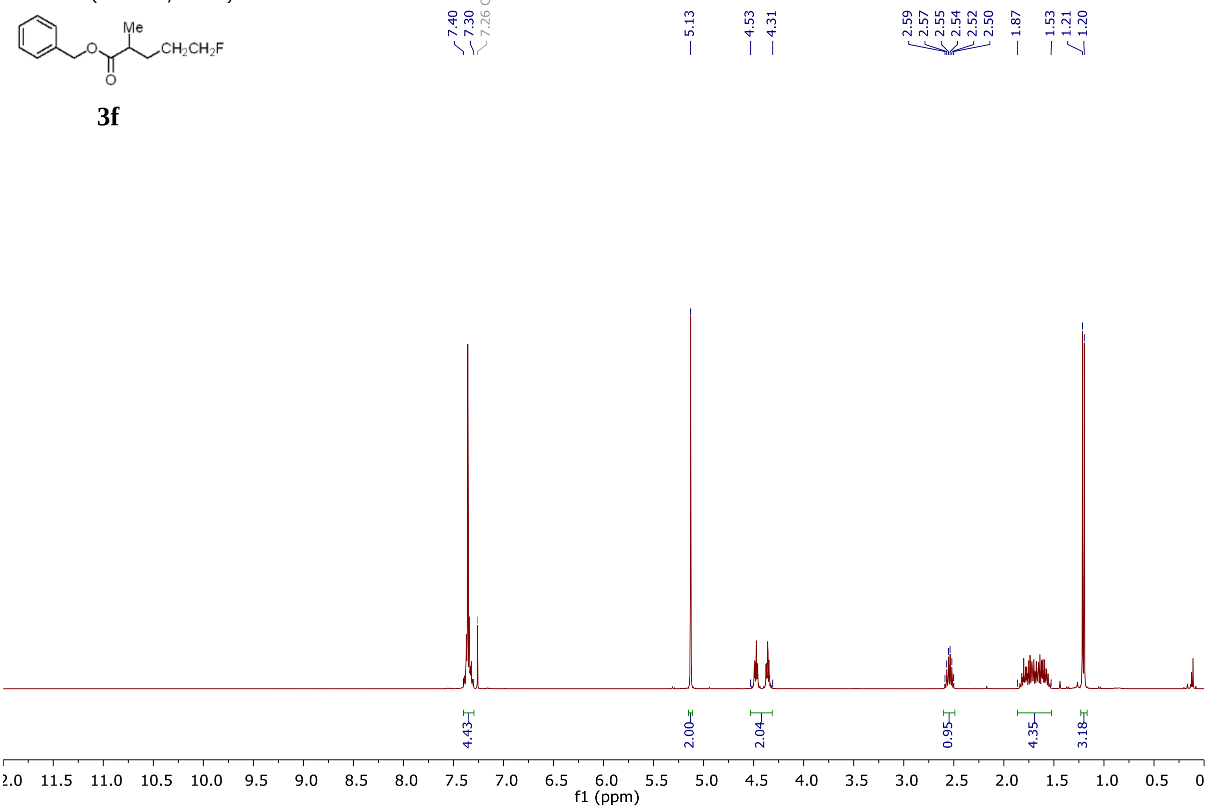

3f

13C NMR (101 MHz, CDCl3)

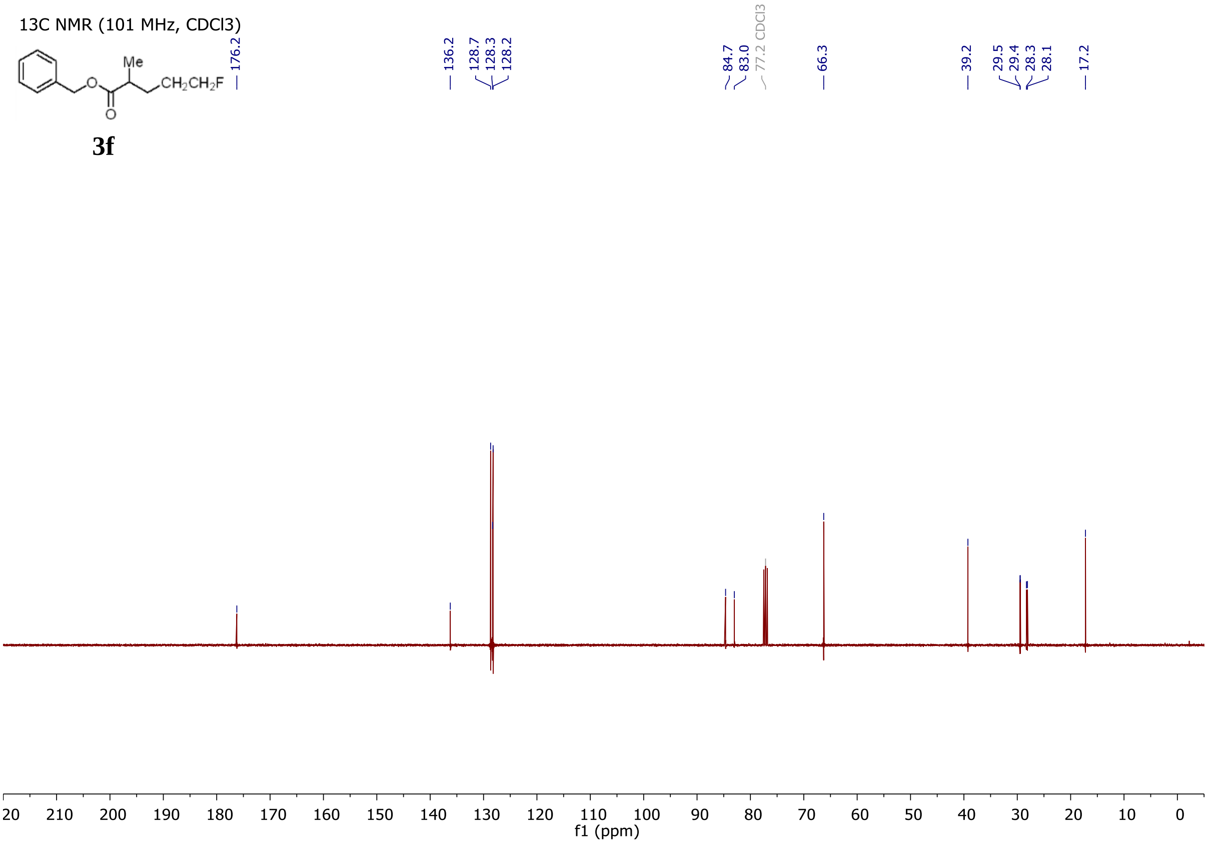

3f

19F NMR (377 MHz, CDCl3)

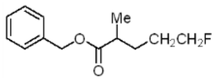

3f

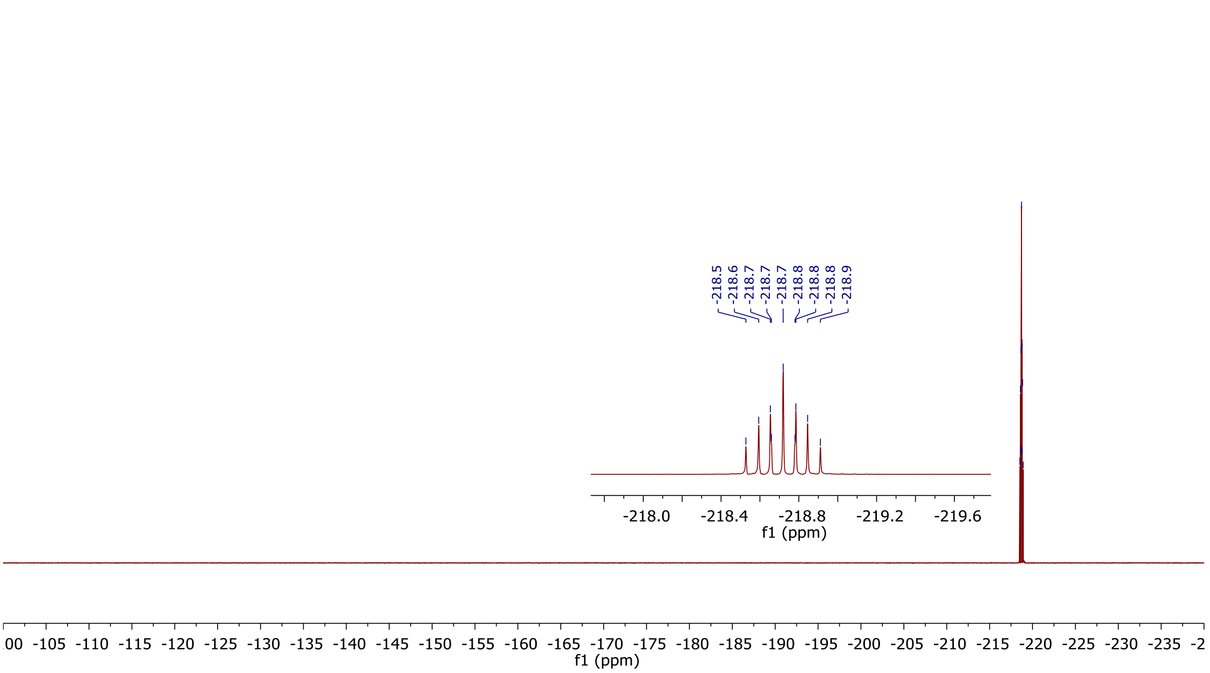

1H NMR (400 MHz, CDCl3)

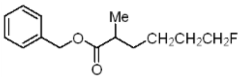

3g

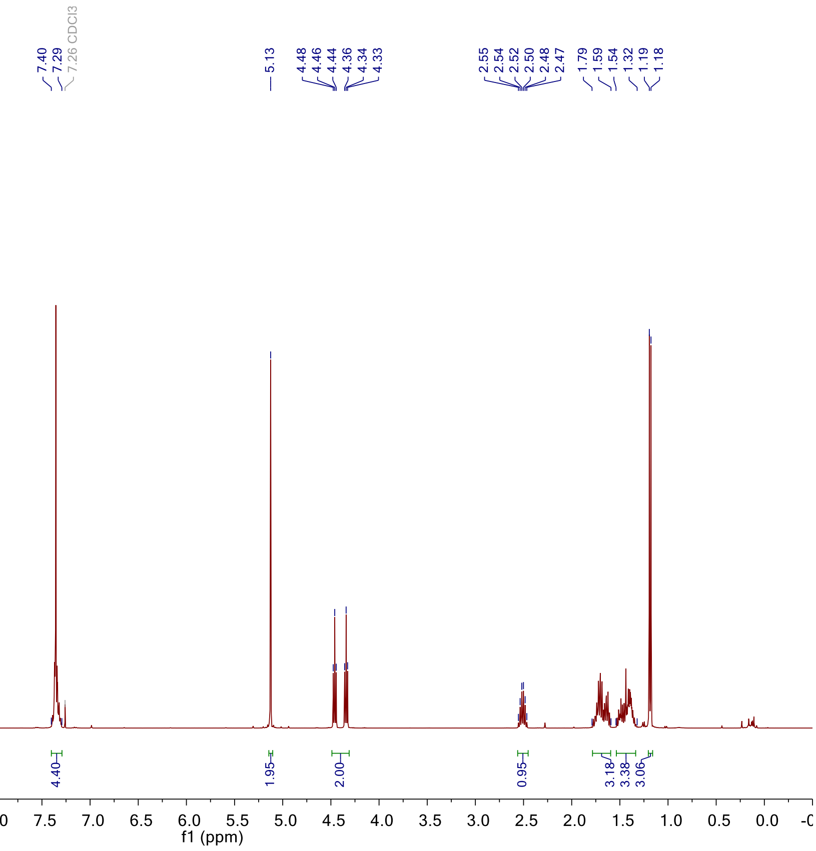

13C NMR (101 MHz, CDCl3)

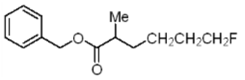

3g

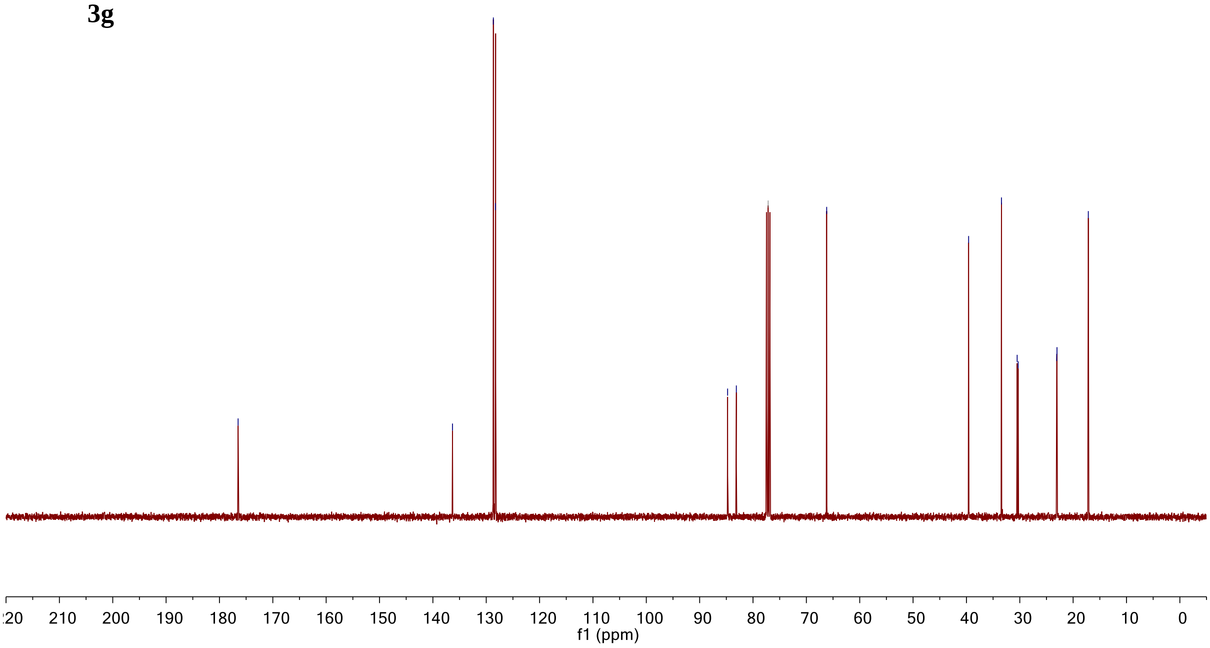

19F NMR (377 MHz, CDCl3)

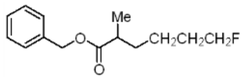

3g

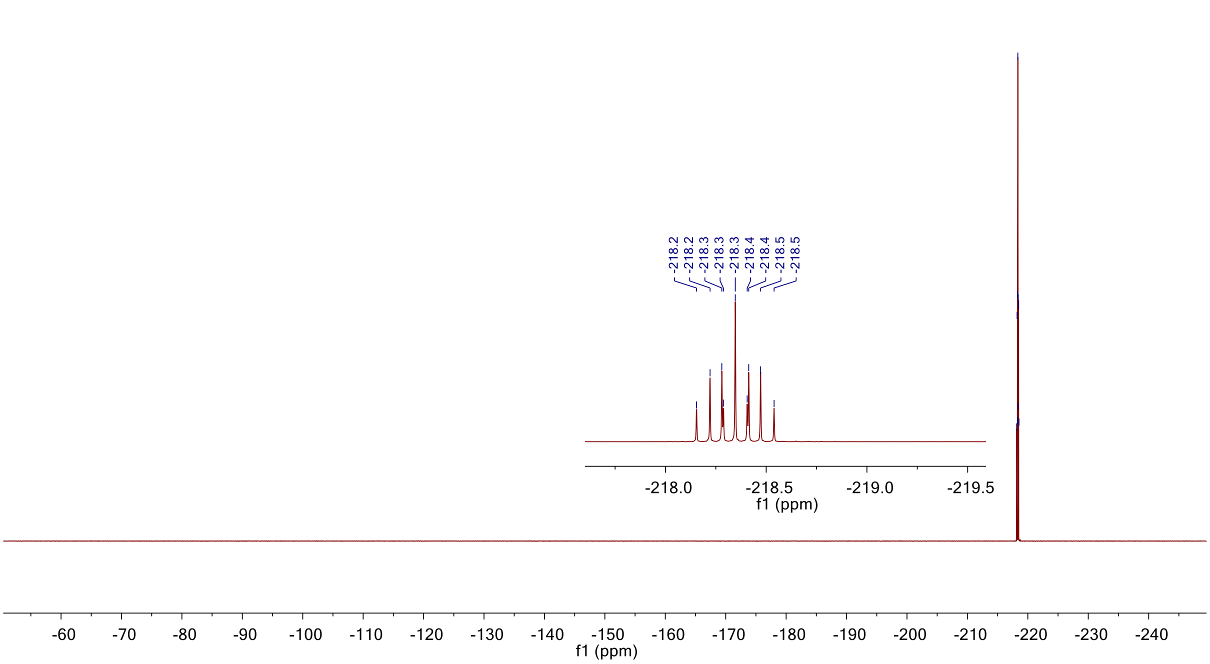

1H NMR (400 MHz, CDCl3)

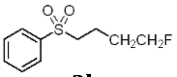

3h

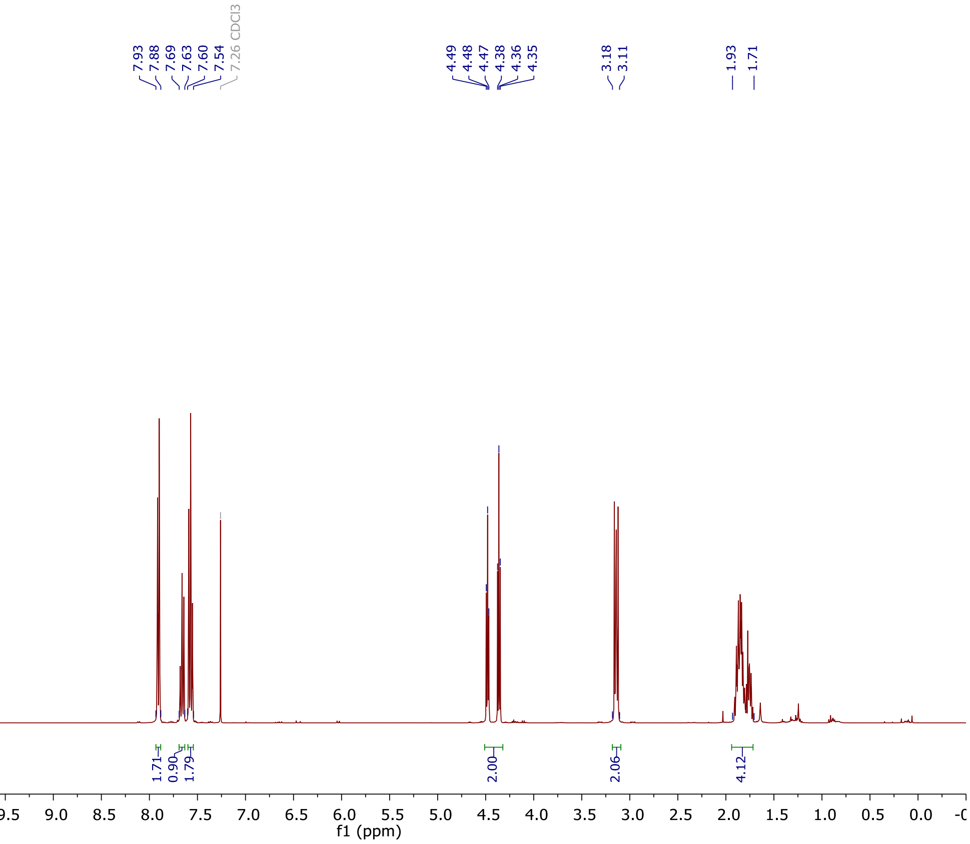

13C NMR (101 MHz, CDCl3)

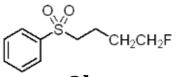

3h

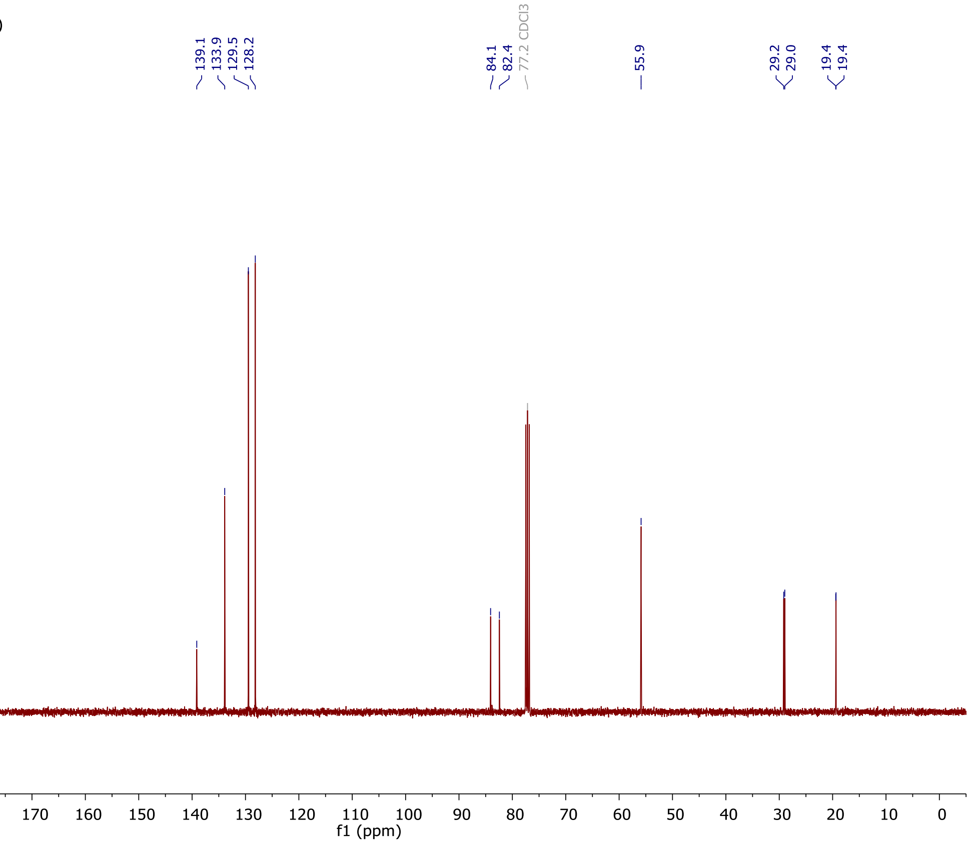

19F NMR (377 MHz, CDCl3)

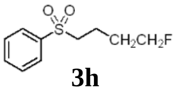

3h

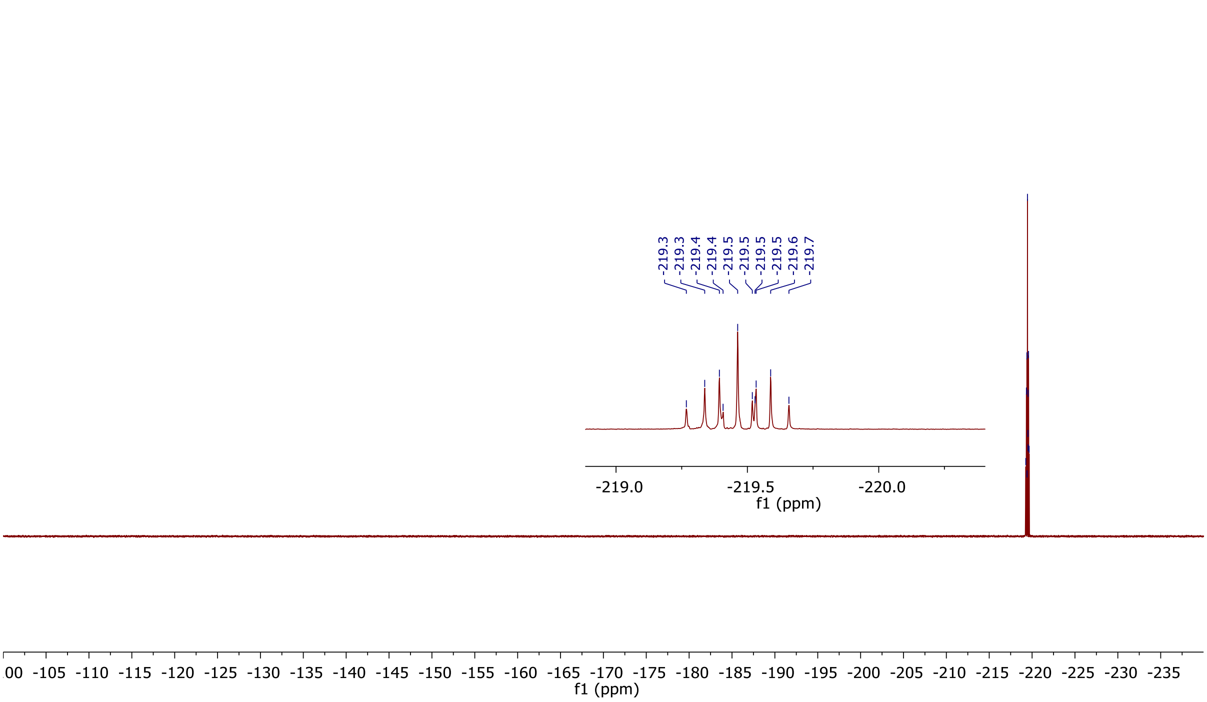

1H NMR (400 MHz, CDCl3)

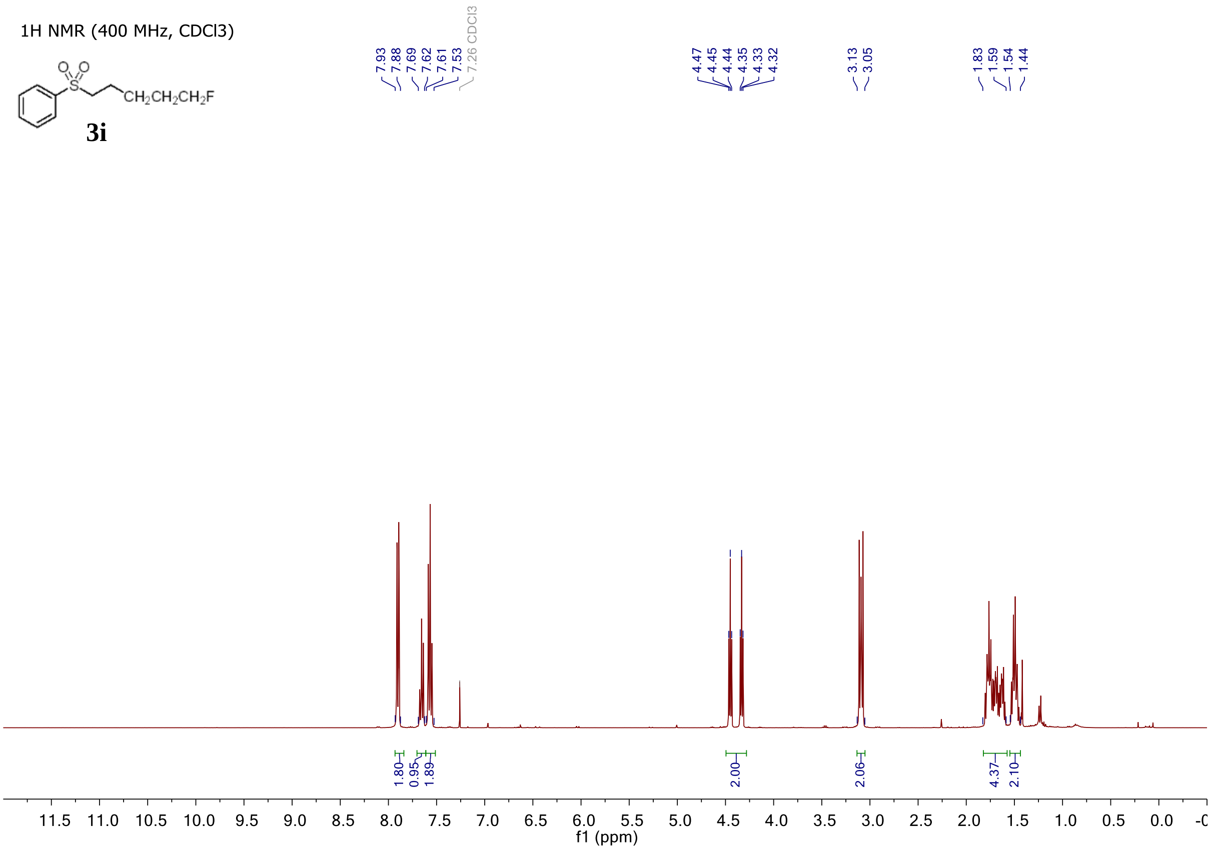

13C NMR (101 MHz, CDCl3)

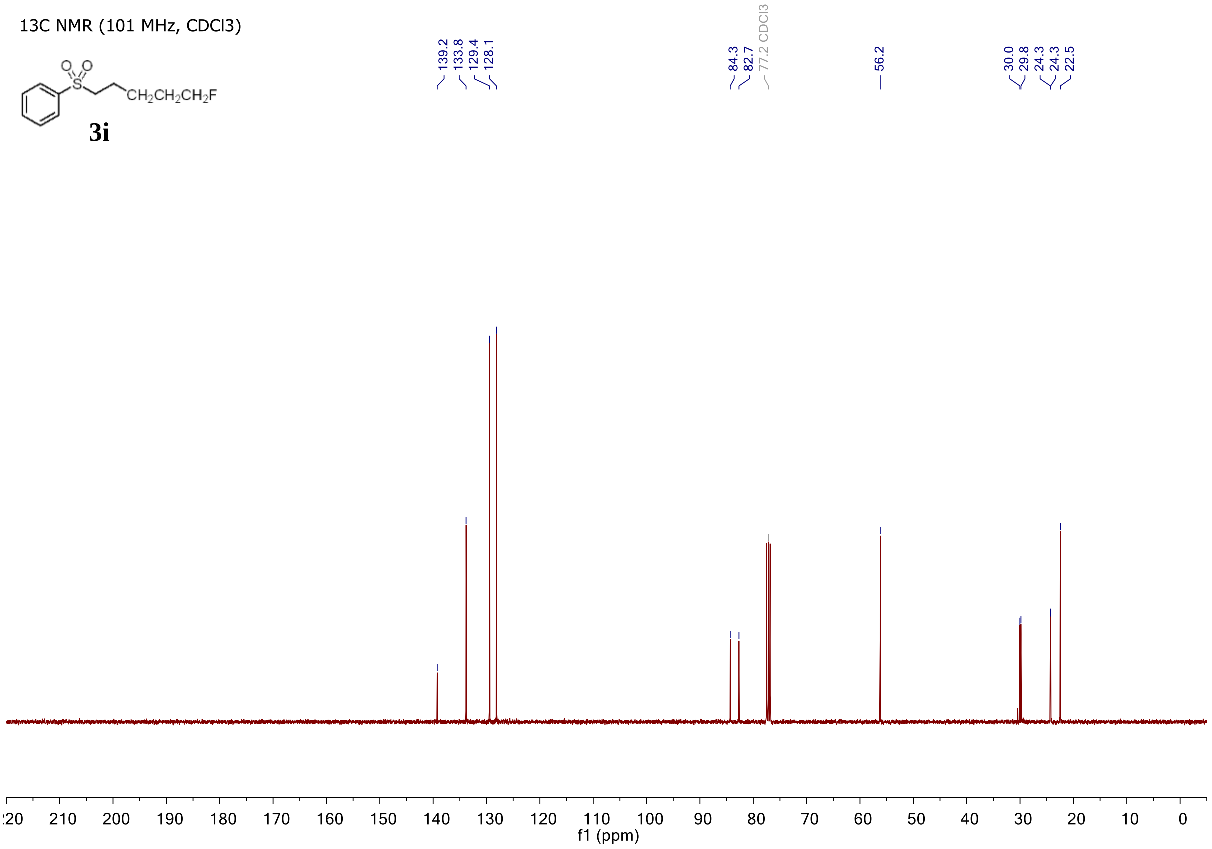

19F NMR (377 MHz, CDCl3)

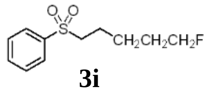

3i

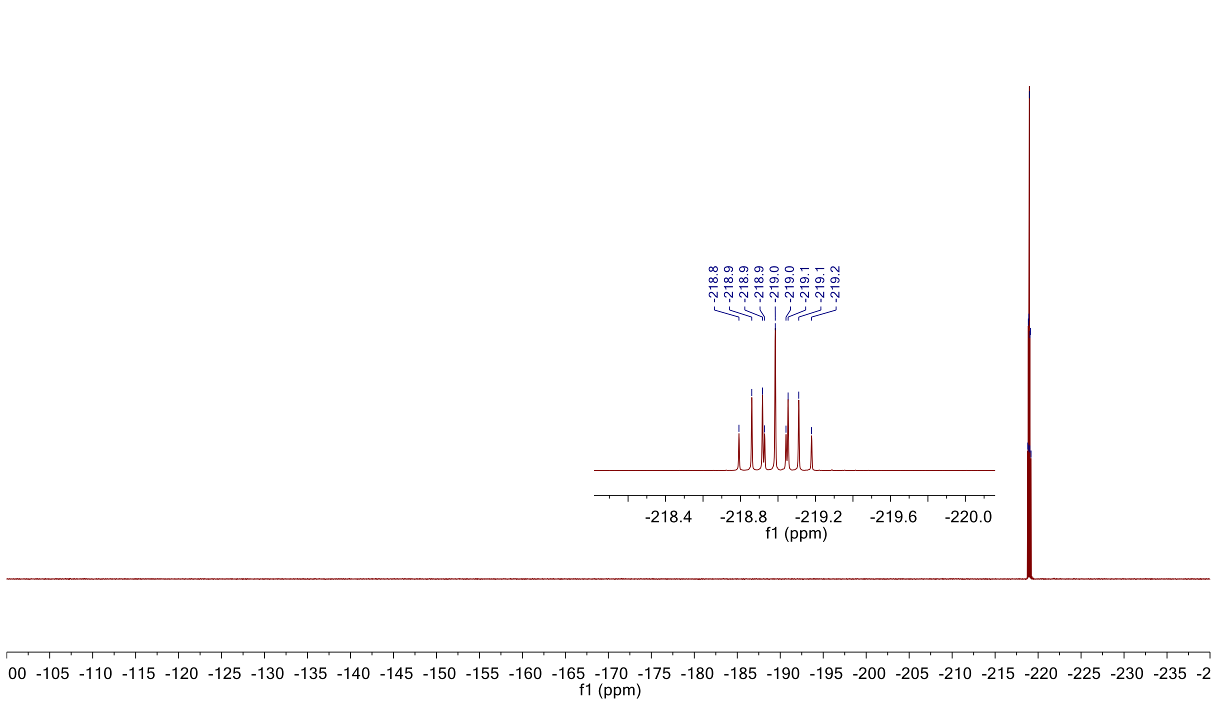

1H NMR (400 MHz, CDCl3)

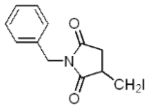

4a

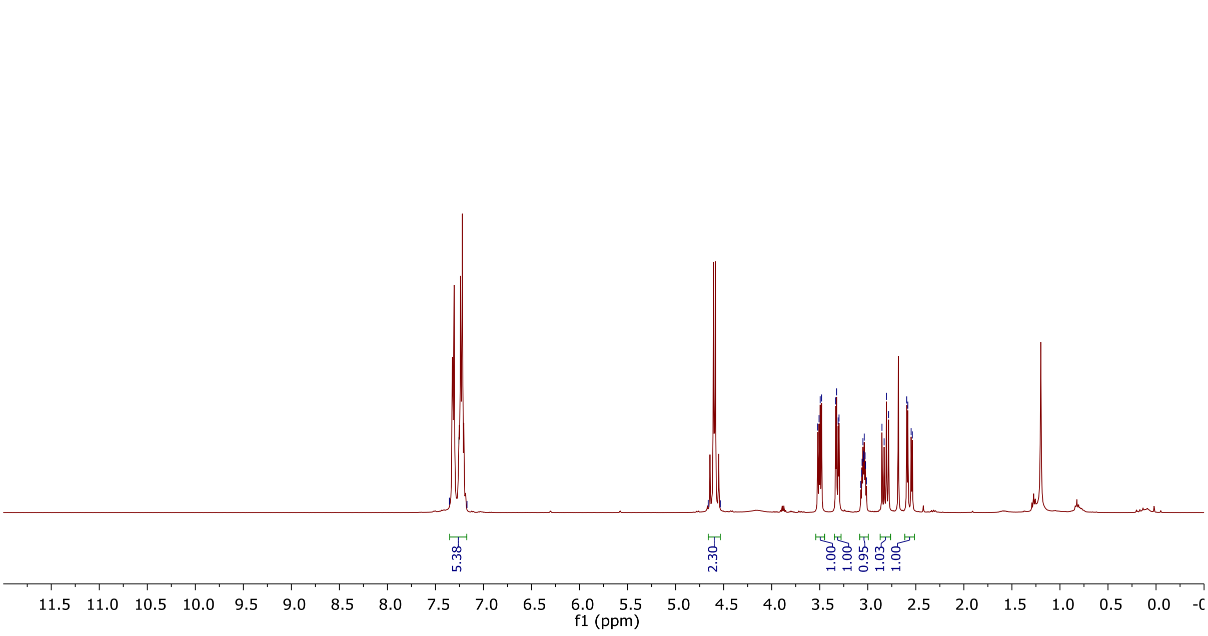

13C NMR (101 MHz, CDCl3)

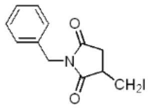

4a

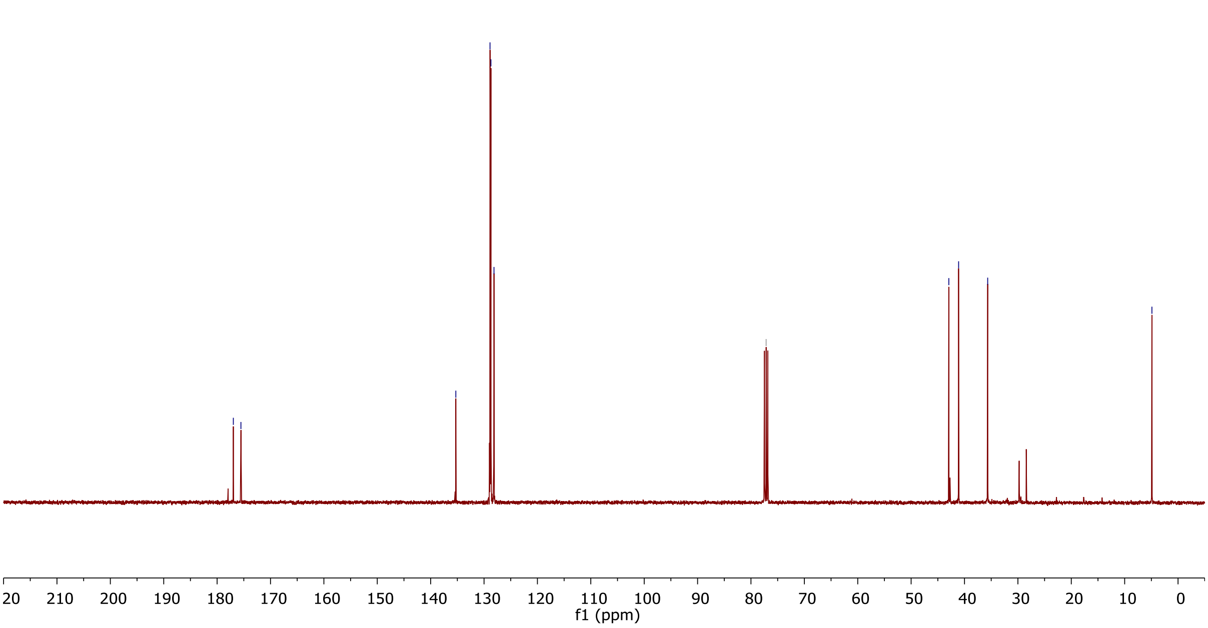

1H NMR (400 MHz, CDCl3)

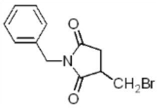

4b

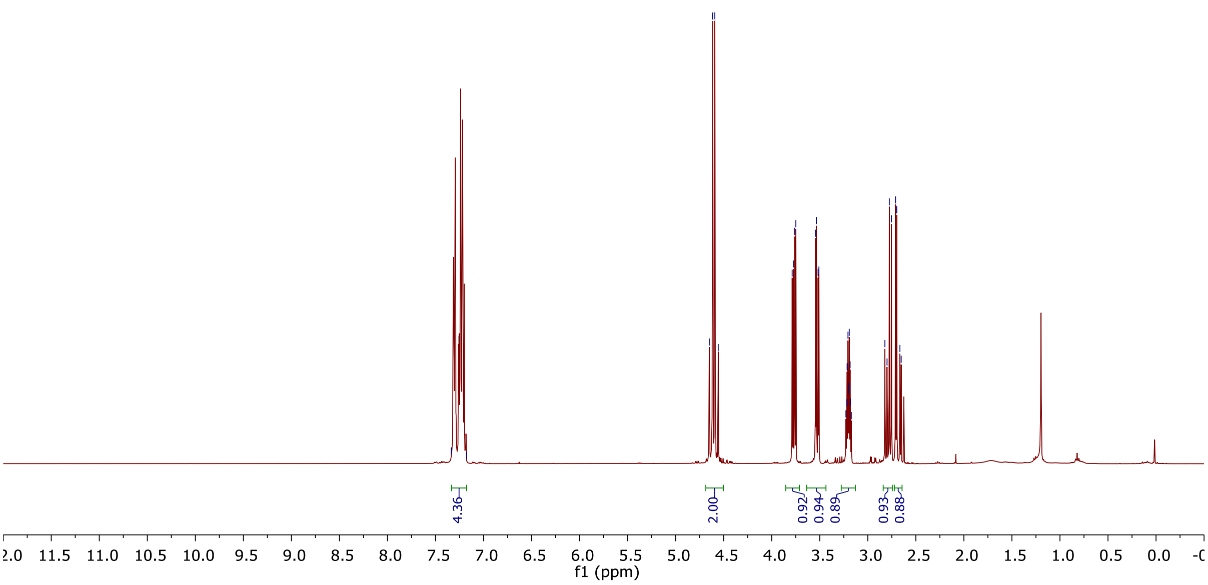

13C NMR (101 MHz, CDCl3)

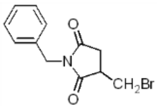

4b

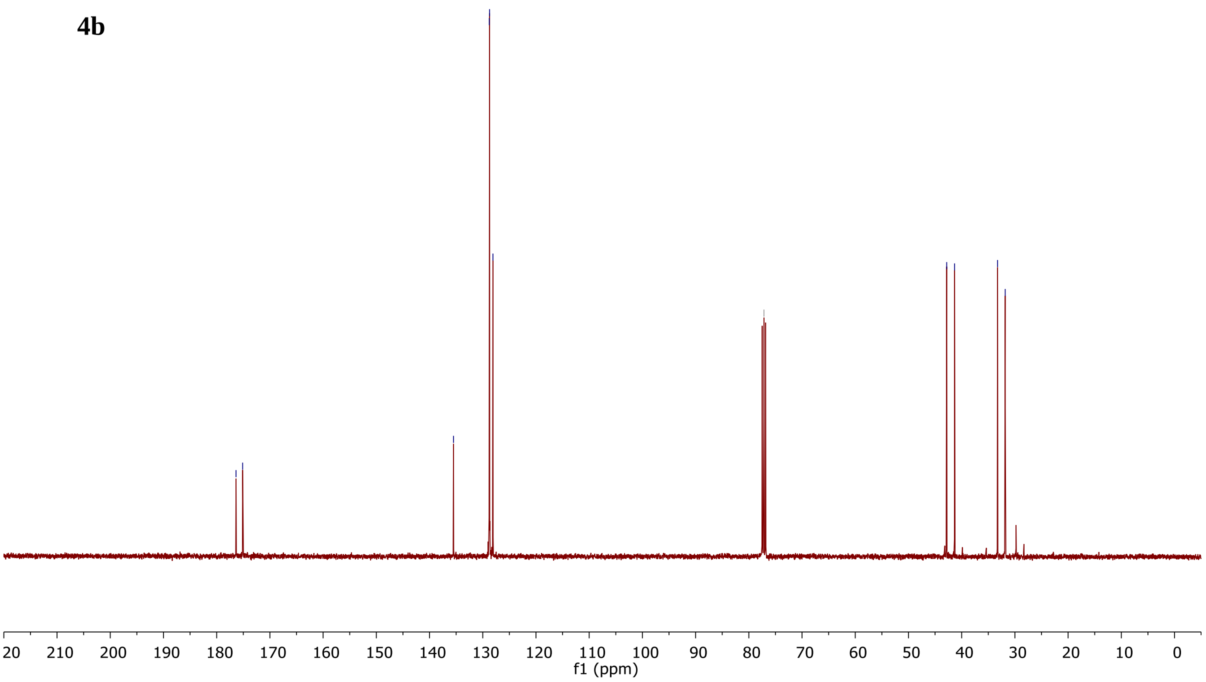

1H NMR (400 MHz, CDCl3)

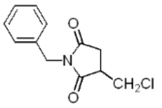

4c

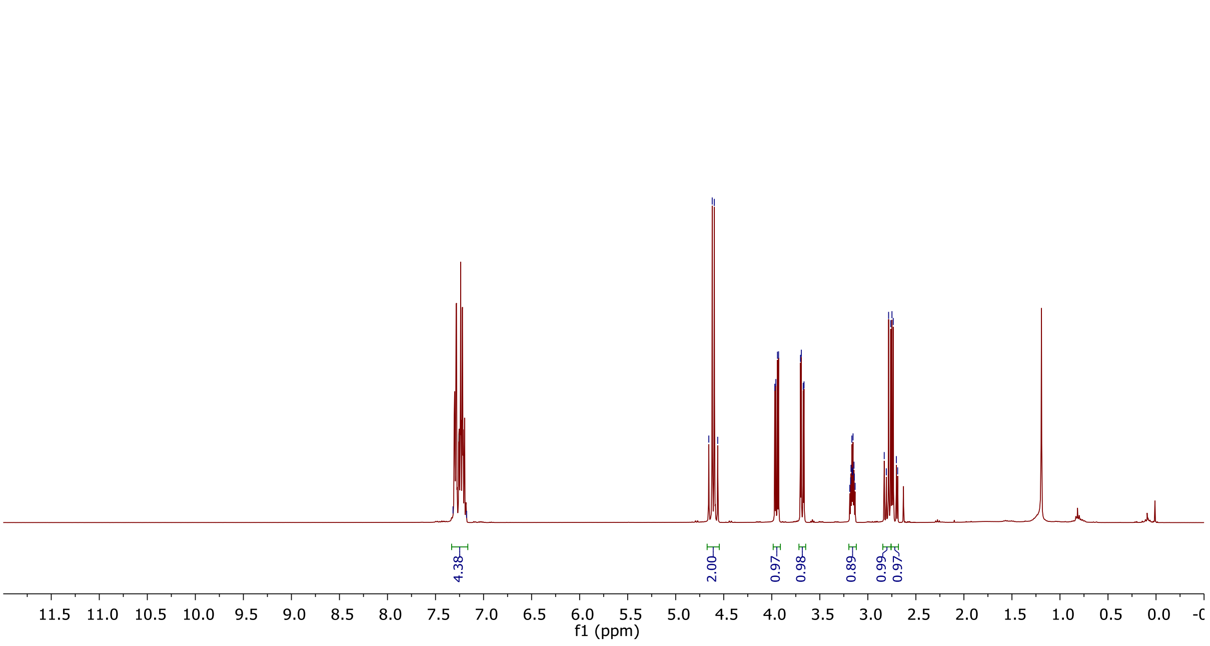

13C NMR (101 MHz, CDCl3)

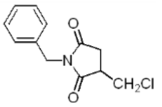

4c

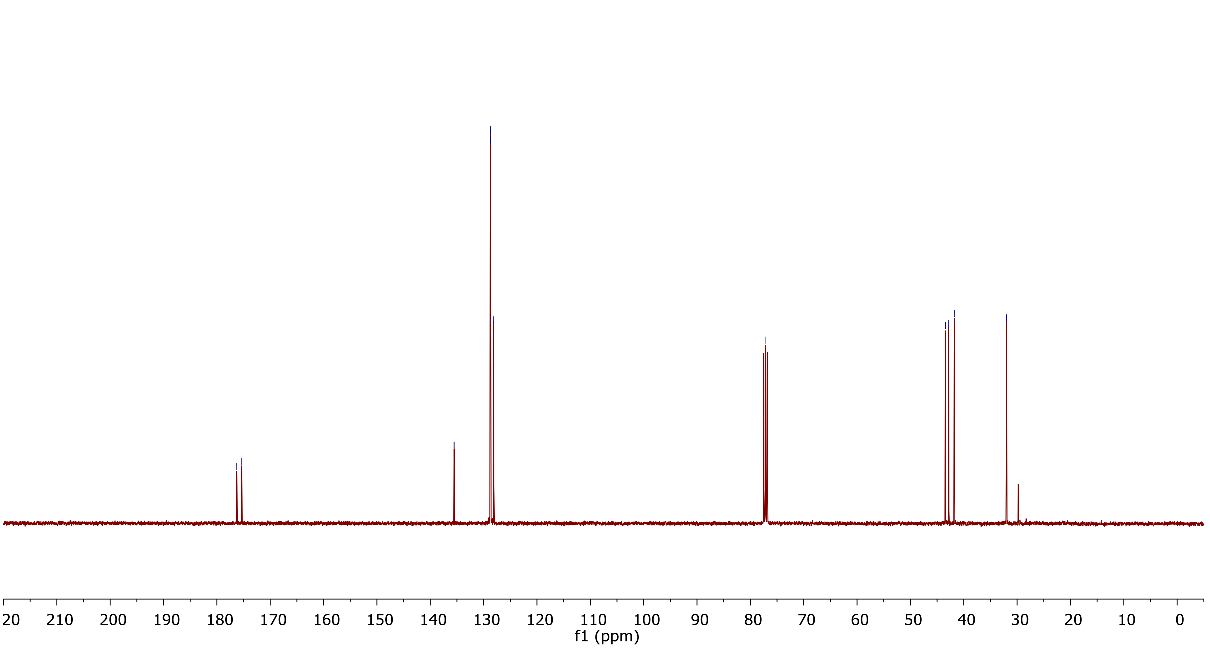

1H NMR (400 MHz, CDCl3)

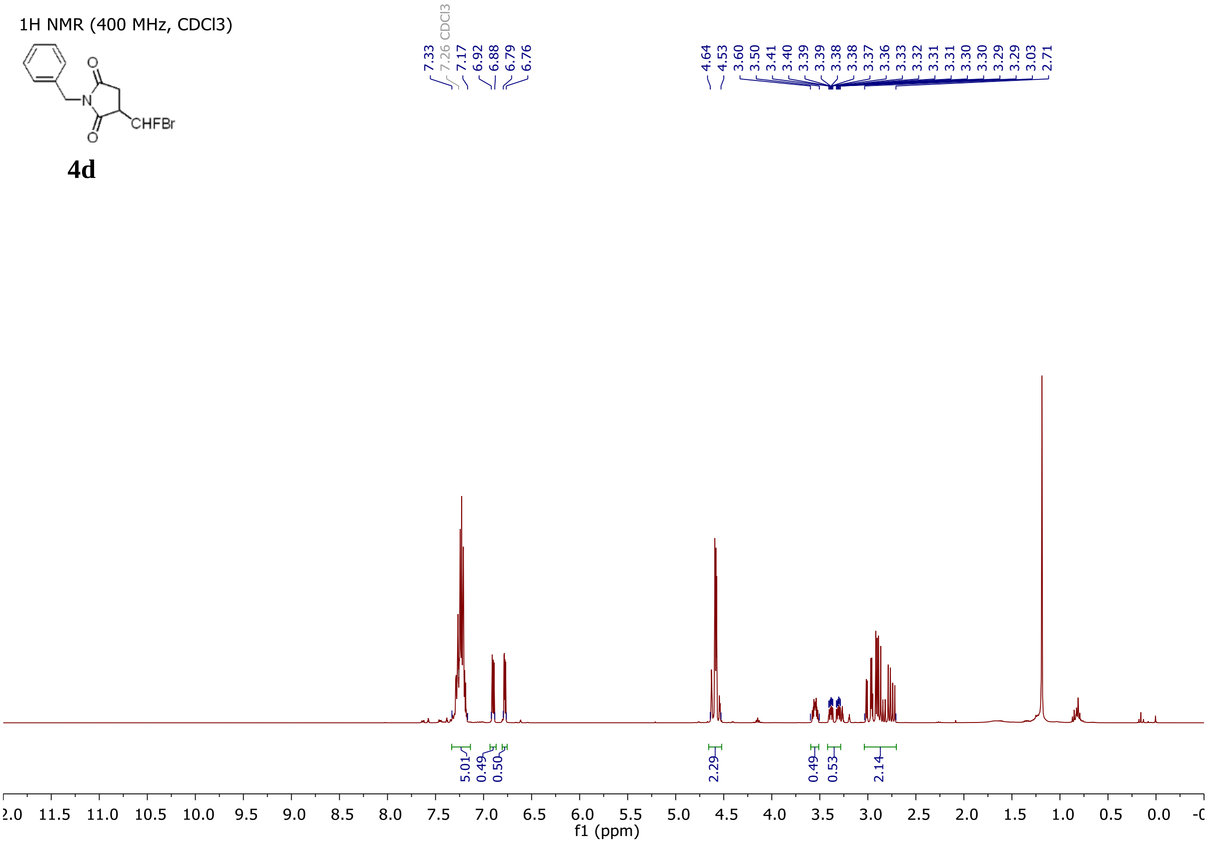

4d

13C NMR (101 MHz, CDCl3)

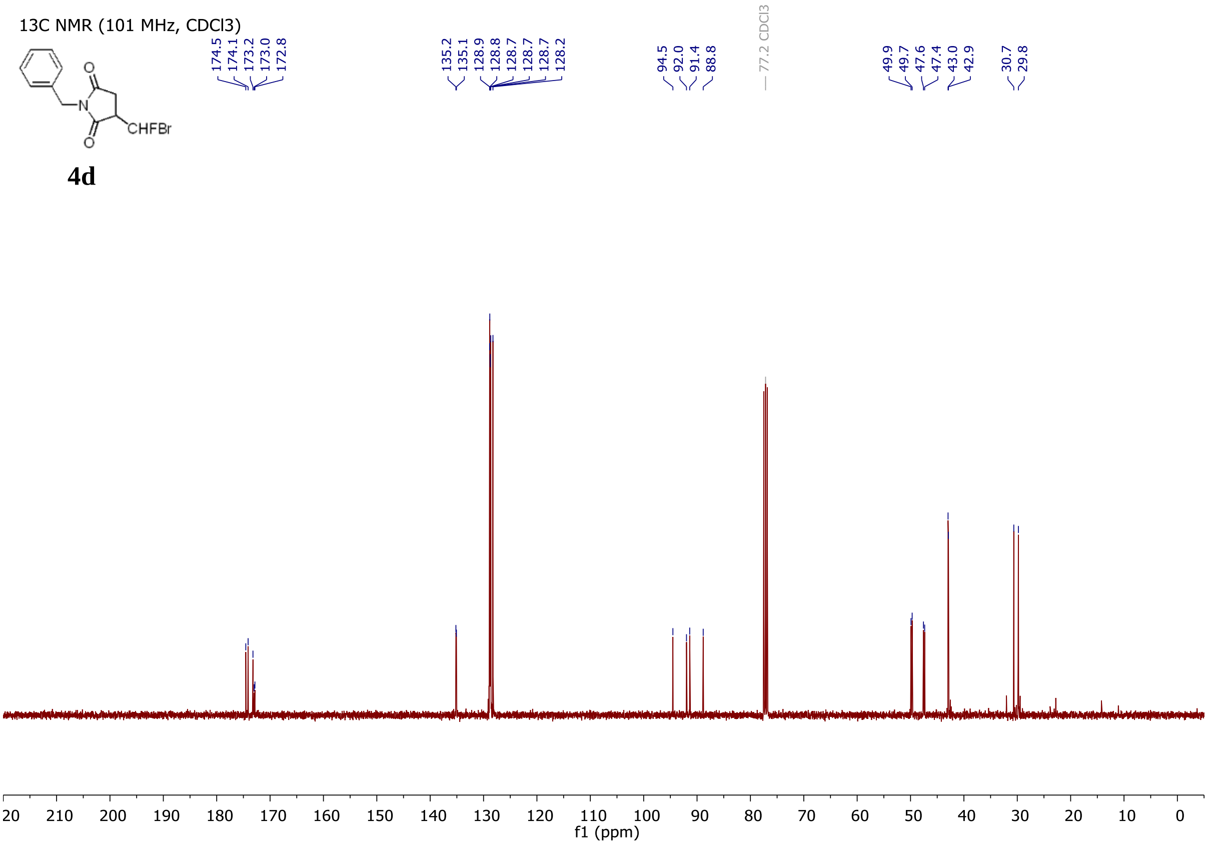

4d

19F NMR (377 MHz, CDCl3)

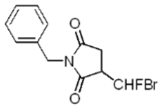

4d

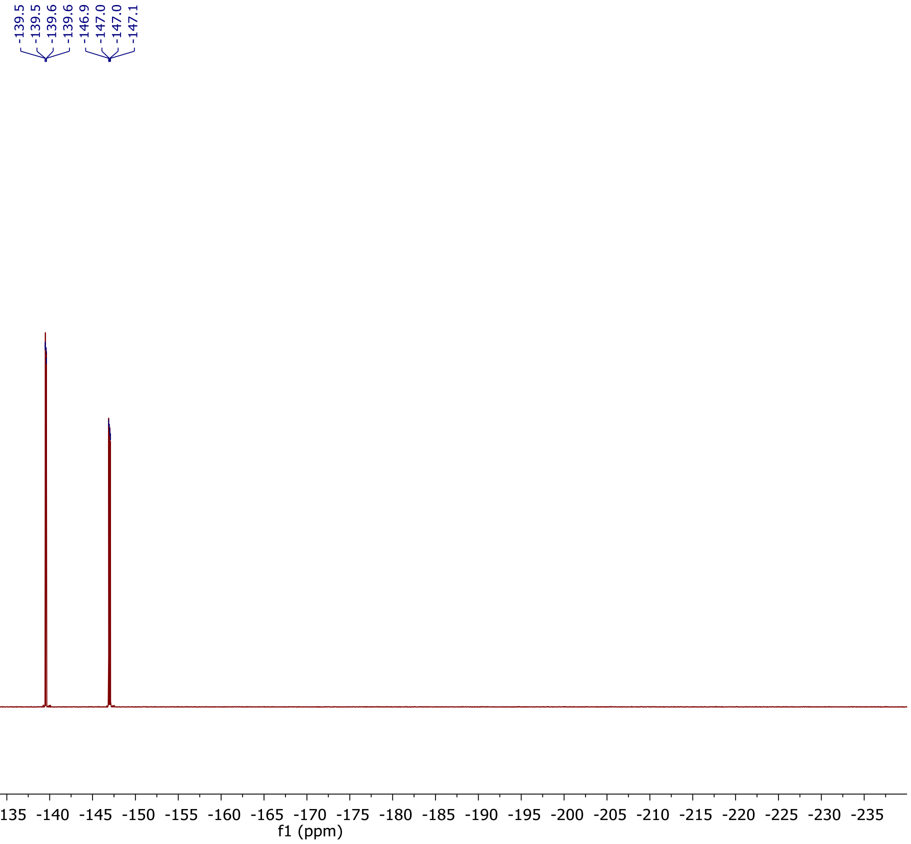

1H NMR (400 MHz, CDCl3)

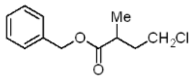

4f

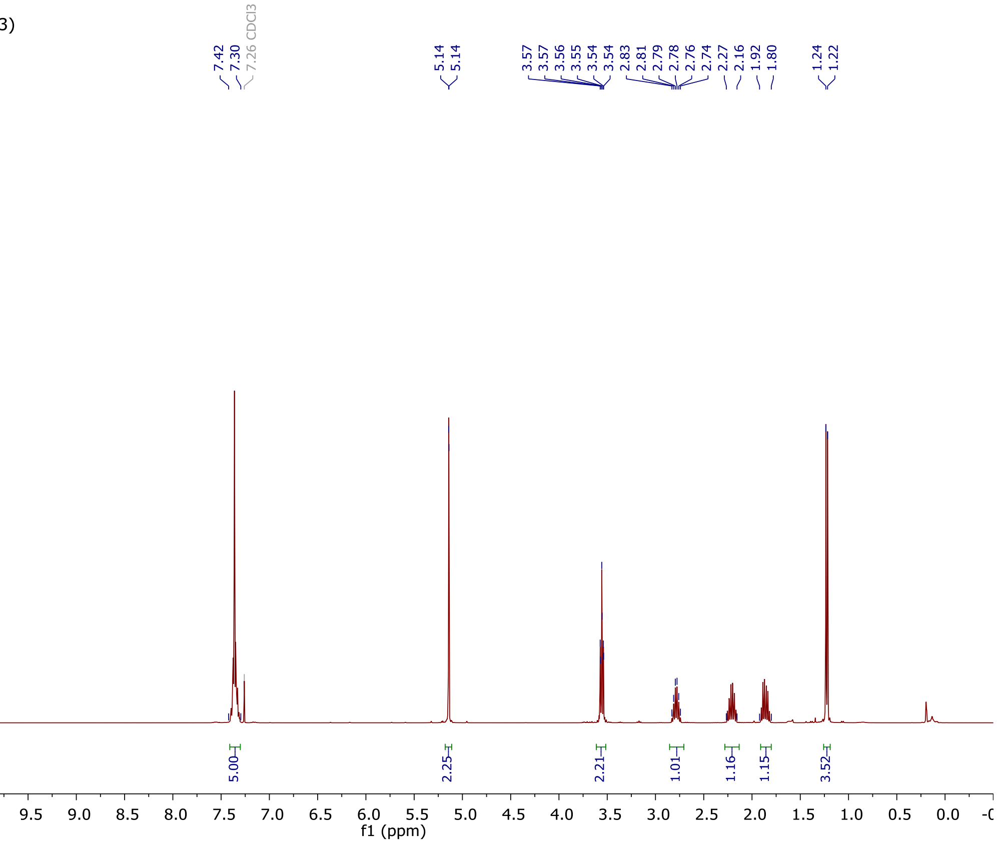

13C NMR (101 MHz, CDCl3)

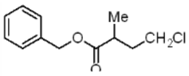

4f

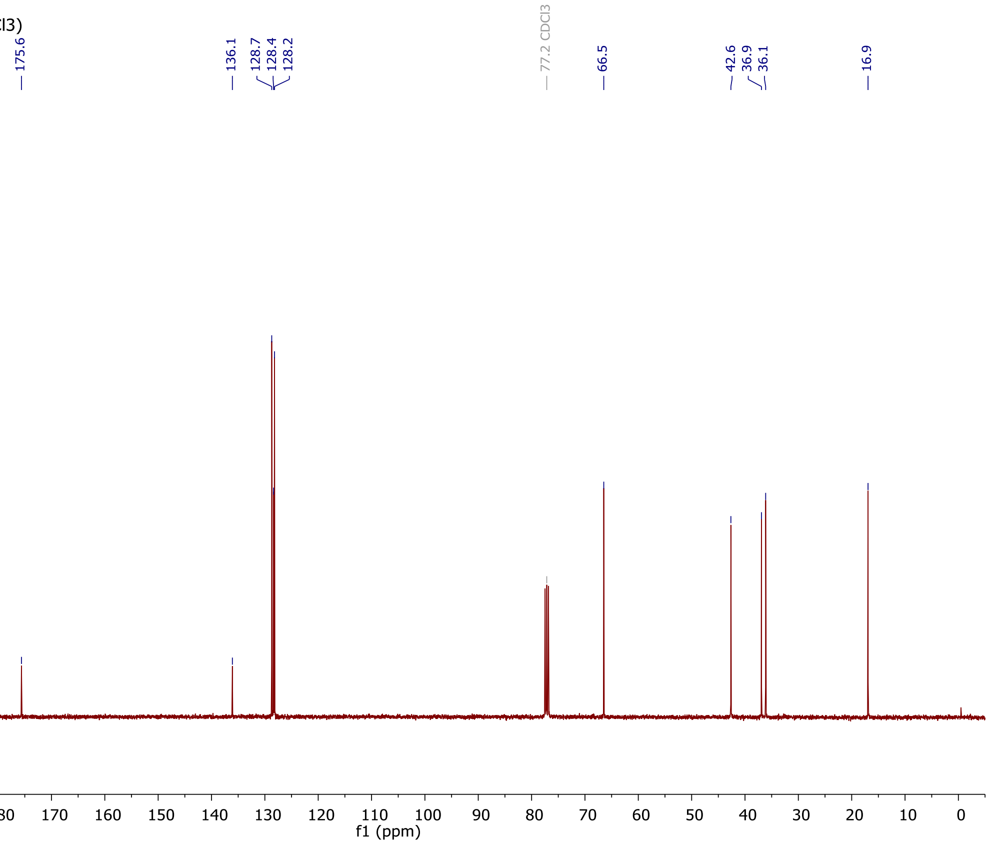

1H NMR (400 MHz, CDCl3)

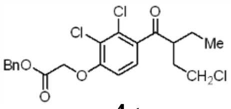

4g

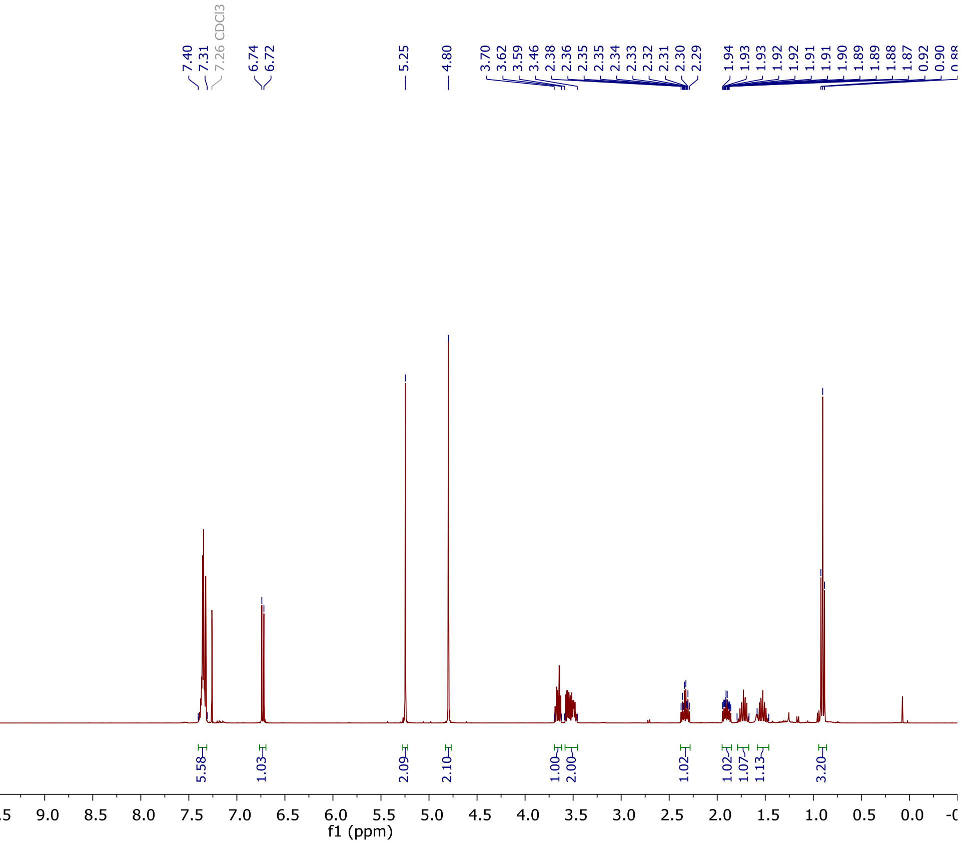

13C NMR (101 MHz, CDCl3)

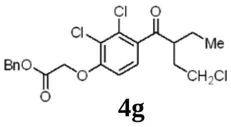

4g

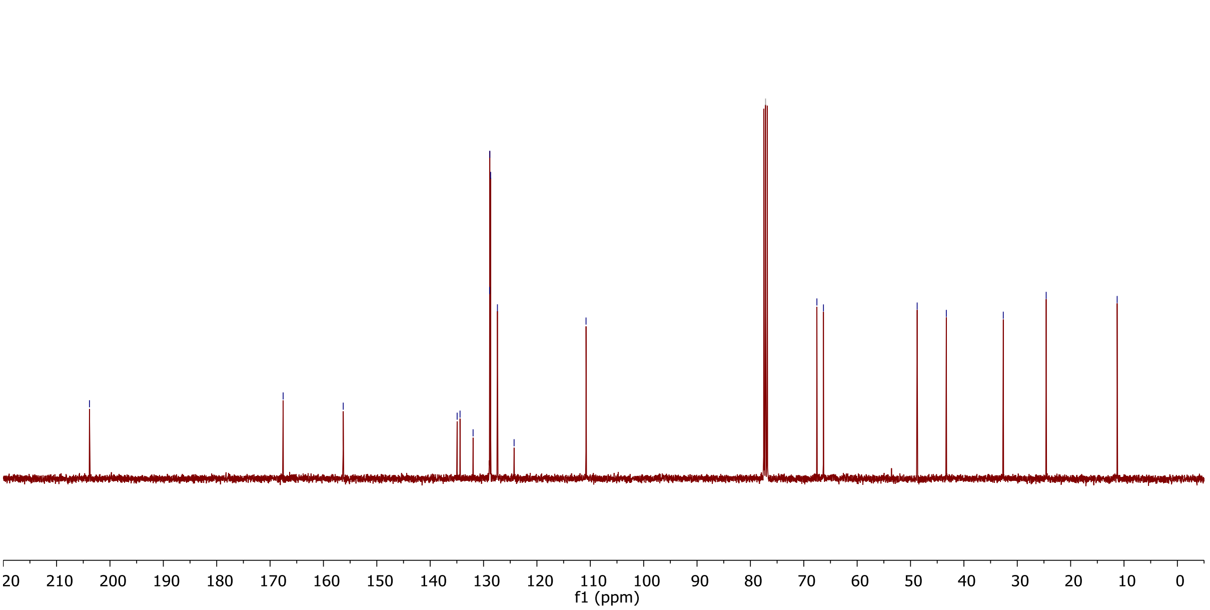

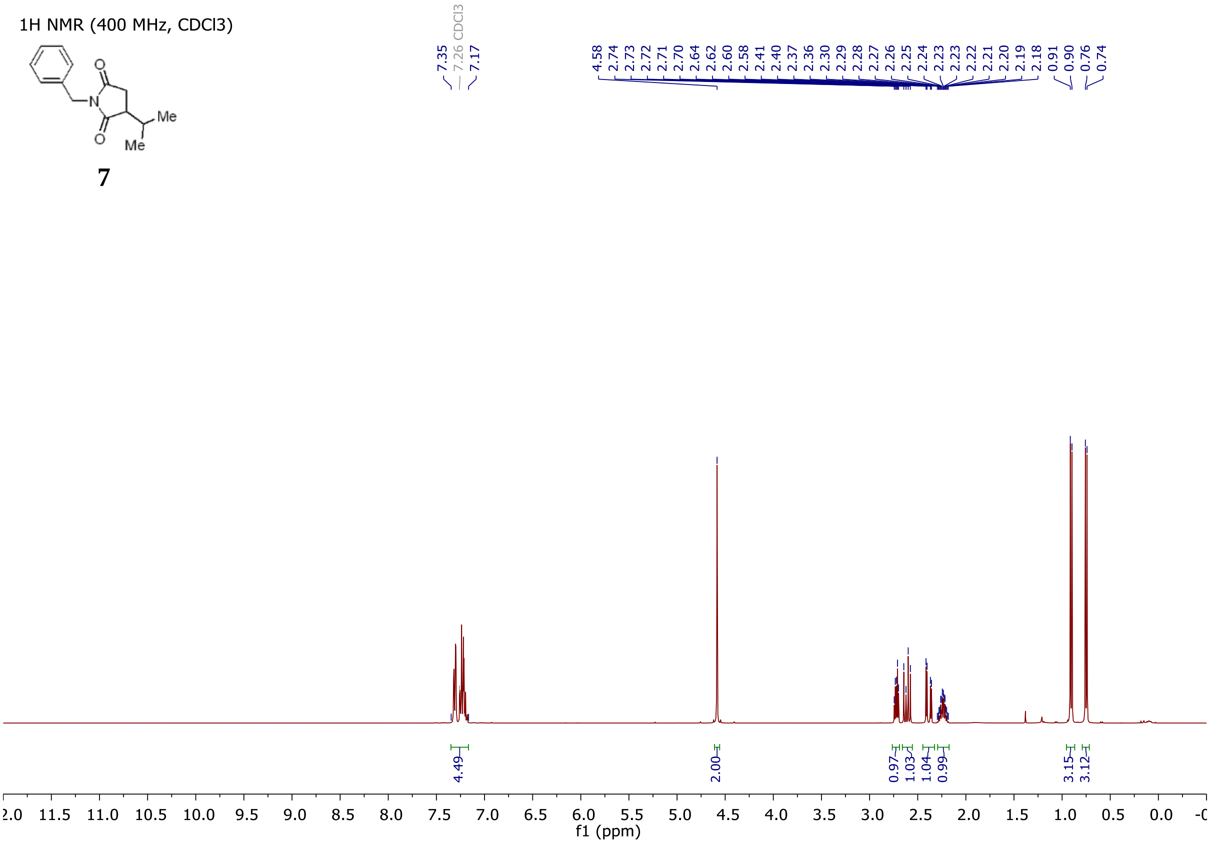
1H NMR (400 MHz, CDCl3)
7
7.35
7.26 CDCl3
7.17
4.58
2.74
2.73
2.72
2.71
2.70
2.64
2.62
2.60
2.58
2.41
2.40
2.37
2.36
2.30
2.29
2.28
2.27
2.26
2.25
2.24
2.23
2.23
2.22
2.21
2.20
2.19
2.18
0.91
0.90
0.76
0.74
4.49
2.00
0.97
1.03
1.04
0.99
3.15
3.12
f1 (ppm)

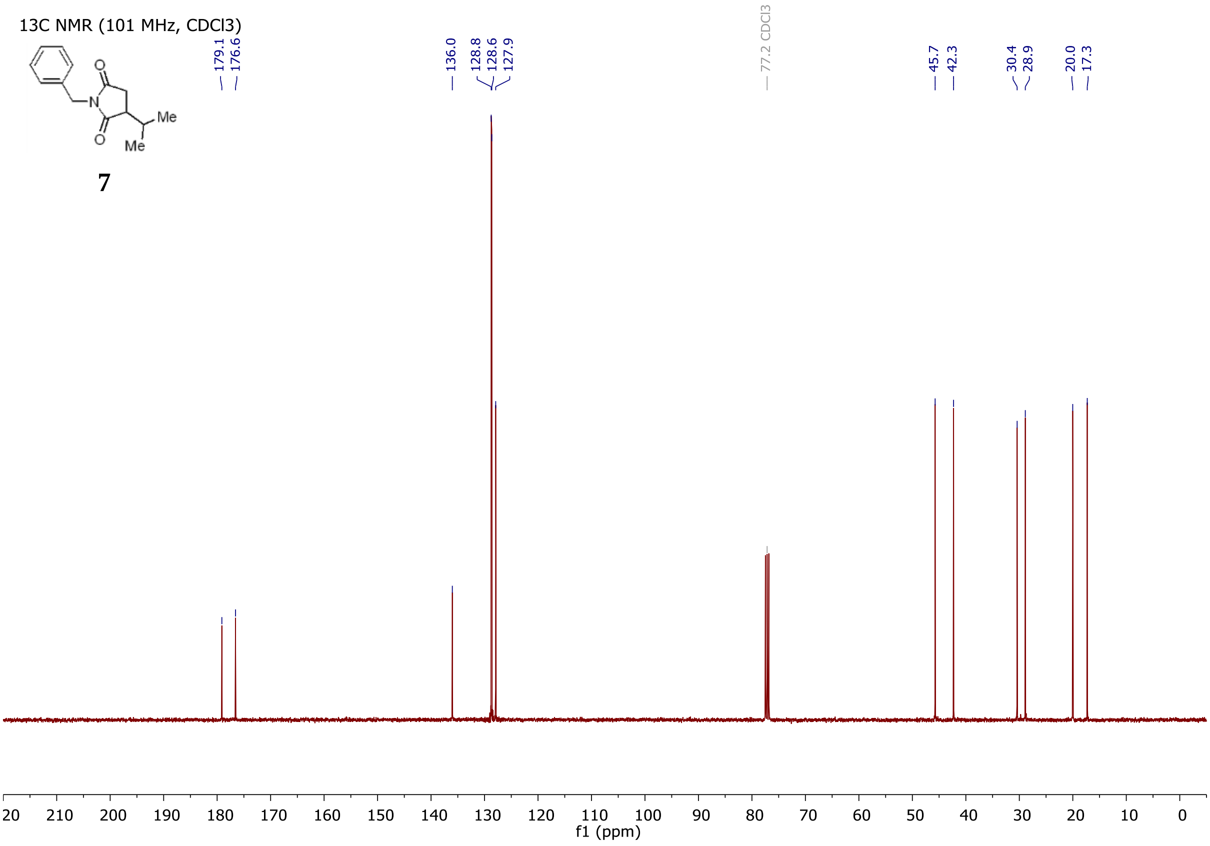
13C NMR (101 MHz, CDCl3)
7
179.1
176.6
136.0
128.8
128.6
127.9
77.2 CDCl3
45.7
42.3
30.4
28.9
20.0
17.3
f1 (ppm)

1H NMR (400 MHz, CDCl3)

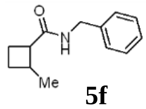

5f

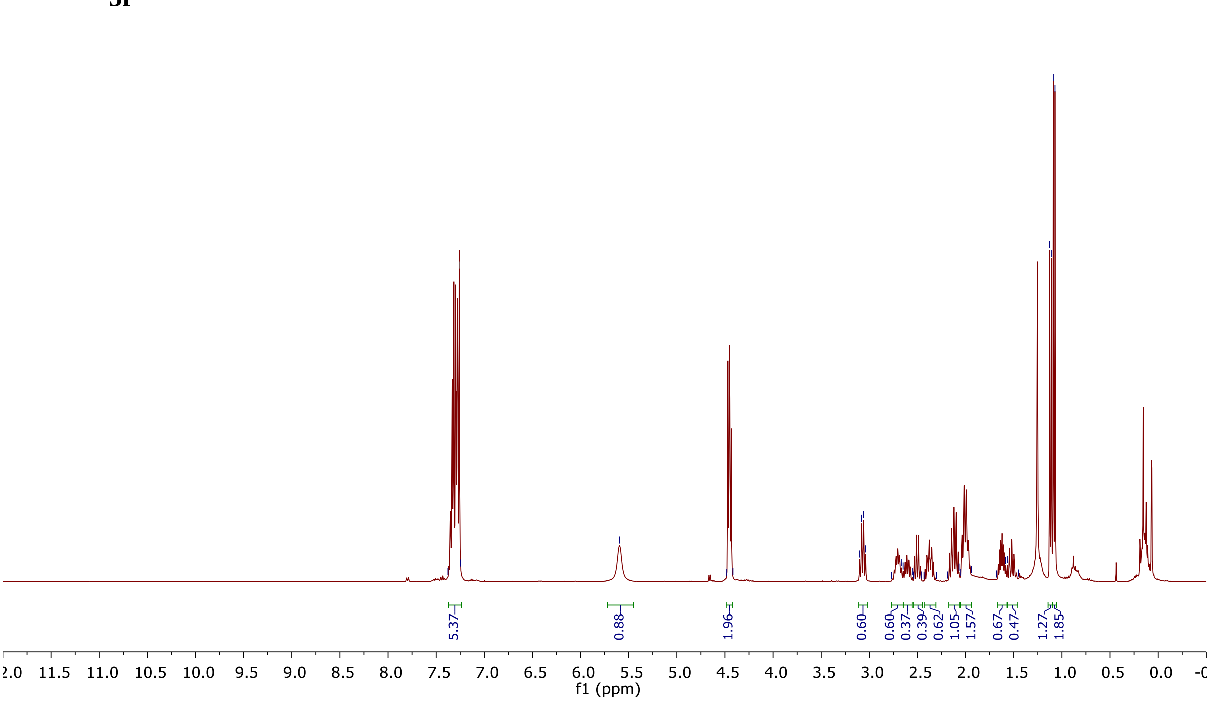

13C NMR (101 MHz, CDCl3)

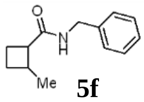

5f

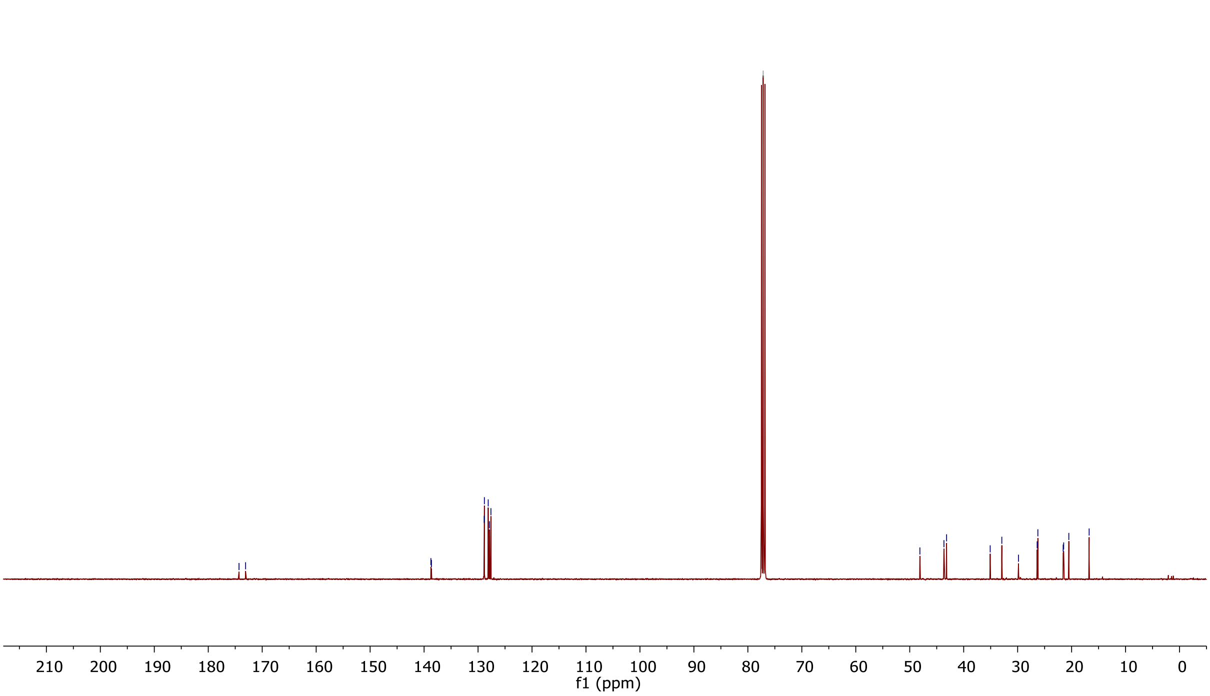

1H NMR (400 MHz, CDCl3

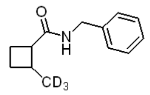

5g

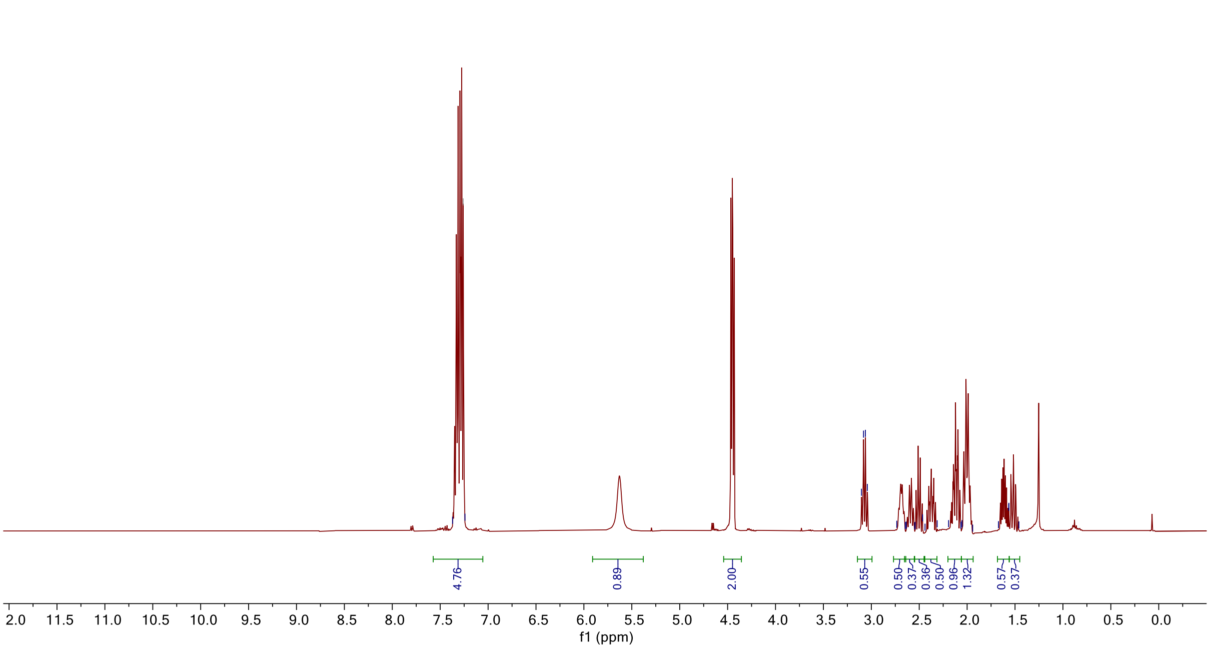

13C NMR (101 MHz, CDCl3)

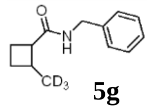

5g

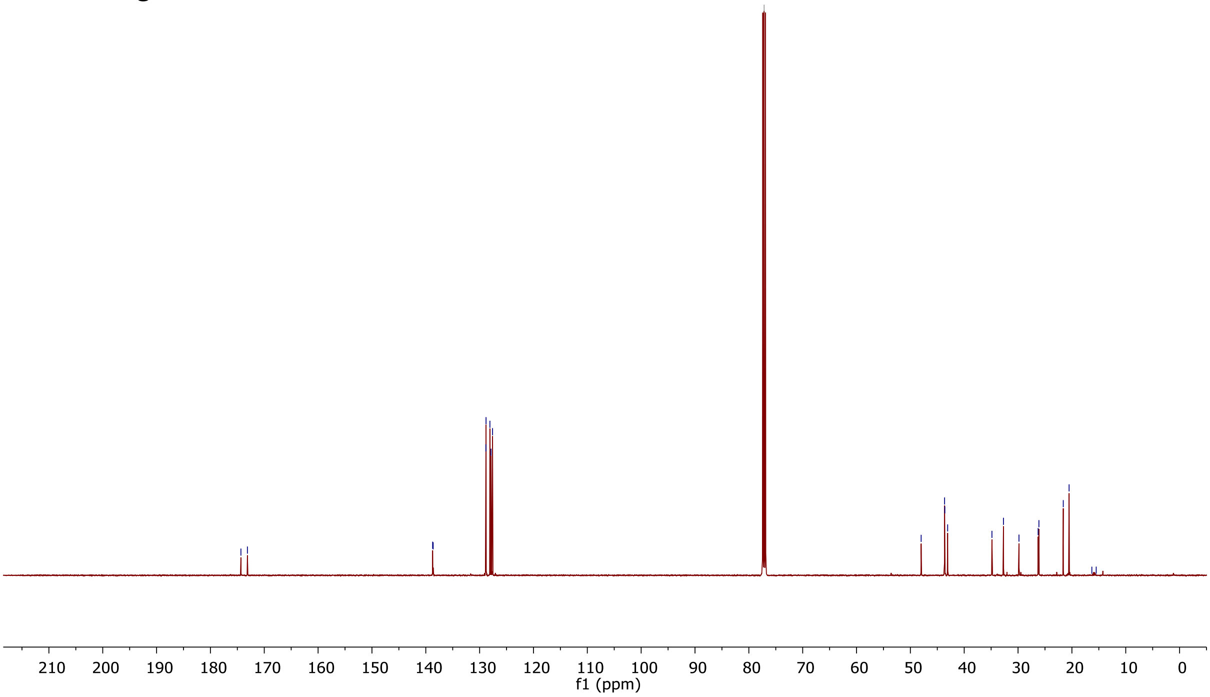

1H NMR (400 MHz, CDCl3)

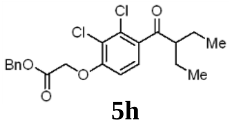

5h

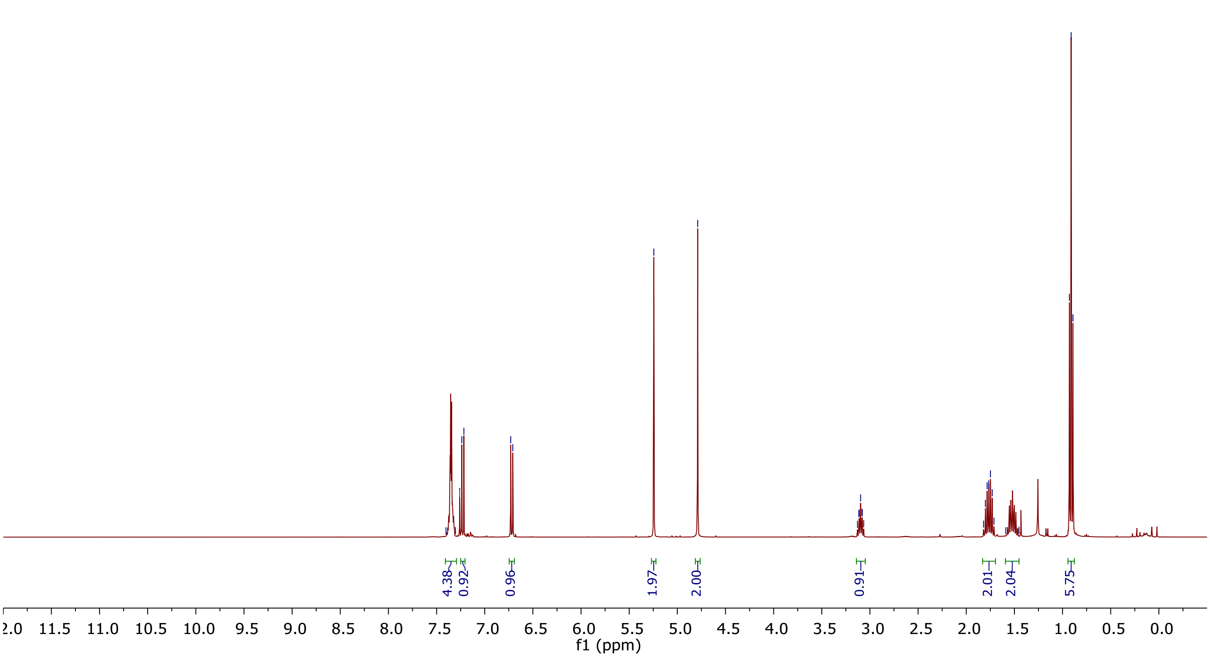

13C NMR (101 MHz, CDCl3)

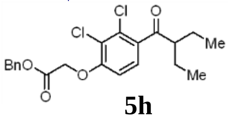

5h

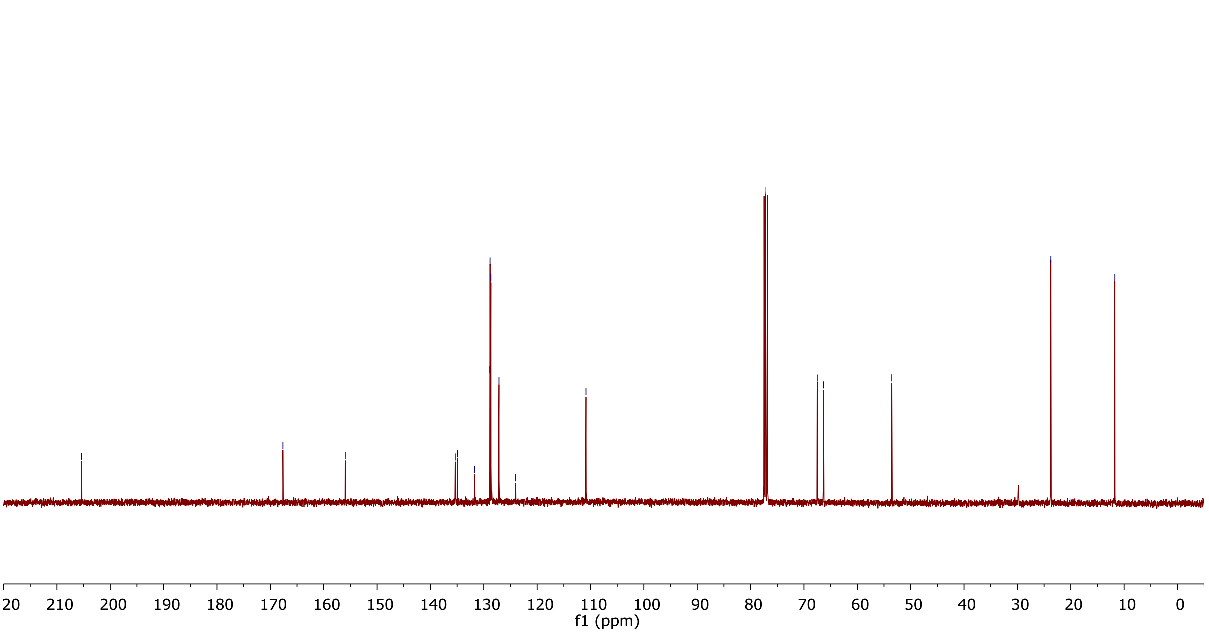

1H NMR (500 MHz, CDCl3)

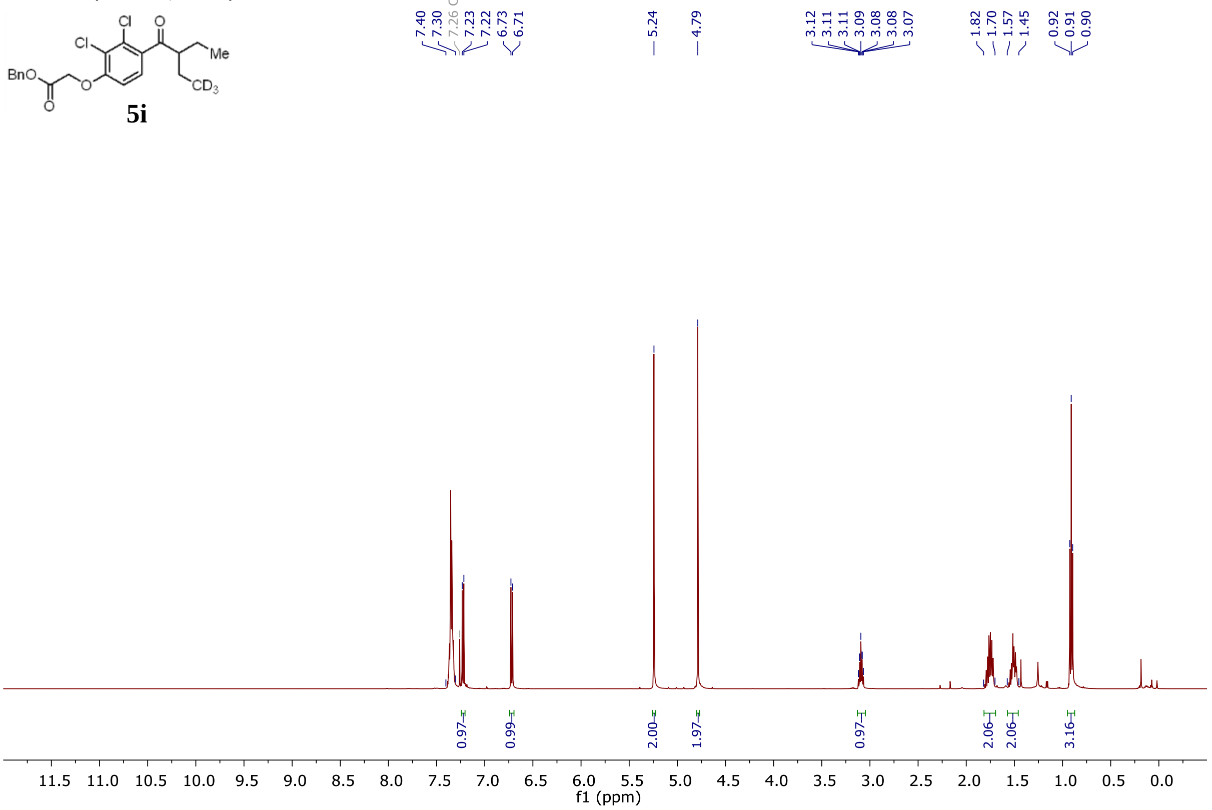

13C NMR (126 MHz, CDCl3)

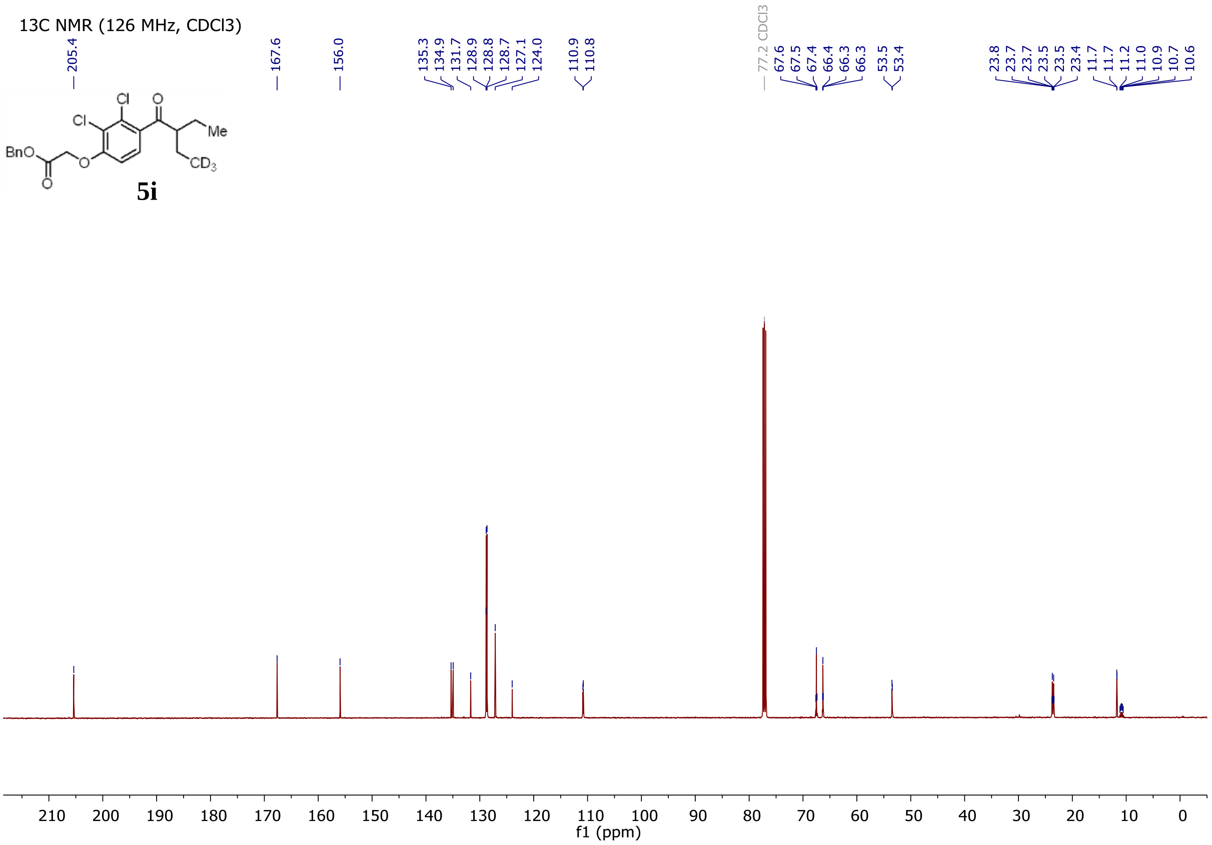

1H NMR (500 MHz, CDCl3)

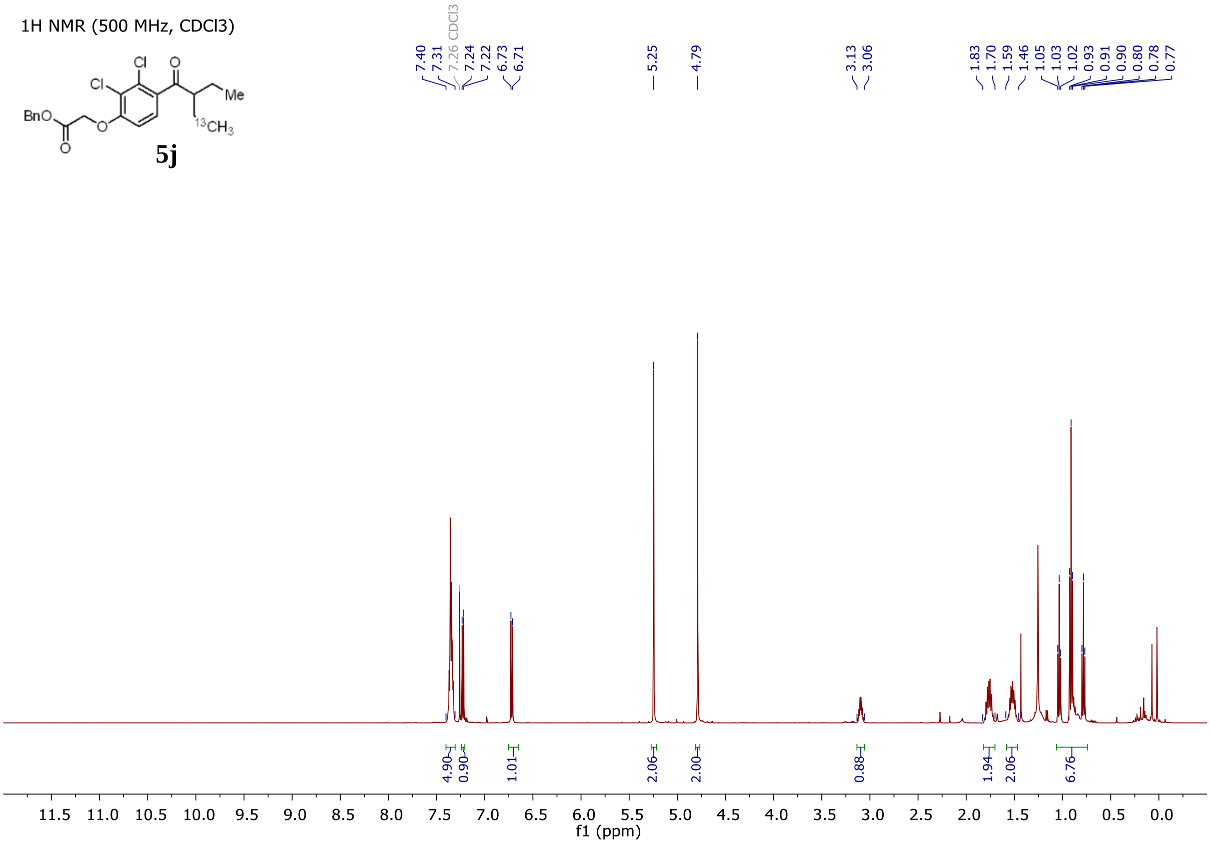

5j

13C NMR (126 MHz, CDCl3)

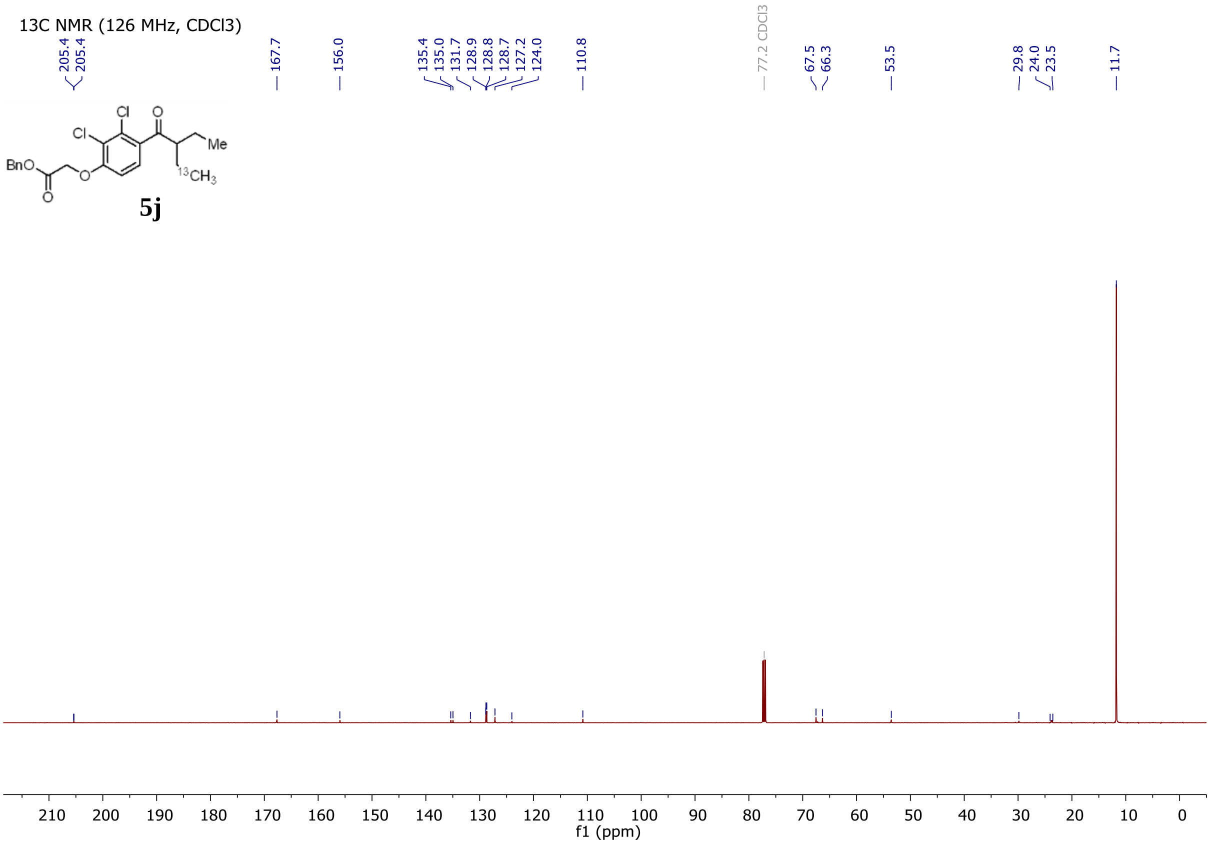

5j

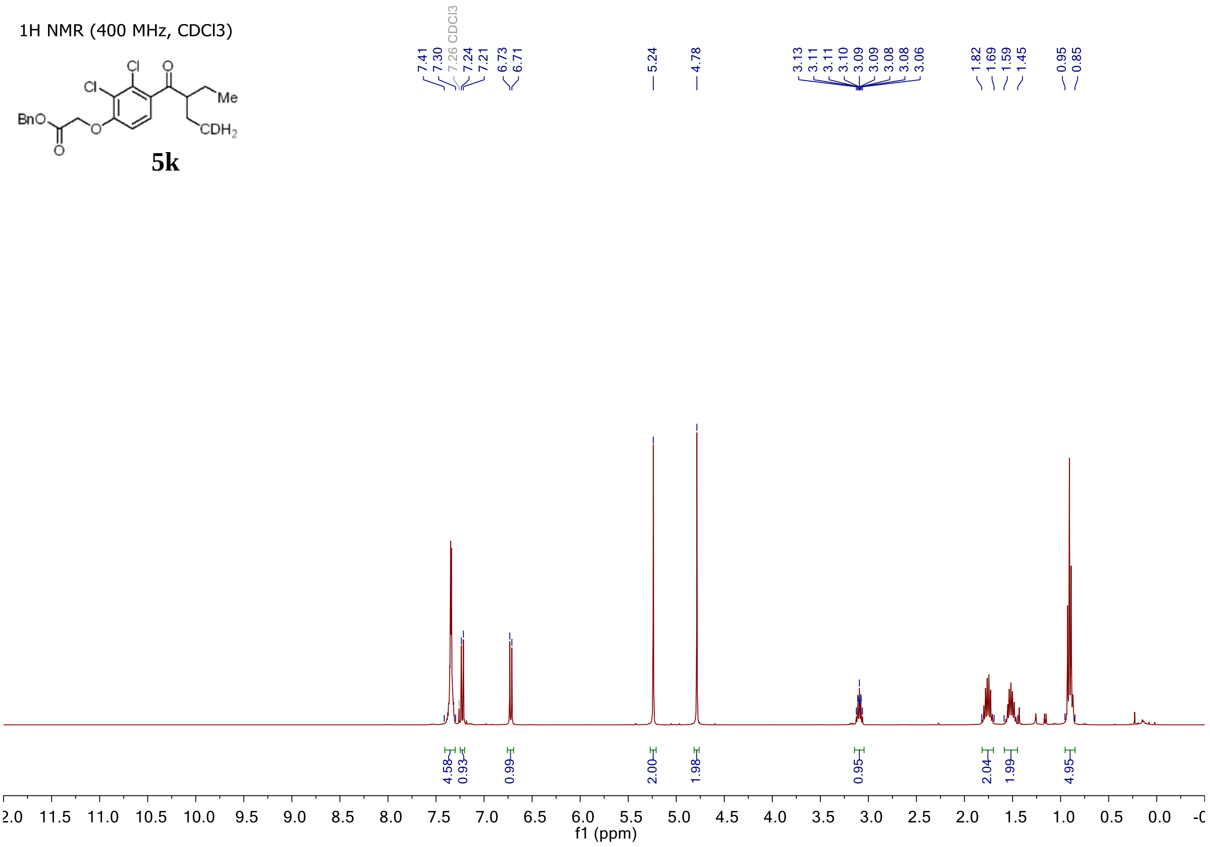
1H NMR (400 MHz, CDCl3)
Cl
O
Cl
Me
BnO
O
CDH2
O
5k
7.41
7.30
7.26 CDCl3
7.24
7.21
6.73
6.71
5.24
4.78
3.13
3.11
3.11
3.10
3.09
3.09
3.08
3.08
3.06
1.82
1.69
1.59
1.45
0.95
0.85
4.58
0.93
0.99
2.00
1.98
0.95
2.04
1.99
4.95
f1 (ppm)

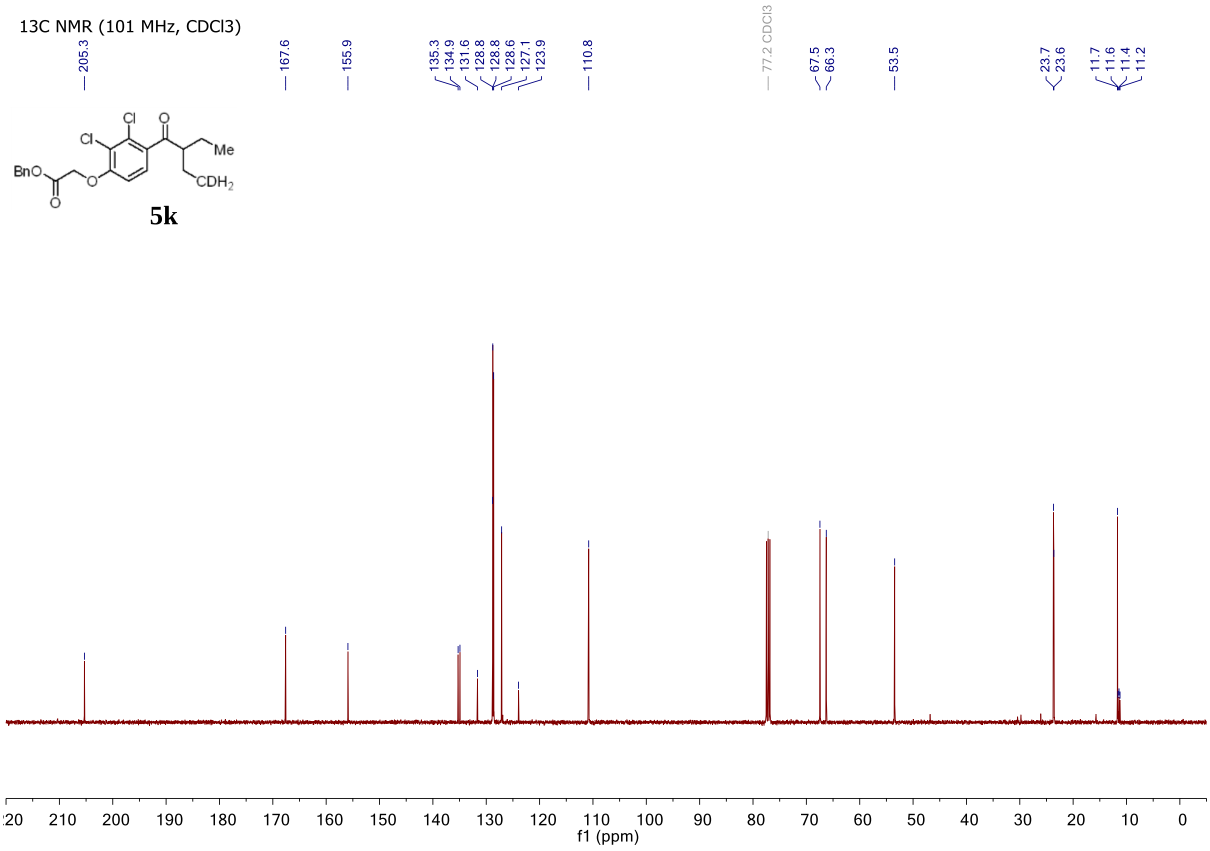
13C NMR (101 MHz, CDCl3)
205.3
167.6
155.9
135.3
134.9
131.6
128.8
128.8
128.6
127.1
123.9
110.8
77.2 CDCl3
67.5
66.3
53.5
23.7
23.6
11.7
11.6
11.4
11.2
Cl
O
Cl
Me
BnO
O
CDH2
O
5k
f1 (ppm)

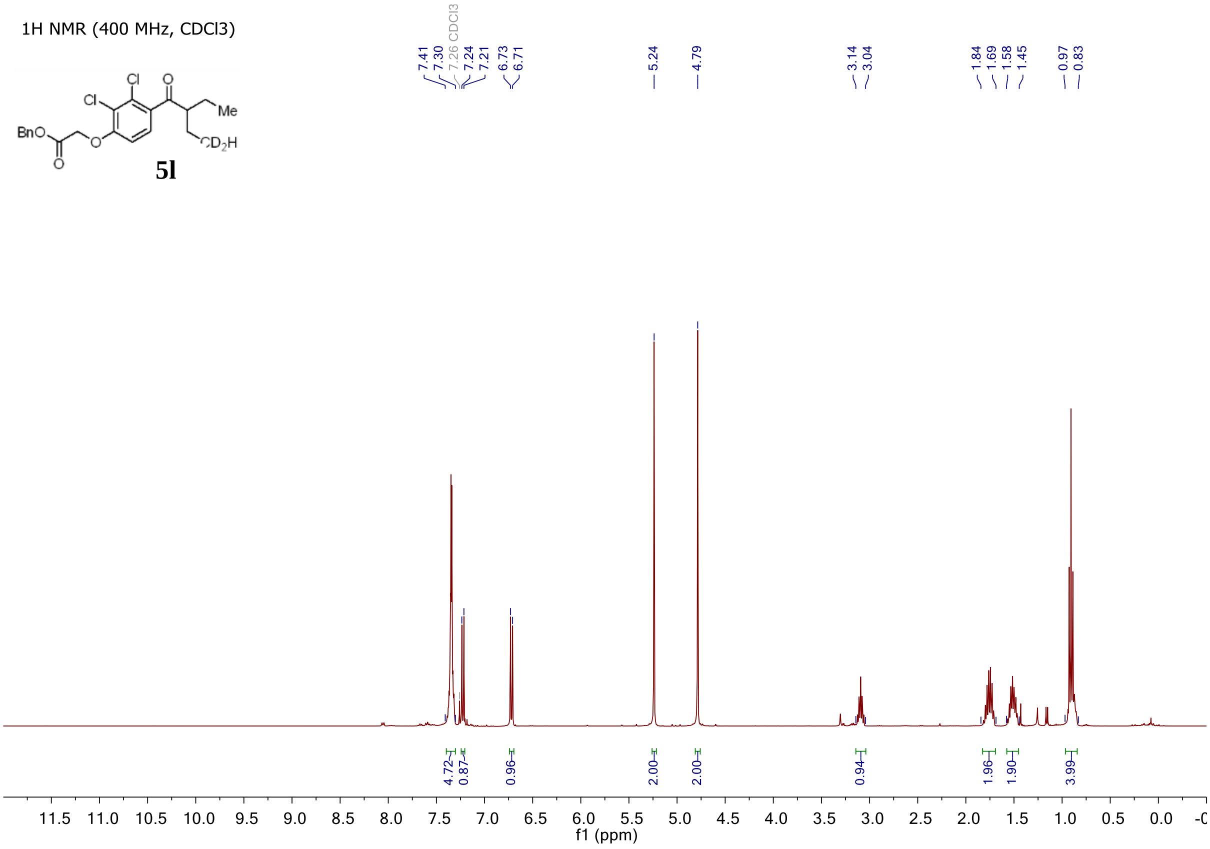
1H NMR (400 MHz, CDCl3)
5l
7.41
7.30
7.26 CDCl3
7.24
7.21
6.73
6.71
5.24
4.79
3.14
3.04
1.84
1.69
1.58
1.45
0.97
0.83
4.72
0.87
0.96
2.00
2.00
0.94
1.96
1.90
3.99
f1 (ppm)

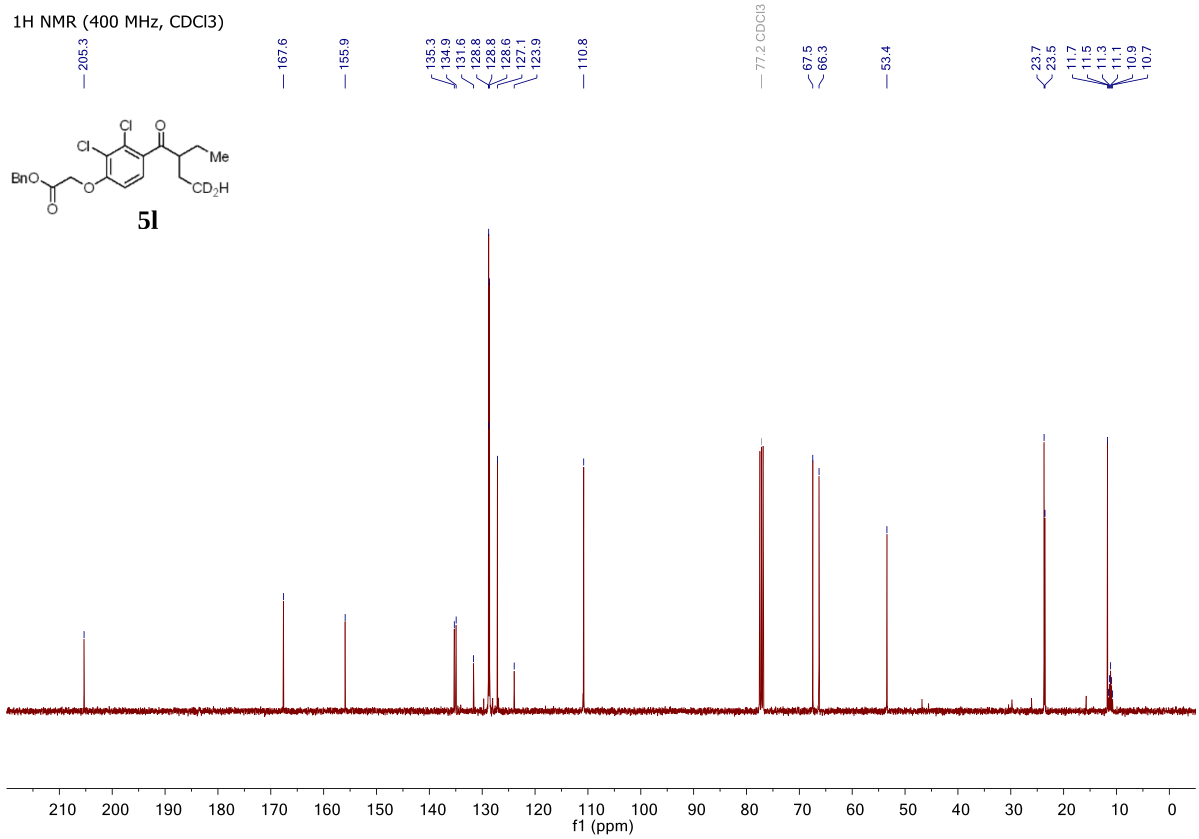
1H NMR (400 MHz, CDCl3)
5l
205.3
167.6
155.9
135.3
134.9
131.6
128.8
128.8
128.6
127.1
123.9
110.8
77.2 CDCl3
67.5
66.3
53.4
23.7
23.5
11.7
11.5
11.3
11.1
10.9
10.7
f1 (ppm)

1H NMR (400 MHz, CDCl3)

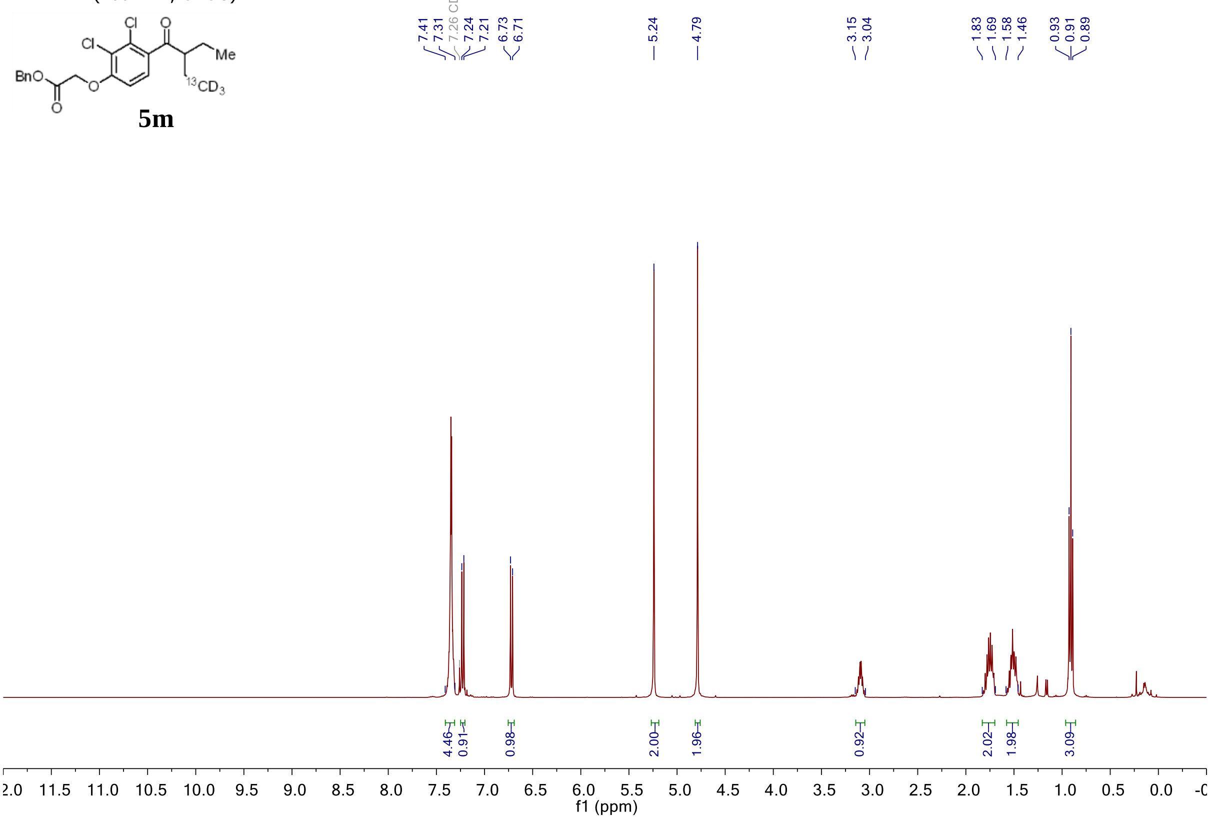

5m

13C NMR (101 MHz, CDCl3)

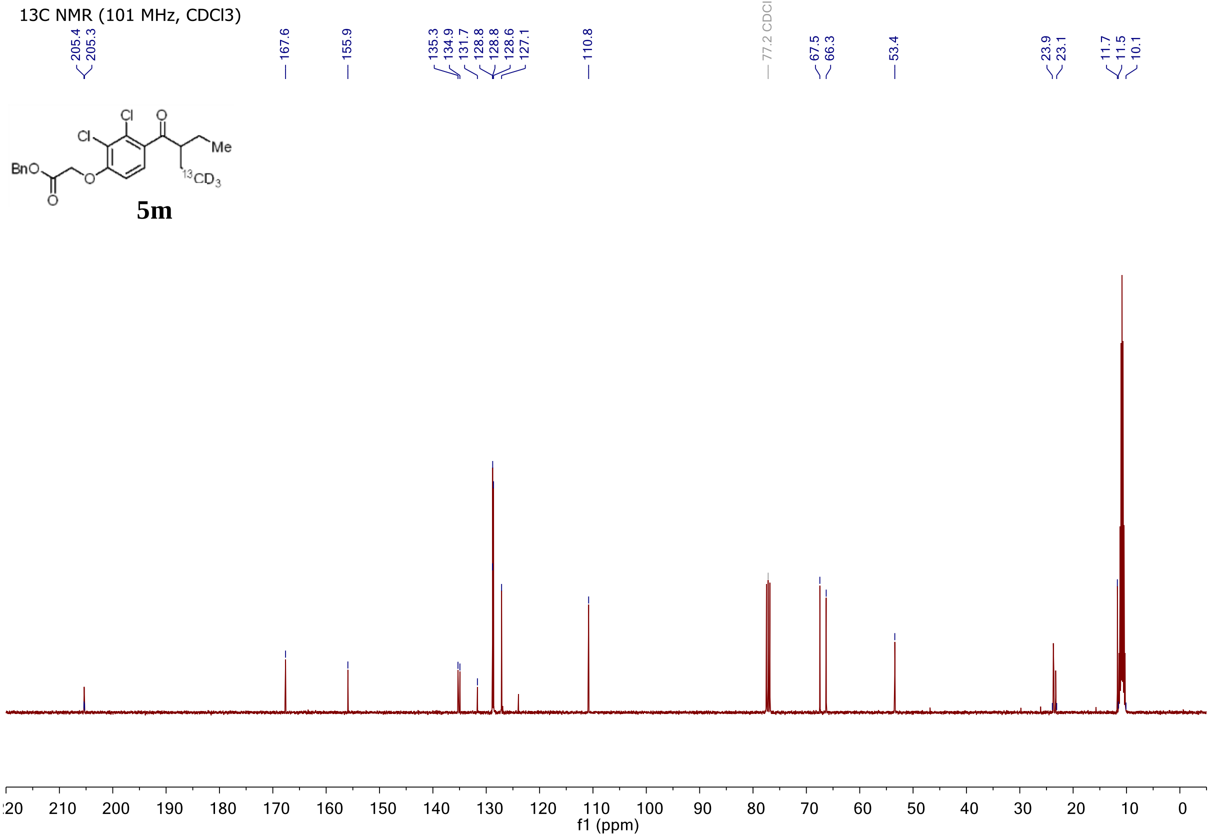

5m
